# Diarylamine Synthesis via Desulfinylative Smiles Rearrangement

**DOI:** 10.1021/acs.orglett.1c04122

**Published:** 2022-01-30

**Authors:** Thomas Sephton, Jonathan M. Large, Sam Butterworth, Michael F. Greaney

**Affiliations:** † Department of Chemistry, University of Manchester, Oxford Road, Manchester M13 9PL, U.K.; ‡ Accelerator Building, LifeArc, Open Innovation Campus, Stevenage SG1 2FX, U.K.; § Division of Pharmacy and Optometry, School of Health Sciences, Manchester Academic Health Sciences Centre, University of Manchester, Manchester M13 9PL, U.K.

## Abstract

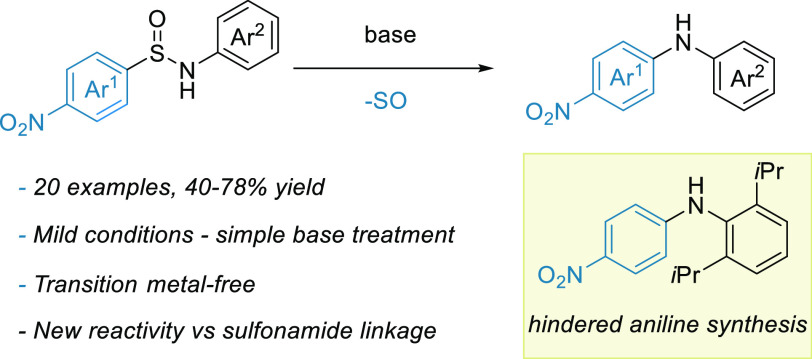

Diarylamines are obtained directly from sulfinamides through a novel rearrangement sequence. The transformation is transition metal-free and proceeds under mild conditions, providing facile access to highly sterically hindered diarylamines that are otherwise inaccessible by traditional S_N_Ar chemistry. The reaction highlights the distinct reactivity of the sulfinamide group in Smiles rearrangements versus that of the more common sulfonamides.

Diarylamines are important building blocks in organic synthesis and are present as privileged structures in numerous pharmaceuticals and biologically active compounds. Due to the moiety’s sustained importance to medicinal chemistry, many methods exist for diarylamine synthesis, with transition metal-catalyzed C–N bond formation being especially prominent in recent years.^1−3^ When the target diarylamine features an electron-deficient arene, a transition metal-free synthesis can be achieved by intermolecular nucleophilic aromatic substitution (S_N_Ar), which remains the third-most-used reaction in medicinal chemistry.^4^ However, S_N_Ar loses its utility when the substrate’s reactivity is attenuated by steric or electronic constraints or when the target molecule contains multiple reactive sites.

We were interested in harnessing the Smiles rearrangement as a potential route to diarylamines (Scheme 1). Smiles reactions are regiospecific, proceed under mild metal-free conditions, and can be used to construct very sterically hindered systems (Scheme 1A).^5^ The rearrangement has enjoyed a renaissance in recent years, offering new arylation pathways in both ionic and radical reaction manifolds without recourse to stoichiometric metals and attendant precious metal catalysis. One of the most common substrates utilized in contemporary Smiles chemistry is the sulfonamide^6^ because it is readily available and provides an entropically driven Smiles pathway via SO_2_ extrusion.

**Scheme 1:**
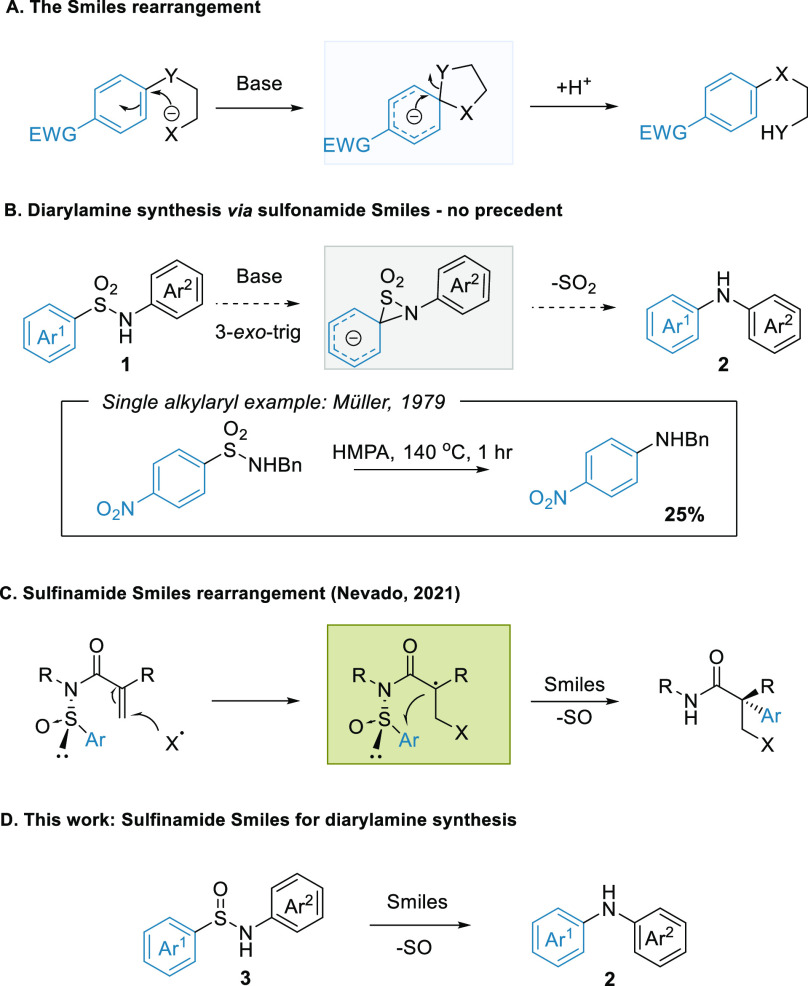
Smiles Rearrangements

Such a reaction could, in principle, be applied directly to diarylamine synthesis from diarylsulfonamides (Scheme 1B). The requisite 3-*exo*-trig *ipso* substitution pathway, however, is disfavored,^7^ and very few examples are known in the Smiles literature for any substrate class.^8^

There is no precedent for such reactivity with diarylsulfonamides in synthetic chemistry.^9^ Indeed, the functional group is valued for its stability to base. A single report does describe amine formation from an alkylarylsulfonamide, with Müller reporting the rearrangement of *N*-benzylnosylamide to the aniline in a low yield after heating to 140 °C in HMPA.^10^ Interestingly, the transformation is well-documented in the gas phase, with SO_2_ extrusion being an established fragmentation pathway for sulfonamides in mass spectrometry.^11^

As expected, our initial investigations underlined the difficulty of this transformation with diarylsulfonamides, with sulfonamide **1a** (Ar^1^ = *p*-NO_2_C_6_H_5_, Ar^2^ = Ph) failing to produce any diarylamine **2** upon base treatment even under forcing conditions (e.g., excess Cs_2_CO_3_ in refluxing DMA). We thus turned our attention to the sulfinamide group as a possible alternative Smiles substrate. Recent work from Nevado and co-workers has demonstrated that sulfinamides are productive in Smiles rearrangements, exploiting the chirality of the S(IV) functionality to achieve challenging stereoselective arylations (Scheme 1C).^12^ Outside of this work, however, sulfinamides have been underexplored both as substrates in Smiles rearrangements and in synthetic methodology in general. Their current utility is limited mostly to chiral auxiliaries, such as those developed by Davis and Ellman,^13,14^ or as intermediates in sulfonamide synthesis.^15^ We were interested in exploring possible reactivity differences between the sulfinamides and sulfonamides in aryl transfer and thus synthesized sulfinamide **3a** (Ar^1^ = *p*-NO_2_C_6_H_5_, Ar^2^ = Ph) to study as a potential diarylamine precursor.

We were surprised to find that **3a** did indeed produce the diarylamine **2a** in good yields upon base treatment under relatively mild conditions such as with K_2_CO_3_ in DMF at 60 °C (Table 1, entry 1).

**Table 1:**
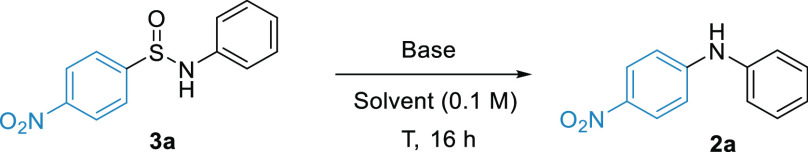
Reaction Optimization^a^

entry	base (equiv)	solvent	*T* (°C)	yield (%)^b^
1	K_2_CO_3_ (3)	DMF	60	54
2		DMF	60	0
3	LiOH (6)	DMF	60	74
4	Cs_2_CO_3_ (3)	DMF	60	74
5	NEt_3_ (3)	DMF	60	0
6	LiOH (6)	DMSO	60	70
7	LiOH (6)	DMA	60	64
8	LiOH (6)	DMF/H_2_O	60	71
9	LiOH (6)	THF	60	7
10	LiOH (6)	DMF	70	74 (71)^c^
11	Cs_2_CO_3_ (6)	DMF	70	74 (73)^c^
12	Cs_2_CO_3_ (6)	DMA	70	66^d^

a 0.05 mmol scale.

b ^1^H NMR yield.

c Isolated yield, 0.2 mmol scale.

d Microwave heating, 30 min reaction time.

Following an extensive screen of the reaction conditions, we found that the transformation proceeded with most inorganic bases tested, with LiOH and Cs_2_CO_3_ performing particularly well (entries 2–5). Furthermore, the rearrangement proceeded in a variety of solvent systems, including with the addition of water as a cosolvent; however, DMF proved the most effective (entries 6–11).

With the reaction conditions in hand, we then looked to examine the substrate scope of the system (Scheme 2). Beginning our investigation with the scope of the *N*-aryl group, we found that the system was tolerant to simple methyl-substituted rings at all positions (**2b**–**d**). Similar success was achieved with halogenated rings **2e**–**h**, which can be challenging to synthesize using transition metal catalysis.

**Scheme 2:**
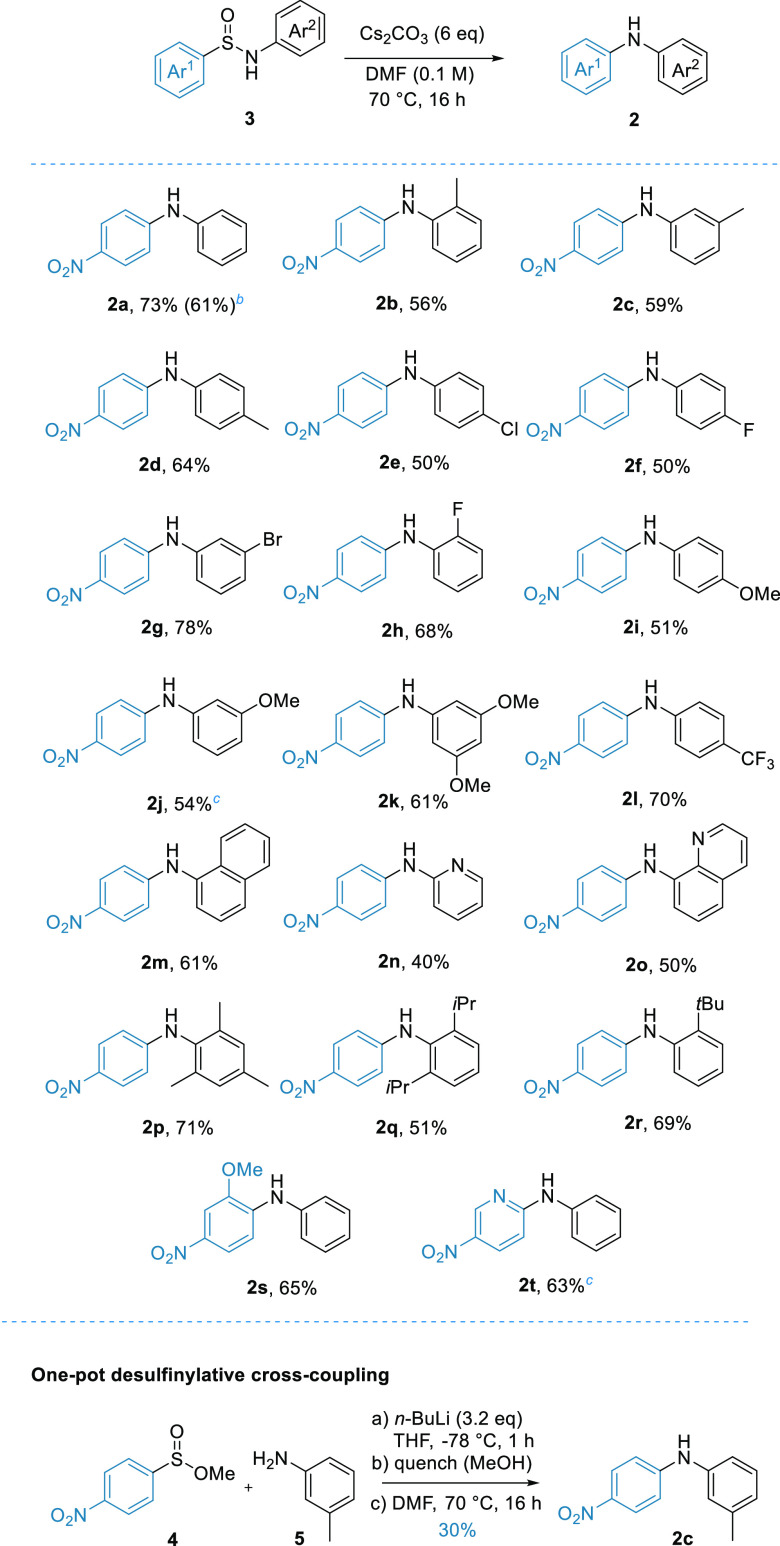
Substrate Scope Isolated yields, 0.2 mmol scale. 1.0 mmol scale. 0.1 mmol scale.

Additionally, the scope encompassed substrates featuring both electron-rich rings (**2i**–**k**) and relatively electron-poor ones (**2l**), although substrates with highly electron-deficient rings were unsuccessful (see the Supporting Information).

Furthermore, the reaction proved effective for alternative arenes, such as the *N*-naphthyl example **2m**, and heterocyclic compounds **2n** and **2o**. Importantly, and in line with literature precedent for desulfonylative Smiles processes,^16^ the system proved exceptionally tolerant to highly hindered substrates, affording diarylamines **2p**–**r**. For comparison, the treatment of *p*-nitrofluorobenzene with the analogous anilines under standard S_N_Ar conditions (K_2_CO_3_, DMF, 150 °C, and 16 h) failed to yield any amount of **2p**–**r**. The scope of the sulfonyl component was more restricted, with alternative electron-withdrawing groups such as *p-*CN, *p-*Cl, and pentafluoro being unsuccessful in the reaction. We could, however, successfully use an azine heterocycle in the reaction to afford the aminopyridine product **2t**. We were also able to develop a one-pot protocol utilizing a solvent swap to synthesize the target diarylamine directly from sulfinate **4** and aniline **5**. This result was especially encouraging, as it presented a strategy for a transition metal-free desulfinylative cross-coupling.

We then conducted experiments to elucidate the mechanism of the transformation (Scheme 3). As expected, the corresponding sulfonamide **1a** was an ineffective substrate under the optimized reaction conditions, returning only the starting material (Scheme 3A). We further established that *N*-alkylated substrates were similarly unsuccessful as they also solely afforded the starting material (Scheme 3B), supporting the idea that the deprotonation of the sulfinamide is vital to the reaction. A crossover experiment was then considered to probe possible intermolecular pathways, but the documented rapid exchange between *N*-aryl sulfinamides in solution^17^ would prevent a useful interpretation of the results. In view of this, we conducted a competition experiment for the rearrangement of **3a** in the presence of 4-methoxyaniline (Scheme 4C). No crossover product was detected, supporting an intramolecular mechanism.

**Scheme 3:**
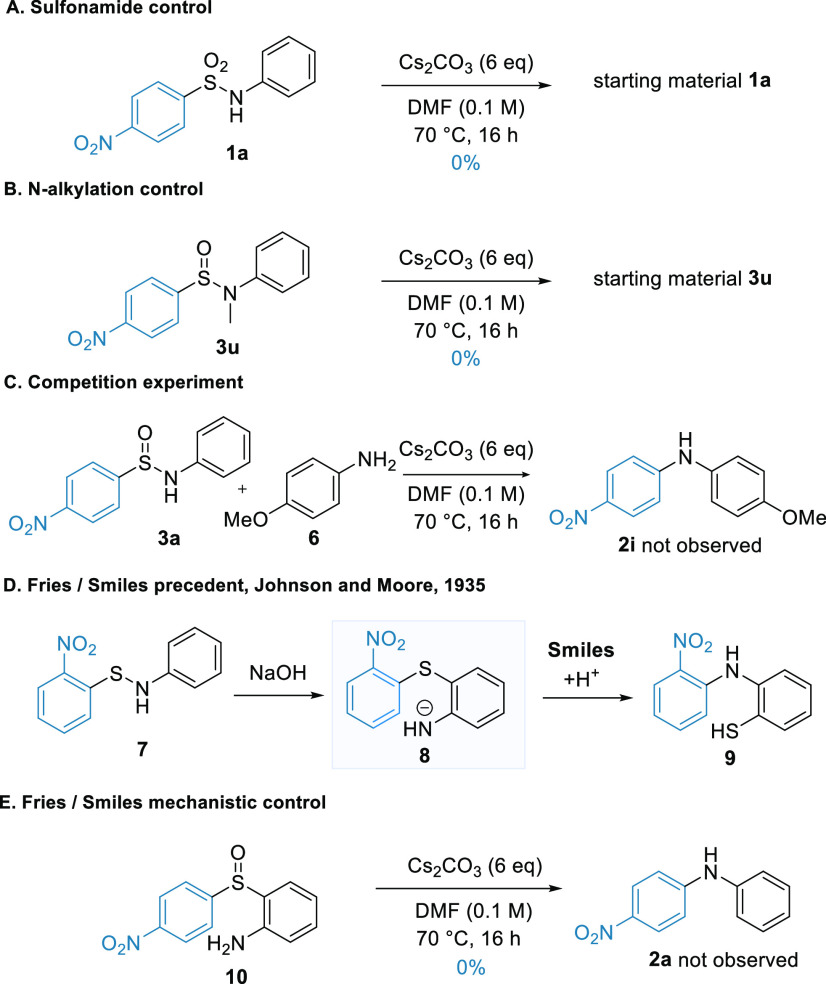
Mechanistic Investigations

**Scheme 4:**
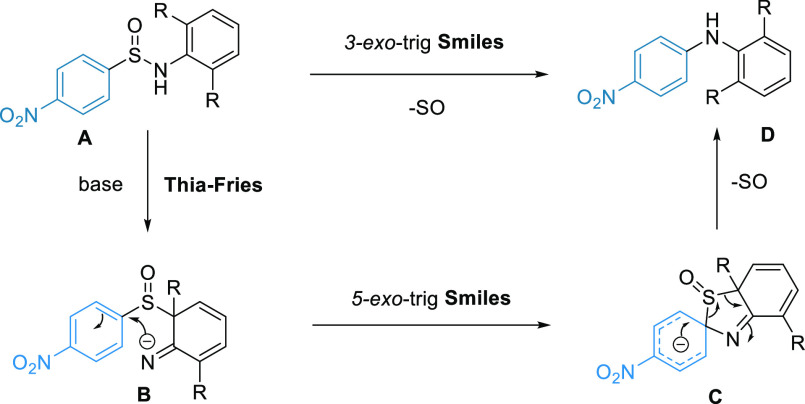
Proposed Mechanisms

We then considered the possibility of thia-Fries-type processes operating in the reaction. This reactivity is well-documented for sulfenamides, sulfinamides, and sulfonamides and can set up a prospective Smiles rearrangement to produce diarylamines (albeit with the C–S bond retained in the products).^18,19^ The seminal work from Johnson and Moore in 1935, for example, described the rearrangement of *ortho*-nitrophenylsulfenamide **7** into diarylamine **9** upon treatment with an alcoholic NaOH solution (Scheme 3D).^20^ The Smiles rearrangement of analogous sulfoxides and sulfones to **8** to give diarylamines is likewise known.^21−23^ A possible thia-Fries/SO extrusion pathway is illustrated in Scheme 4. To explore this possibility, we synthesized the aryl sulfoxide **10** and exposed it to our reaction conditions (Scheme 3E). No diarylamine product was detected, suggesting this thia-Fries product is not an intermediate in the rearrangement pathway.

Overall, these observations suggest the direct 3*-exo*-trig Smiles pathway to be the most plausible (**A** → **D**, Scheme 4) given the data in hand. While a thia-Fries/Smiles sequence (**A** → **B** → **C** → **D**) is conceivable and features a standard 5-*exo*-trig Smiles step, it requires an initial thia-Fries reaction to take place upon mild base treatment that will be dearomatizing in the case of *ortho*-substituted substrates. The failure of **10** to undergo the reaction, a tautomer of **B** for unsubstituted cases (R = H), lends further support to the direct 3-*exo*-trig pathway.

To conclude, we have described a transition metal-free desulfinylative diarylamine synthesis that proceeds under mild conditions and is especially successful in affording highly hindered products that were previously inaccessible by intermolecular S_N_Ar. A preliminary mechanistic survey points to the transformation proceeding via a novel desulfinylative 3-*exo*-trig Smiles rearrangement, a reactivity not observed with the more common sulfonamide functional group. Further investigations into the aryl transfer reactivity of sulfinamides are underway in our laboratory.

## Supporting Information Available

Preparative procedures and spectroscopic data for all starting materials and Smiles rearrangement products (PDF)
