# Supplementary material for: Diarylamine Synthesis via Desulfinylative Smiles Rearrangement
Source: Org Lett. 2022 Jan 30;24(5):1132–5. doi: 10.1021/acs.orglett.1c04122 (PMC8893360; doi:10.1021/acs.orglett.1c04122)
Supplement: Supplementary file 1 — ol1c04122_si_001.pdf [file ol1c04122_si_001.pdf]

Diarylamine synthesis *via* desulfinylative Smiles rearrangement

Thomas Sephton,[a] Jonathan M. Large,[b] Sam Butterworth,[c]* Michael F. Greaney[a]*

[a] *Dept. of Chemistry, University of Manchester, Oxford Rd, Manchester, M13 9PL, UK.*

[b] *LifeArc, Accelerator Building, Open Innovation Campus, Stevenage, SG1 2FX, UK.*

[c] *Division of Pharmacy and Optometry, School of Health Sciences, Manchester Academic Health Sciences Centre, University of Manchester, Manchester, M13 9PL, UK.*

**michael.greaney@manchester.ac.uk*

**sam.butterworth@manchester.ac.uk*

Supporting Information

1. General Remarks 2
2. General Procedures 3
3. Data for Synthesised Compounds 5
4. Poor-yielding and Unsuccessful Substrates 25
5. NMR Spectra for Synthesised Compounds 26
6. References 75

1. General Remarks

All solvents and reagents were purchased from Sigma Aldrich, Thermo Fisher Scientific, Apollo Scientific or Fluorochem and were used as received without further purification. Flash column chromatography was performed using either Biotage Snap Ultra cartridges or Biotage Sfar Silica cartridges on a Biotage Isolera automated column. ^{1}H, ^{13}C and ^{19}F NMR spectroscopy were recorded on either 400 MHz or 500 MHz Bruker Avance NMR spectrometers. Chemical shifts (δ) are reported in parts per million (ppm) and multiplicities are reported as either singlets (s), doublets (d), triplets (t), quartets (q) or multiplets (m). Coupling constants (*J*) are reported in Hertz (Hz). All ^{1}H NMR and ^{13}C NMR shifts were referenced to the residual solvent peak of $CDCl_3$ (^{1}H referenced to 7.26 ppm and ^{13}C referenced to 77.16 ppm), DMSO-d_6 (^{1}H referenced to 2.50 ppm and ^{13}C referenced to 39.52 ppm) and acetone-d_6 (^{1}H referenced to 2.06 ppm and ^{13}C referenced to 29.84 ppm). All ^{19}F chemical shifts were unadjusted from raw data. 2D heteronuclear single quantum coherence (HSQC), heteronuclear multiple bond correlation (HMBC) and homonuclear correlation spectroscopy (COSY) NMR spectroscopy was used to assist the assignment of signals. High resolution mass spectrometry (HRMS) was recorded on a Waters QTOF, using either ESI or APCI as ionisation methods. Thin layer chromatography (TLC) was carried out using commercially available coated TLC plates and spots were illuminated either by UV light (254 nm) or by staining the plate with a $KMnO_4$ solution. Compound names are those generated by ChemBioDraw^{TM} (CambridgeSoft) following International Union of Pure and Applied Chemistry (IUPAC) nomenclature. Novel compounds are labelled in *italics*. Melting points (MPs) were recorded on a Griffin melting point apparatus to the nearest degree. Reactions which proceeded under microwave irradiation were performed in a Biotage Initiator Microwave Synthesizer.

2. General Procedures

General Procedure A for the Synthesis of N-Aryl Sulfinamides

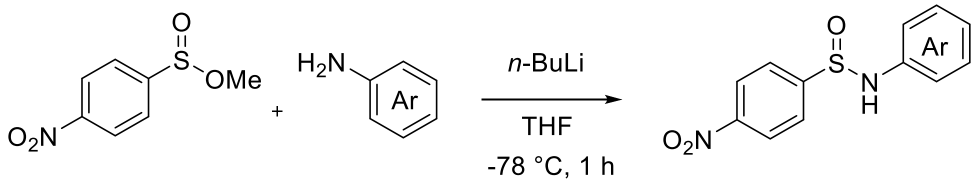

Carried out by adaptation of a literature procedure.[1] A 2-5 mL microwave vial was charged with the corresponding aniline (2.5 mmol) and the vial was evacuated under vacuum and filled with nitrogen. Anhydrous THF (2 mL) was added and the solution was stirred at -78 ̊C. *n*-BuLi (1.6 M in hexanes, 1 mL, 1.6 mmol) was added dropwise and the mixture was stirred at -78 ̊C for 20 minutes. A second 2-5 mL microwave vial was charged with methyl sulfinate **8** (200 mg, 2 mmol), evacuated under vacuum and filled with nitrogen. Dry THF (1.5 mL) was added, and the resulting solution was added dropwise to the reaction mixture, which was then stirred for 1-2 hours at -78 ̊C. The reaction mixture was then quenched with saturated aqueous $NaHCO_3$ (3 mL) and extracted with EtOAc (3 x 20 mL). The organic layer was then washed with saturated aqueous $NaHCO_3$ (3 x 20 mL) and dried with anhydrous $MgSO_4$. This was then filtered, and concentrated *in vacuo* to afford the crude product, which was purified by flash column chromatography.

General Procedure B for the Desulfinylative Smiles Rearrangement

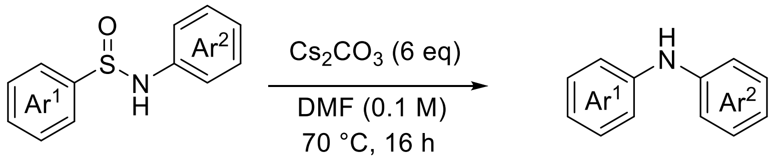

A 2-5 mL microwave vial was charged with the corresponding sulfinamide (0.2 mmol) and Cs_2CO_3 (392 mg, 1.2 mmol), evacuated under vacuum and filled with nitrogen. Anhydrous DMF (2 mL) was added, and the mixture was stirred in a pre-warmed oil bath at 70 ̊C for 16h. The vial was opened, the reaction mixture was dissolved in EtOAc (20 mL), washed with 1M aqueous HCl (2 x 20 mL) and 10% (w/v) aqueous LiCl (2 x 20 mL). The aqueous layer was then extracted with EtOAc (3 x 20 mL), and the combined organic layers were dried with anhydrous $MgSO_4$, filtered and concentrated *in vacuo*. The crude product was then purified by flash column chromatography, affording the pure product.

General Procedure C for the Intermolecular S_NAr Control Reaction

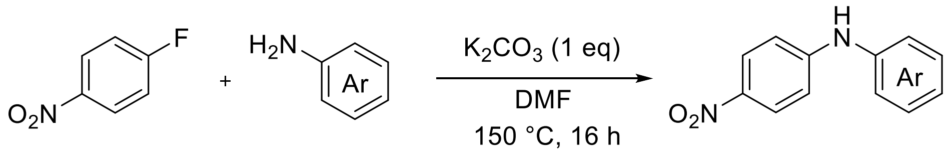

Carried out according to a literature procedure.[2] A 2-5 mL microwave vial was charged with 1-fluoro-4-nitrobenzene (141 mg, 1 mmol) and K_2CO_3 (140 mg, 1 mmol), evacuated under vacuum and filled with nitrogen. Anhydrous DMF (2 mL) and the corresponding aniline (1.1 mmol) were added and the reaction mixture was heated to reflux for 16 h. After 16 h, the reaction was monitored by TLC (30:70

EtOAc:hexane). No product was observed, and this was confirmed by NMR analysis of the crude product. No purification was necessary.

General Procedure D for the One-Pot Desulfinylative Cross-Coupling

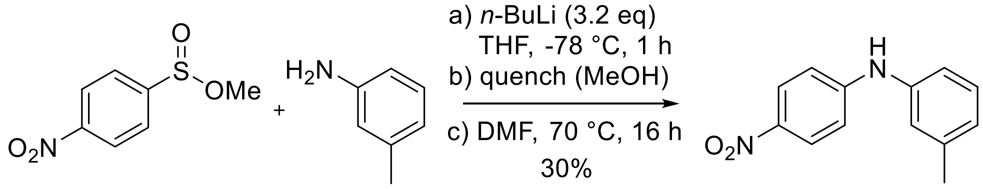

A 50 mL round-bottomed flask was charged with the corresponding aniline (2.5 mmol), evacuated under vacuum and filled with nitrogen. Anhydrous THF (2 mL) was added and the solution was stirred at -78 ̊C. n-BuLi (1.6 M in hexanes, 2 mL, 3.2 mmol) was added dropwise and the mixture was stirred at -78 ̊C for 20 minutes. A second 50 mL round-bottomed flask was charged with methyl sulfinate **8** (200 mg, 2 mmol), evacuated under vacuum and filled with nitrogen. Anhydrous THF (1.5 mL) was added, and the resulting solution was added dropwise to the reaction mixture, which was then stirred for 1 h at -78 ̊C. The reaction mixture was quenched with MeOH (3 mL) and the crude product was concentrated *in vacuo.* The flask was then filled with nitrogen, and the crude product dissolved in anhydrous DMF (5 mL). The flask was then stirred at 70°C in a pre-warmed oil bath for 16 h. After this, the mixture was extracted with EtOAc (3x 20 mL), washed with 1M aqueous HCl (2 x 20 mL) and 10% (w/v) aqueous LiCl (3 x 20 mL). The organic layers were combined and dried with anhydrous $MgSO_4$. This was filtered, and then concentrated *in vacuo* to afford the crude product, which was purified by flash column chromatography to afford the pure product.

General Procedure E for the Microwave Reaction

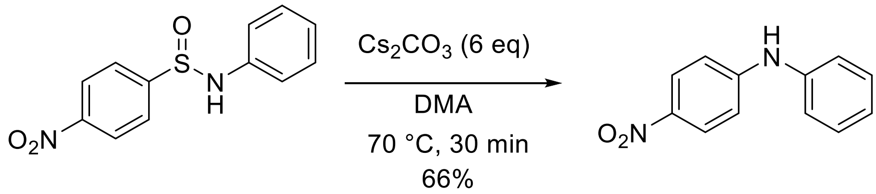

A sealed 2-5 mL microwave vial was charged with the corresponding sulfinamide (0.2 mmol) and Cs_2CO_3 (392 mg, 1.2 mmol), evacuated under vacuum and filled with nitrogen. Anhydrous DMA (2 mL) was added, and the mixture was placed in a Biotage Initiator Microwave Synthesizer at 70 °C for 30 min. The vial was then opened, the reaction mixture was dissolved in EtOAc (20 mL), washed with 1M aqueous HCl (2 x 20 mL) and 10% (w/v) aqueous LiCl (2 x 20 mL). The aqueous layer was then extracted with EtOAc (3 x 20 mL), and the combined organic layers were dried with anhydrous $MgSO_4$, filtered and concentrated *in vacuo*. The crude product was then purified by flash column chromatography, affording the pure product.

3. Data for Synthesised Compounds

Products from the Desulfinylative Smiles

4-nitro-N-phenylaniline (2a)

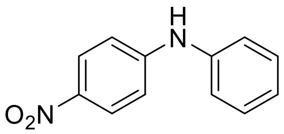

Synthesised according to general procedure B (column conditions: 0-30% EtOAc in hexane). The pure product was afforded as a yellow solid (31 mg, 73% yield).

1 mmol scale procedure:

A 50 mL round-bottomed flask was charged with sulfinamide **3a** (262 mg, 1 mmol) and Cs_2CO_3 (1.96 g, 6 mmol), evacuated under vacuum and filled with nitrogen. Anhydrous DMF (8 mL) was added and the reaction mixture was left to stir for in a pre-warmed oil bath at 70 °C for 16 h. the reaction mixture was dissolved in EtOAc (30 mL), washed with 1M aqueous HCl (2 x 40 mL) and 10% (w/v) aqueous LiCl (2 x 40 mL). The aqueous layer was then extracted with EtOAc (3 x 40 mL), and the combined organic layers were dried with anhydrous $MgSO_4$, filtered and concentrated *in vacuo*. The crude product was then purified by flash column chromatography, affording the pure product as a yellow solid (130 mg, 61% yield).

^{1}H NMR (500 MHz, $CDCl_3$) δ 8.12 (d, *J* = 9.2 Hz, 2H), 7.39 (t, *J* = 7.8 Hz, 2H), 7.21 (d, *J* = 7.9 Hz, 2H), 7.19 (t, *J* = 7.4 Hz, 1H), 6.94 (d, *J* = 9.2 Hz, 2H), 6.34 (s, 1H).

^{13}C NMR (126 MHz, $CDCl_3$) δ 150.2, 139.8, 139.5, 129.8, 126.3, 124.7, 122.0, 113.7.

HRMS (ESI) Calculated for $C_{12}H_{10}N_2O_2Na$: 237.0634. Found: 237.0627 $[M+Na]^+$

Data is in accordance with literature.[3]

2-methyl-N-(4-nitrophenyl)aniline (2b)

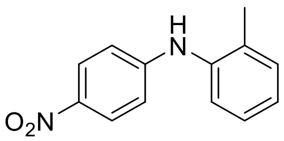

Synthesised according to general procedure B (column conditions: 5-30% EtOAc in hexane). The pure product was afforded as a yellow solid (26 mg, 56% yield).

^{1}H NMR (400 MHz, Acetone-d$_6$) δ 8.09 (d, *J* = 9.3 Hz, 2H), 8.02 (s, 1H), 7.38 – 7.15 (m, 4H), 6.85 (d, *J* = 9.2 Hz, 2H), 2.26 (s, 3H).

^{13}C NMR (101 MHz, Acetone-d$_6$) δ 153.5, 139.4, 139.2, 134.6, 132.2, 127.9, 126.8, 126.1, 113.6, 18.0.

HRMS (APCI) Calculated for $C_{13}H_{13}N_2O_2$: 229.0972. Found: 229.0964 $[M+H]^+$

Data is in accordance with literature.[4]

3-methyl-N-(4-nitrophenyl)aniline (2c)

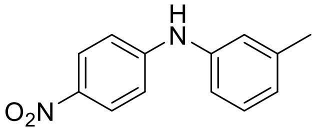

Synthesised according to general procedure B (column conditions: 5-30% EtOAc in hexane). The pure product was afforded as a yellow solid (27 mg, 59% yield).

^{1}H NMR (400 MHz, Acetone-d$_6$) δ 8.36 (s, 1H), 8.11 (d, *J* = 9.2 Hz, 2H), 7.28 (t, *J* = 7.7 Hz, 1H), 7.18 – 7.07 (m, 4H), 7.00 – 6.93 (m, 1H), 2.34 (s, 3H).

^{13}C NMR (101 MHz, Acetone-d$_6$) δ 152.0, 141.2, 140.2, 139.9, 130.2, 126.8, 125.4, 122.8, 119.3, 114.4, 21.4.

HRMS (APCI) Calculated for $C_{13}H_{13}N_2O_2$: 229.0972. Found: 229.0964 $[M+H]^+$

Data is in accordance with literature.[4]

4-methyl-N-(4-nitrophenyl)aniline (2d)

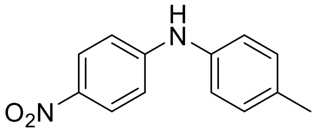

Synthesised according to general procedure B (column conditions: 0-30% EtOAc in hexane). The pure product was afforded as an orange solid (29 mg, 64% yield).

^{1}H NMR (400 MHz, CDCl$_3$) δ 8.10 (d, *J* = 9.1 Hz, 2H), 7.20 (d, *J* = 8.3 Hz, 2H), 7.10 (d, *J* = 8.0 Hz, 2H), 6.87 (d, *J* = 9.3 Hz, 2H), 6.29 (s, 1H), 2.36 (s, 3H).

^{13}C NMR (101 MHz, CDCl$_3$) δ 151.0, 139.5, 136.8, 134.9, 130.4, 126.4, 122.8, 113.3, 21.1.

HRMS (ESI) Calculated for $C_{13}H_{11}N_2O_2$: 227.0821 Found: 227.0815 $[M-H]^-$

Data is in accordance with literature.[4]

4-chloro-N-(4-nitrophenyl)aniline (2e)

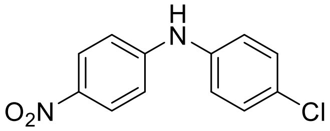

Synthesised according to general procedure B (column conditions: 0-30% EtOAc in hexane). The pure product was afforded as an orange solid (25 mg, 50% yield).

^{1}**H NMR** (500 MHz, $CDCl_3$) δ 8.14 (d, *J* = 9.2 Hz, 2H), 7.35 (d, *J* = 8.8 Hz, 2H), 7.15 (d, *J* = 8.8 Hz, 2H), 6.92 (d, *J* = 9.3 Hz, 2H), 6.21 (s, 1H).

^{13}**C NMR** (101 MHz, $CDCl_3$) δ 149.8, 140.3, 138.3, 130.0, 129.8, 126.4, 123.2, 114.0.

HRMS (ESI) Calculated for $C_{12}H_8ClN_2O_2$: 247.0280. Found: 247.0266 $[M-H]^-$

Data is in accordance with literature.[4]

4-fluoro-N-(4-nitrophenyl)aniline (2f)

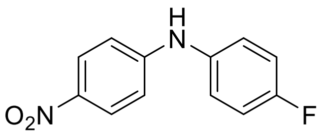

Synthesised according to general procedure B (column conditions: 0-30% EtOAc in hexane). The pure product was afforded as an orange solid (23 mg, 50% yield).

^{1}**H NMR** (400 MHz, $CDCl_3$) δ 8.11 (d, *J* = 9.4 Hz, 2H), 7.22 – 7.16 (m, 2H), 7.13 – 7.05 (m, 2H), 6.83 (d, *J* = 9.5 Hz, 2H), 6.16 (s, 1H).

^{13}**C NMR** (101 MHz, $CDCl_3$) δ 160.2 (d, *J* = 245.0 Hz), 150.8, 139.9, 135.5 (d, *J* = 2.8 Hz), 126.4, 125.0 (d, *J* = 8.0 Hz), 116.6 (d, *J* = 22.6 Hz), 113.3.

^{19}**F NMR** (376 MHz, $CDCl_3$) δ -116.63 (s).

HRMS (APCI) Calculated for $C_{12}H_9FN_2O_2$: 232.0643. Found: 232.0646 $[M]^+$

Data is in accordance with literature.[4]

3-bromo-N-(4-nitrophenyl)aniline (2g)

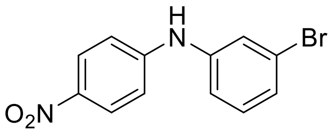

Synthesised according to general procedure B (column conditions: 0-30% EtOAc in hexane). The pure product was afforded as an orange solid (46 mg, 78% yield).

^{1}**H NMR** (400 MHz, Acetone-d_6) δ 8.55 (s, 1H), 8.15 (d, *J* = 9.2 Hz, 2H), 7.47 (s, 1H), 7.37 – 7.31 (m, 2H), 7.29 – 7.24 (m, 1H), 7.22 (d, *J* = 9.4 Hz, 2H).

^{13}**C NMR** (101 MHz, Acetone-d_6) δ 150.8, 143.4, 140.8, 132.1, 126.9, 126.7, 123.9, 123.4, 120.0, 115.4.

HRMS (APCI) Calculated for $C_{12}H_{10}BrN_2O_2$: 292.9920. Found: 292.9922 $[M+H]^+$

Data is in accordance with literature.[4]

2-fluoro-N-(4-nitrophenyl)aniline (2h)

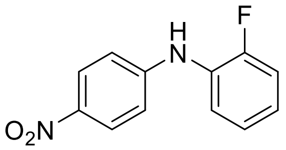

Synthesised according to general procedure B (column conditions: 0-30% EtOAc in hexane). The pure product was afforded as a yellow solid (32 mg, 68% yield).

^{1}H NMR (400 MHz, Acetone-d$_6$) δ 8.24 (s, 1H), 8.14 (d, *J* = 9.2 Hz, 2H), 7.55 – 7.46 (m, 1H), 7.35 – 7.19 (m, 3H), 7.06 (d, *J* = 8.5 Hz, 2H).

^{13}C NMR (101 MHz, Acetone-d$_6$) δ 156.9 (d, *J* = 245.3 Hz), 151.9, 140.4, 128.9 (d, *J* = 11.8 Hz), 126.7 (d, *J* = 7.9 Hz), 126.6, 25.9 (d, *J* = 3.7 Hz), 125.5 (d, *J* = 1.7 Hz), 117.3 (d, *J* = 19.8 Hz), 114.6.

^{19}F NMR (376 MHz, Acetone-d$_6$) δ -124.76 (s).

HRMS (ESI) Calculated for $C_{12}H_9FN_2O_2Na$: 255.0546. Found: 255.0540 $[M+Na]^+$

4-methoxy-N-(4-nitrophenyl)aniline (2i)

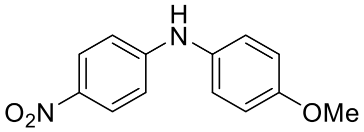

Synthesised according to general procedure B (column conditions: 0-30% EtOAc in hexane). The pure product was afforded as a red solid (25 mg, 51% yield).

^{1}H NMR (400 MHz, CDCl$_3$) δ 8.08 (d, *J* = 9.2 Hz, 2H), 7.16 (d, *J* = 9.0 Hz, 2H), 6.94 (d, *J* = 8.9 Hz, 2H), 6.75 (d, *J* = 9.2 Hz, 2H), 6.15 (s, 1H), 3.83 (s, 3H).

^{13}C NMR (101 MHz, CDCl$_3$) δ 157.6, 151.8, 139.2, 132.1, 126.5, 125.7, 115.1, 112.8, 55.7.

HRMS (ESI) Calculated for $C_{13}H_{12}N_2O_2Na$: 267.0740. Found: 267.0736 $[M+Na]^+$

Data is in accordance with literature.[4]

3-methoxy-N-(4-nitrophenyl)aniline (2j)

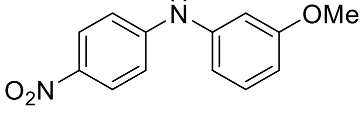

Synthesised according to general procedure B, on a 0.1 mmol scale (column conditions: 0-30% EtOAc in hexane). The pure product was afforded as an orange solid (9 mg, 54% yield).

^{1}H NMR (500 MHz, CDCl$_3$) δ 8.12 (d, *J* = 9.2 Hz, 2H), 7.29 (t, *J* = 8.1 Hz, 1H), 6.97 (d, *J* = 9.1 Hz, 2H), 6.81 – 6.78 (m, 1H), 6.75 (t, *J* = 2.3 Hz, 1H), 6.73 – 6.68 (m, 1H), 3.82 (s, 3H).

^{13}C NMR (126 MHz, CDCl$_3$) δ 160.9, 150.0, 140.9, 140.1, 130.7, 126.4, 114.2, 114.1, 109.9, 107.8, 55.5.

HRMS (ESI) Calculated for $C_{13}H_{12}N_2O_2Na$: 267.0740. Found: 267.0736 $[M+Na]^+$

Data is in accordance with literature.[4]

3,5-dimethoxy-N-(4-nitrophenyl)aniline (2k)

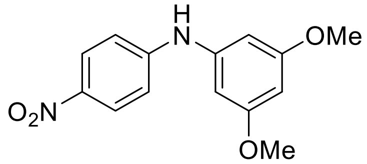

Synthesised according to general procedure B (column conditions: 0-30% EtOAc in hexane). The pure product was afforded as a red solid (33 mg, 61% yield).

^{1}H NMR (500 MHz, DMSO-d$_6$) δ 9.27 (s, 1H), 8.09 (d, *J* = 9.5 Hz, 2H), 7.10 (d, *J* = 9.5 Hz, 2H), 6.37 (d, *J* = 2.2 Hz, 2H), 6.25 (t, *J* = 2.2 Hz, 1H), 3.74 (s, 6H).

^{13}C NMR (126 MHz, DMSO-d$_6$) δ 161.2, 150.5, 141.9, 138.1, 126.1, 114.0, 98.7, 95.3, 55.2.

HRMS (APCI) Calculated for $C_{14}H_{15}N_2O_4$: 275.1026. Found: 275.1015 $[M+H]^+$

MP 150-152 °C

4-nitro-N-(4-(trifluoromethyl)phenyl)aniline (2l)

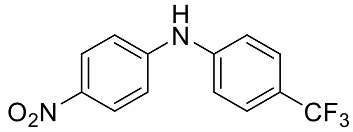

Synthesised according to general procedure B (column conditions: 0-30% EtOAc in hexane). The pure product was afforded as a yellow solid (40 mg, 70% yield).

^{1}H NMR (400 MHz, Acetone-d$_6$) δ 8.76 (s, 1H), 8.18 (d, *J* = 8.8 Hz, 2H), 7.70 (d, *J* = 8.6 Hz, 2H), 7.48 (d, *J* = 8.3 Hz, 2H), 7.33 (d, *J* = 9.2 Hz, 2H).

^{13}C NMR (101 MHz, Acetone-d$_6$) δ 150.0, 145.5, 141.4, 127.6 (q, *J* = 3.8 Hz), 126.6, 124.3 (q, *J* = 32.6 Hz), 121.5, 120.0, 116.3.

^{19}F NMR (376 MHz, Acetone-d$_6$) δ -62.28 (s).

HRMS (APCI) Calculated for $C_{13}H_{10}F_3N_2O_2$: 283.0689. Found: 283.0686 $[M+H]^+$

Data is in accordance with literature.[5]

N-(4-nitrophenyl)naphthalen-1-amine (2m)

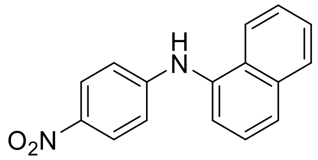

Synthesised according to general procedure B (column conditions: 0-25% EtOAc in hexane). The pure product was afforded as a brown solid (33 mg, 61% yield).

^{1}H NMR (400 MHz, Acetone-d$_6$) δ 9.41 (s, 1H), 8.06 (d, *J* = 9.0 Hz, 2H), 8.02 – 7.96 (m, 2H), 7.84 (d, *J* = 7.9 Hz, 1H), 7.62 – 7.48 (m, 4H), 6.89 (d, *J* = 9.4 Hz, 2H).

^{13}C NMR (101 MHz, Acetone-d$_6$) δ 153.0, 137.6, 135.6, 134.4, 128.8, 128.4, 126.5, 126.3, 126.2, 126.1, 125.7, 122.8, 121.3, 113.0.

HRMS (ESI) Calculated for $C_{16}H_{11}N_2O_2$: 263.0815. Found: 263.0812 $[M-H]^-$

Data is in accordance with literature.[4]

N-(4-nitrophenyl)pyridin-2-amine (2n)

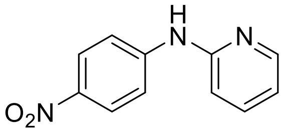

Synthesised according to general procedure B (column conditions: 0-50% EtOAc in hexane). The pure product was afforded as an orange solid (17 mg, 40% yield).

^{1}H NMR (500 MHz, Acetone-d$_6$) δ 9.12 (s, 1H), 8.36 – 8.31 (m, 1H), 8.18 (d, *J* = 9.4 Hz, 2H), 8.01 (d, *J* = 9.3 Hz, 2H), 7.69 (t, *J* = 8.7 Hz, 1H), 7.01 (d, *J* = 8.3 Hz, 1H), 6.97 – 6.93 (m, 1H).

^{13}C NMR (126 MHz, Acetone-d$_6$) δ 154.9, 148.2, 147.4, 140.3, 137.8, 125.0, 116.7, 116.6, 112.2.

HRMS (ESI) Calculated for $C_{13}H_9N_3O_2Na$: 238.0587. Found: 238.0581 $[M+Na]^+$

Data is in accordance with literature.[6]

N-(4-nitrophenyl)quinolin-8-amine (2o)

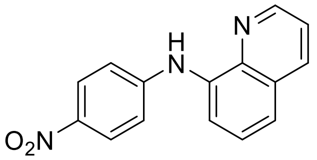

Synthesised according to general procedure B (column conditions: 0-50% EtOAc in hexane). The pure product was afforded as an orange solid (26 mg, 50% yield).

^{1}H NMR (400 MHz, Acetone-d$_6$) δ 9.19 (s, 1H), 8.90 (dd, *J* = 4.2, 1.7 Hz, 1H), 8.38 (dd, *J* = 8.3, 1.7 Hz, 1H), 8.25 (d, *J* = 9.1 Hz, 2H), 7.87 (dd, *J* = 5.9, 2.9 Hz, 1H), 7.69 – 7.55 (m, 5H).

^{13}C NMR (101 MHz, Acetone-d$_6$) δ 150.0, 149.4, 140.4, 138.1, 137.4, 129.9, 127.8, 126.6, 123.2, 121.3, 116.9, 116.9, 113.8.

HRMS (ESI) Calculated for $C_{15}H_{11}N_3O_2Na$: 288.0743. Found: 288.0731 $[M+Na]^+$

Data is in accordance with literature.[4]

2,4,6-trimethyl-N-(4-nitrophenyl)aniline (2p)

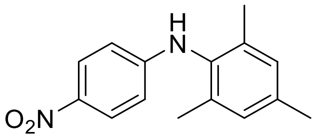

Synthesised according to general procedure B (column conditions: 0-25% EtOAc in hexane). The pure product was afforded as a yellow solid (37 mg, 71% yield).

^{1}H NMR (500 MHz, $CDCl_3$) δ 8.06 (d, *J* = 8.7 Hz, 2H), 6.97 (s, 2H), 6.44 (d, *J* = 8.7 Hz, 2H), 5.86 (s, 1H), 2.32 (s, 3H), 2.16 (s, 6H).

^{13}C NMR (126 MHz, $CDCl_3$) δ 152.6, 138.7, 137.5, 136.5, 133.1, 129.6, 126.6, 111.7, 21.1, 18.2.

HRMS (APCI) Calculated for $C_{15}H_{17}N_2O_2$: 257.1285. Found: 257.1272 $[M+H]^+$

MP 125-127 ˚C

2,6-diisopropyl-N-(4-nitrophenyl)aniline (2q)

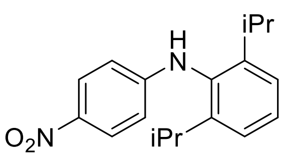

Synthesised according to general procedure B (column conditions: 0-25% EtOAc in hexane). The pure product was afforded as a yellow solid (31 mg, 51% yield).

^{1}H NMR (400 MHz, DMSO-d_6) δ 8.76 (s, 1H), 7.99 (d, *J* = 34.0 Hz, 2H), 7.39 – 7.32 (m, 1H), 7.27 (d, *J* = 7.1 Hz, 2H), 6.99 (s, 1H), 5.90 (s, 1H), 3.00 (h, *J* = 6.9 Hz, 2H), 1.13 (d, *J* = 6.8 Hz, 6H), 1.05 (d, *J* = 6.9 Hz, 6H).

^{13}C NMR (101 MHz, DMSO-d_6) δ 154.8, 146.9, 136.4, 133.2, 128.2, 126.8, 124.0, 113.2, 108.8, 27.9, 24.1, 23.0.

HRMS (APCI) Calculated for $C_{20}H_{25}N_2O_2$: 297.1603. Found: 297.1609 $[M-H]^-$

MP 140-142 ˚C

2-(tert-butyl)-N-(4-nitrophenyl)aniline (2r)

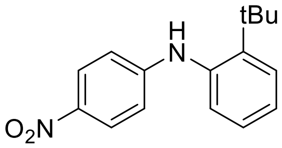

Synthesised according to general procedure B (column conditions: 0-30% EtOAc in hexane). The pure product was afforded as an orange solid (38 mg, 69% yield).

^{1}H NMR (500 MHz, DMSO-d_6) δ 8.64 (s, 1H), 8.01 (d, *J* = 9.3 Hz, 2H), 7.55 – 7.48 (m, 1H), 7.34 – 7.27 (m, 2H), 7.16 – 7.09 (m, 1H), 6.58 (d, *J* = 9.3 Hz, 2H), 1.31 (s, 9H).

^{13}C NMR (126 MHz, DMSO-d$_6$) δ 154.6, 147.7, 138.1, 136.6, 130.9, 127.6, 127.5, 127.4, 126.2, 112.3, 34.9, 30.6.

HRMS (APCI) Calculated for $C_{16}H_{19}N_2O_2$: 271.1441. Found: 271.1435 $[M+H]^+$

MP 91-94 ̊C

2-methoxy-4-nitro-N-phenylaniline (2s)

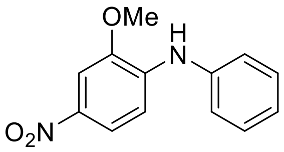

Synthesised according to general procedure B (column conditions: 0-30% EtOAc in hexane). The pure product was afforded as a yellow solid (32 mg, 65% yield).

^{1}H NMR (400 MHz, $CDCl_3$) δ 7.80 – 7.70 (m, 1H), 7.66 (s, 1H), 7.31 (t, *J* = 8.1 Hz, 2H), 7.21 – 7.13 (m, 2H), 7.12 – 7.06 (m, 1H), 7.04 (d, *J* = 9.0 Hz, 1H), 3.93 (s, 3H).

^{13}C NMR (101 MHz, $CDCl_3$) δ 146.0, 140.8, 139.5, 139.0, 129.8, 124.6, 122.2, 119.2, 109.5, 105.7, 56.3.

HRMS (APCI) Calculated for $C_{13}H_{13}N_2O_2$: 245.0921. Found: 245.0914 $[M+H]^+$

Data is in accordance with literature.[7]

5-nitro-N-phenylpyridin-2-amine (2t)

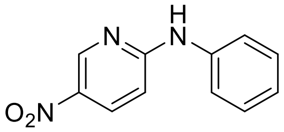

Synthesised according to general procedure B, 0.1 mmol scale (column conditions: 0-40% EtOAc in hexane). The pure product was afforded as a yellow solid (14 mg, 63% yield).

^{1}H NMR (500 MHz, Acetone-d$_6$) δ 9.07 (d, *J* = 2.7 Hz, 1H), 8.31 (dd, *J* = 9.3, 2.8 Hz, 1H), 7.76 (d, *J* = 8.1 Hz, 2H), 7.39 (t, *J* = 7.6 Hz, 2H), 7.13 (t, *J* = 7.4 Hz, 1H), 6.99 (d, *J* = 9.3 Hz, 1H).

^{13}C NMR (126 MHz, Acetone-d$_6$) δ 160.0, 146.3, 140.3, 137.7, 133.5, 129.8, 124.6, 121.5, 110.4.

HRMS (APCI) Calculated for $C_{11}H_{10}N_3O_2$: 216.0768. Found: 216.0761 $[M+H]^+$

Data is in accordance with literature.[8]

Sulfinamide Starting Materials

4-nitro-N-phenylbenzenesulfinamide (3a)

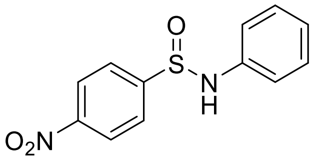

Prepared according to general procedure A, 4 mmol scale (column conditions: 0-40% EtOAc in hexane). The pure product was afforded as a yellow solid (519 mg, 49% yield).

^{1}H NMR (500 MHz, DMSO-d$_6$) δ 9.59 (s, 1H), 8.40 (d, *J* = 9.1 Hz, 2H), 7.98 (d, *J* = 9.1 Hz, 2H), 7.25 (t, *J* = 7.7 Hz, 2H), 7.07 (d, *J* = 7.4 Hz, 2H), 6.97 (t, *J* = 7.4 Hz, 1H).

^{13}C NMR (126 MHz, DMSO-d$_6$) δ 151.5, 149.1, 141.2, 129.4, 127.4, 124.2, 122.7, 118.2.

HRMS (ESI) Calculated for $C_{12}H_9N_2O_3S$: 261.0339. Found: 261.0334 $[M-H]^-$

Data is in accordance with literature.[9]

4-nitro-N-(o-tolyl)benzenesulfinamide (3b)

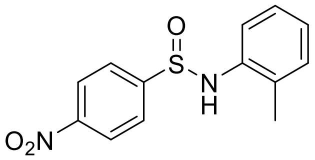

Prepared according to general procedure A (column conditions: 5-40% EtoAc in hexane). The pure product was afforded as an orange solid (83 mg, 30% yield).

^{1}H NMR (500 MHz, Acetone-d$_6$) δ 8.40 (d, *J* = 9.0 Hz, 2H), 8.12 – 8.03 (m, 3H), 7.39 – 7.33 (m, 1H), 7.19 (d, *J* = 7.2 Hz, 1H), 7.11 (t, *J* = 7.7 Hz, 1H), 7.01 (t, *J* = 7.5 Hz, 1H), 2.32 (s, 3H).

^{13}C NMR (126 MHz, Acetone-d$_6$) δ 153.2, 150.5, 140.1, 131.7, 131.0, 128.3, 127.5, 125.3, 124.7, 122.6, 18.2.

HRMS (APCI) Calculated for $C_{13}H_{13}N_2O_3S$: 277.0641. Found: 277.0631 $[M+H]^+$

MP 120-123 ˚C

4-nitro-N-(m-tolyl)benzenesulfinamide (3c)

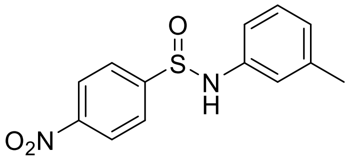

Prepared according to general procedure A (column conditions: 5-40% EtOAc in hexane). The pure product was afforded as a yellow solid (202 mg, 73% yield).

1H NMR (500 MHz, Acetone-d_6) δ 8.45 – 8.39 (m, 3H), 8.07 (d, J = 8.2 Hz, 2H), 7.15 (t, J = 7.8 Hz, 1H), 7.02 (s, 1H), 6.97 (d, J = 8.5 Hz, 1H), 6.86 (d, J = 7.5 Hz, 1H), 2.28 (s, 3H).

^{13}C NMR (126 MHz, Acetone-d_6) δ 153.1, 150.5, 142.2, 140.0, 130.0, 128.2, 124.8, 124.8, 120.2, 116.8, 21.4.

HRMS (APCI) Calculated for $C_{13}H_{13}N_2O_3S$: 277.0641. Found: 277.0634 $[M+H]^+$

MP 119-121 ˚C

4-nitro-N-(p-tolyl)benzenesulfinamide (3d)

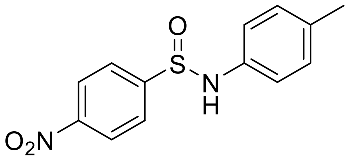

Synthesised according to general procedure A (column conditions: 5-40% EtOAc in hexane). The pure product was afforded as a yellow solid (185 mg, 67% yield).

^{1}H NMR (400 MHz, Acetone-d_6) δ 8.39 (d, *J* = 8.8 Hz, 2H), 8.37 (s, 1H), 8.06 (d, *J* = 8.8 Hz, 2H), 7.12 – 7.02 (m, 4H), 2.25 (s, 3H).

^{13}C NMR (101 MHz, Acetone-d_6) δ 153.2, 150.5, 139.4, 133.7, 130.6, 128.2, 124.8, 120.5, 20.7.

HRMS (ESI) Calculated for $C_{13}H_{11}N_2O_3S$: 275.0496. Found: 275.0487 $[M-H]^-$

Data is in accordance with literature.[10]

N-(4-chlorophenyl)-4-nitrobenzenesulfinamide (3e)

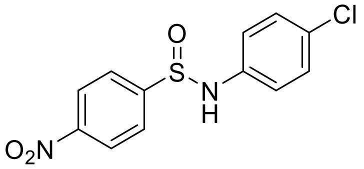

Synthesised according to general procedure A (column conditions: 5-40% EtOAc in hexane). The pure product was afforded as a red solid (85 mg, 29% yield).

^{1}H NMR (400 MHz, Acetone-d_6) δ 8.66 (s, 1H), 8.43 (d, *J* = 9.1 Hz, 2H), 8.07 (d, *J* = 9.1 Hz, 2H), 7.29 (d, *J* = 9.1 Hz, 2H), 7.19 (d, *J* = 8.9 Hz, 2H).

^{13}C NMR (101 MHz, Acetone-d_6) δ 152.6, 150.6, 141.0, 130.1, 128.7, 128.2, 124.9, 121.4.

HRMS (ESI) Calculated for $C_{12}H_8ClN_2O_3S$: 294.9950. Found: 294.9942 $[M-H]^-$

MP 154-156 ˚C

N-(4-fluorophenyl)-4-nitrobenzenesulfinamide (3f)

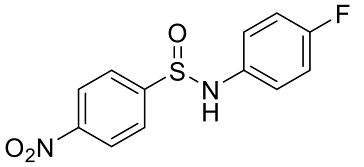

Synthesised according to general procedure A (column conditions: 5-40% EtOAc in hexane). The pure product was afforded as a yellow solid (144 mg, 52% yield).

^{1}H NMR (400 MHz, Acetone-d_6) δ 8.51 (s, 1H), 8.41 (d, *J* = 8.9 Hz, 2H), 8.06 (d, *J* = 8.9 Hz, 2H), 7.25 – 7.15 (m, 2H), 7.11 – 7.00 (m, 2H).

^{13}C NMR (101 MHz, Acetone-d_6) δ 159.2 (d, *J* = 240.2 Hz), 151.8, 149.6, 137.0, 127.3, 123.9, 121.8 (d, *J* = 8.2 Hz), 115.7 (d, *J* = 22.9 Hz).

^{19}F NMR (376 MHz, Acetone-d_6) δ -121.16 (s).

HRMS (APCI) Calculated for $C_{12}H_{10}FN_2O_3S$: 281.0391. Found: 281.0383 $[M+H]^+$

MP 132-134 ˚C

N-(3-bromophenyl)-4-nitrobenzenesulfinamide (3g)

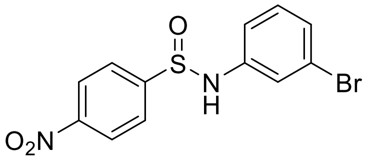

Synthesised according to general procedure A (column conditions: 5-40% EtOAc in hexane). The pure product was afforded as a yellow solid (241 mg, 71% yield).

^{1}H NMR (500 MHz, DMSO-d_6) δ 9.81 (s, 1H), 8.41 (d, *J* = 8.5 Hz, 2H), 8.00 (d, *J* = 8.9 Hz, 2H), 7.25 (t, *J* = 2.0 Hz, 1H), 7.21 (t, *J* = 7.9 Hz, 1H), 7.18 – 7.13 (m, 1H), 7.11 – 7.05 (m, 1H).

^{13}C NMR (126 MHz, DMSO-d_6) δ 151.0, 149.3, 143.1, 131.4, 127.4, 125.2, 124.2, 122.1, 120.3, 116.8.

HRMS (APCI) Calculated for $C_{12}H_8BrN_2O_3S$: 338.9444. Found: 338.9442 $[M-H]^-$

MP 108-109 ˚C

N-(2-fluorophenyl)-4-nitrobenzenesulfinamide (3h)

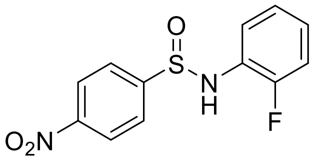

Synthesised according to general procedure A (column conditions: 5-40% EtOAc in hexane). The pure product was afforded as an off-white solid (189 mg, 68% yield).

^{1}H NMR (500 MHz, DMSO-d$_6$) δ 9.59 (s, 1H), 8.39 (d, *J* = 9.1 Hz, 2H), 7.95 (d, *J* = 8.8 Hz, 2H), 7.31 – 7.15 (m, 2H), 7.09 – 7.00 (m, 2H).

^{13}C NMR (126 MHz, DMSO-d$_6$) δ 153.7 (d, *J* = 243.6 Hz), 151.2, 149.1, 128.2 (d, *J* = 12.6 Hz), 127.4, 124.8 – 124.6 (m), 124.1, 122.2 (d, *J* = 1.9 Hz), 116.0 (d, *J* = 19.2 Hz).

^{19}F NMR (471 MHz, DMSO-d$_6$) δ -126.15 – -126.23 (m).

HRMS (APCI) Calculated for $C_{12}H_{10}FN_2O_3S$: 281.0391. Found: 281.0387 $[M+H]^+$

MP 142-144 °C

N-(4-methoxyphenyl)-4-nitrobenzenesulfinamide (3i)

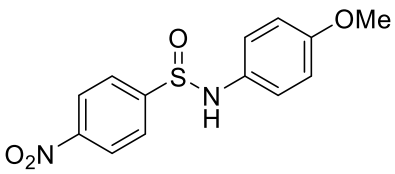

Synthesised according to general procedure A (column conditions: 5-40% EtOAc in hexane). The pure product was afforded as a bronze solid (161 mg, 55% yield).

^{1}H NMR (400 MHz, Acetone-d$_6$) δ 8.39 (d, *J* = 9.1 Hz, 2H), 8.20 (s, 1H), 8.03 (d, *J* = 9.1 Hz, 2H), 7.09 (d, *J* = 9.0 Hz, 2H), 6.84 (d, *J* = 8.5 Hz, 2H), 3.74 (s, 3H).

^{13}C NMR (101 MHz, Acetone-d$_6$) δ 157.6, 153.2, 150.4, 134.2, 128.3, 124.7, 123.6, 115.2, 55.7.

HRMS (ESI) Calculated for $C_{13}H_{11}N_2O_4S$: 291.0445. Found: 291.0435 $[M-H]^-$

MP 162-163 °C

N-(3-methoxyphenyl)-4-nitrobenzenesulfinamide (3j)

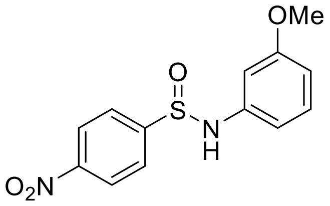

Synthesised according to general procedure A (column conditions: 0-40% EtOAc in hexane). The pure product was afforded as a brown solid (37 mg, 13% yield).

^{1}H NMR (400 MHz, Acetone-d$_6$) δ 8.52 (s, 1H), 8.43 (d, *J* = 8.9 Hz, 2H), 8.08 (d, *J* = 8.9 Hz, 2H), 7.23 – 7.14 (m, 1H), 6.80 – 6.73 (m, 2H), 6.64 – 6.57 (m, 1H), 3.75 (s, 3H).

^{13}C NMR (101 MHz, Acetone-d$_6$) δ 161.6, 53.1, 143.5, 131.0, 128.2, 126.2, 124.8, 111.8, 109.4, 105.5, 55.5.

HRMS (ESI) Calculated for $C_{13}H_{11}N_2O_4S$: 291.0445. Found: 291.0435 $[M-H]^-$

MP 100-102 °C

N-(3,5-dimethoxyphenyl)-4-nitrobenzenesulfinamide (3k)

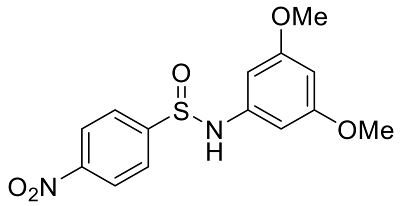

Synthesised according to general procedure A (column conditions: 0-50% EtOAc in hexane). The pure product was afforded as an orange solid (170 mg, 53% yield).

^{1}H NMR (400 MHz, DMSO-d$_6$) δ 9.56 (s, 1H), 8.42 (d, *J* = 8.9 Hz, 2H), 7.98 (d, *J* = 8.7 Hz, 2H), 6.23 (d, *J* = 2.2 Hz, 2H), 6.13 (t, *J* = 2.2 Hz, 1H), 3.67 (s, 6H).

^{13}C NMR (101 MHz, DMSO-d$_6$) δ 161.0, 151.4, 149.1, 143.1, 127.3, 124.2, 96.2, 94.4, 55.1.

HRMS (APCI) Calculated for $C_{14}H_{15}N_2O_5S$: 323.0696. Found: 323.0688 [M+H]$^+$

MP 130-132 ˚C

4-nitro-N-(4-(trifluoromethyl)phenyl)benzenesulfinamide (3l)

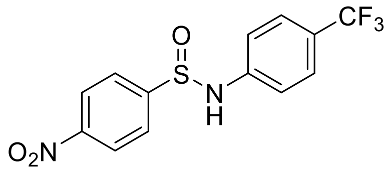

Synthesised according to general procedure A (column conditions: 5-40% EtOAc in hexane). The pure product was afforded as a yellow solid (204 mg, 62% yield).

^{1}H NMR (400 MHz, Acetone-d$_6$) δ 9.03 (s, 1H), 8.44 (d, *J* = 9.1 Hz, 2H), 8.11 (d, *J* = 8.7 Hz, 2H), 7.64 (d, *J* = 8.3 Hz, 2H), 7.37 (d, *J* = 8.4 Hz, 2H).

^{13}C NMR (101 MHz, Acetone-d$_6$) δ 152.3 150.8, 146.0, 128.2, 127.5 (q, *J* = 3.9 Hz), 125.0, 124.8 – 124.0 (m), 118.6.

^{19}F NMR (376 MHz, Acetone-d$_6$) δ -62.36 (s).

HRMS (APCI) Calculated for $C_{13}H_9F_3N_2O_3S$: 330.0280. Found: 330.0280 {M]$^+$

MP 148-152 ˚C

N-(naphthalen-1-yl)-4-nitrobenzenesulfinamide (3m)

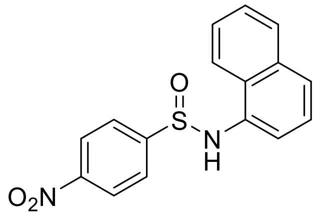

Synthesised according to general procedure A (column conditions: 0-40% EtOAc in hexane). The pure product was afforded as a brown solid (113 mg, 18% yield).

^{1}H NMR (500 MHz, DMSO-d_6) δ 9.79 (s, 1H), 8.36 (d, *J* = 8.9 Hz, 2H), 8.22 – 8.16 (m, 1H), 8.04 (d, *J* = 8.8 Hz, 2H), 7.92 – 7.87 (m, 1H), 7.65 (d, *J* = 8.1 Hz, 1H), 7.59 – 7.50 (m, 2H), 7.46 (d, *J* = 7.5 Hz, 1H), 7.38 (t, *J* = 7.8 Hz, 1H).

^{13}C NMR (126 MHz, DMSO-d_6) δ 151.5, 149.0, 136.6, 133.8, 128.1, 127.5, 127.3, 126.4, 126.0, 125.7, 124.4, 124.0, 122.6, 117.8.

HRMS (ESI) Calculated for $C_{16}H_{12}N_2O_3SNa$: 335.0466. Found: 335.0461 $[M+Na]^+$

MP 120-122 ̊C

4-nitro-N-(pyridin-2-yl)benzenesulfinamide (3n)

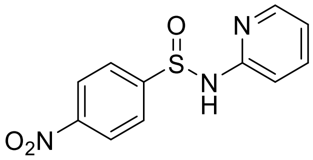

Synthesised according to general procedure A (column conditions: 0-40% EtOAc in hexane). The pure product was afforded as a white solid (144 mg, 55% yield).

^{1}H NMR (400 MHz, DMSO-d_6) δ 10.23 (s, 1H), 8.43 (d, *J* = 8.6 Hz, 2H), 8.29 – 8.22 (m, 1H), 8.04 (d, *J* = 9.1 Hz, 2H), 7.73 – 7.64 (m, 1H), 7.03 – 6.96 (m, 1H), 6.77 (d, *J* = 8.2 Hz, 1H).

^{13}C NMR (101 MHz, DMSO-d_6) δ 154.3, 152.0, 149.1, 147.7, 138.6, 127.1, 124.1, 117.7, 110.7.

HRMS (ESI) Calculated for $C_{11}H_9N_3O_3SNa$: 286.0257. Found: 286.0251 $[M+Na]^+$

MP 132-134 ̊C

4-nitro-N-(quinolin-8-yl)benzenesulfinamide (3o)

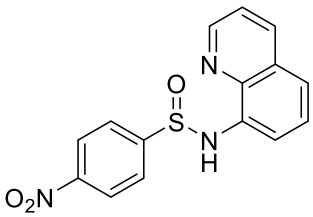

Synthesised according to general procedure A (column conditions: 0-60% EtOAc in hexane). The pure product was afforded as a brown solid (113 mg, 36% yield).

^{1}H NMR (400 MHz, DMSO-d_6) δ 9.99 (s, 1H), 8.90 (dd, *J* = 4.2, 1.7 Hz, 1H), 8.43 – 8.31 (m, 3H), 8.00 (d, *J* = 8.9 Hz, 2H), 7.64 – 7.56 (m, 1H), 7.56 – 7.49 (m, 1H), 7.44 – 7.34 (m, 2H).

^{13}C NMR (101 MHz, DMSO-d_6) δ 151.6, 149.0, 139.0, 136.8, 136.4, 128.3, 127.6, 127.5, 126.6, 124.2, 122.2, 121.3, 114.7.

HRMS (ESI) Calculated for $C_{15}H_{11}N_3O_3SNa$: 336.0413. Found: 336.0410 $[M+Na]^+$

MP 154-156 °C

N-mesityl-4-nitrobenzenesulfinamide (3p)

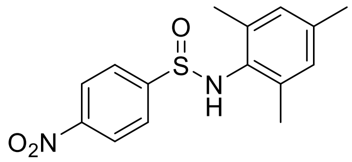

Synthesised according to general procedure A (column conditions: 0-30% EtOAc in hexane). The pure product was afforded as an off-white solid (166 mg, 55% yield).

^{1}H NMR (500 MHz, DMSO-d_6) δ 8.45 (d, *J* = 8.8 Hz, 2H), 8.35 (s, 1H), 8.11 (d, *J* = 9.0 Hz, 2H), 6.91 (s, 2H), 2.32 (s, 6H), 2.22 (s, 3H).

^{13}C NMR (126 MHz, DMSO-d_6) δ 152.6, 149.1, 135.5, 135.1, 134.5, 129.1, 127.2, 124.1, 20.4, 19.0.

HRMS (ESI) Calculated for $C_{15}H_{15}N_2O_3S$: 303.0798. Found: 303.0798 $[M-H]^-$

MP 150-152 °C

N-(2,6-diisopropylphenyl)-4-nitrobenzenesulfinamide (3q)

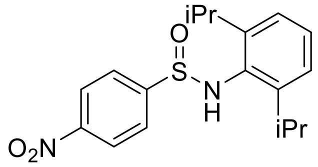

Synthesised according to general procedure A (column conditions: 0-30% EtOAc in hexane). The pure product was afforded as a yellow solid (167 mg, 24% yield).

^{1}H NMR (400 MHz, DMSO-d_6) δ 8.56 (s, 1H), 8.48 (d, *J* = 9.3 Hz, 2H), 8.08 (d, *J* = 9.2 Hz, 2H), 7.30 – 7.17 (m, 3H), 3.52 (p, *J* = 6.8 Hz, 2H), 1.17 (dd, *J* = 9.9, 6.8 Hz, 12H).

^{13}C NMR (101 MHz, DMSO-d_6) δ 152.6, 149.1, 146.7, 133.6, 127.5, 127.0, 124.2, 123.8, 27.6, 24.1, 24.0.

HRMS (ESI) Calculated for $C_{18}H_{21}N_2O_3S$: 345.1273. Found: 345.1278 $[M-H]^-$

MP 90-92 °C

N-(2-(tert-butyl)phenyl)-4-nitrobenzenesulfinamide (3r)

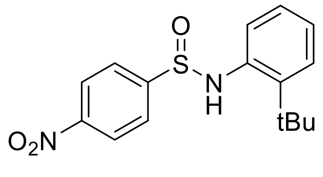

Synthesised according to general procedure A (column conditions: 0-40% EtOAc in hexane). The pure product was afforded as a beige solid (179 mg, 56% yield).

^{1}H NMR (400 MHz, DMSO-d$_6$) δ 8.51 (s, 1H), 8.42 (d, *J* = 9.0 Hz, 2H), 8.03 (d, *J* = 8.6 Hz, 2H), 7.50 (dd, *J* = 7.5, 1.9 Hz, 1H), 7.39 – 7.33 (m, 1H), 7.21 – 7.08 (m, 2H), 1.38 (s, 9H).

^{13}C NMR (101 MHz, DMSO-d$_6$) δ 151.8, 149.0, 143.9, 139.1, 127.31, 127.3, 126.9, 126.6, 125.8, 124.1, 34.9, 30.8.

HRMS (ESI) Calculated for $C_{16}H_{18}N_2O_3SNa$: 341.0930. Found: 341.0917 $[M+Na]^+$

MP 96-98 °C

2-methoxy-4-nitro-N-phenylbenzenesulfinamide (3s)

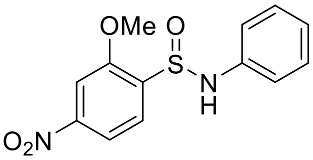

Synthesised by adaptation of a literature procedure.[11] A 100 mL round-bottomed flask was charged with 2-methoxy-4-nitrobenzenesulfonyl chloride (1 g, 4 mmol), evacuated under vacuum and filled with nitrogen. Anhydrous DCM (10 mL) was added, alongside NEt_3 (8 mmol, 1.12 mL) and the solution was stirred at 0°C . A separate 100 mL round-bottomed flask was charged with PPh_3 (1.052 g, 4 mmol), evacuated under vacuum and filled with nitrogen. Anhydrous DCM (10 mL) and aniline (365 µL, 4 mmol) was added and the solution was stirred at 0°C. The contents of the second flask were added dropwise to the first flask over ice, and the reaction mixture was left stirring for 16 hours. The reaction mixture was extracted with DCM (20 mL), washed with H_2O (3 x 20 mL), dried with anhydrous $MgSO_4$. The organic layers were then filtered, concentrated *in vacuo* and purified by flash column chromatography (0-40% EtOAc in hexane). The pure product was afforded as a yellow solid (247 mg, 21% yield).

^{1}H NMR (500 MHz, DMSO-d$_6$) δ 9.28 (s, 1H), 8.07 – 7.97 (m, 2H), 7.89 (d, *J* = 1.9 Hz, 1H), 7.24 (t, 2H), 7.02 (d, *J* = 7.7 Hz, 2H), 6.95 (t, *J* = 7.4 Hz, 1H), 3.94 (s, 3H).

^{13}C NMR (126 MHz, DMSO-d$_6$) δ 156.3, 150.6, 142.0, 138.6, 129.2, 127.1, 122.3, 117.7, 115.5, 106.9, 57.0.

HRMS (ESI) Calculated for $C_{13}H_{12}N_2O_4SNa$: 315.0410. Found: 315.0405 $[M+Na]^+$

MP 104-106 °C

5-nitro-N-phenylpyridine-2-sulfinamide (3t)

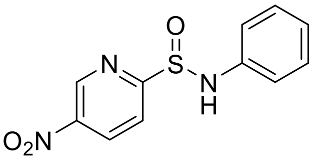

Synthesised by adaptation of a literature procedure.[10] A 50 mL round-bottomed flask was charged with KF (1 mmol, 59 mg). CH_3CN (4.2 mL) and H_2O (0.8 mL) were added and the flask was stirred at 0 °C. *Meta*-chloroperoxybenzoic acid, 70% (*m*-CBPA) (1 mmol, 172 mg) was added slowly and the mixture was left to stir for 30 min. Sulfenamide ***3tp*** (0.5 mmol, 132 mg) was added portion-wise and

the reaction mixture was stirred at 0 °C for 30 min. The reaction mixture was then extracted with EtOAc (20 mL), washed with saturated aqueous $NaHCO_3$ (3 x 20 mL) and dried with anhydrous $MgSO_4$. The organic layers were then filtered, concentrated *in vacuo* and purified by flash column chromatography (0-50% EtOAc in hexane). The pure product was afforded as a white solid (58 mg, 45% yield).

^{1}H NMR (500 MHz, DMSO-d$_6$) δ 9.71 (s, 1H), 9.48 – 9.40 (m, 1H), 8.84 (dd, *J* = 8.7, 2.6 Hz, 1H), 8.26 (dd, *J* = 8.5, 0.7 Hz, 1H), 7.22 (t, *J* = 7.9 Hz, 2H), 7.04 (d, *J* = 7.4 Hz, 2H), 6.93 (t, *J* = 7.4 Hz, 1H).

^{13}C NMR (126 MHz, DMSO-d$_6$) δ 168.8, 145.1, 144.9, 141.2, 133.5, 129.3, 122.5, 121.9, 117.7.

HRMS (APCI) Calculated for $C_{11}H_{10}N_3O_3S$: 264.0437. Found: 264.0430 $[M+H]^+$

MP 155-158 °C

Compounds Synthesised for Mechanistic investigations

4-nitro-N-phenylbenzenesulfonamide (1a)

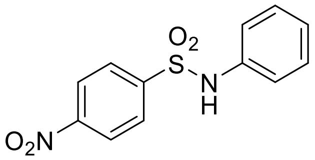

Prepared according to a literature procedure.[12] A 50 mL round-bottomed flask was charged with 4-nitrobenzenesulfonyl chloride (244 mg, 1.1 mmol), which was evacuated under vacuum and filled with nitrogen. Pyridine (5 mL) was added, alongside aniline (91 µL, 1 mmol), and the reaction mixture was stirred at room temperature for 3 hours. Toluene (10 mL) was added to the reaction mixture and the solvent was evaporated *in vacuo*. The reaction mixture was then dissolved in DCM (20 mL), extracted from H_2O (2 x 20 mL), dried with anhydrous $MgSO_4$, filtered and concentrated *in vacuo*. The crude product was then purified by flash column chromatography (10-30% EtOAc in hexane) affording the pure product as a white solid (126 mg, 45%).

^{1}H NMR (500 MHz, Acetone-d$_6$) δ 9.29 (s, 1H), 8.38 (d, *J* = 8.6 Hz, 2H), 8.05 (d, *J* = 9.0 Hz, 2H), 7.29 (t, *J* = 7.2 Hz, 2H), 7.23 (d, *J* = 7.6 Hz, 2H), 7.14 (t, *J* = 6.9 Hz, 1H).

^{13}C NMR (126 MHz, Acetone-d$_6$) δ 151.2, 146.2, 137.9, 130.2, 129.5, 126.2, 125.2, 122.4.

HRMS (APCI) Calculated for $C_{12}H_9N_2O_4S$: 277.0289. Found: 277.0280 $[M-H]^-$

Data is in accordance with literature.[12]

N-methyl-4-nitro-N-phenylbenzenesulfinamide (3u)

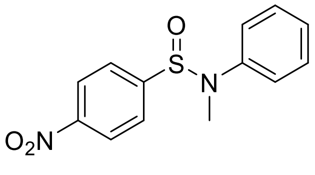

Synthesised according to general procedure A (column conditions: 5-40% EtOAc in hexane). The pure product was afforded as a brown solid (138 mg, 50% yield).

^{1}H NMR (500 MHz, Acetone-d$_6$) δ 8.46 (d, *J* = 9.0 Hz, 2H), 8.03 (d, *J* = 8.2 Hz, 2H), 7.45 – 7.36 (m, 4H), 7.23 – 7.16 (m, 1H), 2.90 (s, 3H).

^{13}C NMR (126 MHz, Acetone-d$_6$) δ 151.6, 146.6, 130.3, 128.3, 125.4, 125.1, 122.1, 31.6.

HRMS (APCI) Calculated for $C_{13}H_{13}N_2O_3S$: 277.0641. Found: 277.0640 $[M+H]^+$

MP 90-95 °C

2-((4-nitrophenyl)thio)aniline (11)

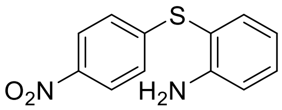

A 100 mL round-bottomed flask was charged with 4-fluoronitrobenzene (282 mg, 2 mmol) and K_2CO_3 (553 mg, 4 mmol), which was evacuated under vacuum and filled with nitrogen. Anhydrous DMF (10 mL) was added to the mixture which was stirred in a pre-warmed oil bath at 70 °C. 2-aminobenzenethiol (0.207 mL, 2 mmol) was added dropwise and the mixture was left to stir at 70 °C for 1 h. The reaction mixture was dissolved in EtOAc (20 mL) and washed with 10% (w/v) aqueous LiCl (3 x 20 mL). The aqueous layer was then extracted with EtOAc (3 x 20 mL) and the combined organic layers were dried with anhydrous $MgSO_4$, filtered and concentrated *in vacuo*. The crude product was then purified by flash column chromatography (0-40% EtOAc in hexane), affording the pure product as a brown solid (456 mg, 93% yield).

^{1}H NMR (500 MHz, $CDCl_3$) δ 8.06 (d, *J* = 9.2 Hz, 2H), 7.47 – 7.42 (m, 1H), 7.36 – 7.29 (m, 1H), 7.11 (d, *J* = 9.1 Hz, 2H), 6.86 – 6.83 (m, 1H), 6.83 – 6.78 (m, 1H), 4.29 (s, 2H).

^{13}C NMR (126 MHz, $CDCl_3$) δ 149.2, 147.3, 145.4, 137.8, 132.5, 125.6, 124.2, 119.3, 115.8, 111.4.

HRMS (ESI) Calculated for $C_{12}H_{10}N_2O_2SNa$: 269.0355. Found: 269.0347 $[M+Na]^+$

Data is in accordance with literature.[13]

2-((4-nitrophenyl)sulfinyl)aniline (10)

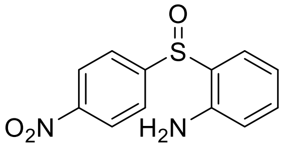

A 100 mL round-bottomed flask was charged with 2-((4-nitrophenyl)thio)aniline **11** (440 mg, 1.78 mmol), evacuated under vacuum and filled with nitrogen. DCM (10 mL) was added and the mixture was stirred at 0 °C. A second 100 mL round-bottomed flask was charged with *Meta*-chloroperoxybenzoic acid, 70% (*m*-CBPA) (230 mg, 1.9 mmol), evacuated under vacuum and filled with nitrogen. DCM (10 mL) was added and the contents of this flask were added slowly to the first flask. The reaction mixture was stirred at 0 °C for 1 h, at which point the ice bath was removed and the mixture was allowed to warm to room temperature. On consumption of starting material (monitored

by TLC) the reaction was quenched with saturated aqueous $NaHCO_3$ (20 mL) and extracted with DCM (2 x 20 mL). The combined organic layers were then dried with anhydrous $MgSO_4$, filtered and concentrated *in vacuo*. The crude product was then purified by flash column chromatography (20-60% EtOAc in hexane), affording the pure product as a yellow solid (163 mg, 35% yield).

^{1}H NMR (500 MHz, $CDCl_3$) δ 8.29 (d, *J* = 9.1 Hz, 2H), 7.72 (d, *J* = 8.8 Hz, 2H), 7.49 (dd, *J* = 7.7, 1.5 Hz, 1H), 7.32 – 7.25 (m, 1H), 6.82 (td, *J* = 7.6, 1.1 Hz, 1H), 6.64 – 6.58 (m, 1H), 4.89 (s, 2H).

^{13}C NMR (126 MHz, $CDCl_3$) δ 150.9, 149.1, 148.0, 134.0, 128.6, 125.9, 124.0, 122.6, 118.0, 117.8.

HRMS (ESI) Calculated for $C_{12}H_{10}N_2O_3SNa$: 285.0304. Found: 285.0296 $[M+Na]^+$

Sulfinamide Starting Material Precursors

Methyl 4-nitrobenzenesulfinate (4)

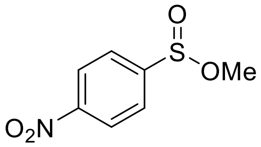

Prepared according to a literature procedure.[14] A 250 ml round-bottomed flask was charged with 1,2-bis(4-nitrophenyl)disulfane (2.48 g, 8 mmol), which was then dissolved in MeOH (50 mL) and DCM (50 mL). The solution was stirred at 0 °C, and then N-bromosuccinimide (7.2 g, 40 mmol) was added slowly. The reaction mixture was stirred overnight. The reaction mixture was then extracted with DCM (50 mL), washed with saturated aqueous $NaHCO_3$ (2 x 50 mL), dried with anhydrous Na_2SO_4, filtered and concentrated *in vacuo*. The crude product was purified by flash column chromatography (0-30% EtOAc in hexane) to afford a light yellow solid (2.752 g, 85% yield).

^{1}H NMR (400 MHz, $CDCl_3$) δ 8.40 (d, *J* = 8.7 Hz), 7.90 (d, *J* = 8.6 Hz, 2H, 3.54 (s, 3H).

^{13}C NMR (101 MHz, $CDCl_3$) δ 150.3, 150.2, 126.9, 124.3, 50.7.

HRMS (ESI) Calculated for $C_7H_7NO_4S$: 201.0096 Found: 201.0101 $[M]^-$

Data is in accordance with literature.[15]

S-(5-nitropyridin-2-yl)-N-phenylthiohydroxylamine (7b)

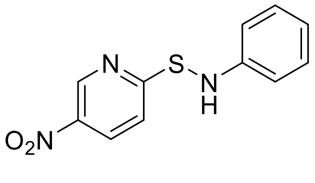

Prepared by adaptation of a literature procedure.[16] a 100 mL round-bottomed flask was charged with $AgNO_3$ (350 mg, 2 mmol) and 5-methyl-2-((5-nitropyridin-2-yl)disulfaneyl)pyridine (612 mg, 2 mmol) and the flask was evacuated under vacuum and filled with nitrogen. Anhydrous DCM (10 mL) and MeOH (20 mL) were added and the flask was stirred at 0 °C. Aniline (0.9 mL, 10 mmol) was added dropwise and the reaction mixture was stirred overnight. The reaction mixture was then filtered and concentrated *in vacuo*. The concentrated product was then extracted with DCM (30 mL), washed with

H_2O (2 x 20 mL), dried with anhydrous $MgSO_4$, filtered and concentrated *in vacuo*. The crude product was purified by flash column chromatography (0-30% EtOAc in hexane) to afford a yellow solid (189 mg, 38% yield).

^{1}H NMR (500 MHz, DMSO-d$_6$) δ 9.23 (d, *J* = 2.4 Hz, 1H), 8.49 (dd, *J* = 8.9, 2.6 Hz, 1H), 8.30 (s, 1H), 7.35 (d, *J* = 8.4 Hz, 1H), 7.23 (t, *J* = 8.3 Hz, 2H), 7.01 (d, *J* = 7.6 Hz, 2H), 6.84 (t, *J* = 7.3 Hz, 1H).

^{13}C NMR (126 MHz, DMSO-d$_6$) δ 173.1, 146.0, 145.1, 141.5, 132.3, 129.4, 120.3, 117.0, 114.4.

HRMS (APCI) Calculated for $C_{11}H_{10}N_3O_2S$: 248.0488. Found: 248.0477 $[M+H]^+$

MP 112-114 °C

4. Poor-yielding and Unsuccessful Substrates

N-Aryl Scope

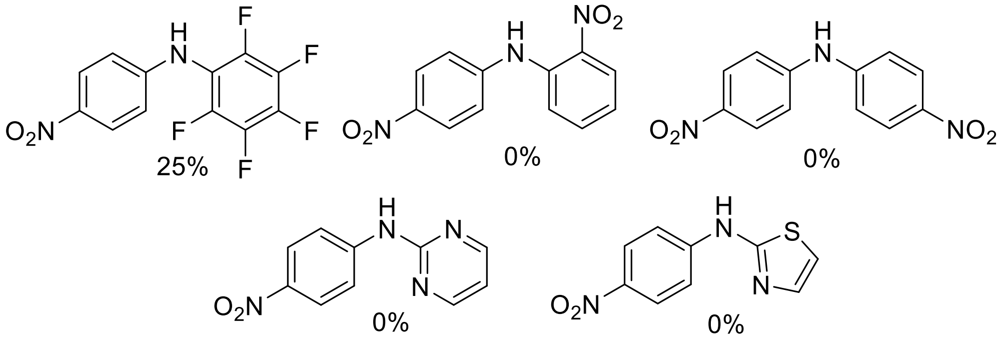

S-Aryl Scope

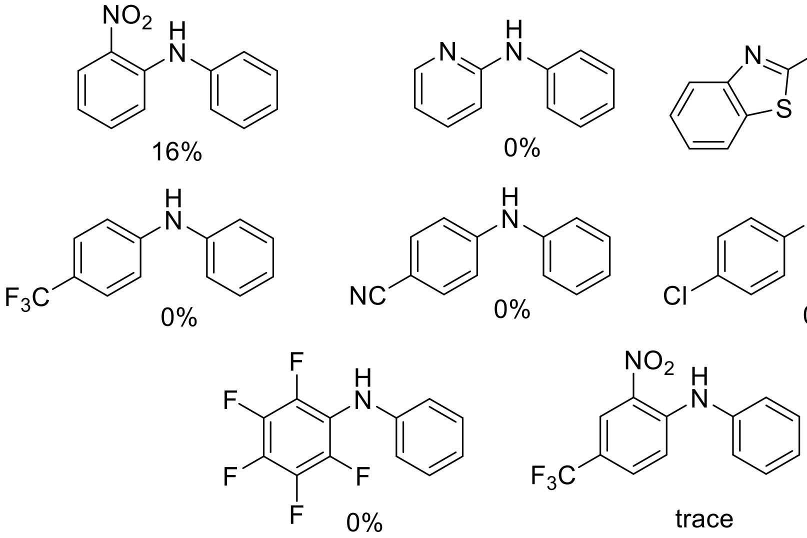

5. NMR Spectra for Synthesised Compounds

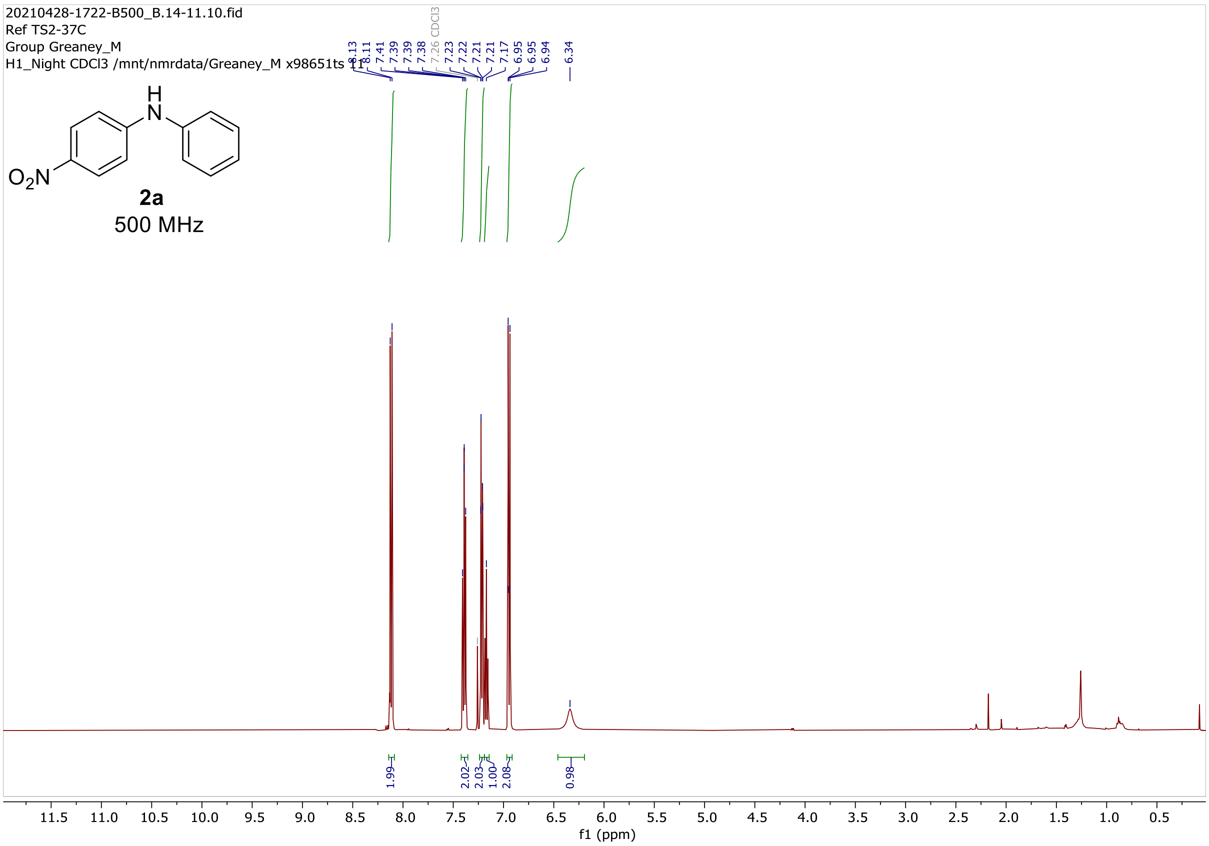

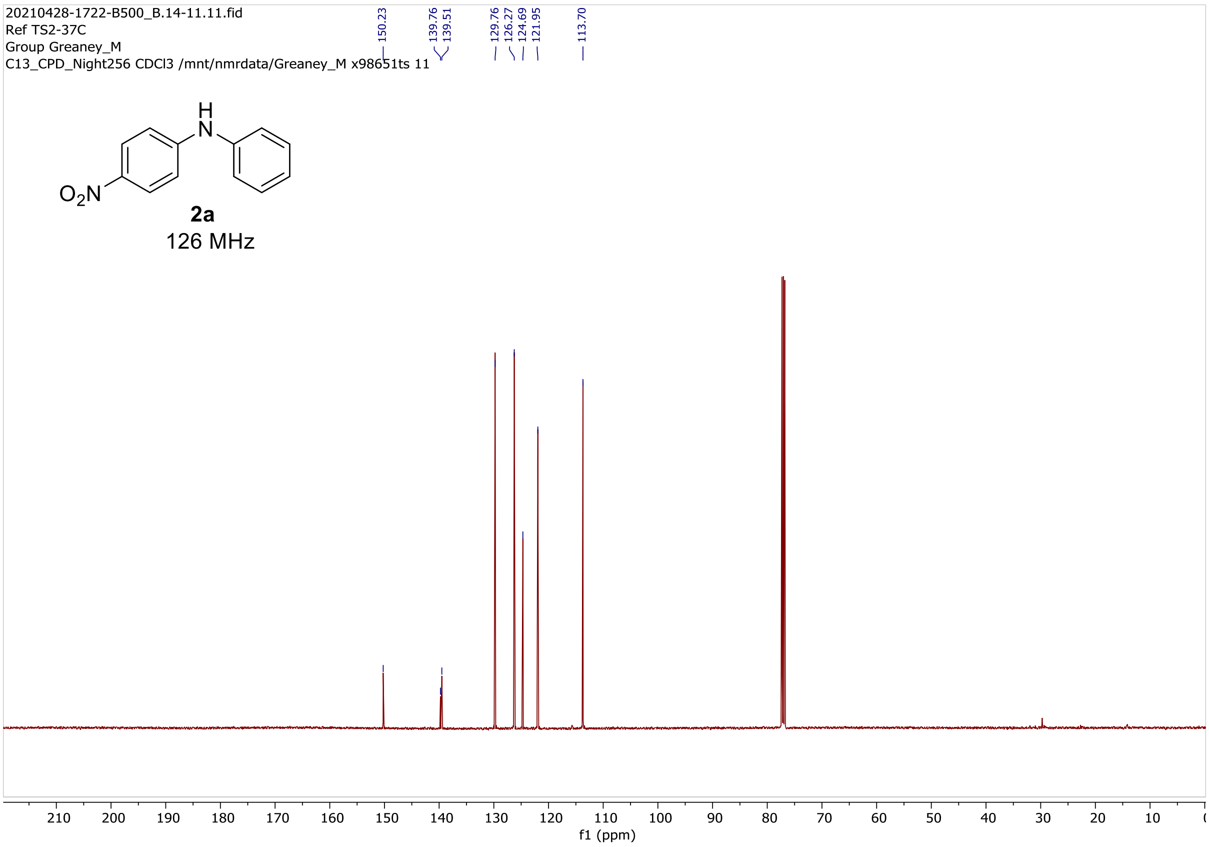

20210604-1124-B400_B.11-31.10.fid
Ref TS2-73C
Group Greaney_M
H1_Night Acetone /mnt/nmrdata/Greaney_M x98651ts 31

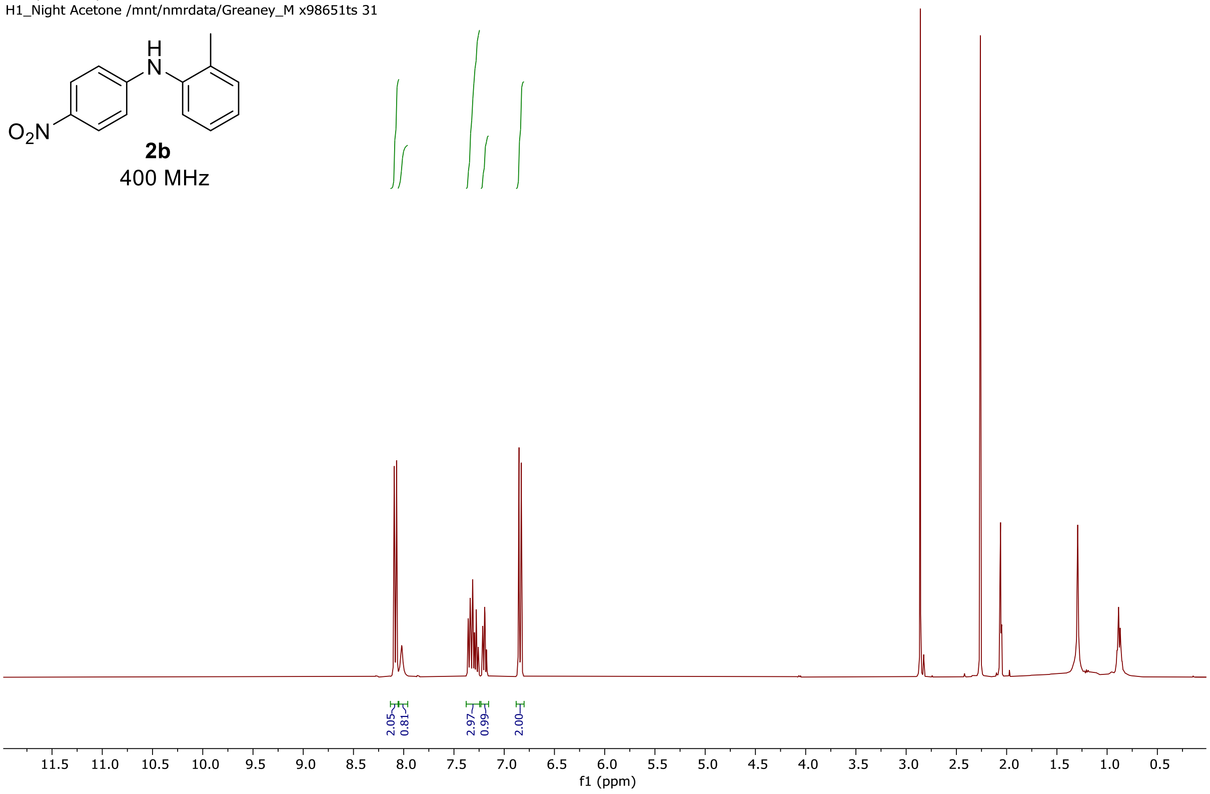

20210604-1124-B400_B.11-31.11.fid
Ref TS2-73C
Group Greaney_M
C13_CPD_Night256 Acetone /mnt/nmrdata/Greaney_M x98651ts 31

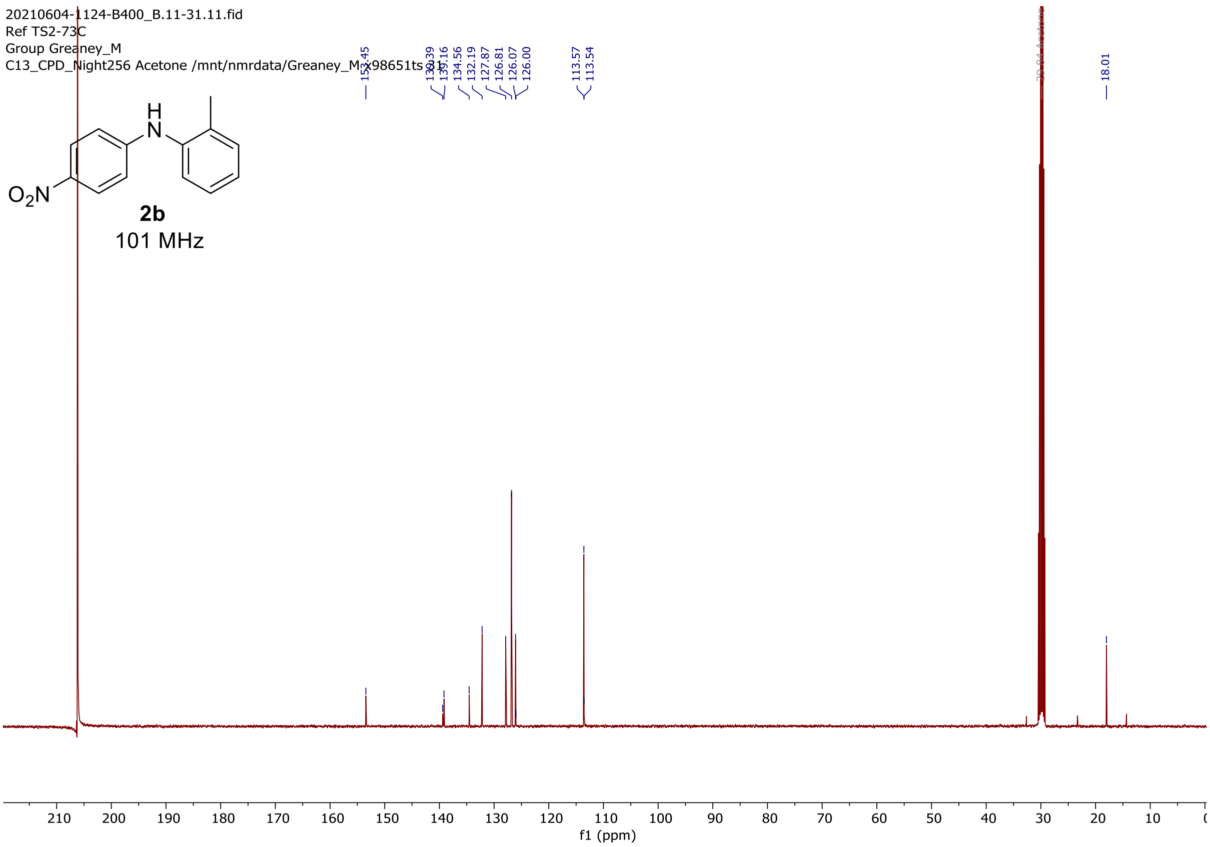

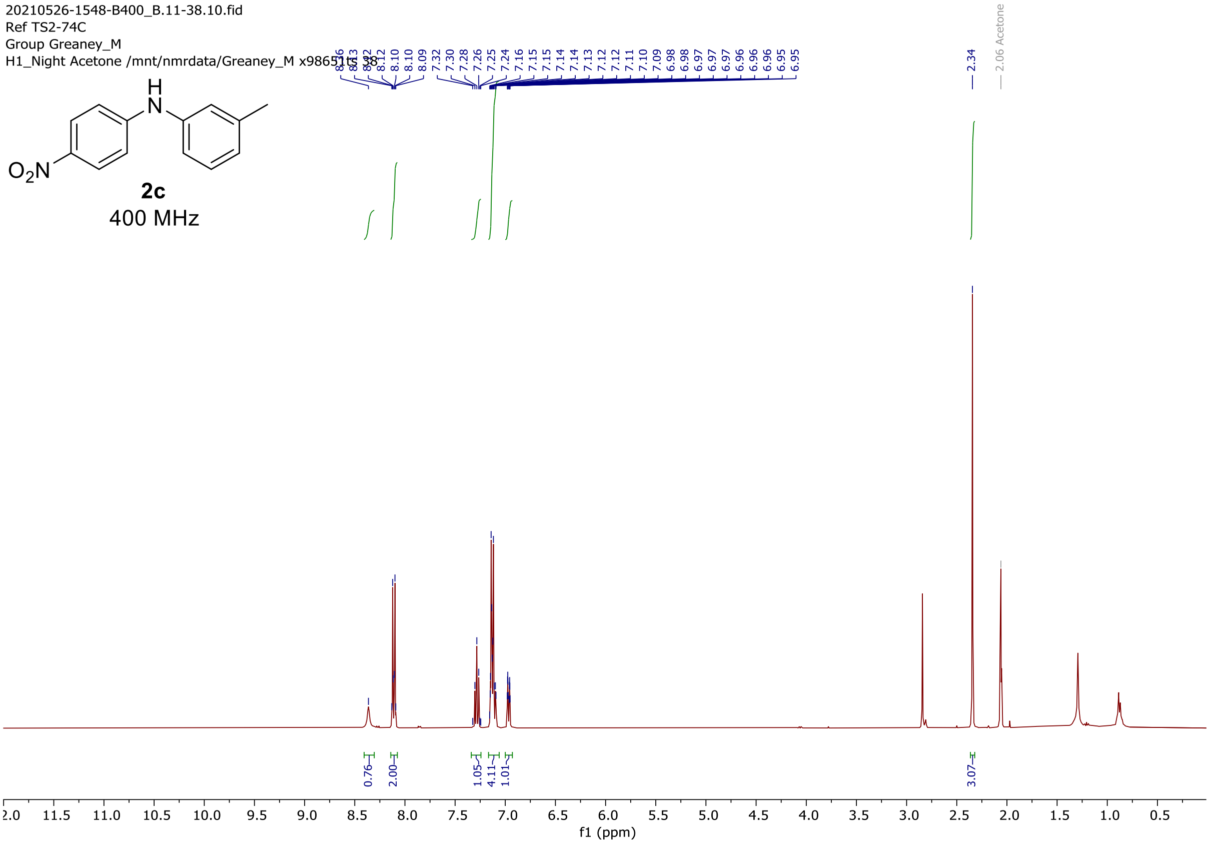
20210526-1548-B400_B.11-38.10.fid
Ref TS2-74C
Group Greaney_M
H1_Night Acetone /mnt/nmrdata/Greaney_M x98651ts-38
2c
400 MHz
2.34
2.06 Acetone
0.76
2.00
1.05
4.11
1.01
3.07
f1 (ppm)

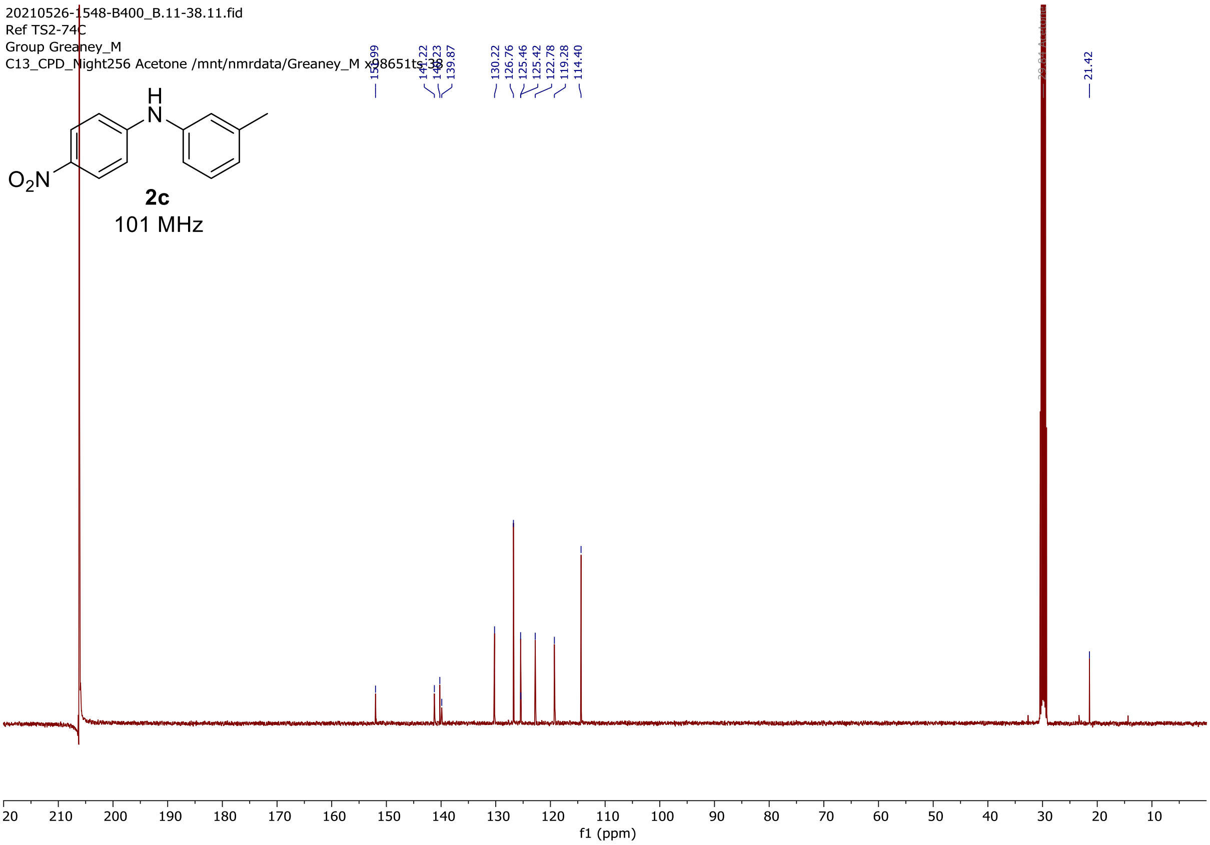
20210526-1548-B400_B.11-38.11.fid
Ref TS2-74C
Group Greaney_M
C13_CPD_Night256 Acetone /mnt/nmrdata/Greaney_M x98651ts-38
2c
101 MHz
151.99
141.22
140.23
139.87
130.22
126.76
125.46
125.42
122.78
119.28
114.40
21.42
f1 (ppm)

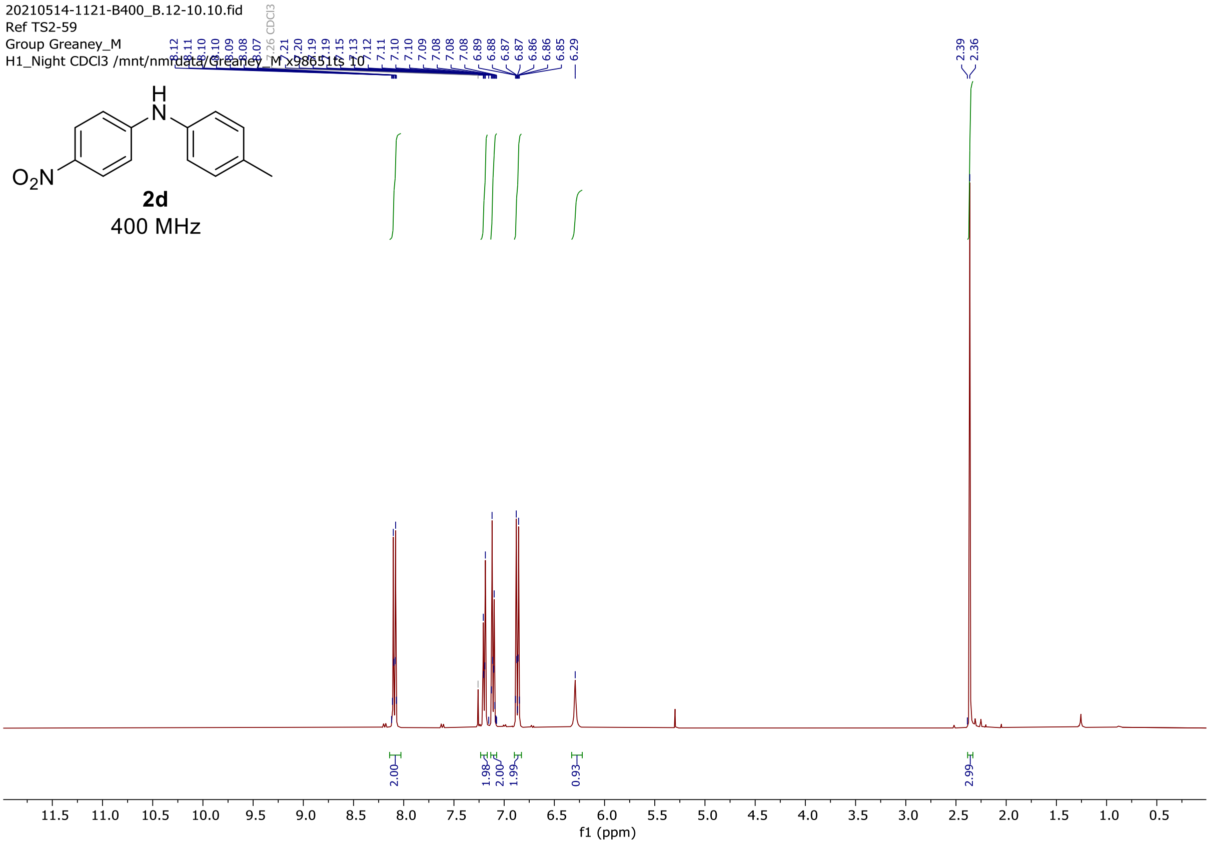
20210514-1121-B400_B.12-10.10.fid
Ref TS2-59
Group Greaney_M
H1_Night CDCl3 /mnt/nmrdata/Greaney_M x98651ts 10
7.26 CDCl3
7.21
7.20
7.19
7.19
7.15
7.13
7.12
7.11
7.10
7.10
7.09
7.08
7.08
7.08
6.89
6.88
6.87
6.87
6.86
6.86
6.85
6.29
2.39
2.36
2d
400 MHz
2.00
1.98
2.00
1.99
0.93
2.99
f1 (ppm)

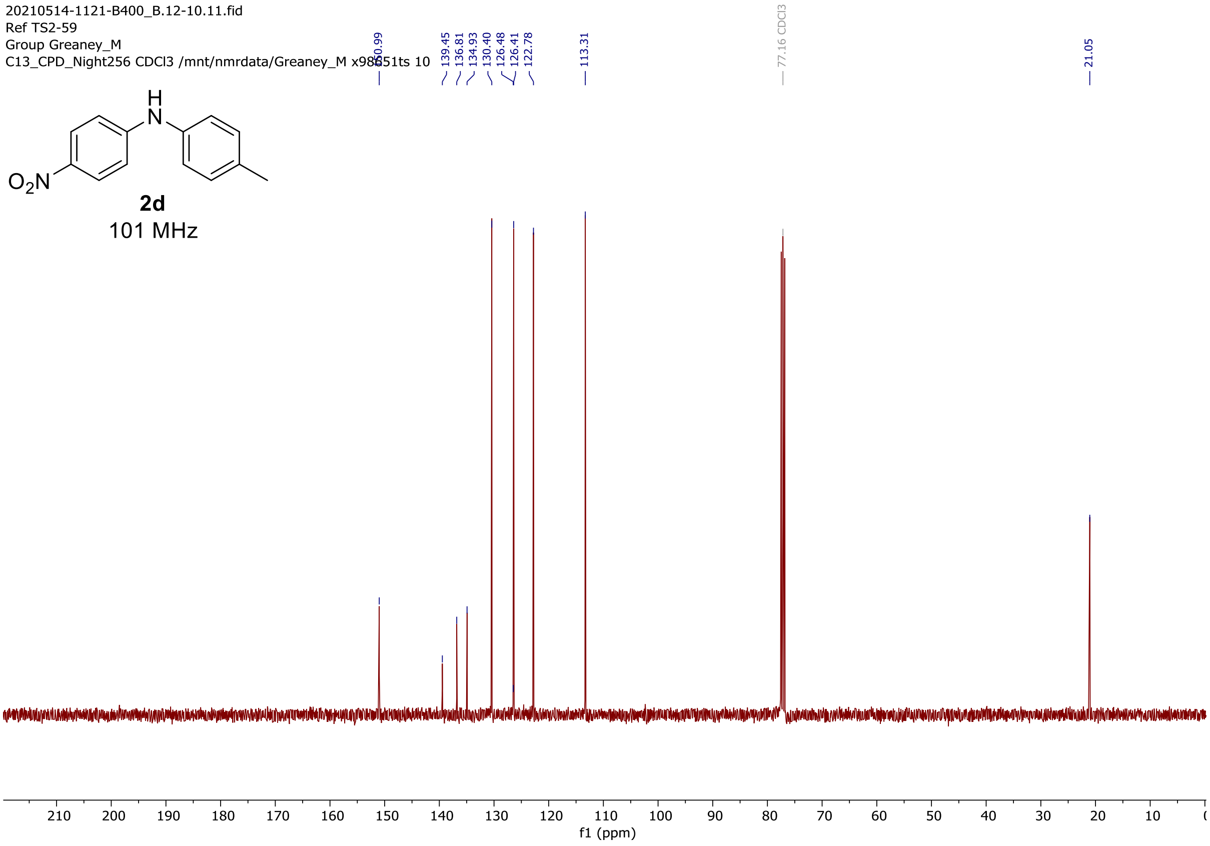
20210514-1121-B400_B.12-10.11.fid
Ref TS2-59
Group Greaney_M
C13_CPD_Night256 CDCl3 /mnt/nmrdata/Greaney_M x98651ts 10
139.45
136.81
134.93
130.40
126.48
126.41
122.78
113.31
77.16 CDCl3
21.05
2d
101 MHz
f1 (ppm)

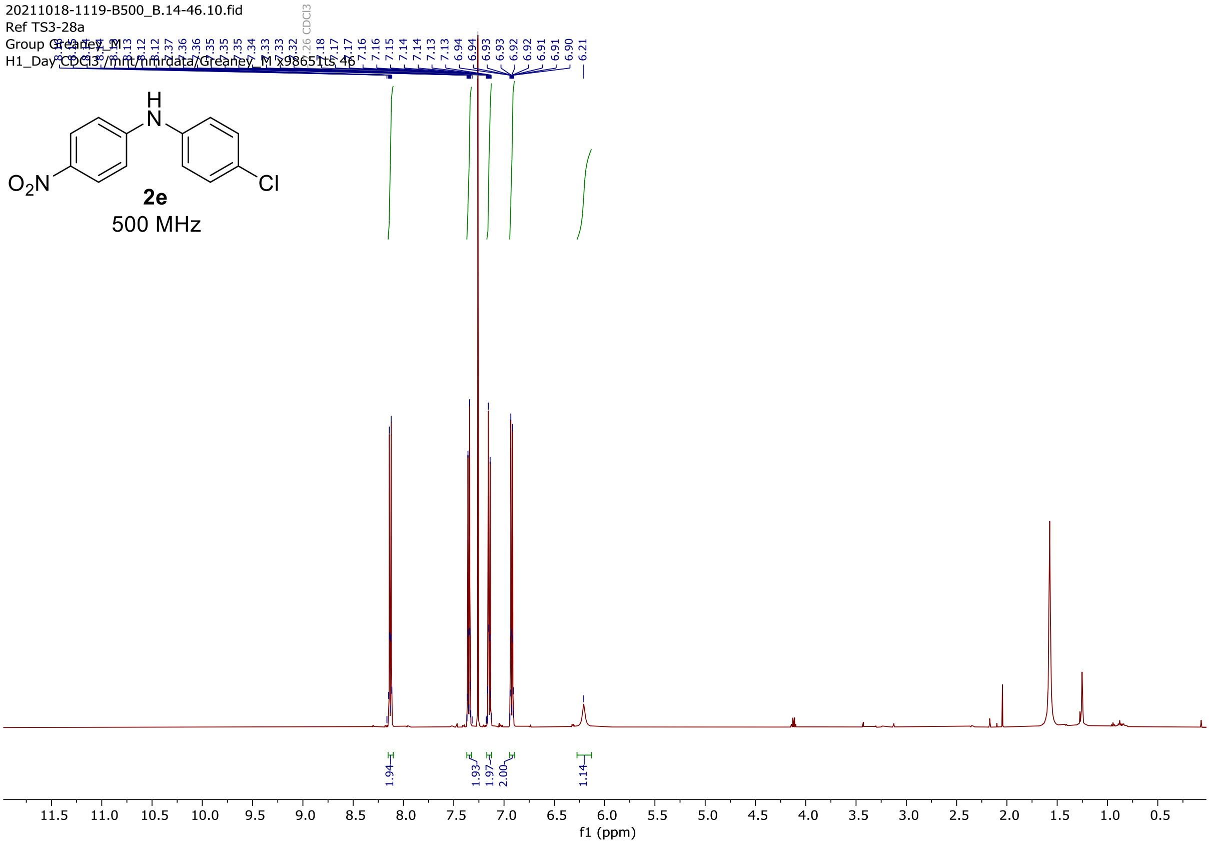
20211018-1119-B500_B.14-46.10.fid
Ref TS3-28a
Group Greaney_M
H1_Day CDCl3 /mnt/nmrdata/Greaney_M x98651ts 46
2e
500 MHz
1.94
1.93
1.97
2.00
1.14
f1 (ppm)

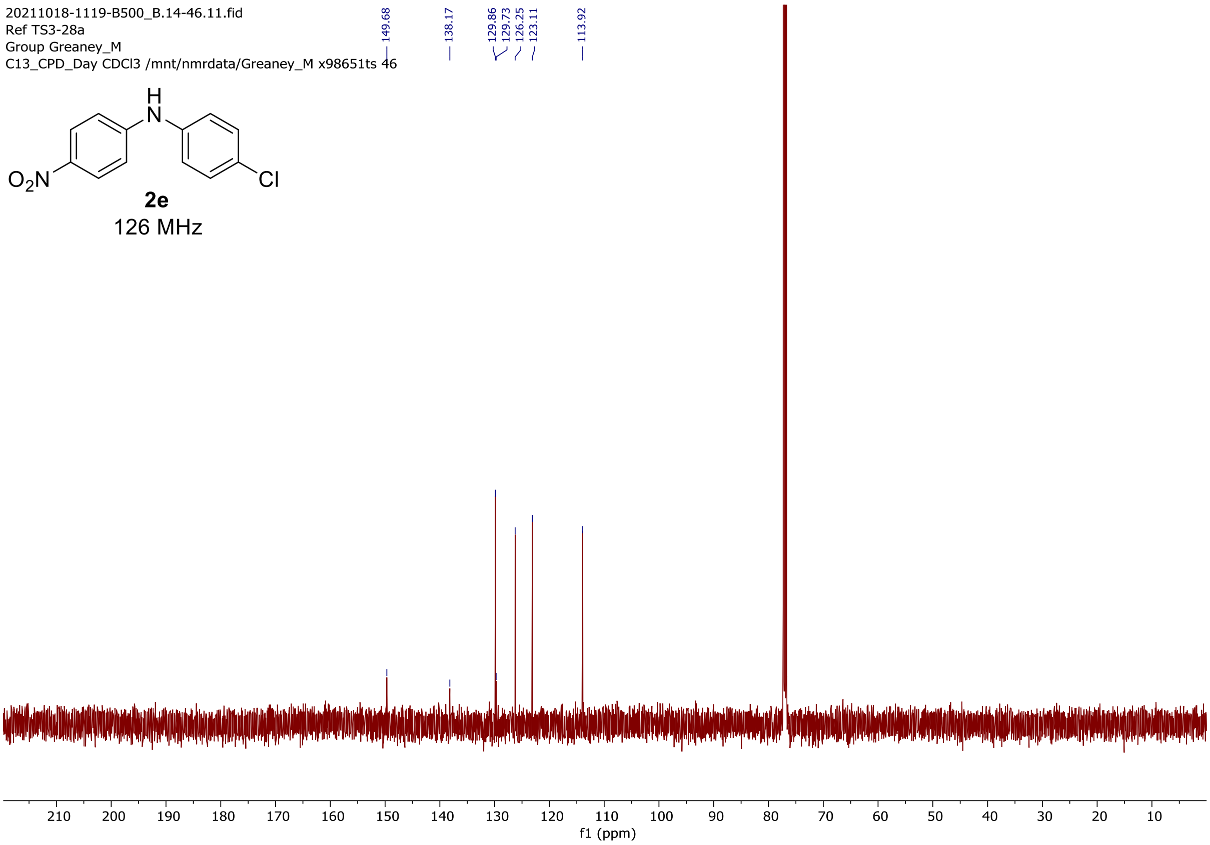
20211018-1119-B500_B.14-46.11.fid
Ref TS3-28a
Group Greaney_M
C13_CPD_Day CDCl3 /mnt/nmrdata/Greaney_M x98651ts 46
149.68
138.17
129.86
129.73
126.25
123.11
113.92
2e
126 MHz
f1 (ppm)

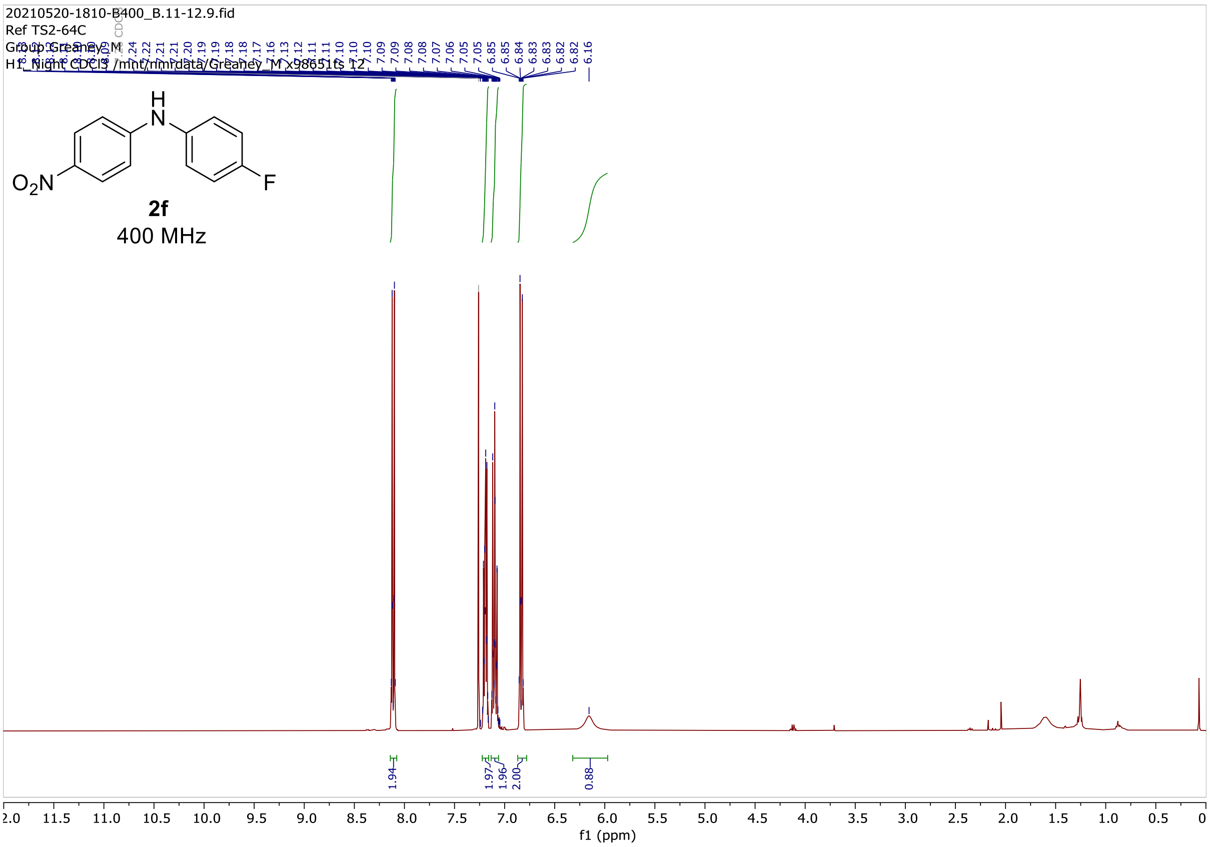

20210520-1810-B400_B.11-12.9.fid
Ref TS2-64C
Group Greaney_M
H1_Night CDCl3 /mnt/nmrdata/Greaney_M x98651ts 12
2f
400 MHz
1.94
1.97
1.96
2.00
0.88
f1 (ppm)

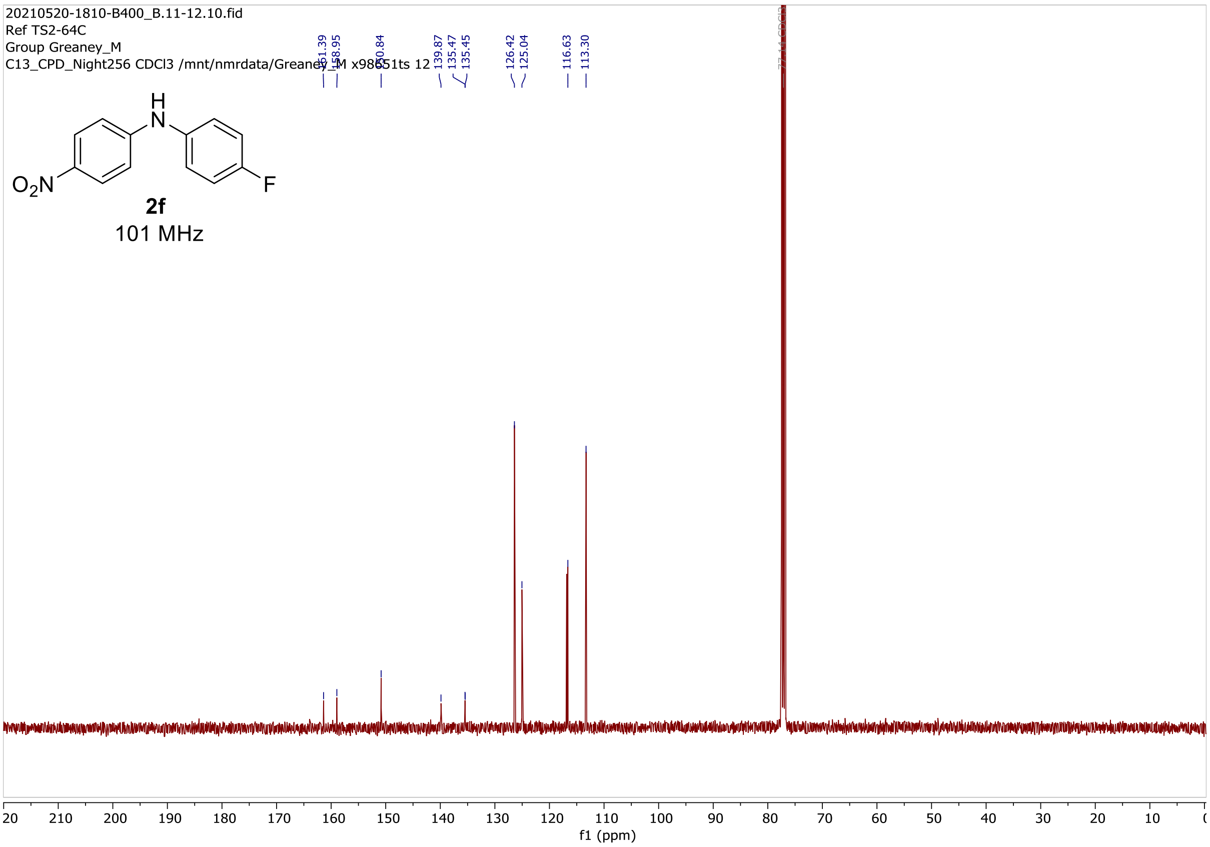

20210520-1810-B400_B.11-12.10.fid
Ref TS2-64C
Group Greaney_M
C13_CPD_Night256 CDCl3 /mnt/nmrdata/Greaney_M x98651ts 12
139.87
135.47
135.45
126.42
125.04
116.63
113.30
2f
101 MHz
f1 (ppm)

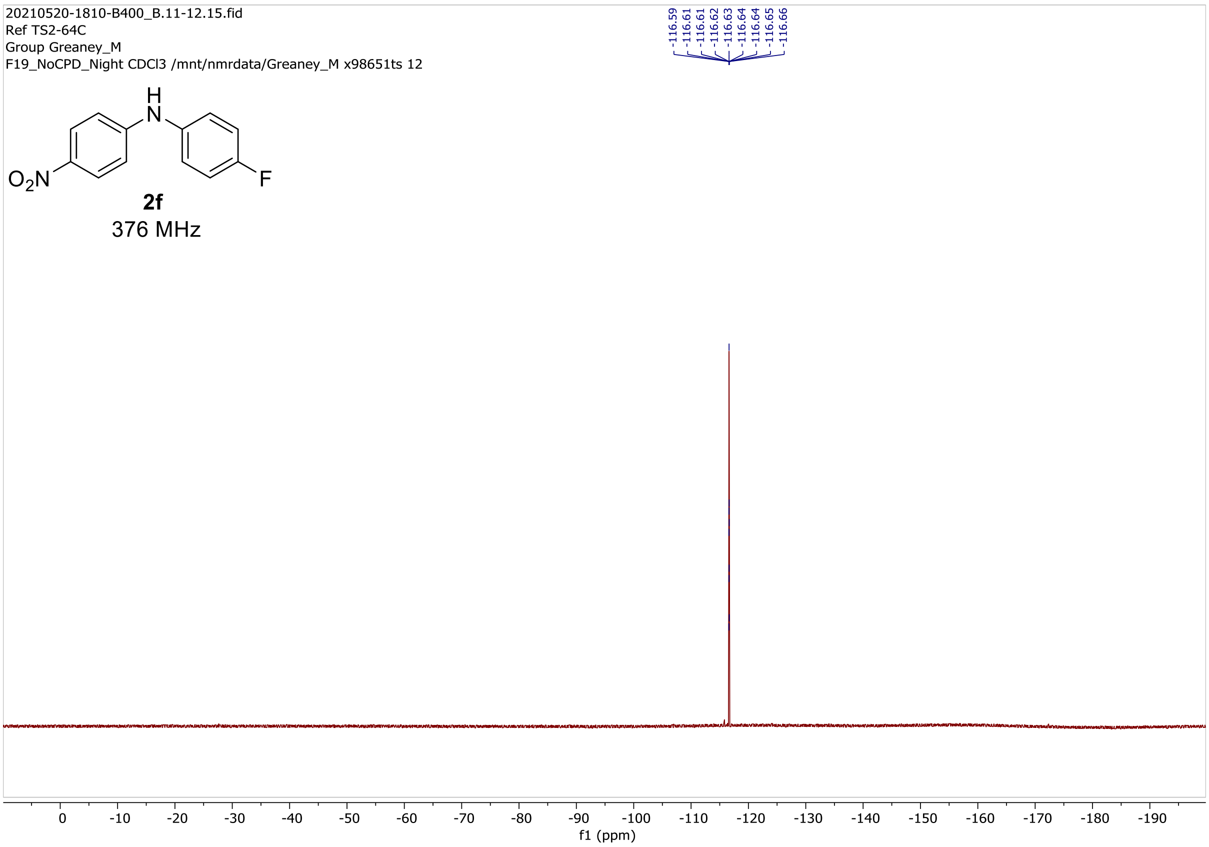
20210520-1810-B400_B.11-12.15.fid
Ref TS2-64C
Group Greaney_M
F19_NoCPD_Night CDCl3 /mnt/nmrdata/Greaney_M x98651ts 12
-116.59 -116.61 -116.61 -116.62 -116.63 -116.64 -116.64 -116.65 -116.66
2f
376 MHz
0 -10 -20 -30 -40 -50 -60 -70 -80 -90 -100 -110 -120 -130 -140 -150 -160 -170 -180 -190
f1 (ppm)

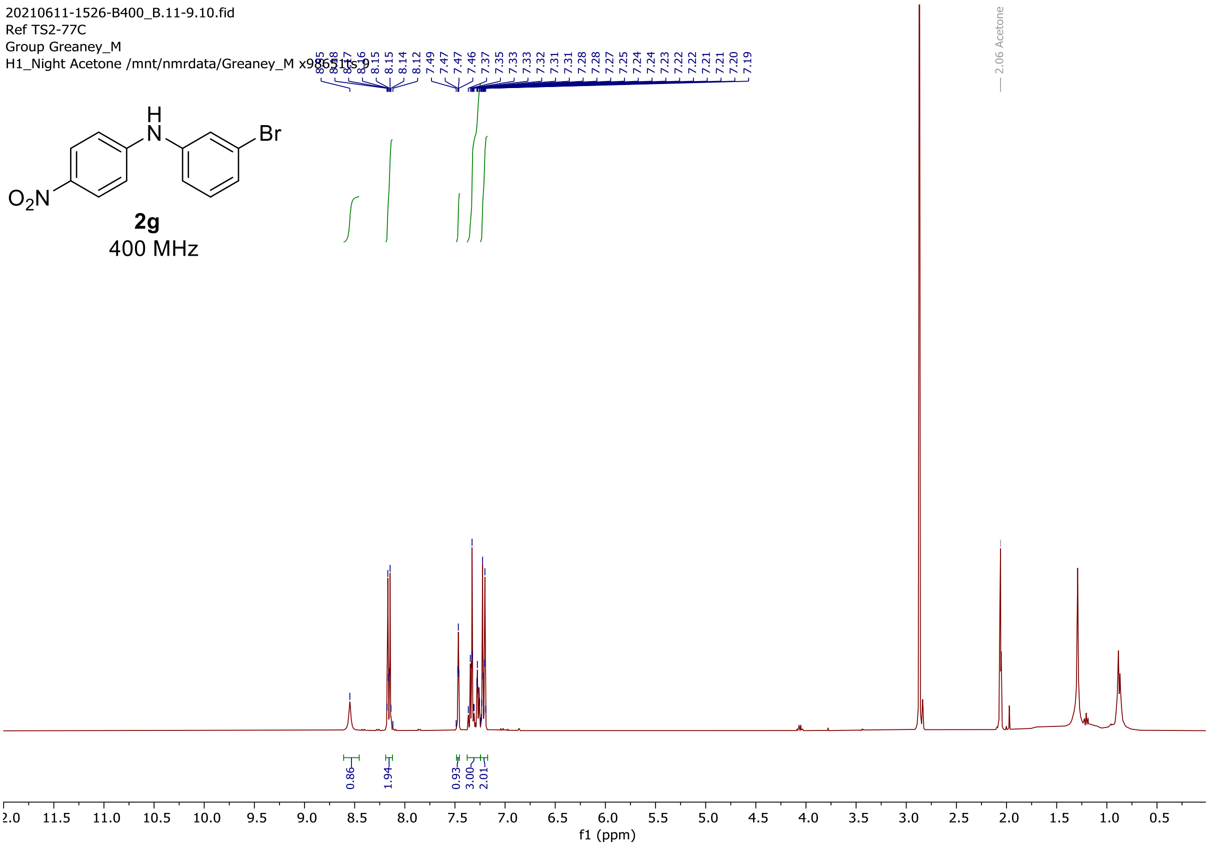
20210611-1526-B400_B.11-9.10.fid
Ref TS2-77C
Group Greaney_M
H1_Night Acetone /mnt/nmrdata/Greaney_M x98651ts 9
8.15 8.15 8.14 8.12 7.49 7.47 7.47 7.46 7.37 7.35 7.33 7.32 7.31 7.31 7.28 7.27 7.25 7.24 7.23 7.22 7.22 7.21 7.20 7.19
2.06 Acetone
2g
400 MHz
0.86 1.94 0.93 3.00 2.01
f1 (ppm)

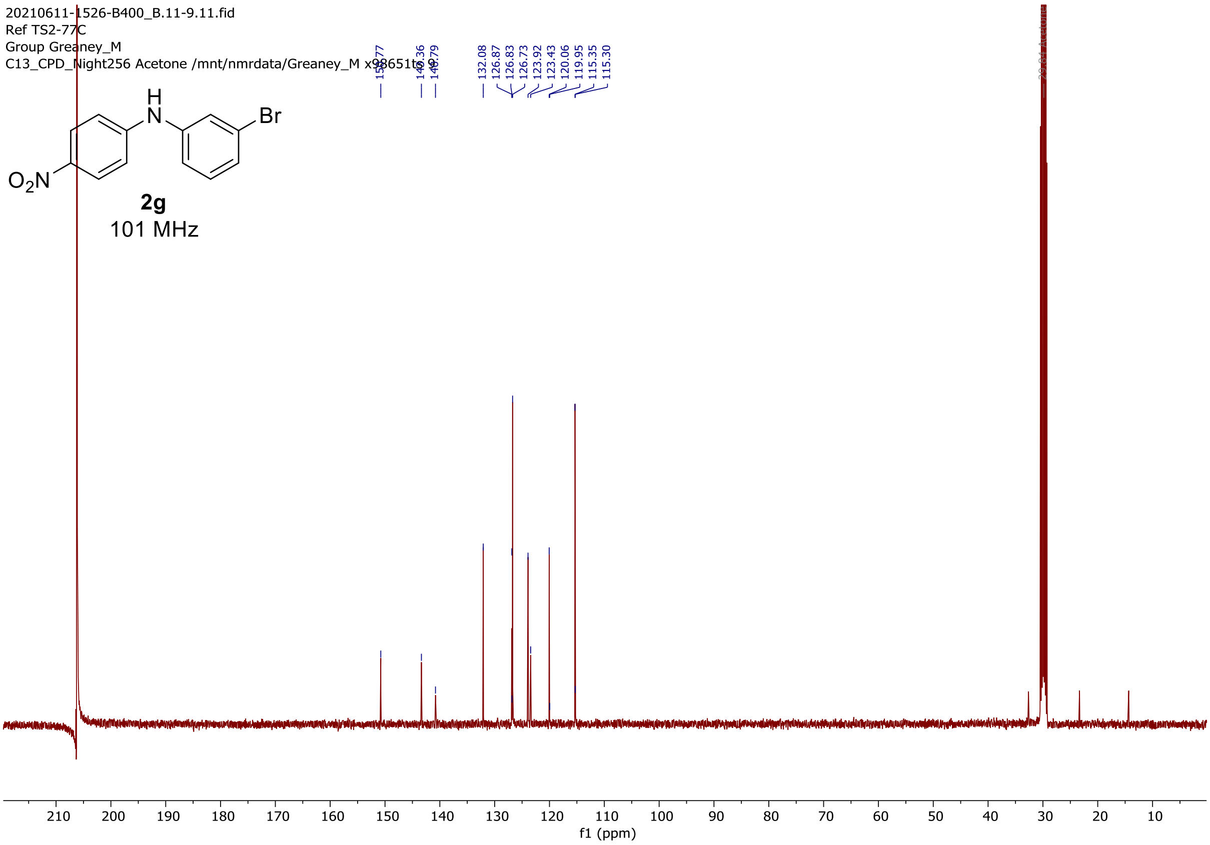
20210611-1526-B400_B.11-9.11.fid
Ref TS2-77C
Group Greaney_M
C13_CPD_Night256 Acetone /mnt/nmrdata/Greaney_M
132.08
126.87
126.83
126.73
123.92
123.43
120.06
119.95
115.35
115.30
H
N
Br
O2N
2g
101 MHz
f1 (ppm)

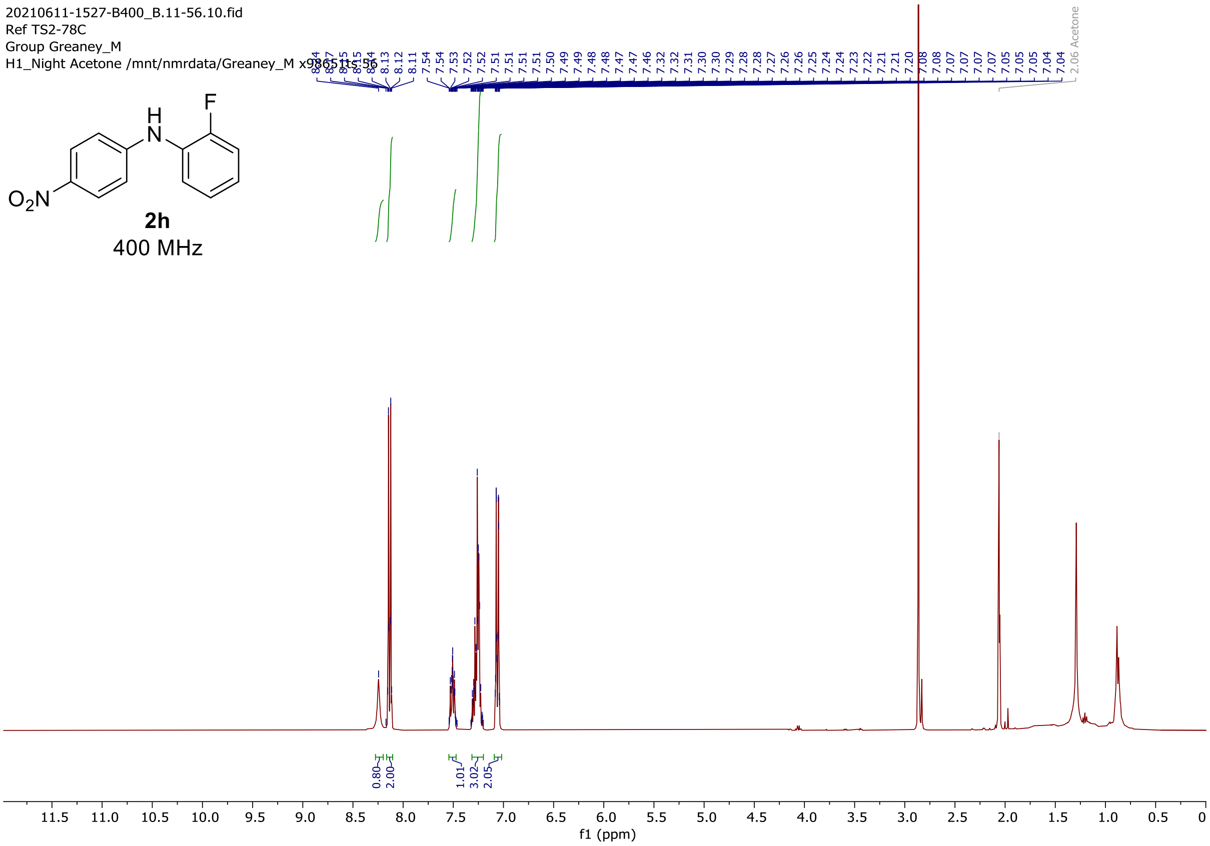
20210611-1527-B400_B.11-56.10.fid
Ref TS2-78C
Group Greaney_M
H1_Night Acetone /mnt/nmrdata/Greaney_M
2.06 Acetone
H
N
F
O2N
2h
400 MHz
0.80
2.00
1.01
3.02
2.05
f1 (ppm)

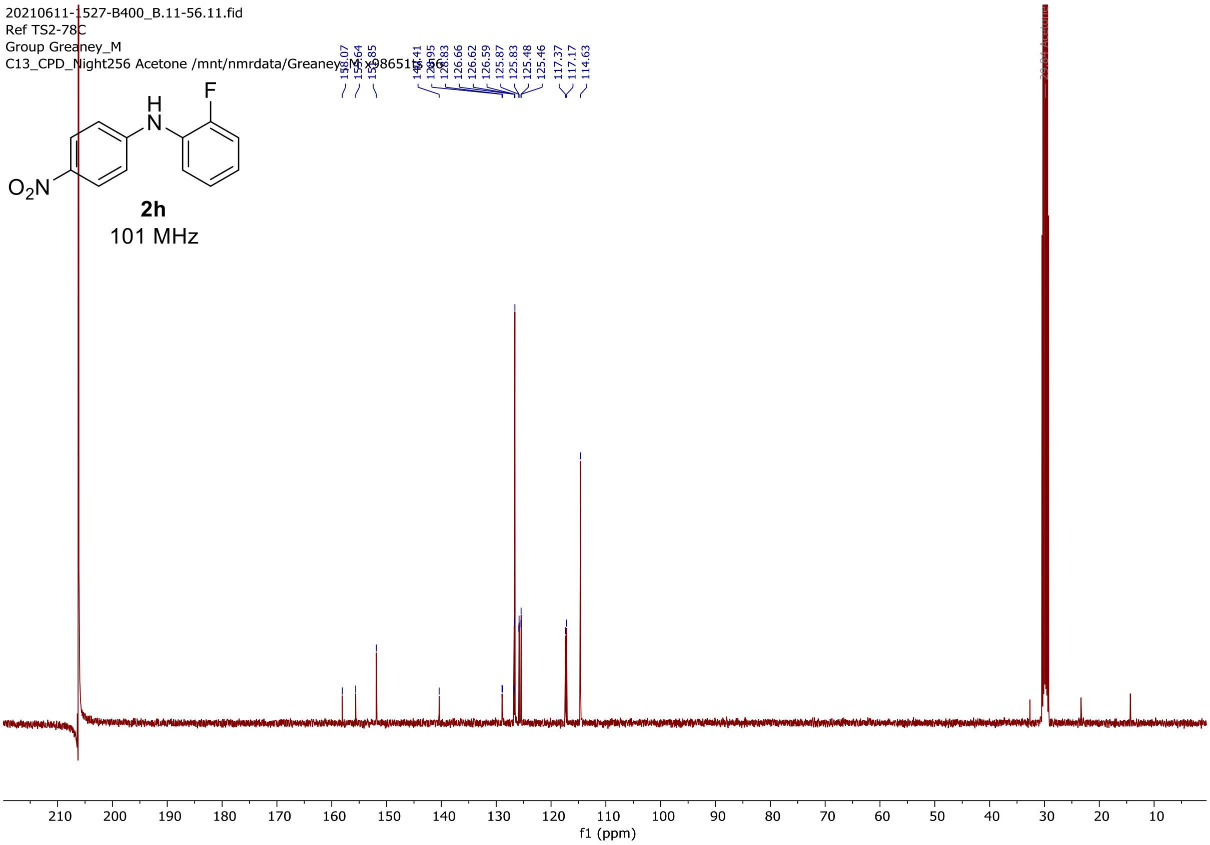
20210611-1527-B400_B.11-56.11.fid
Ref TS2-78C
Group Greaney_M
C13_CPD_Night256 Acetone /mnt/nmrdata/Greaney_M x98651ts 56
128.95
128.83
126.66
126.62
126.59
125.87
125.83
125.48
125.46
117.37
117.17
114.63
H
N
F
O2N
2h
101 MHz
210 200 190 180 170 160 150 140 130 120 110 100 90 80 70 60 50 40 30 20 10
f1 (ppm)

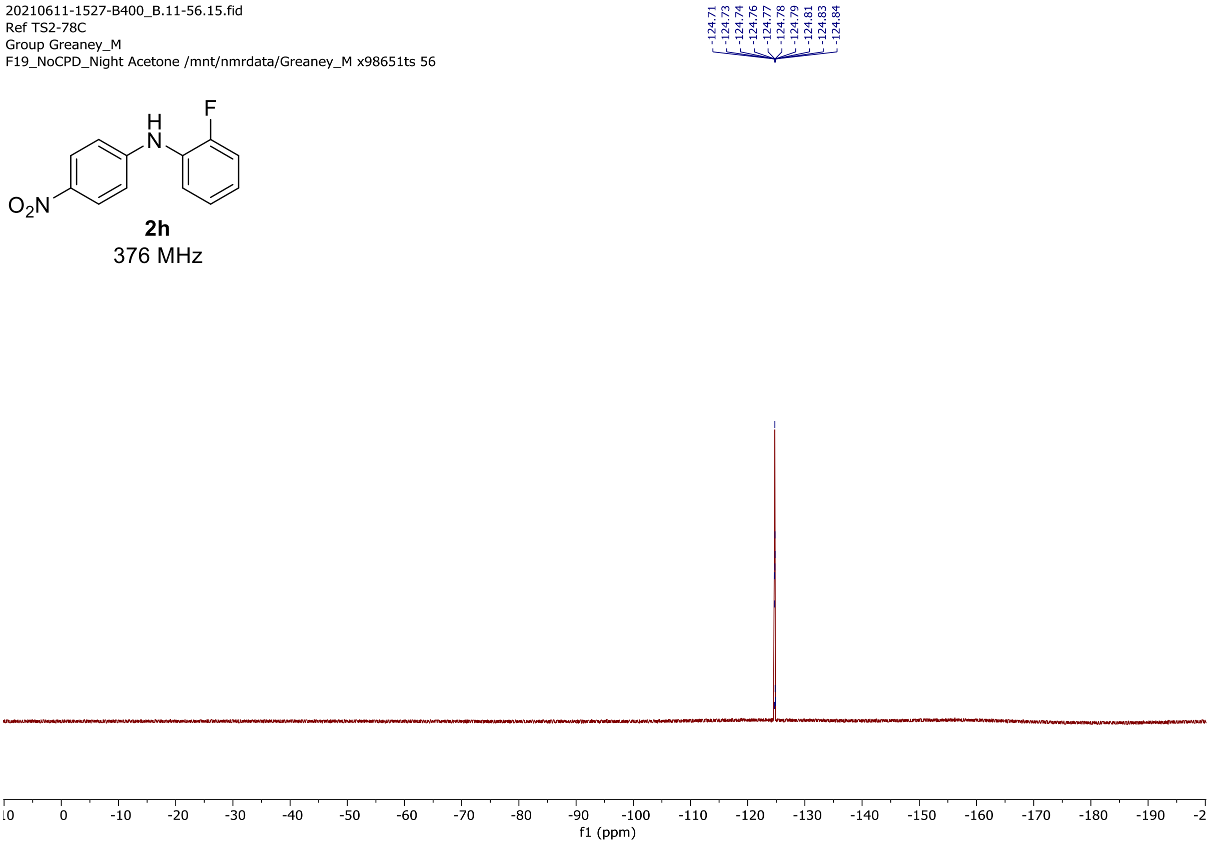
20210611-1527-B400_B.11-56.15.fid
Ref TS2-78C
Group Greaney_M
F19_NoCPD_Night Acetone /mnt/nmrdata/Greaney_M x98651ts 56
-124.71
-124.73
-124.74
-124.76
-124.77
-124.78
-124.79
-124.81
-124.83
-124.84
H
N
F
O2N
2h
376 MHz
0 -10 -20 -30 -40 -50 -60 -70 -80 -90 -100 -110 -120 -130 -140 -150 -160 -170 -180 -190
f1 (ppm)

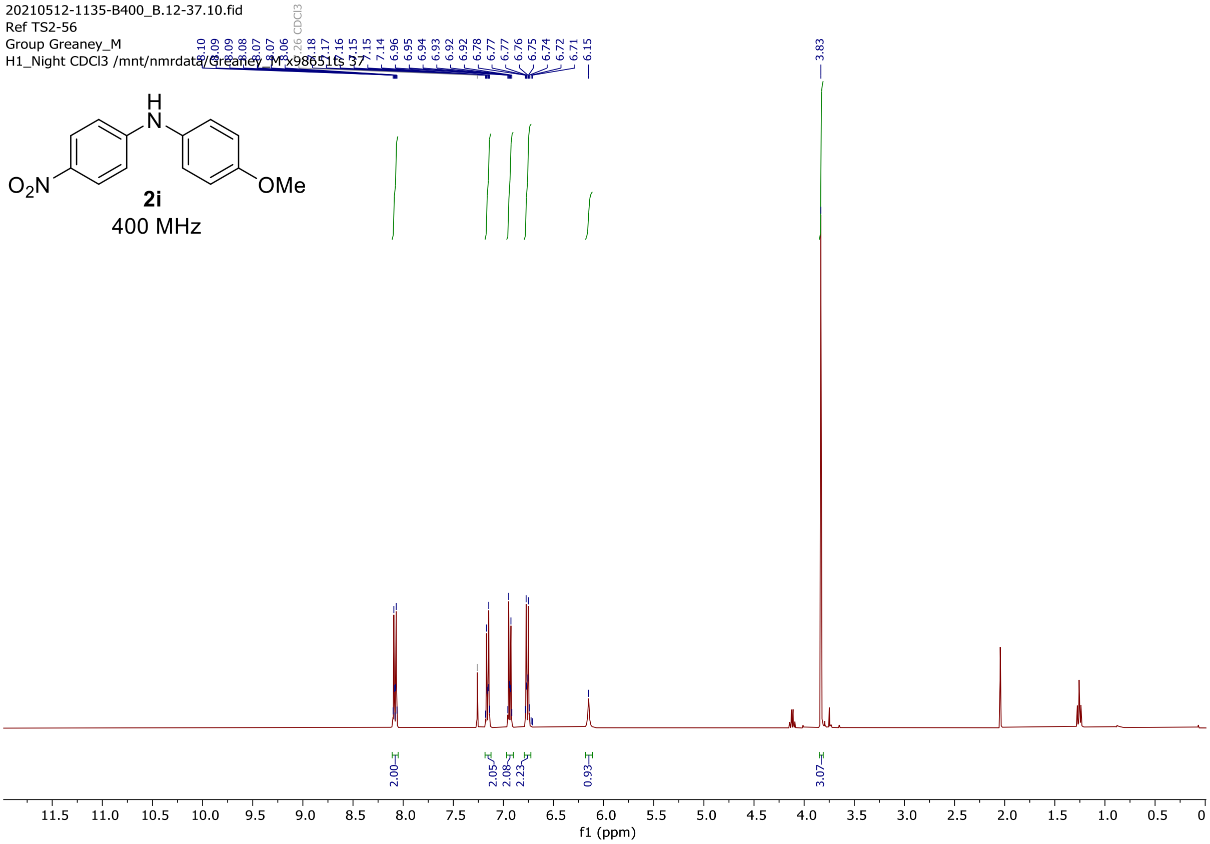
20210512-1135-B400_B.12-37.10.fid
Ref TS2-56
Group Greaney_M
H1_Night CDCl3 /mnt/nmrdata/Greaney_M x98651ts 37
2i
400 MHz
3.83
6.15
2.00
2.05
2.08
2.23
0.93
3.07
f1 (ppm)

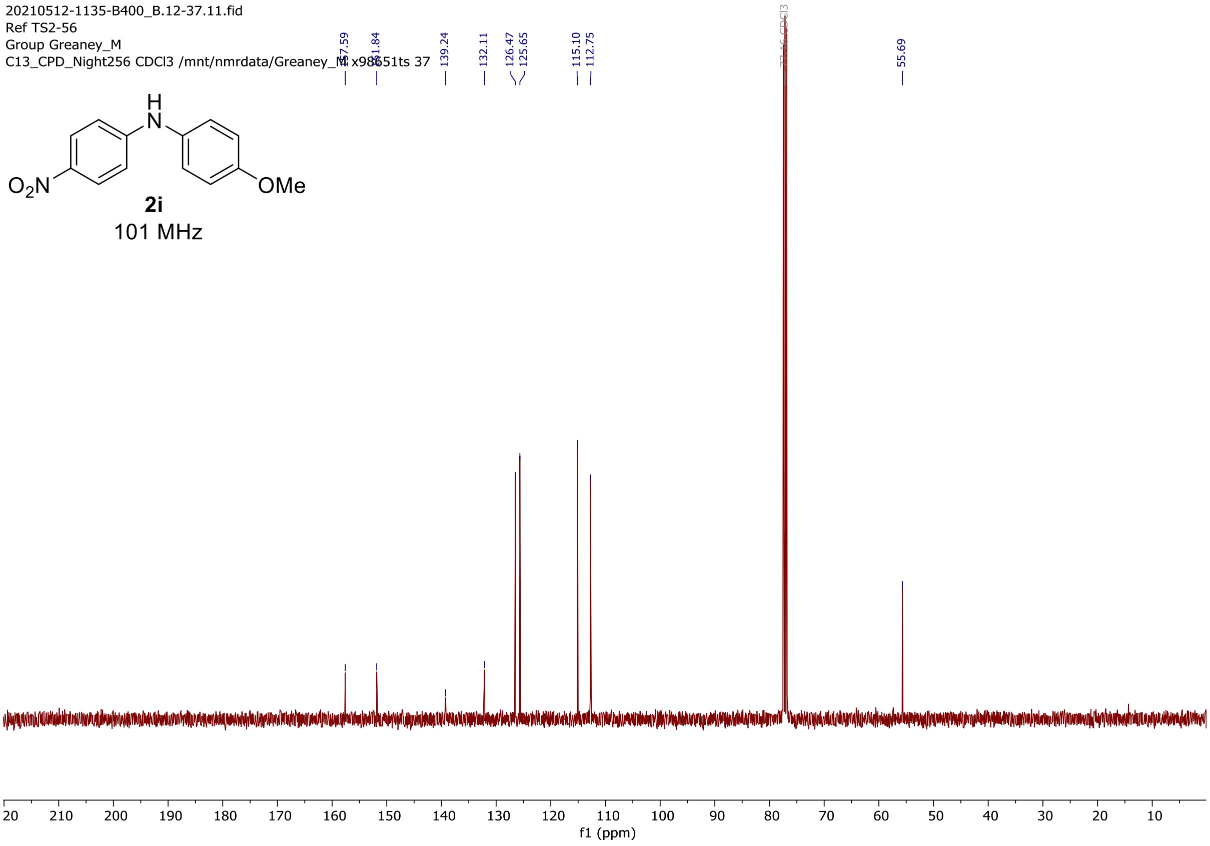
20210512-1135-B400_B.12-37.11.fid
Ref TS2-56
Group Greaney_M
C13_CPD_Night256 CDCl3 /mnt/nmrdata/Greaney_M x98651ts 37
2i
101 MHz
139.24
132.11
126.47
125.65
115.10
112.75
55.69
f1 (ppm)

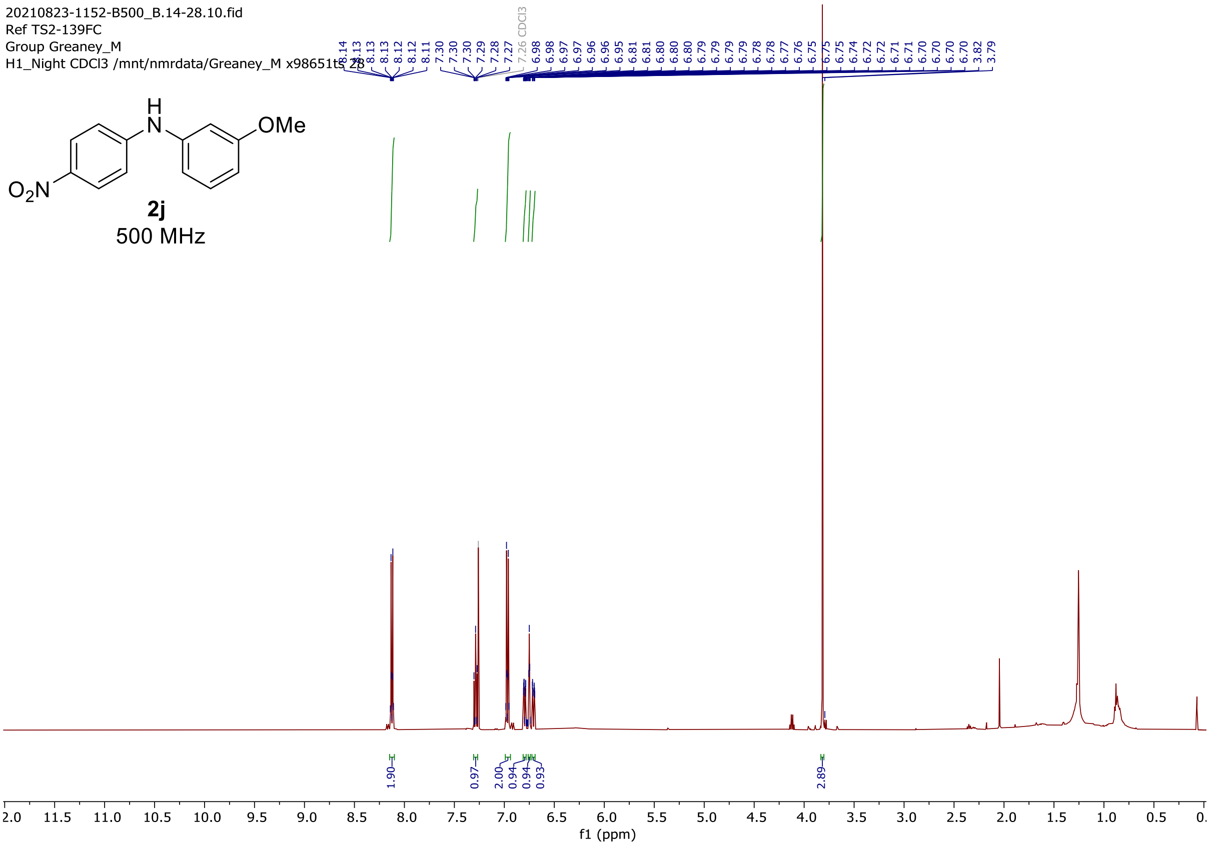
20210823-1152-B500_B.14-28.10.fid
Ref TS2-139FC
Group Greaney_M
H1_Night CDCl3 /mnt/nmrdata/Greaney_M x98651ts 28
8.14 8.13 8.13 8.13 8.12 8.12 8.11 7.30 7.30 7.29 7.28 7.27 7.26 CDCl3 6.98 6.98 6.97 6.97 6.96 6.96 6.95 6.81 6.81 6.80 6.80 6.80 6.79 6.79 6.79 6.79 6.78 6.78 6.77 6.76 6.75 6.75 6.75 6.74 6.72 6.72 6.71 6.71 6.70 6.70 6.70 6.70 3.82 3.79
H
N
OMe
O2N
2j
500 MHz
1.90 0.97 2.00 0.94 0.94 0.93 2.89
f1 (ppm)

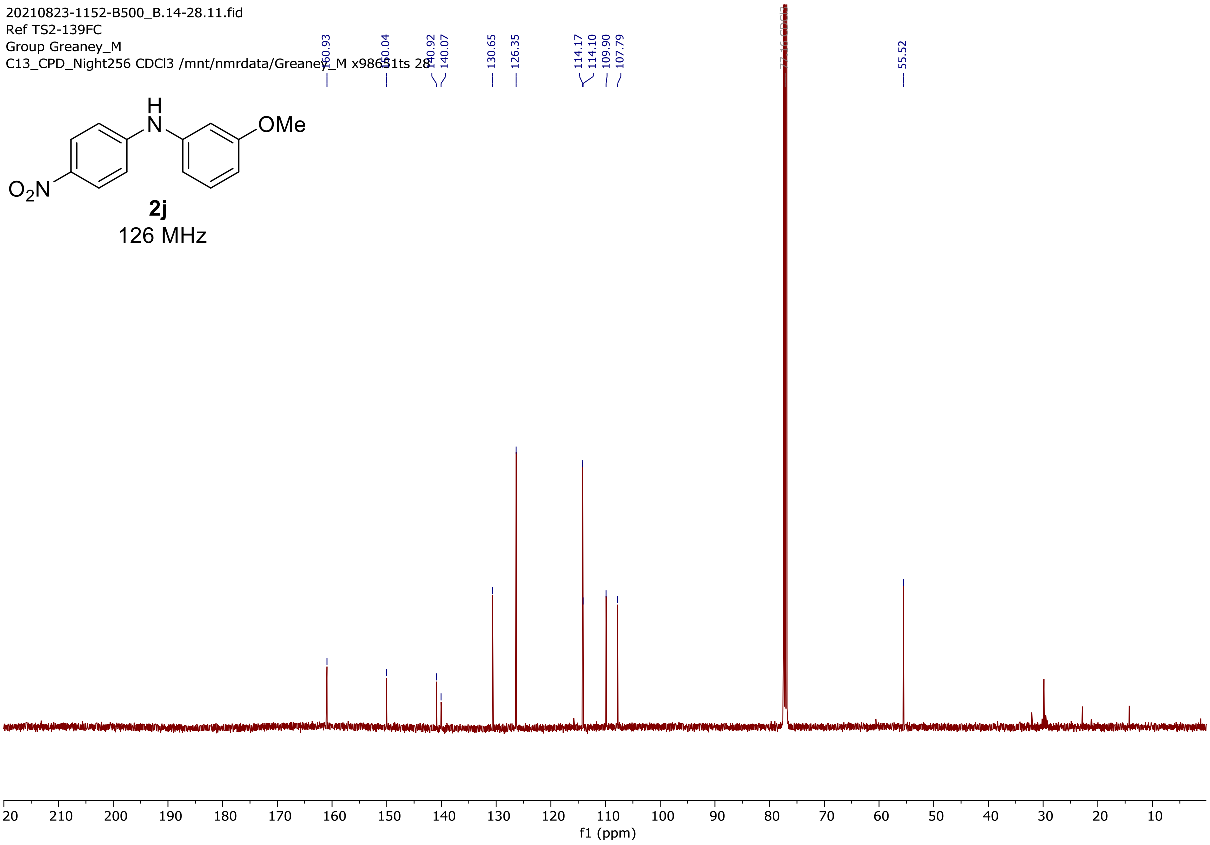
20210823-1152-B500_B.14-28.11.fid
Ref TS2-139FC
Group Greaney_M
C13_CPD_Night256 CDCl3 /mnt/nmrdata/Greaney_M x98651ts 28
160.93 150.04 140.92 140.07 130.65 126.35 114.17 114.10 109.90 107.79 77.16 CDCl3 55.52
H
N
OMe
O2N
2j
126 MHz
f1 (ppm)

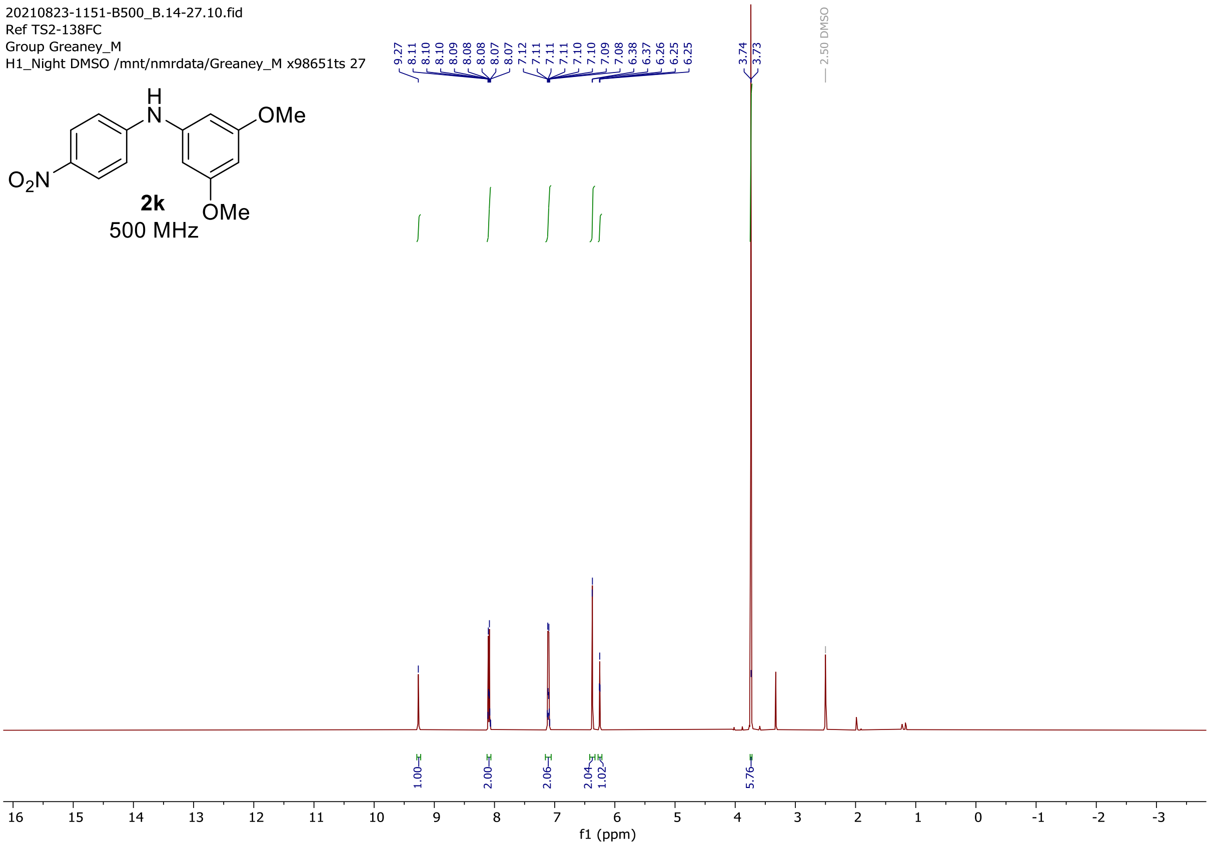
20210823-1151-B500_B.14-27.10.fid
Ref TS2-138FC
Group Greaney_M
H1_Night DMSO /mnt/nmrdata/Greaney_M x98651ts 27
2k
500 MHz
f1 (ppm)

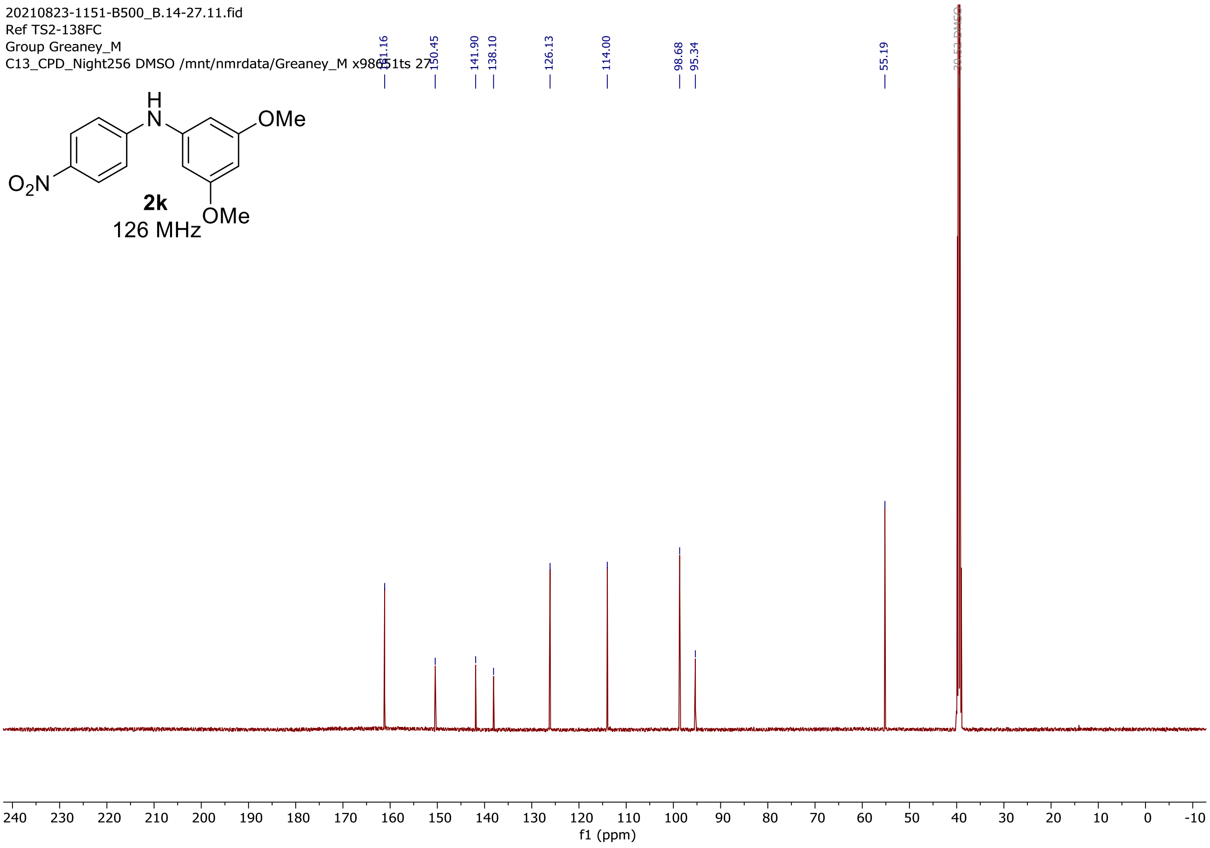
20210823-1151-B500_B.14-27.11.fid
Ref TS2-138FC
Group Greaney_M
C13_CPD_Night256 DMSO /mnt/nmrdata/Greaney_M x98651ts 27
2k
126 MHz
f1 (ppm)

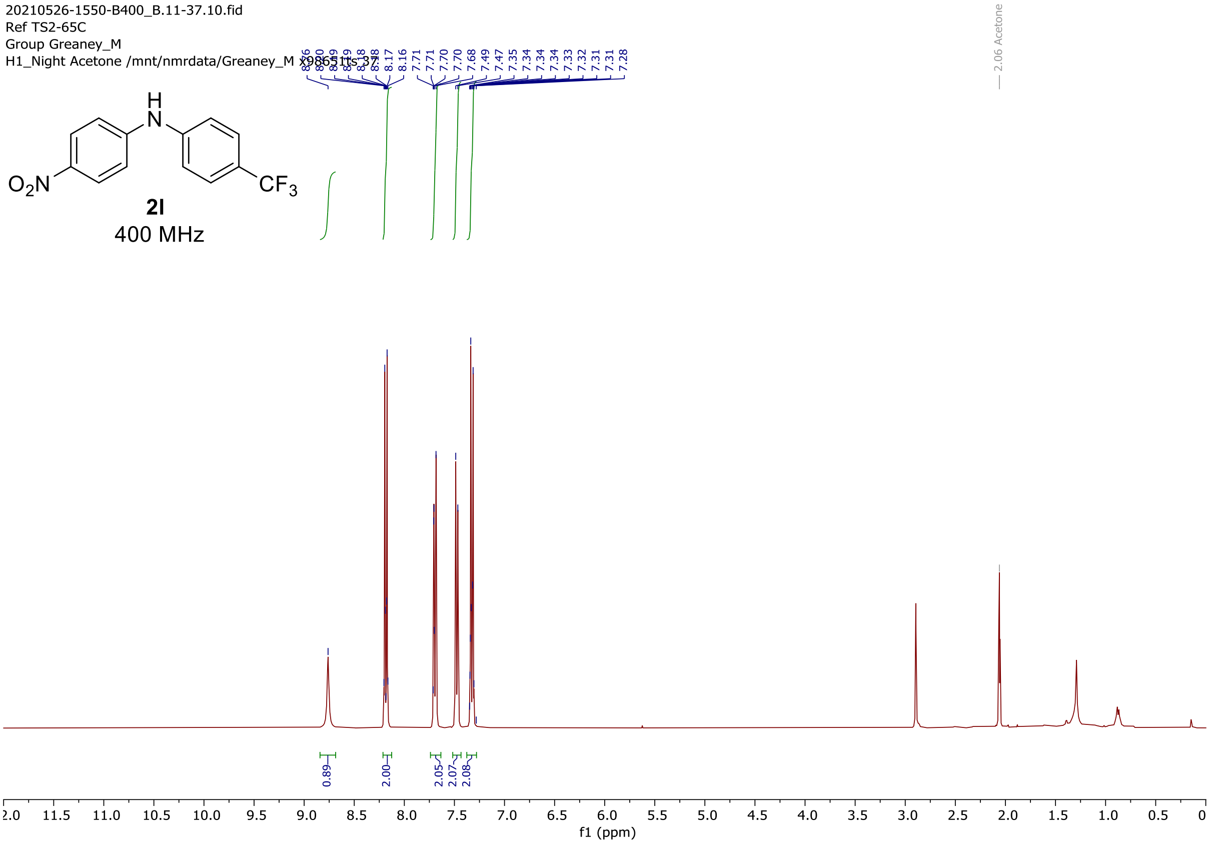
20210526-1550-B400_B.11-37.10.fid
Ref TS2-65C
Group Greaney_M
H1_Night Acetone /mnt/nmrdata/Greaney_M
2.06 Acetone
O2N
CF3
2l
400 MHz
0.89
2.00
2.05
2.07
2.08
f1 (ppm)

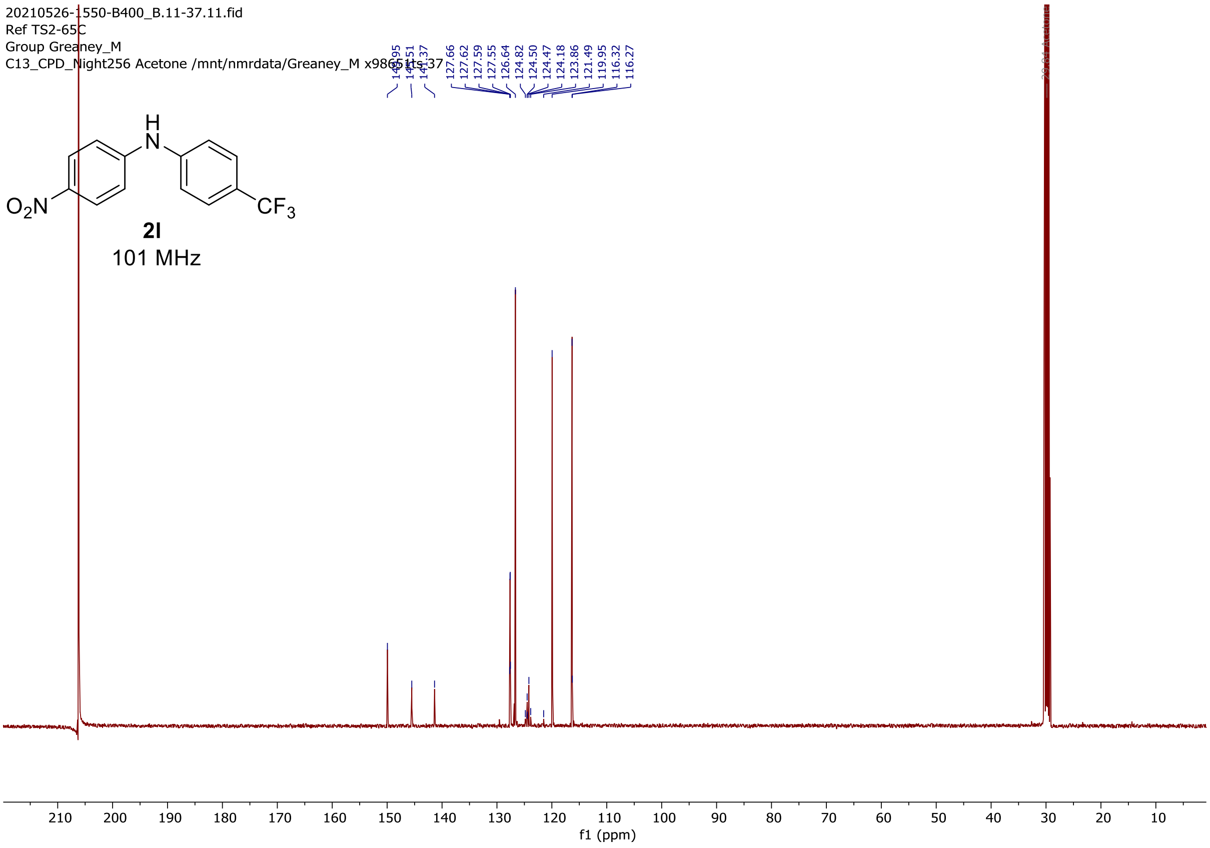
20210526-1550-B400_B.11-37.11.fid
Ref TS2-65C
Group Greaney_M
C13_CPD_Night256 Acetone /mnt/nmrdata/Greaney_M
127.66
127.62
127.59
127.55
126.64
124.82
124.50
124.47
124.18
123.86
121.49
119.95
116.32
116.27
O2N
CF3
2l
101 MHz
f1 (ppm)

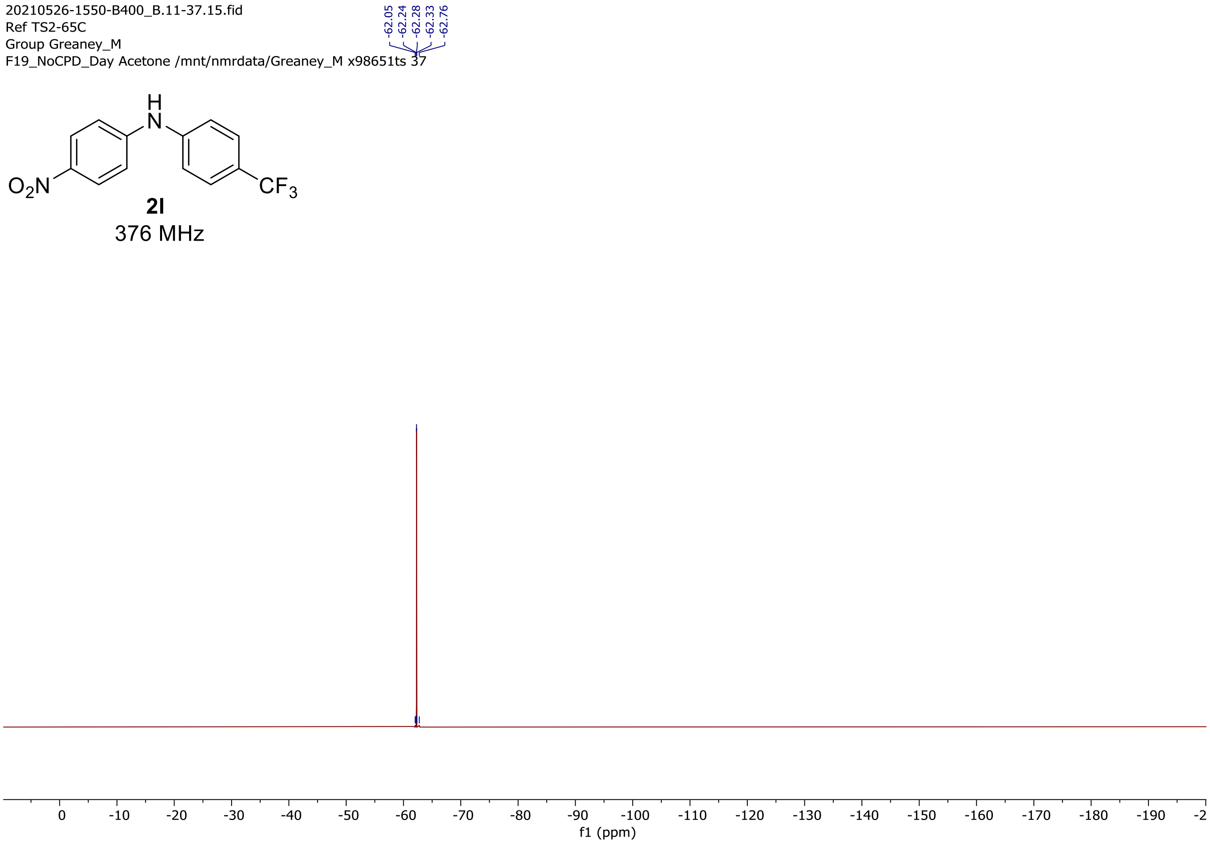
20210526-1550-B400_B.11-37.15.fid
Ref TS2-65C
Group Greaney_M
F19_NoCPD_Day Acetone /mnt/nmrdata/Greaney_M x98651ts 37
-62.05
-62.24
-62.28
-62.33
-62.76
H
N
O2N
CF3
2l
376 MHz
f1 (ppm)

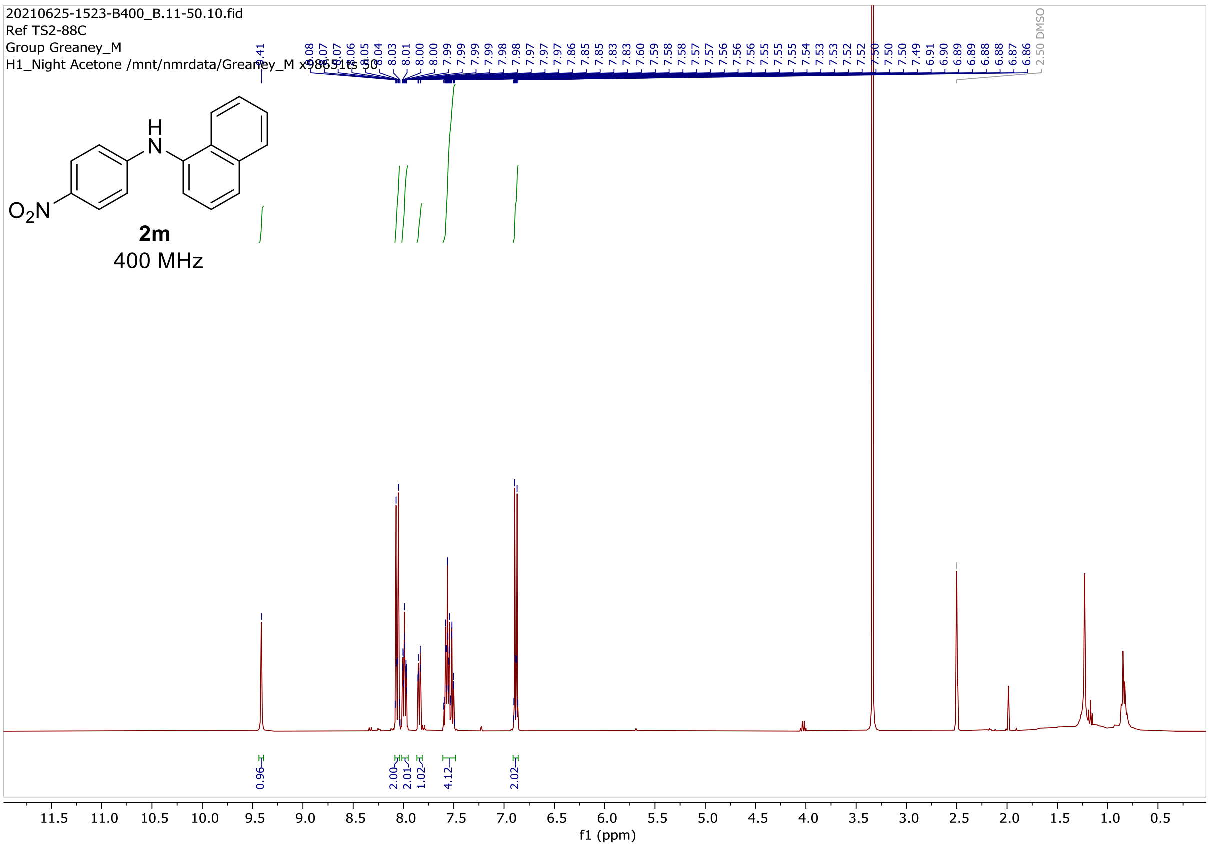
20210625-1523-B400_B.11-50.10.fid
Ref TS2-88C
Group Greaney_M
H1_Night Acetone /mnt/nmrdata/Greaney_M x98651ts 50
2.50 DMSO
H
N
O2N
2m
400 MHz
0.96
2.00
2.01
1.02
4.12
2.02
f1 (ppm)

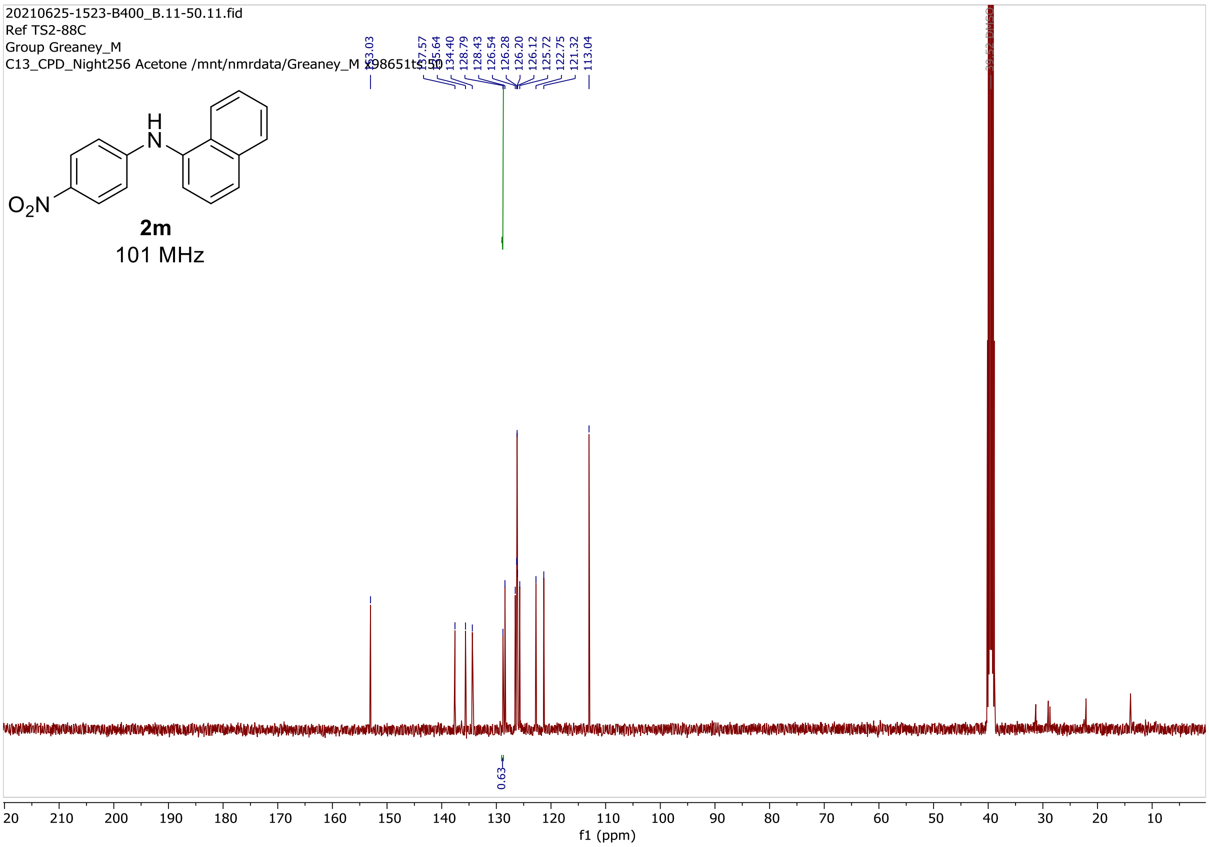

2m
101 MHz

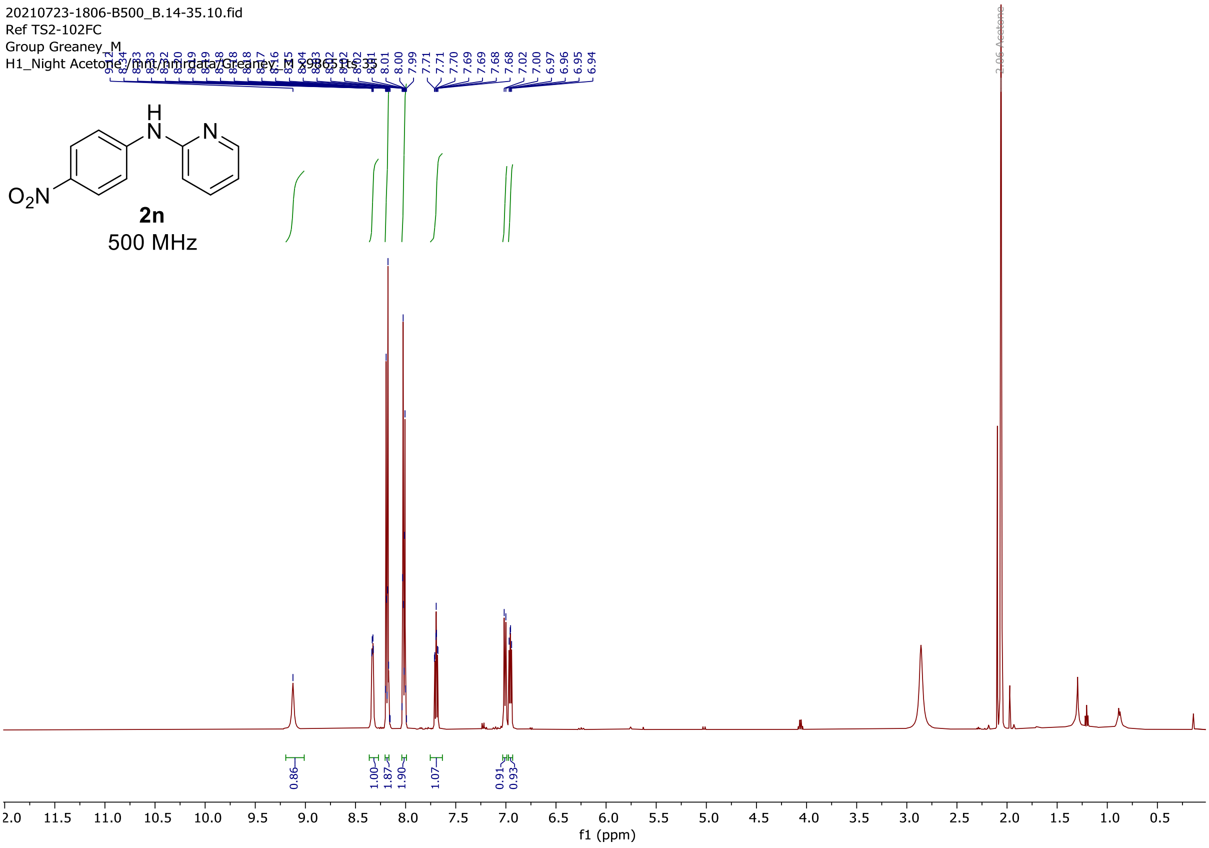

2n
500 MHz

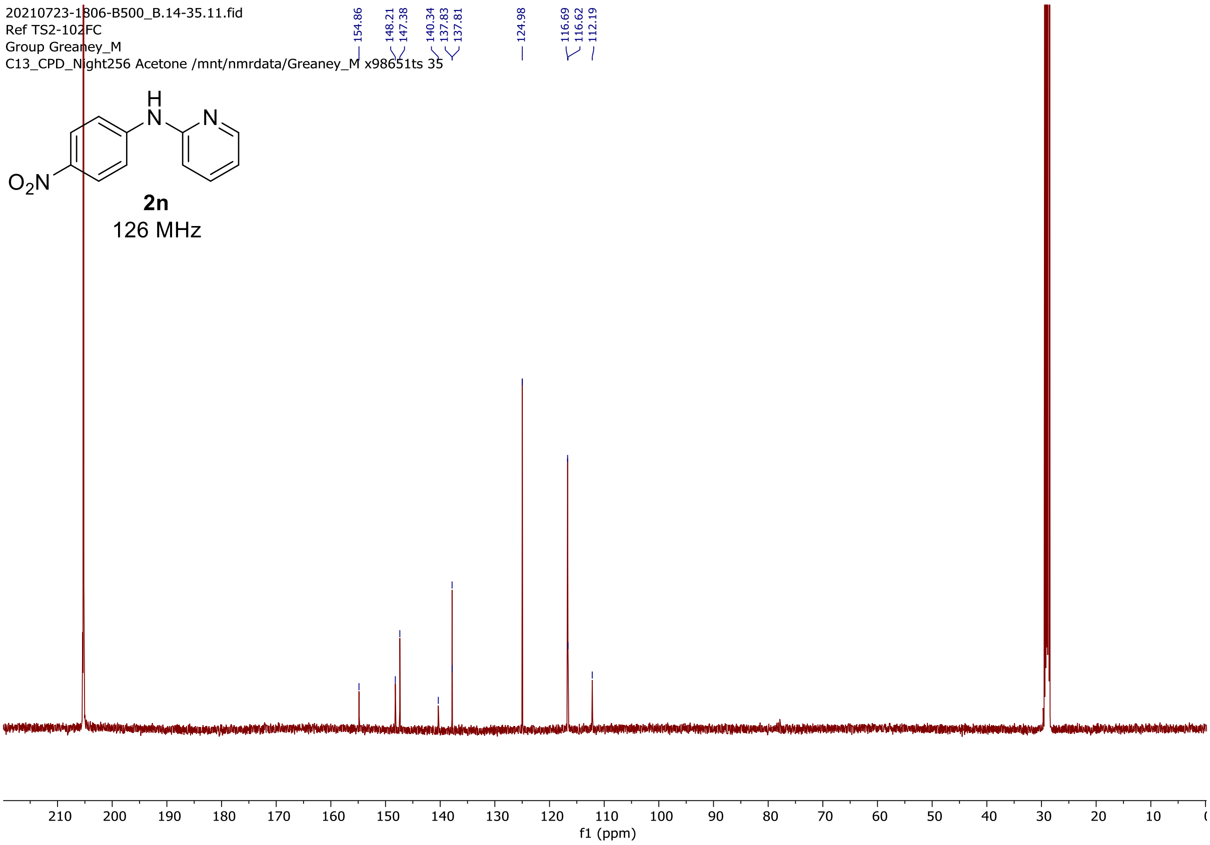
20210723-1806-B500_B.14-35.11.fid
Ref TS2-102FC
Group Greaney_M
C13_CPD_Night256 Acetone /mnt/nmrdata/Greaney_M x98651ts 35
154.86
148.21
147.38
140.34
137.83
137.81
124.98
116.69
116.62
112.19
H
N
N
O_2N
2n
126 MHz
f1 (ppm)

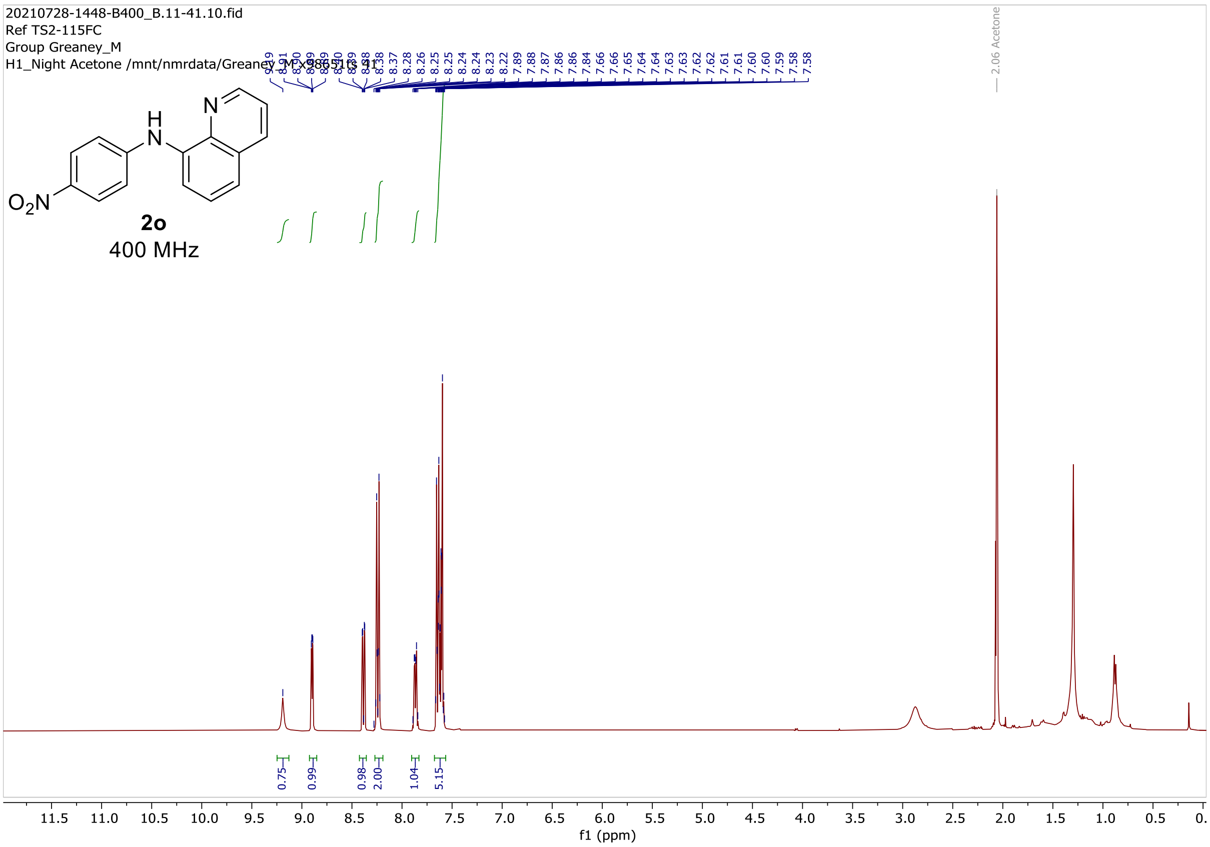
20210728-1448-B400_B.11-41.10.fid
Ref TS2-115FC
Group Greaney_M
H1_Night Acetone /mnt/nmrdata/Greaney_M x98651ts 41
8.37
8.28
8.26
8.25
8.24
8.24
8.23
8.22
7.89
7.88
7.87
7.86
7.86
7.84
7.66
7.65
7.64
7.64
7.63
7.62
7.62
7.61
7.60
7.59
7.58
7.58
2.06 Acetone
N
H
N
O_2N
2o
400 MHz
0.75
0.99
0.98
2.00
1.04
5.15
f1 (ppm)

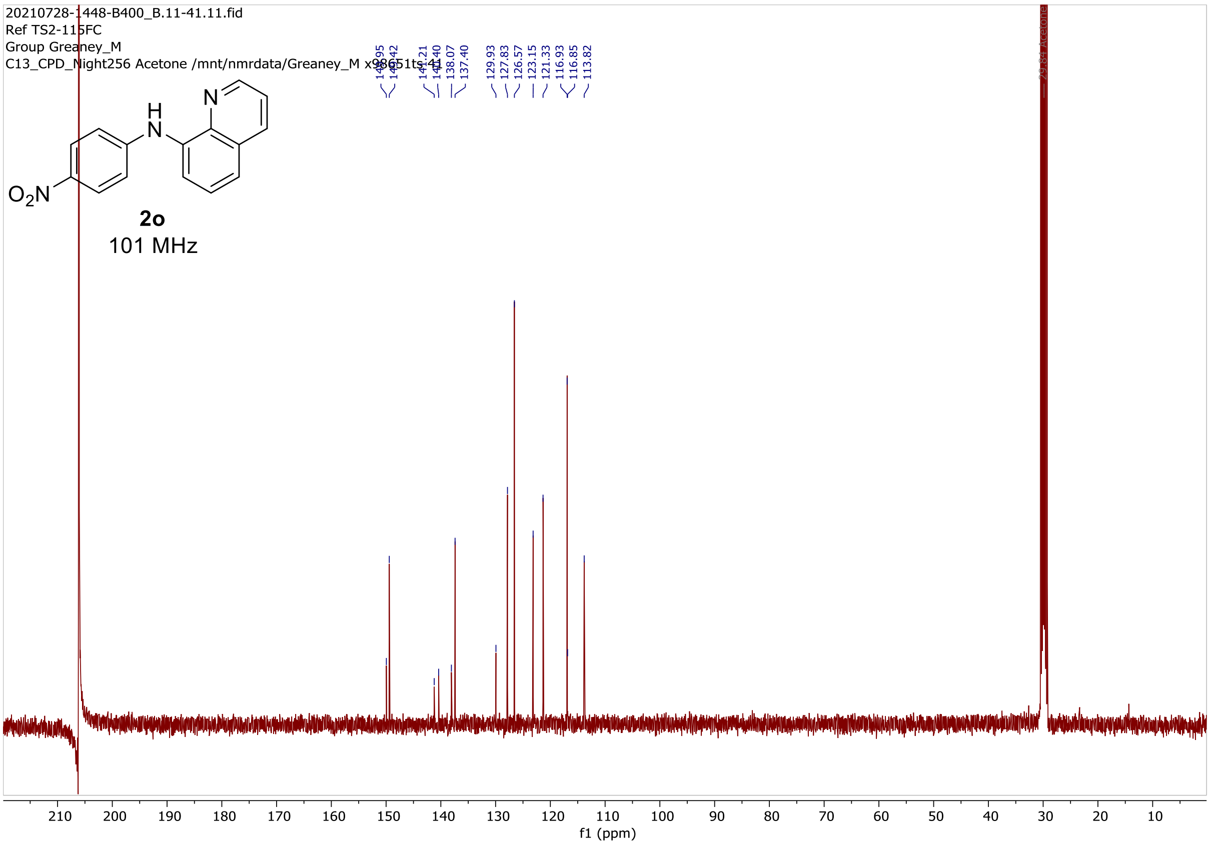

20210728-1448-B400_B.11-41.11.fid
Ref TS2-115FC
Group Greaney_M
C13_CPD_Night256 Acetone /mnt/nmrdata/Greaney_M x98651ts 41
149.95
149.42
141.21
140.40
138.07
137.40
129.93
127.83
126.57
123.15
121.33
116.93
116.85
113.82
H
N
N
O_2N
2o
101 MHz
f1 (ppm)

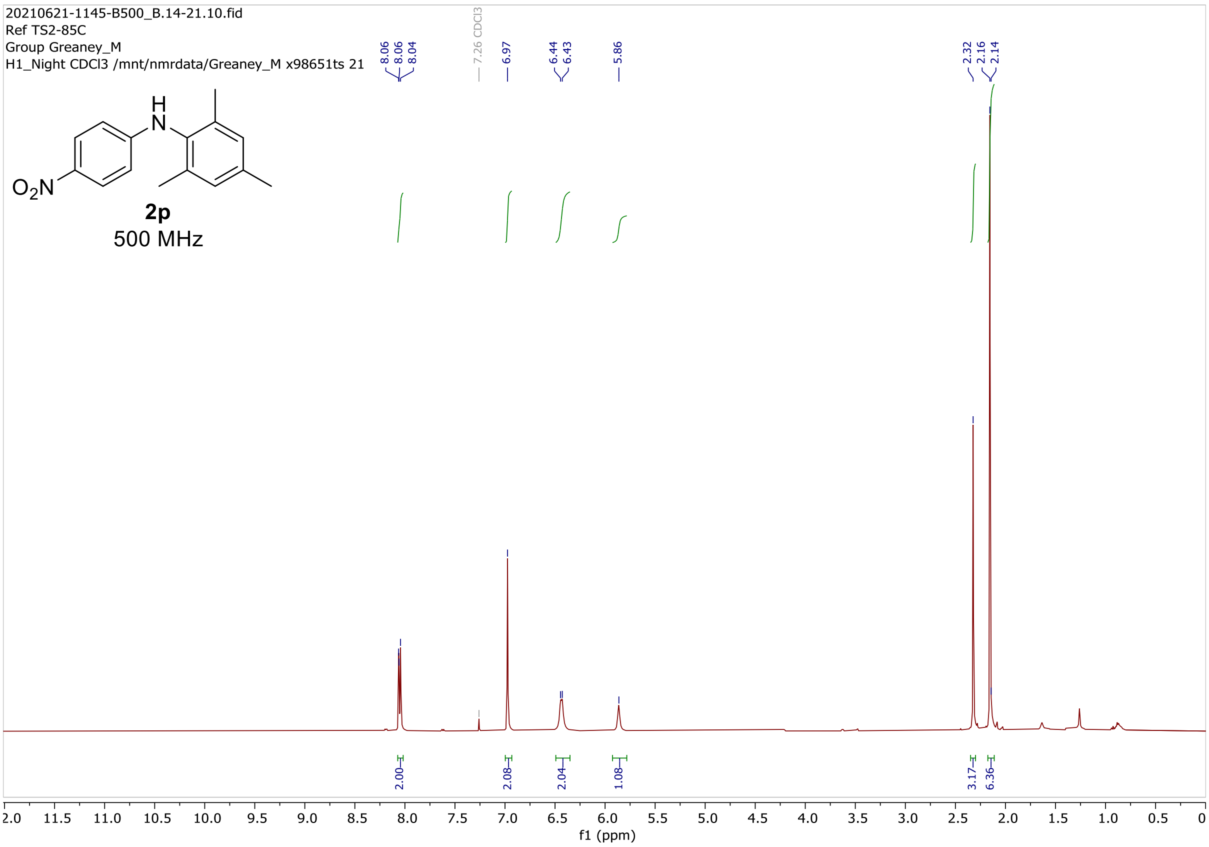

20210621-1145-B500_B.14-21.10.fid
Ref TS2-85C
Group Greaney_M
H1_Night CDCl3 /mnt/nmrdata/Greaney_M x98651ts 21
8.06
8.06
8.04
7.26 CDCl3
6.97
6.44
6.43
5.86
2.32
2.16
2.14
H
N
O_2N
2p
500 MHz
2.00
2.08
2.04
1.08
3.17
6.36
f1 (ppm)

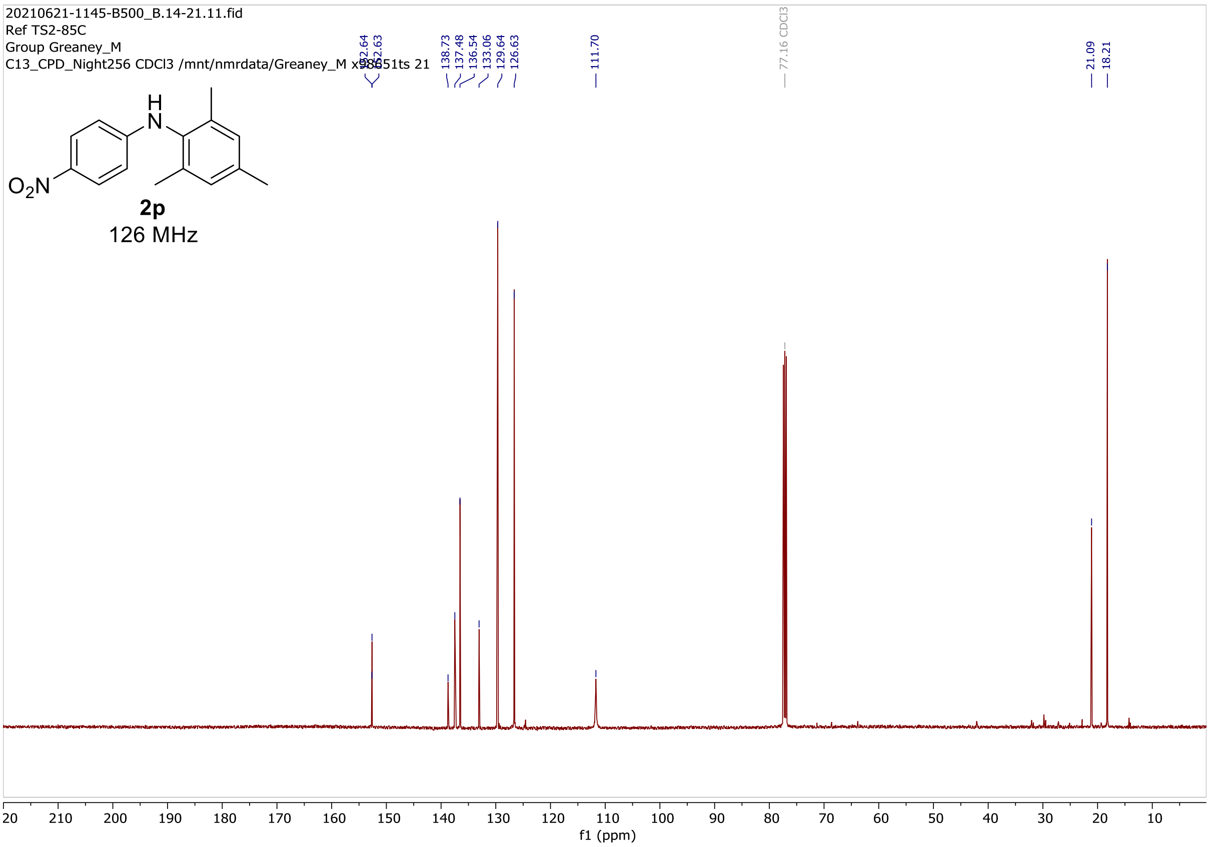
20210621-1145-B500_B.14-21.11.fid
Ref TS2-85C
Group Greaney_M
C13_CPD_Night256 CDCl3 /mnt/nmrdata/Greaney_M x98651ts 21
152.64
152.63
138.73
137.48
136.54
133.06
129.64
126.63
111.70
77.16 CDCl3
21.09
18.21
H
N
O_2N
2p
126 MHz
f1 (ppm)

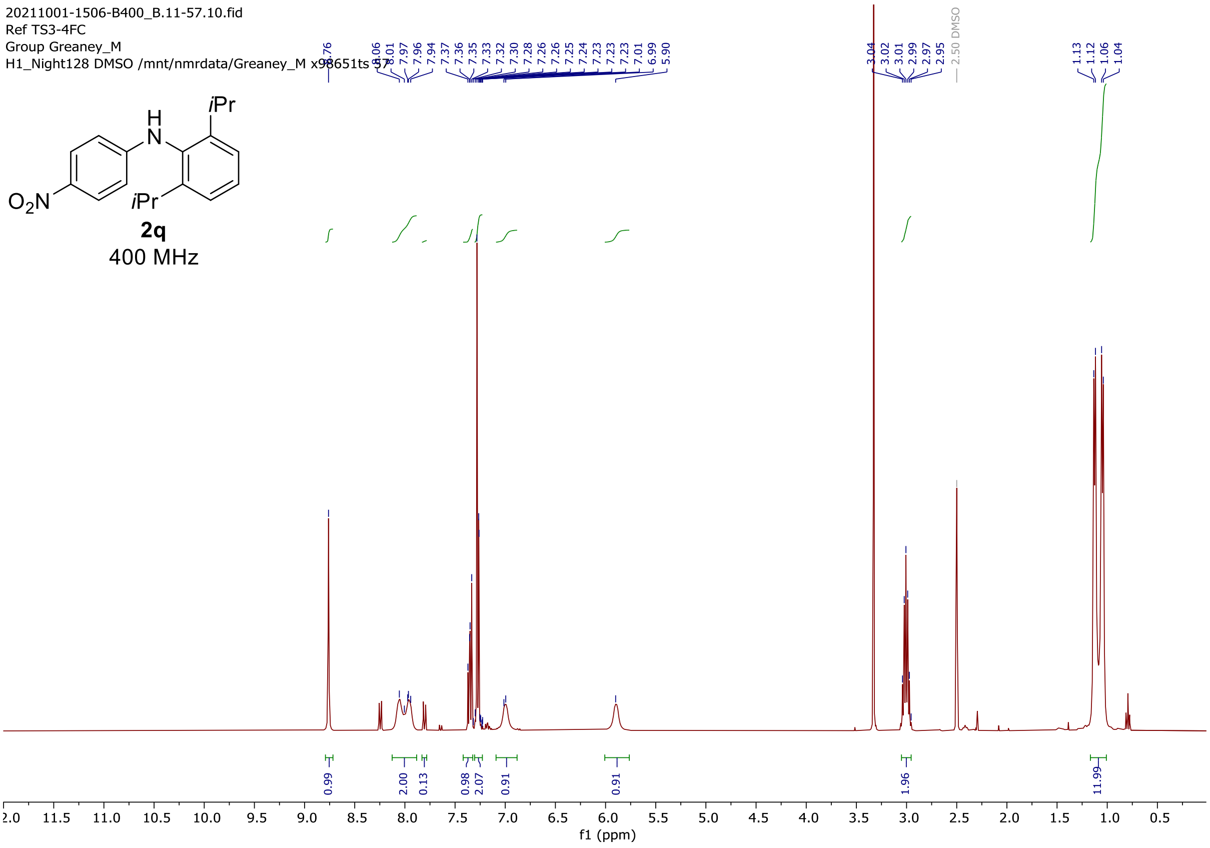
20211001-1506-B400_B.11-57.10.fid
Ref TS3-4FC
Group Greaney_M
H1_Night128 DMSO /mnt/nmrdata/Greaney_M x98651ts 57
8.76
8.06
8.01
7.97
7.96
7.94
7.37
7.36
7.35
7.33
7.32
7.30
7.28
7.26
7.25
7.24
7.23
7.23
7.23
7.01
6.99
5.90
3.04
3.02
3.01
2.99
2.97
2.95
2.50 DMSO
1.13
1.12
1.06
1.04
H
N
iPr
iPr
O_2N
2q
400 MHz
0.99
2.00
0.13
0.98
2.07
0.91
0.91
1.96
11.99
f1 (ppm)

20211001-1506-B400_B.11-57.11.fid
Ref TS3-4FC
Group Greaney_M
C13_CPD_Night1024 DMSO /mnt/nmrdata/Greaney_M x98651ts 57

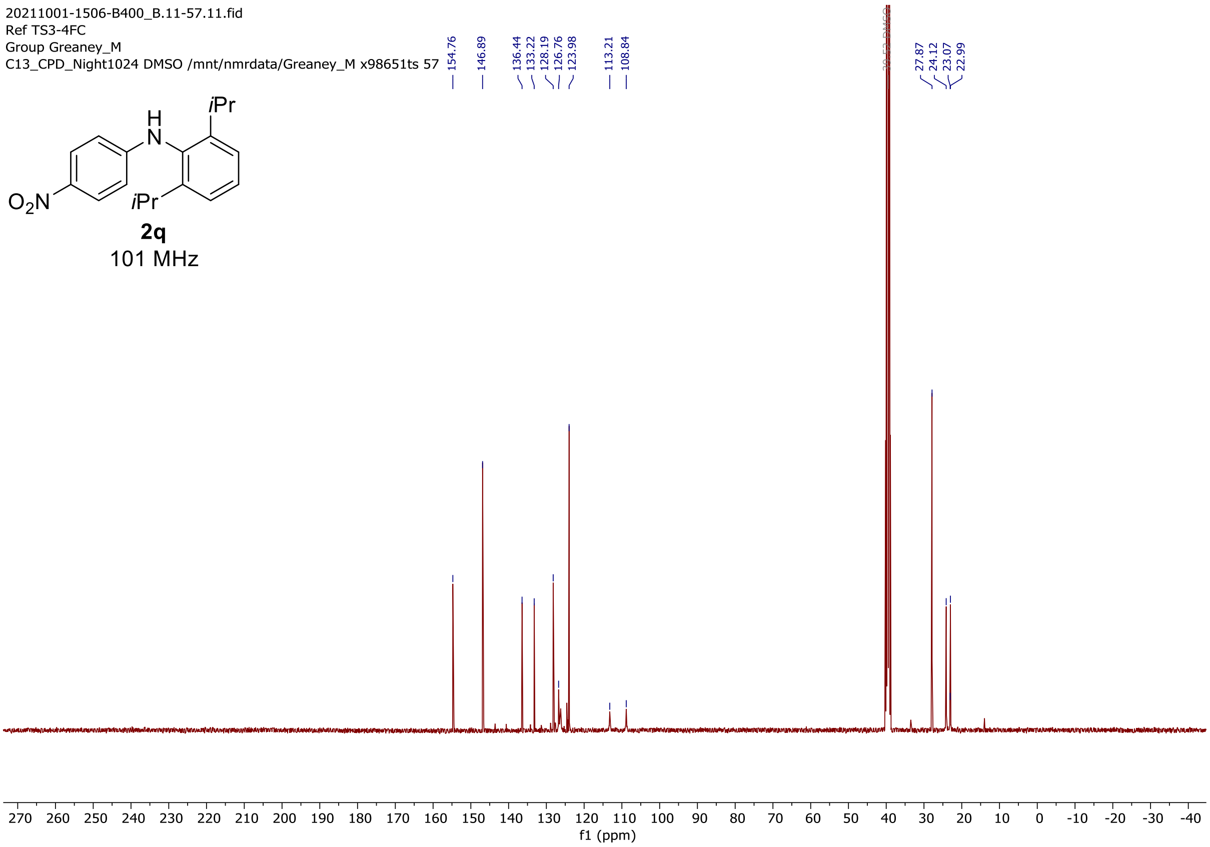

2q
101 MHz

20210816-1120-B500_B.14-11.10.fid
Ref TS2-128FC
Group Greaney_M
H1_Night DMSO /mnt/nmrdata/Greaney_M x98651ts 11

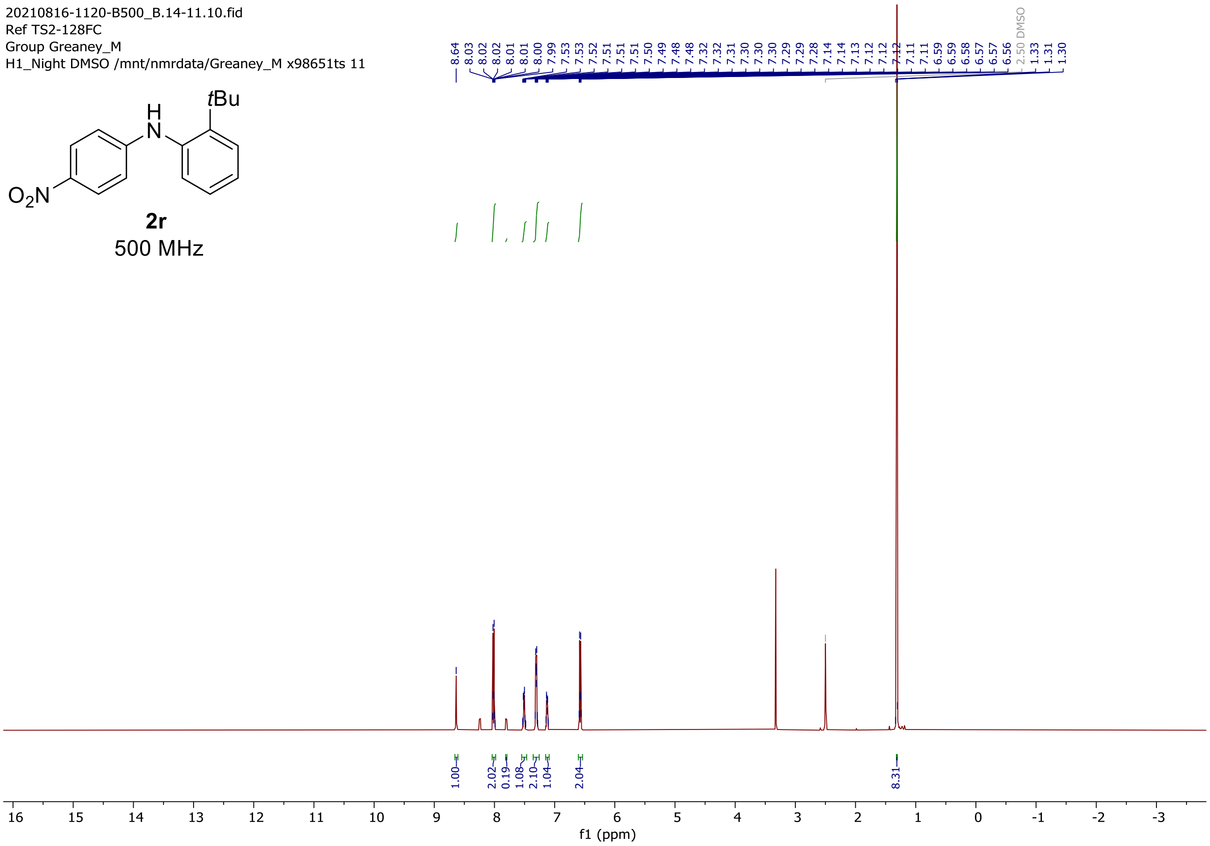

2r
500 MHz

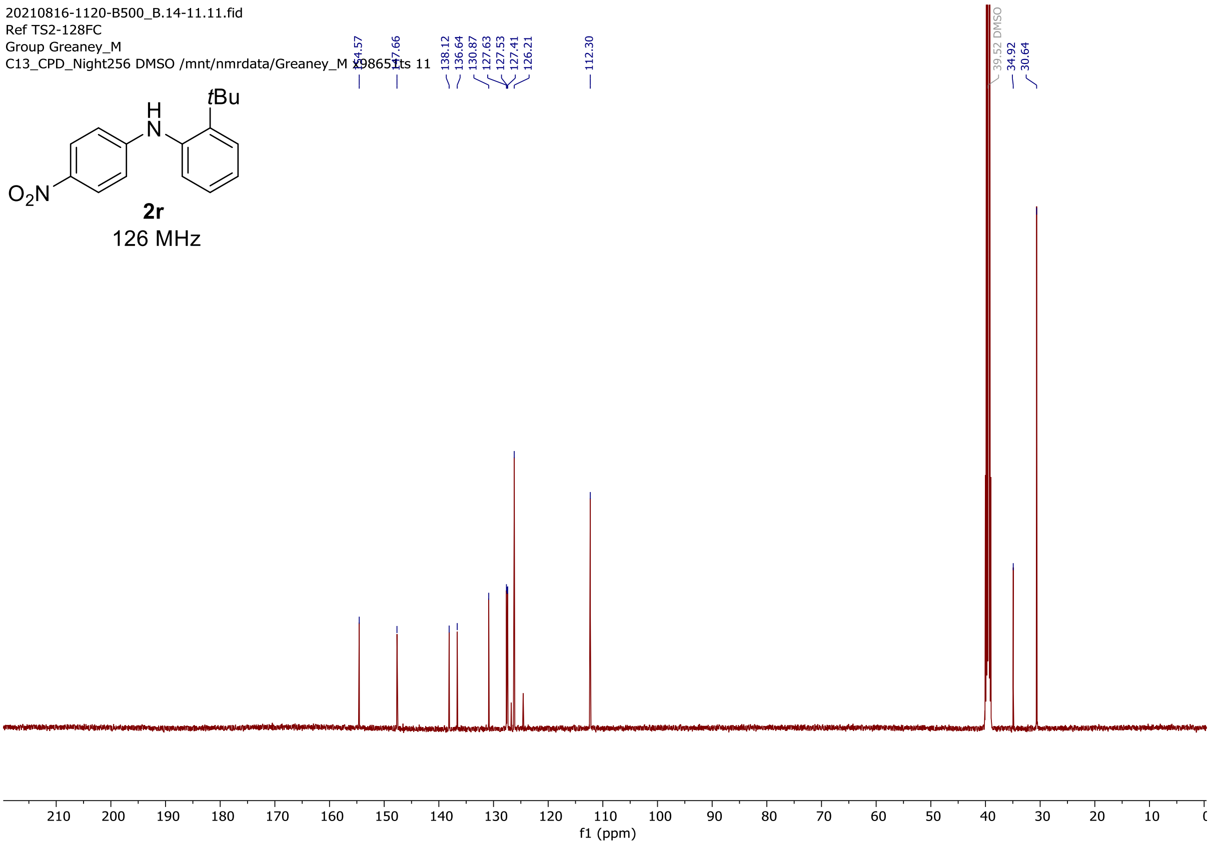
20210816-1120-B500_B.14-11.11.fid
Ref TS2-128FC
Group Greaney_M
C13_CPD_Night256 DMSO /mnt/nmrdata/Greaney_M x98651ts 11
138.12
136.64
130.87
127.63
127.53
127.41
126.21
112.30
39.52 DMSO
34.92
30.64
H
N
tBu
O2N
2r
126 MHz
f1 (ppm)

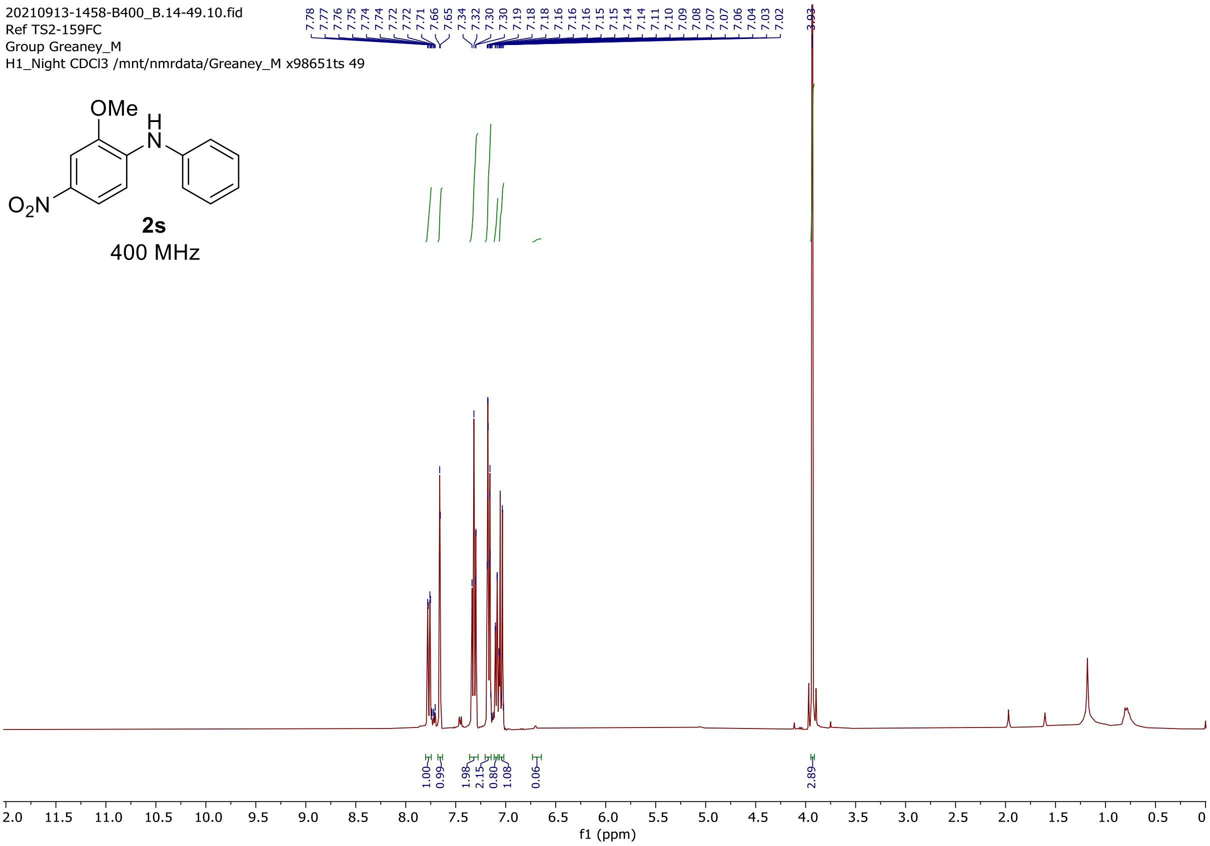
20210913-1458-B400_B.14-49.10.fid
Ref TS2-159FC
Group Greaney_M
H1_Night CDCl3 /mnt/nmrdata/Greaney_M x98651ts 49
7.78 7.77 7.76 7.75 7.74 7.74 7.72 7.72 7.71 7.66 7.65 7.34 7.32 7.30 7.30 7.19 7.18 7.16 7.16 7.15 7.15 7.14 7.14 7.11 7.10 7.09 7.08 7.07 7.06 7.04 7.03 7.02
3.93
OMe
H
N
O2N
2s
400 MHz
1.00 0.99 1.98 2.15 0.80 1.08 0.06 2.89
f1 (ppm)

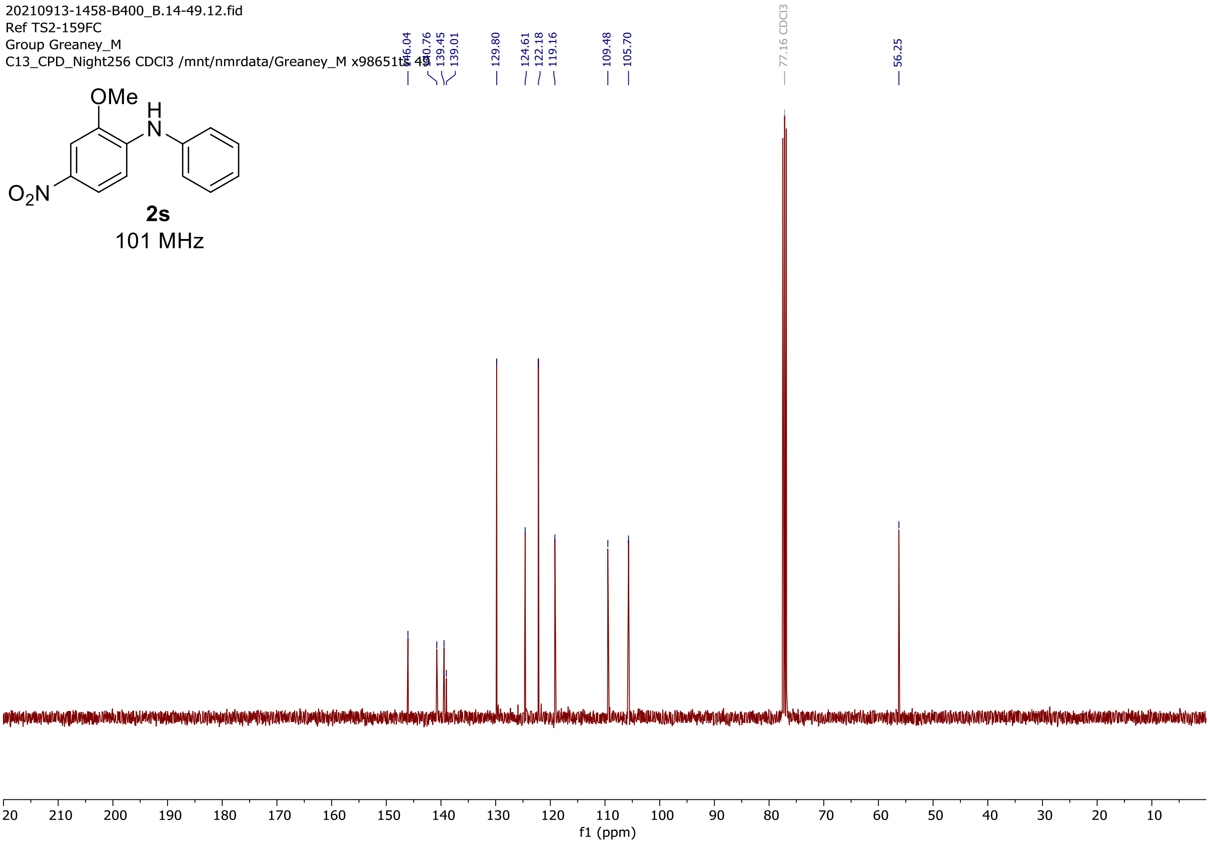
20210913-1458-B400_B.14-49.12.fid
Ref TS2-159FC
Group Greaney_M
C13_CPD_Night256 CDCl3 /mnt/nmrdata/Greaney_M
146.04
140.76
139.45
139.01
129.80
124.61
122.18
119.16
109.48
105.70
77.16 CDCl3
56.25
OMe
H
N
O_2N
2s
101 MHz
f1 (ppm)

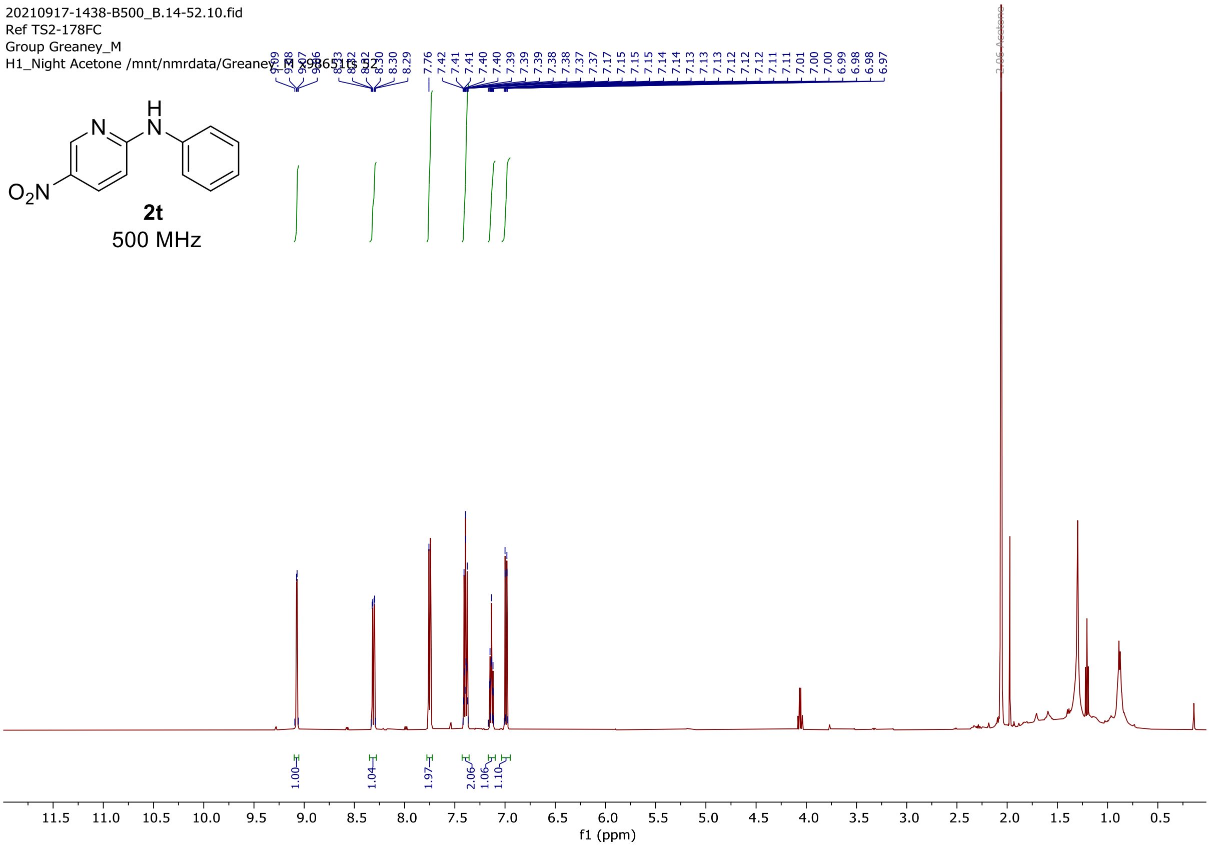
20210917-1438-B500_B.14-52.10.fid
Ref TS2-178FC
Group Greaney_M
H1_Night Acetone /mnt/nmrdata/Greaney
8.30
8.29
7.76
7.42
7.41
7.41
7.40
7.40
7.39
7.39
7.39
7.38
7.38
7.37
7.37
7.17
7.15
7.15
7.15
7.14
7.14
7.13
7.13
7.13
7.12
7.12
7.12
7.11
7.11
7.01
7.00
7.00
6.99
6.98
6.98
6.97
H
N
N
O_2N
2t
500 MHz
1.00
1.04
1.97
2.06
1.06
1.10
f1 (ppm)

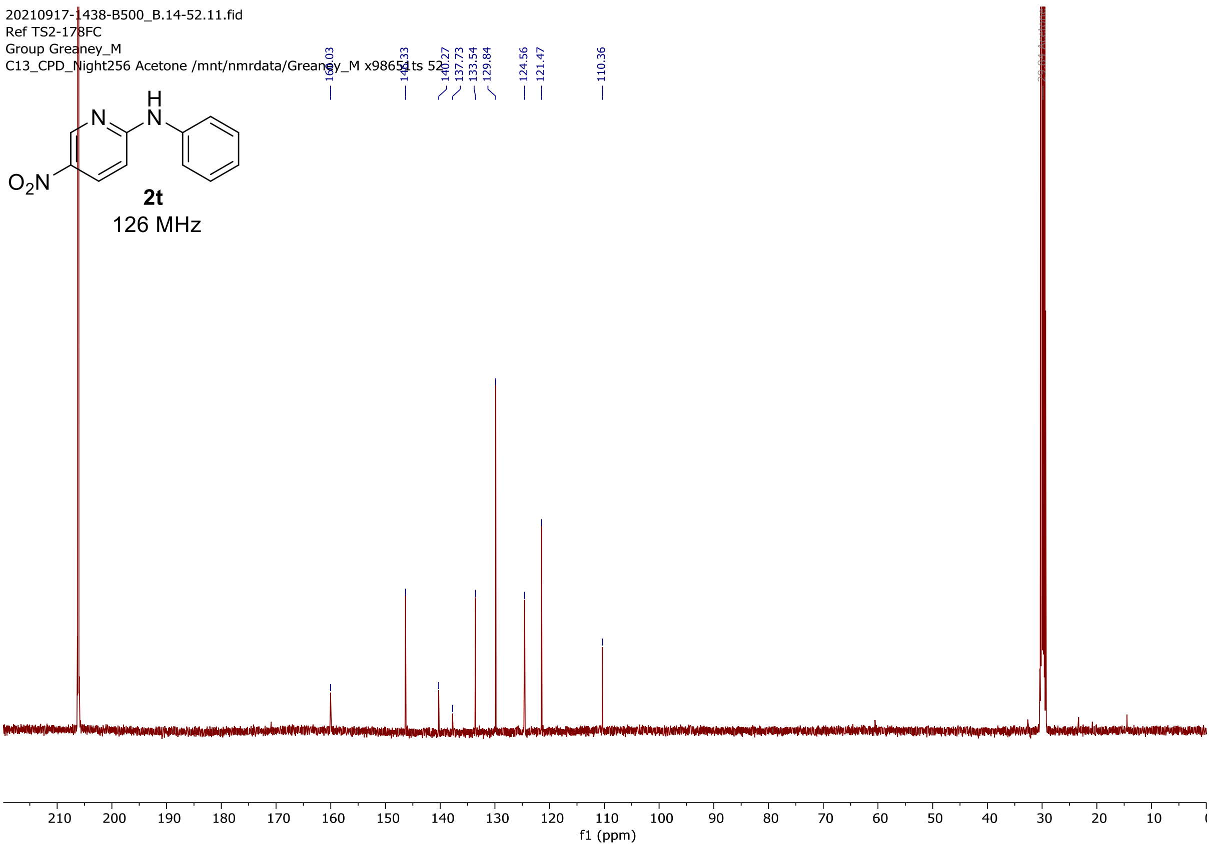
20210917-1438-B500_B.14-52.11.fid
Ref TS2-178FC
Group Greaney_M
C13_CPD_Night256 Acetone /mnt/nmrdata/Greaney_M x98651ts 52
160.03
146.33
140.27
137.73
133.54
129.84
124.56
121.47
110.36
2t
126 MHz
f1 (ppm)

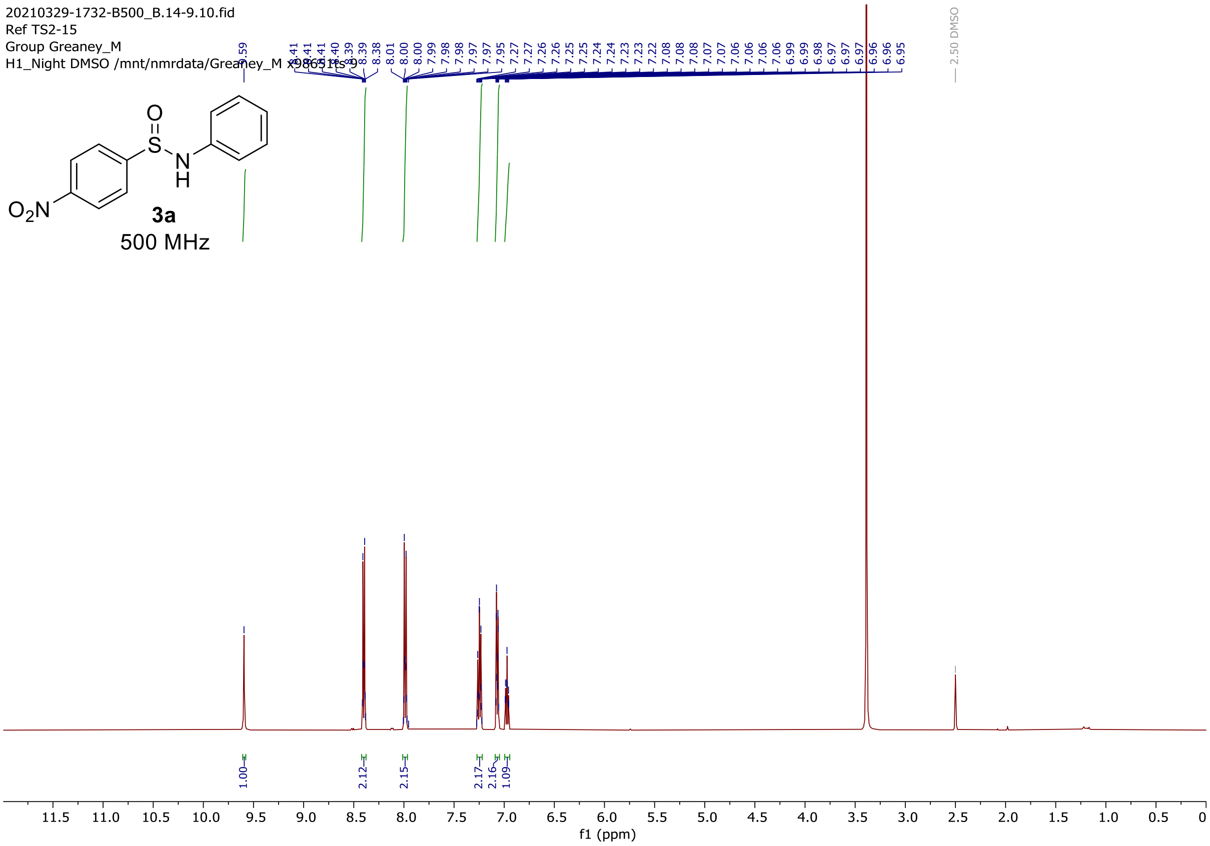
20210329-1732-B500_B.14-9.10.fid
Ref TS2-15
Group Greaney_M
H1_Night DMSO /mnt/nmrdata/Greaney_M x98651ts 9
9.59
8.41
8.41
8.41
8.40
8.39
8.39
8.38
8.01
8.00
8.00
7.99
7.98
7.98
7.97
7.97
7.95
7.27
7.27
7.26
7.25
7.25
7.24
7.24
7.23
7.23
7.22
7.08
7.08
7.08
7.07
7.07
7.06
7.06
7.06
7.06
6.99
6.98
6.97
6.97
6.97
6.96
6.96
6.95
2.50 DMSO
3a
500 MHz
1.00
2.12
2.15
2.17
2.16
1.09
f1 (ppm)

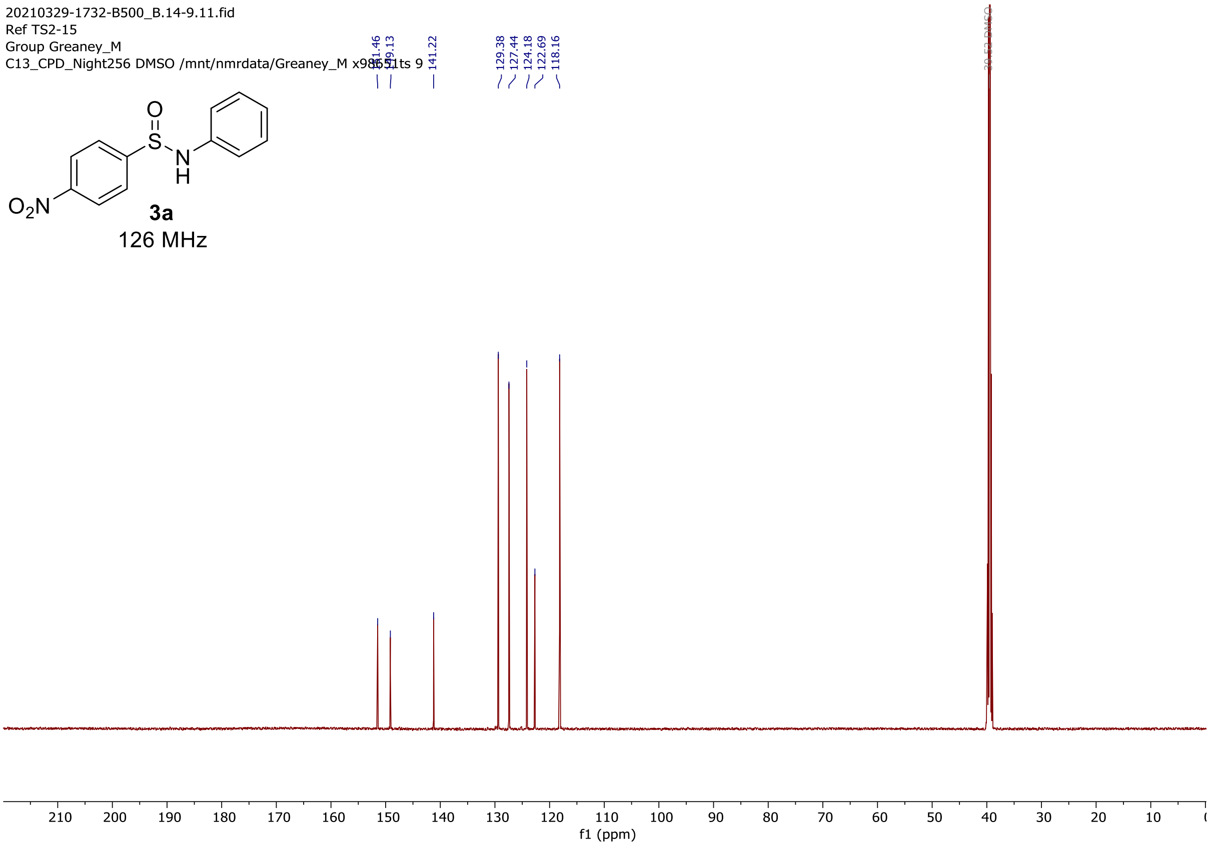
20210329-1732-B500_B.14-9.11.fid
Ref TS2-15
Group Greaney_M
C13_CPD_Night256 DMSO /mnt/nmrdata/Greaney_M x98651ts 9
149.13
141.22
129.38
127.44
124.18
122.69
118.16
3a
126 MHz
f1 (ppm)

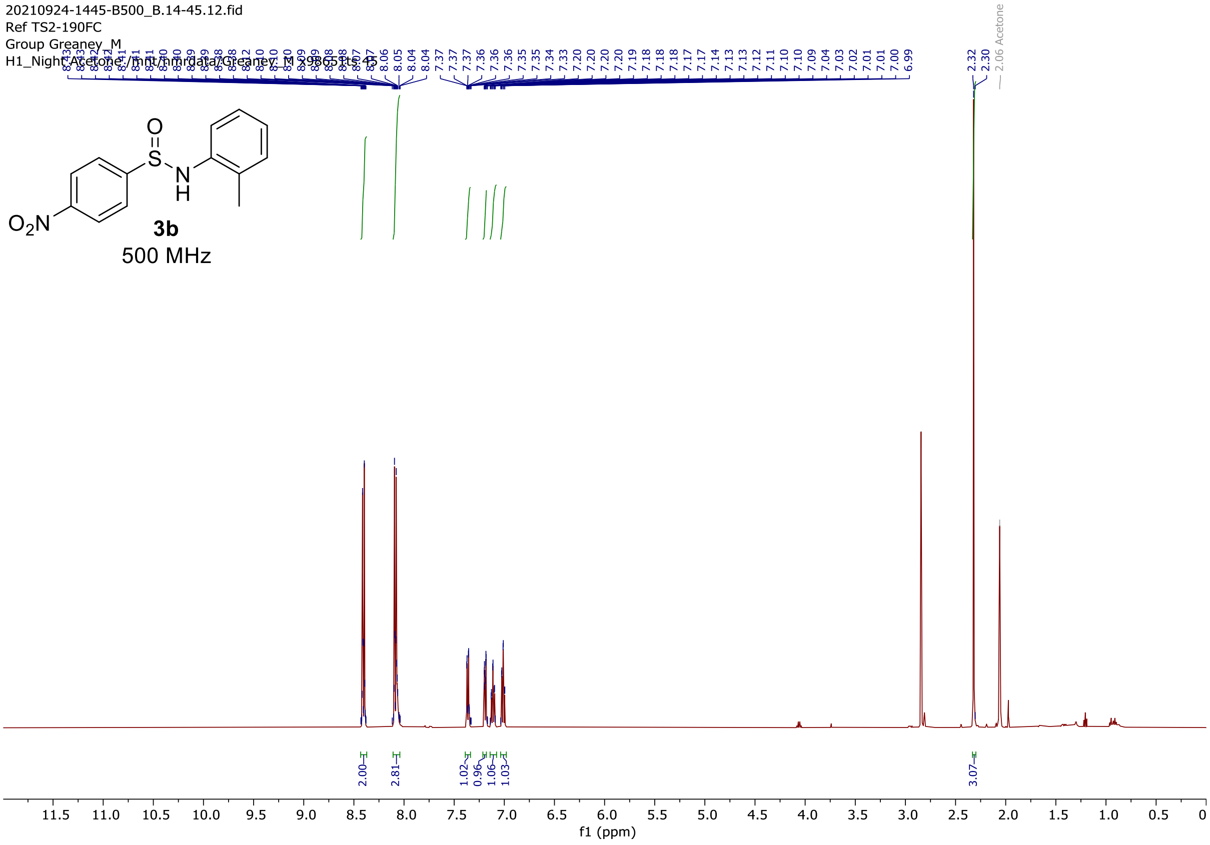
20210924-1445-B500_B.14-45.12.fid
Ref TS2-190FC
Group Greaney_M
8.05
8.04
8.04
7.37
7.37
7.36
7.36
7.35
7.34
7.33
7.20
7.20
7.20
7.19
7.18
7.18
7.18
7.17
7.14
7.13
7.13
7.12
7.11
7.10
7.10
7.09
7.04
7.03
7.02
7.01
7.01
7.00
6.99
2.32
2.30
2.06 Acetone
3b
500 MHz
2.00
2.81
1.02
0.96
1.06
1.03
3.07
f1 (ppm)

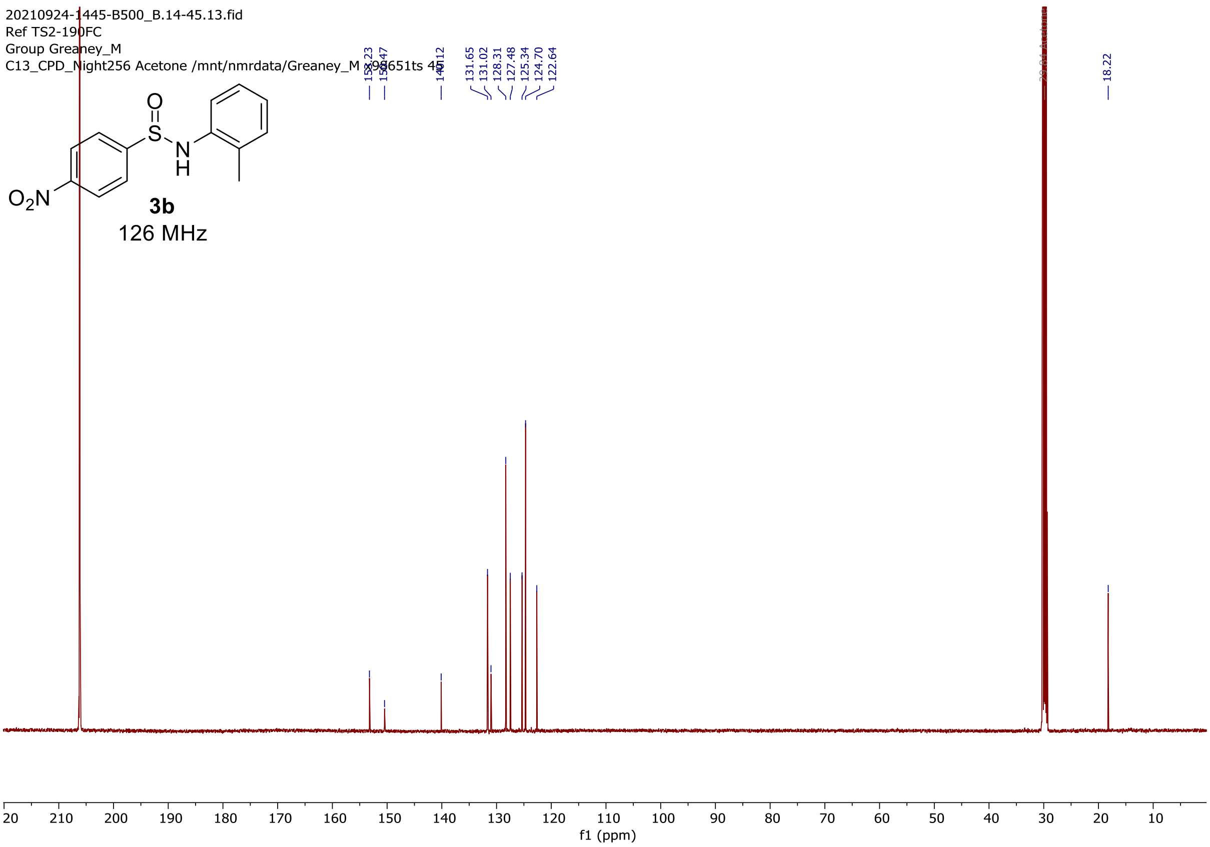
20210924-1445-B500_B.14-45.13.fid
Ref TS2-190FC
Group Greaney_M
C13_CPD_Night256 Acetone /mnt/nmrdata/Greaney_M x98651ts 45
131.65
131.02
128.31
127.48
125.34
124.70
122.64
18.22
O
S
N
H
O2N
3b
126 MHz
f1 (ppm)

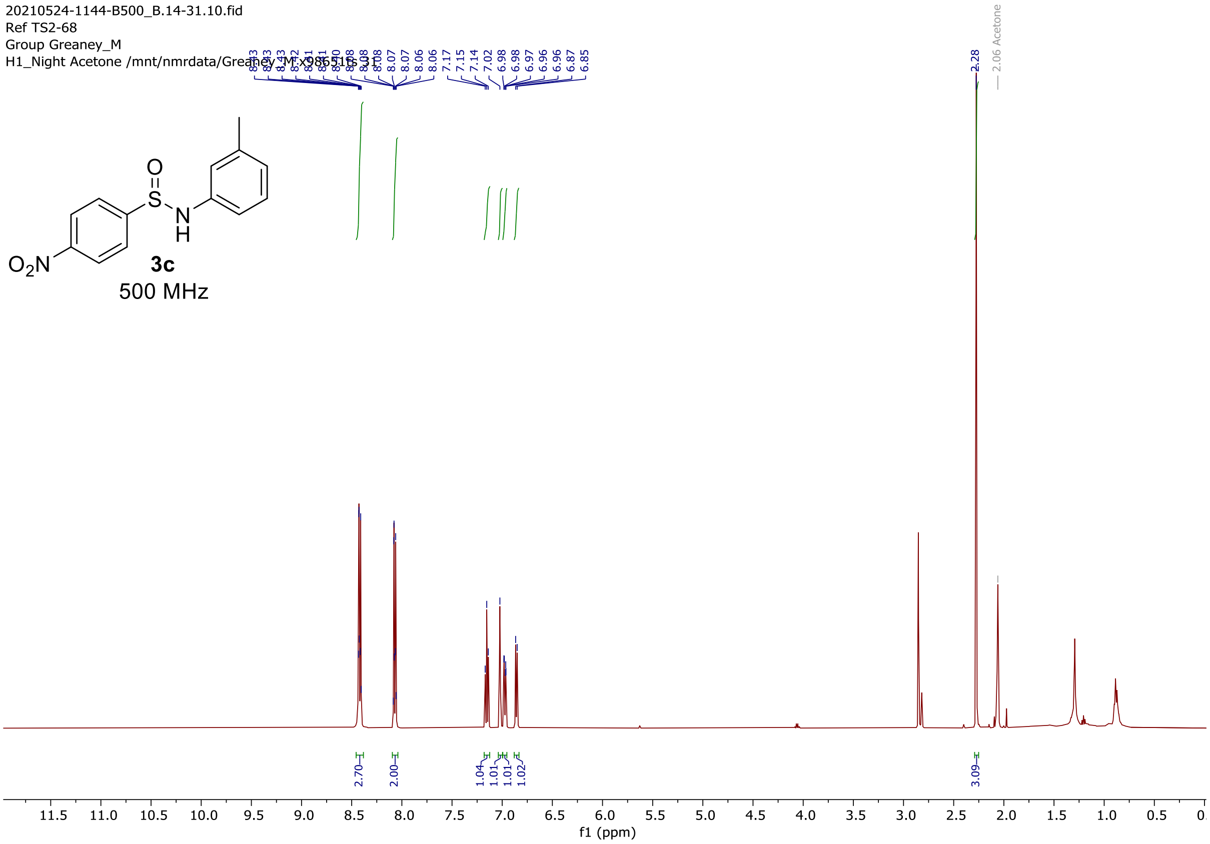
20210524-1144-B500_B.14-31.10.fid
Ref TS2-68
Group Greaney_M
8.08
8.07
8.07
8.06
8.06
7.17
7.15
7.14
7.02
6.98
6.98
6.97
6.96
6.96
6.87
6.85
2.28
2.06 Acetone
O
S
N
H
O2N
3c
500 MHz
2.70
2.00
1.04
1.01
1.01
1.02
3.09
f1 (ppm)

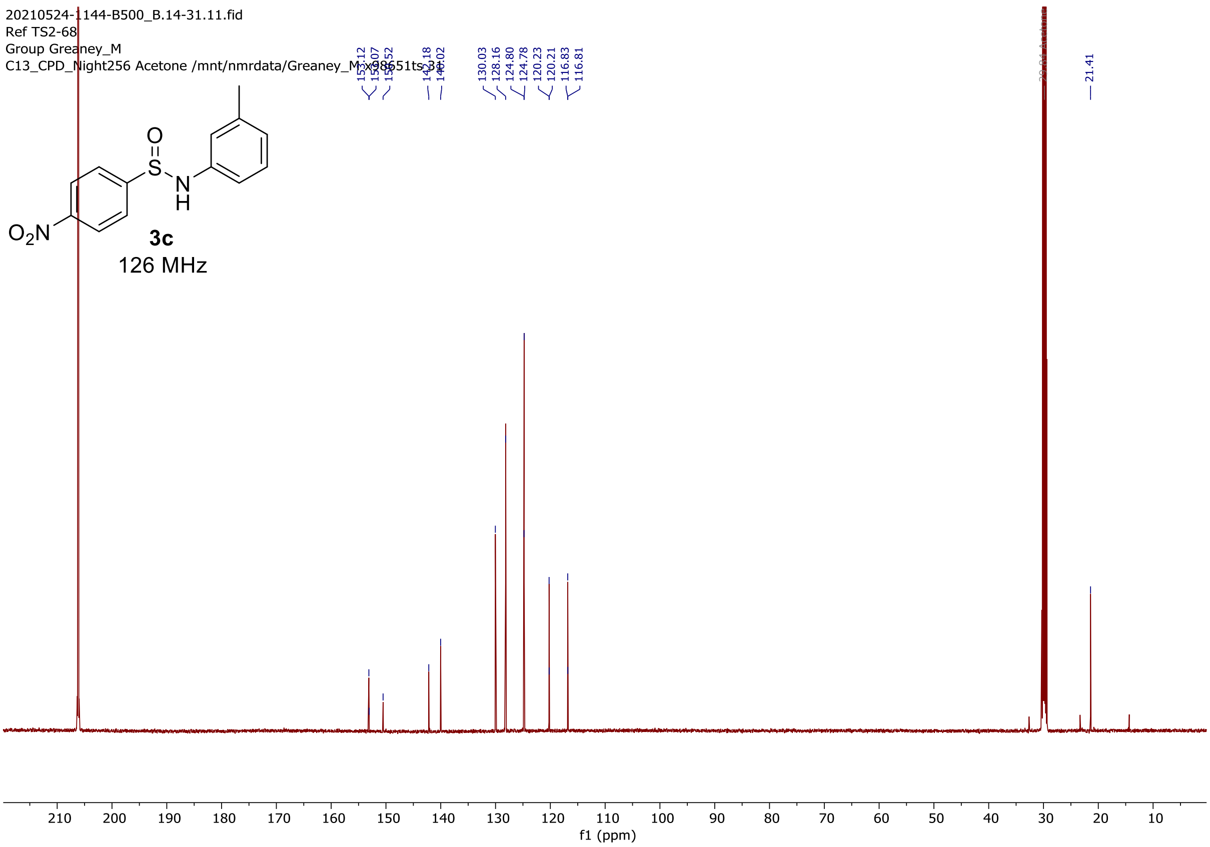
20210524-1144-B500_B.14-31.11.fid
Ref TS2-68
Group Greaney_M
C13_CPD_Night256 Acetone /mnt/nmrdata/Greaney_M
130.03
128.16
124.80
124.78
120.23
120.21
116.83
116.81
21.41
3c
126 MHz
f1 (ppm)

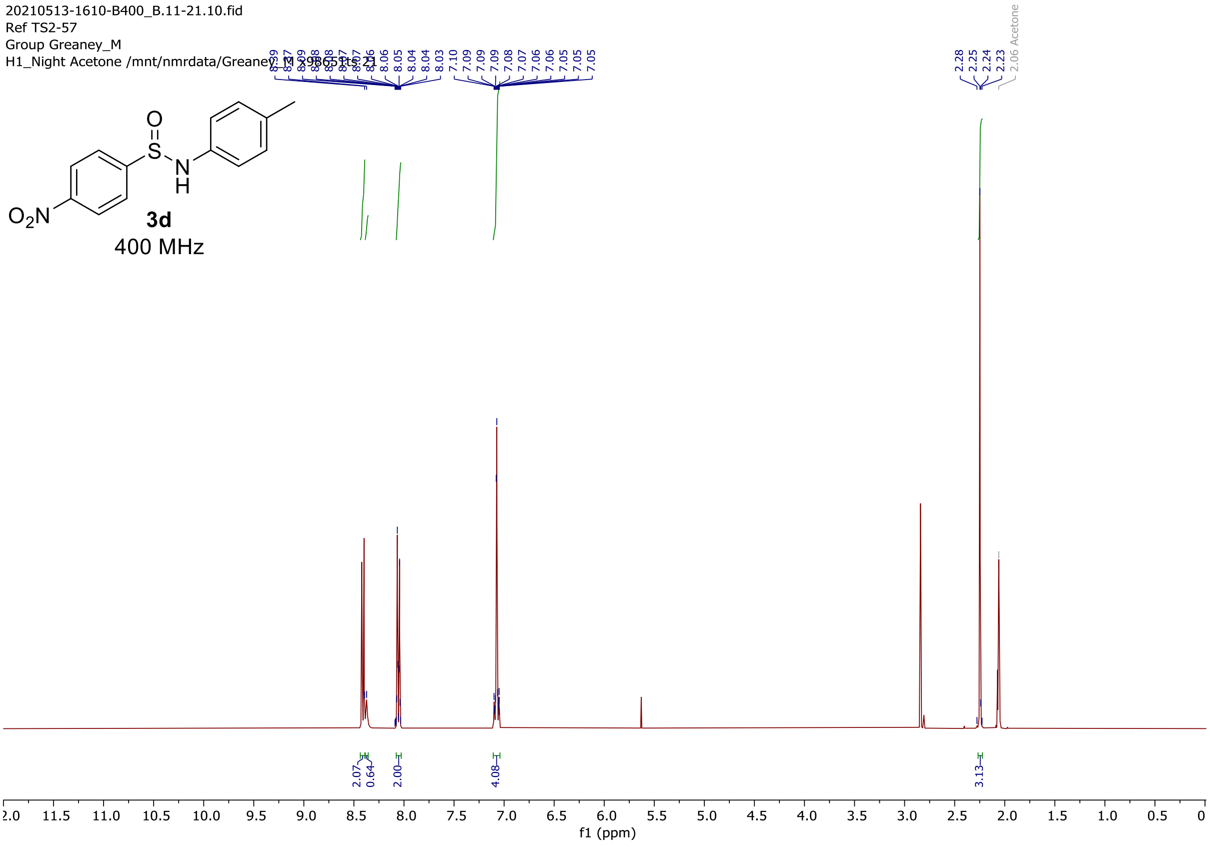
20210513-1610-B400_B.11-21.10.fid
Ref TS2-57
Group Greaney_M
H1_Night Acetone /mnt/nmrdata/Greaney_M
2.28
2.25
2.24
2.23
2.06 Acetone
3d
400 MHz
2.07
0.64
2.00
4.08
3.13
f1 (ppm)

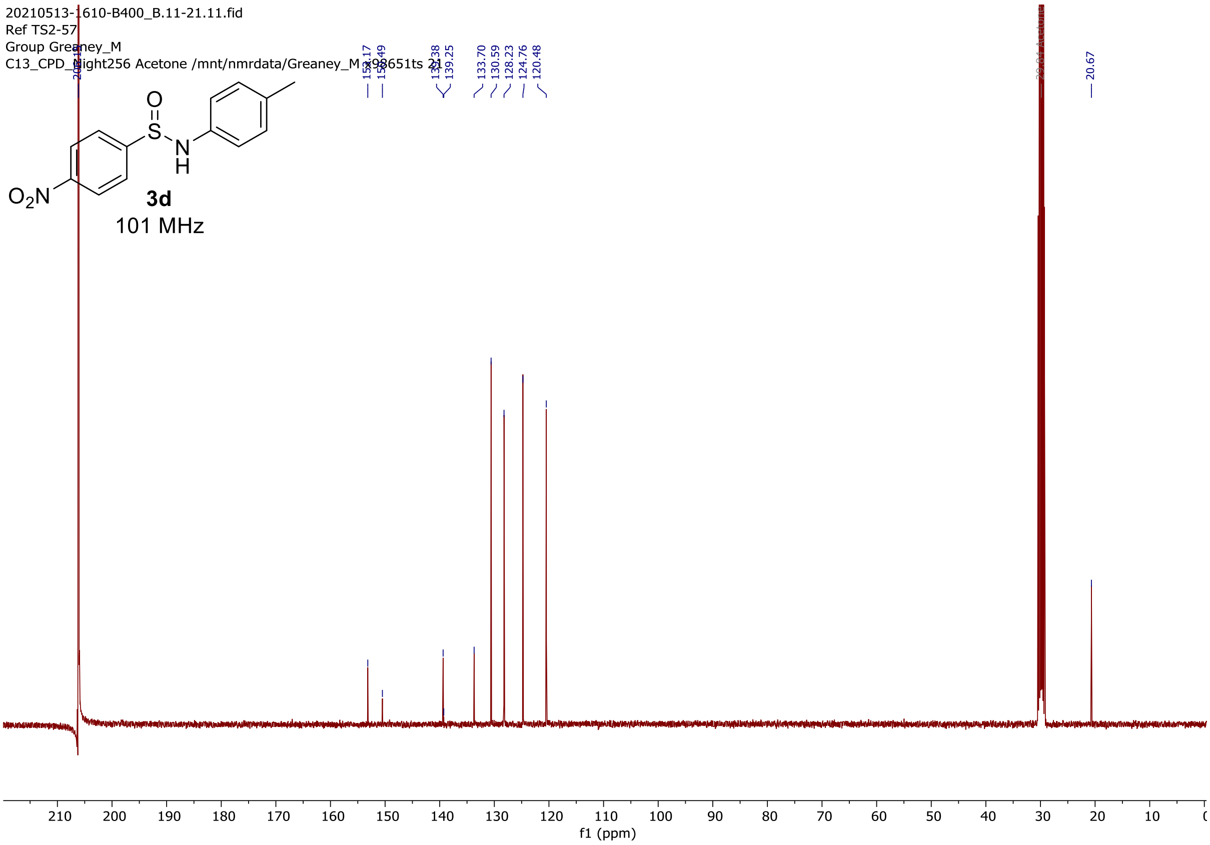
20210513-1610-B400_B.11-21.11.fid
Ref TS2-57
Group Greaney_M
C13_CPD_night256 Acetone /mnt/nmrdata/Greaney_M x98651ts 21
133.70
130.59
128.23
124.76
120.48
20.67
O
S
N
H
O2N
3d
101 MHz
f1 (ppm)

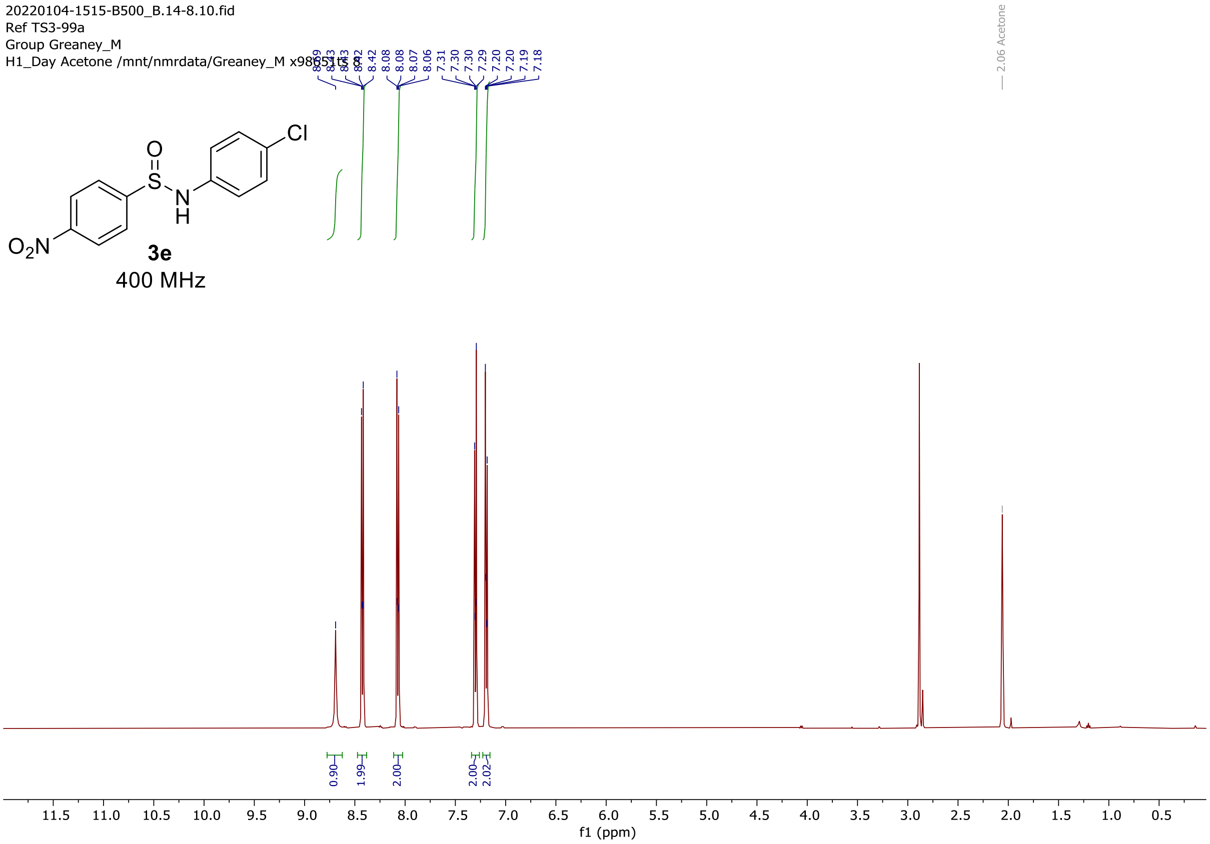
20220104-1515-B500_B.14-8.10.fid
Ref TS3-99a
Group Greaney_M
H1_Day Acetone /mnt/nmrdata/Greaney_M x98651ts 8
8.42
8.08
8.08
8.07
8.06
7.31
7.30
7.30
7.29
7.20
7.20
7.19
7.18
2.06 Acetone
O
S
N
H
Cl
O2N
3e
400 MHz
0.90
1.99
2.00
2.00
2.02
f1 (ppm)

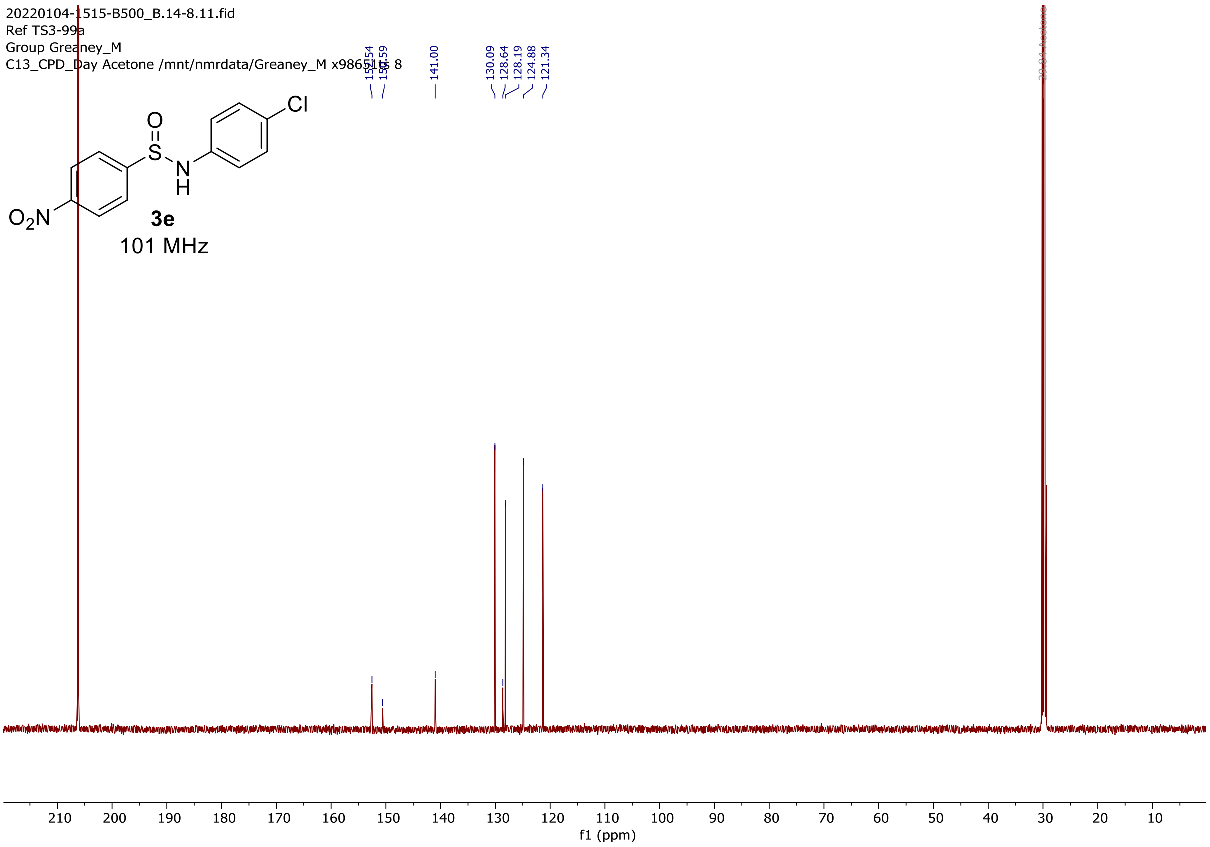

20220104-1515-B500_B.14-8.11.fid
Ref TS3-99a
Group Greaney_M
C13_CPD_Day Acetone /mnt/nmrdata/Greaney_M
141.00
130.09
128.64
128.19
124.88
121.34
Cl
O
S
N
H
O2N
3e
101 MHz
f1 (ppm)

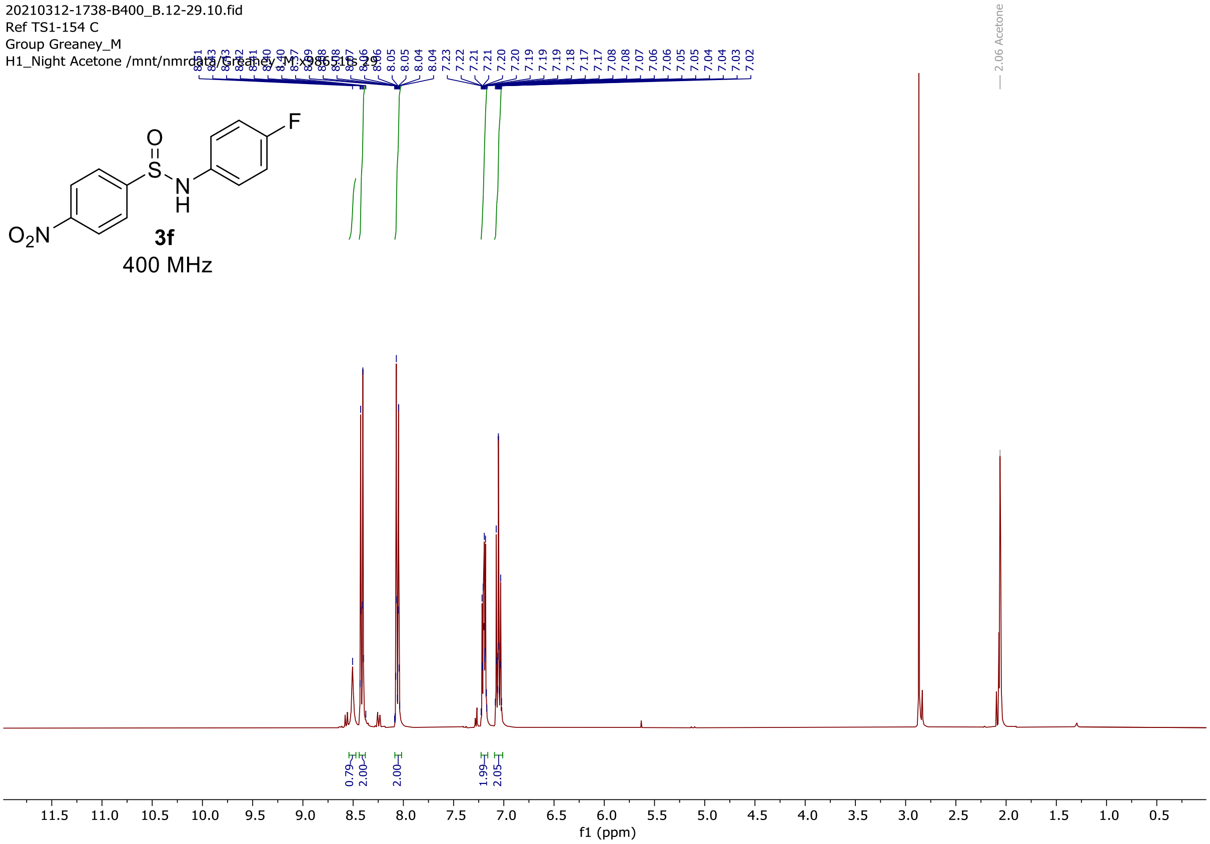

20210312-1738-B400_B.12-29.10.fid
Ref TS1-154 C
Group Greaney_M
H1_Night Acetone /mnt/nmrdata/Greaney_M
8.06
8.05
8.05
8.04
8.04
7.23
7.22
7.21
7.21
7.20
7.19
7.19
7.18
7.17
7.17
7.08
7.07
7.06
7.06
7.05
7.05
7.04
7.04
7.03
7.02
2.06 Acetone
F
O
S
N
H
O2N
3f
400 MHz
0.79
2.00
2.00
1.99
2.05
f1 (ppm)

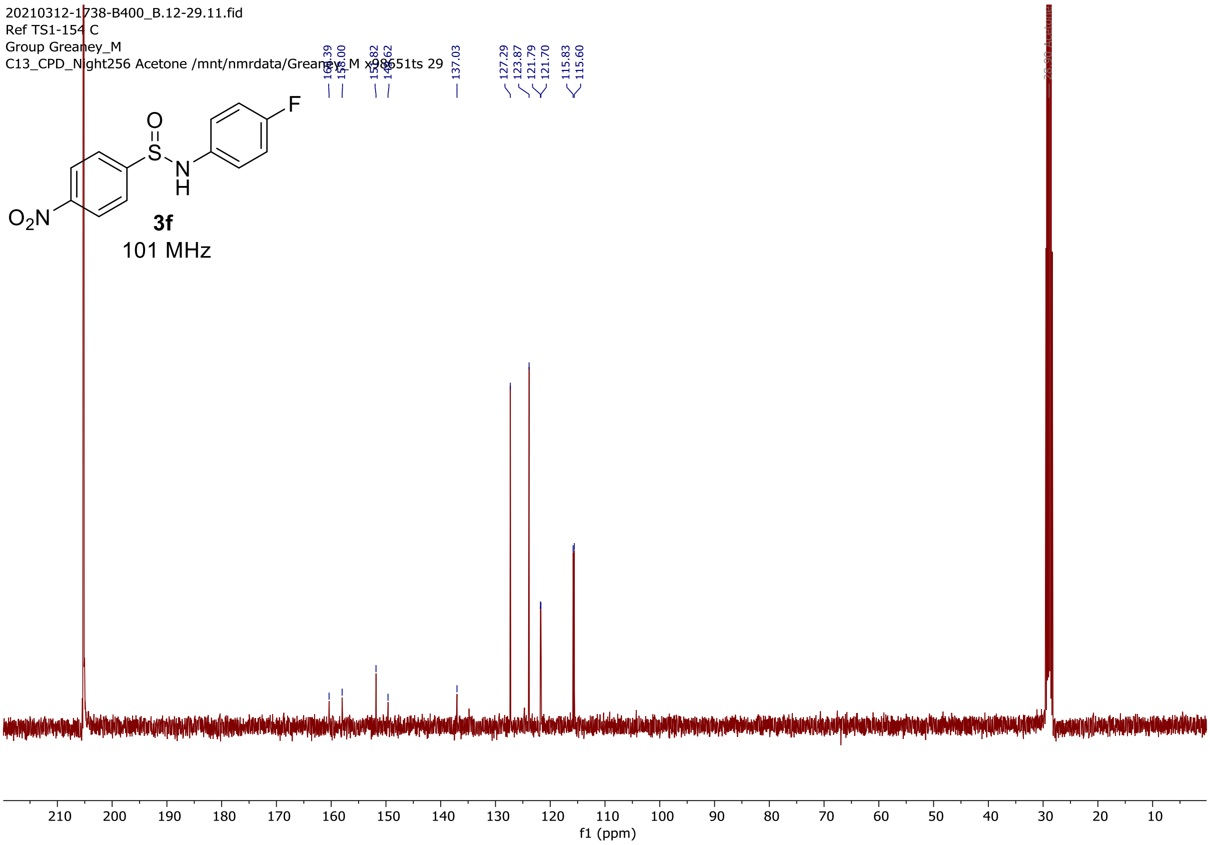
20210312-1738-B400_B.12-29.11.fid
Ref TS1-154 C
Group Greaney_M
C13_CPD_Night256 Acetone /mnt/nmrdata/Greaney_M x98651ts 29
137.03
127.29
123.87
121.79
121.70
115.83
115.60
3f
101 MHz
f1 (ppm)

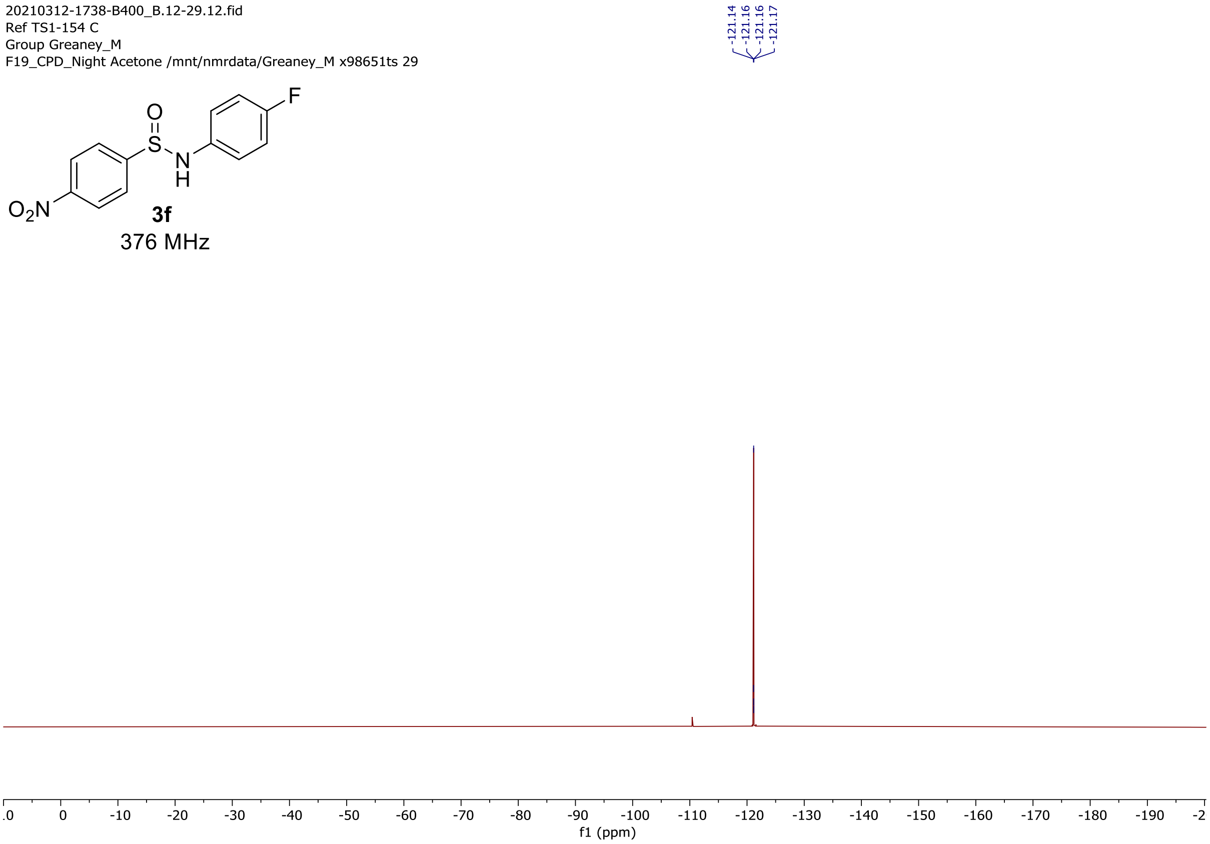
20210312-1738-B400_B.12-29.12.fid
Ref TS1-154 C
Group Greaney_M
F19_CPD_Night Acetone /mnt/nmrdata/Greaney_M x98651ts 29
-121.14
-121.16
-121.16
-121.17
3f
376 MHz
f1 (ppm)

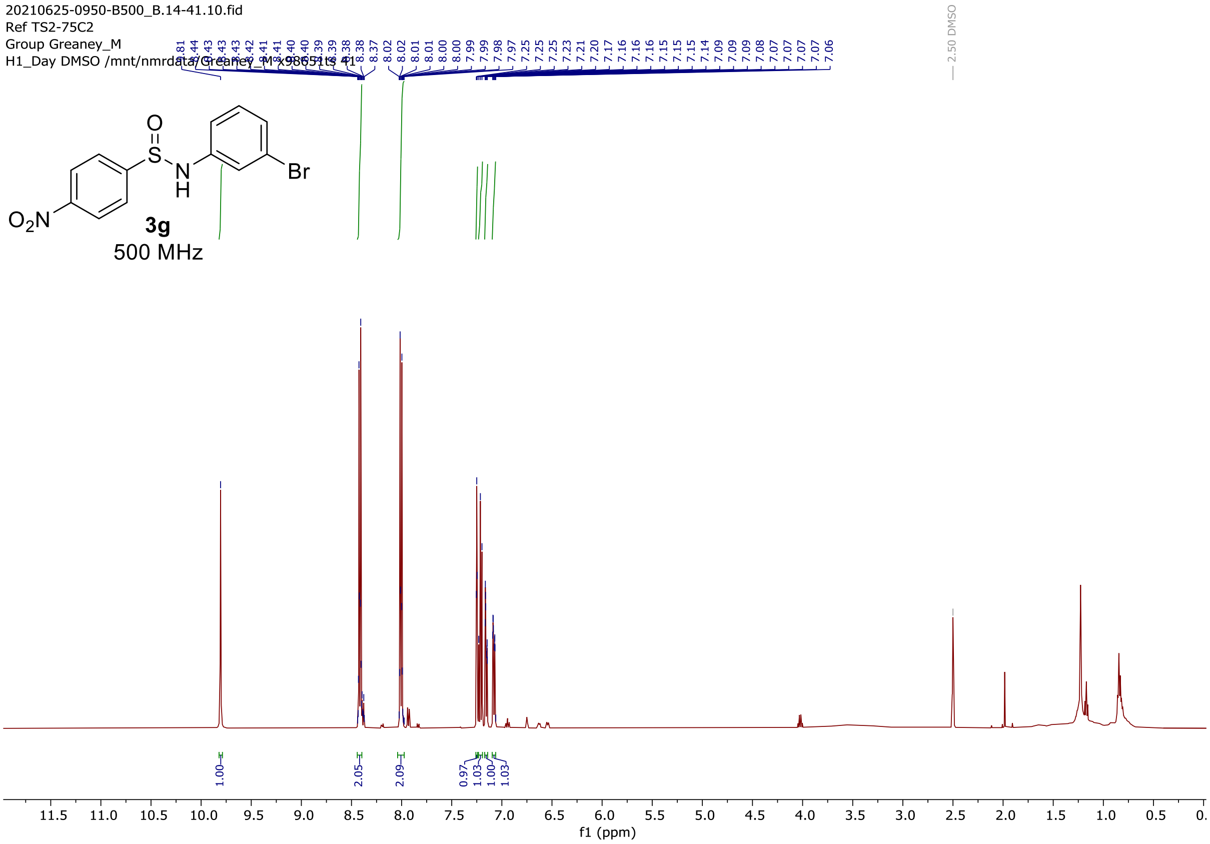
20210625-0950-B500_B.14-41.10.fid
Ref TS2-75C2
Group Greaney_M
H1_Day DMSO /mnt/nmrdata/Greaney_M x98651ts 41
8.44 8.43 8.43 8.43 8.42 8.41 8.41 8.40 8.40 8.39 8.39 8.38 8.38 8.37 8.02 8.02 8.01 8.01 8.00 8.00 7.99 7.99 7.98 7.97 7.25 7.25 7.25 7.23 7.21 7.20 7.17 7.16 7.16 7.15 7.15 7.15 7.14 7.09 7.09 7.09 7.08 7.07 7.07 7.07 7.06
2.50 DMSO
O
S
N
H
Br
O_2N
3g
500 MHz
1.00
2.05
2.09
0.97
1.03
1.00
1.03
f1 (ppm)

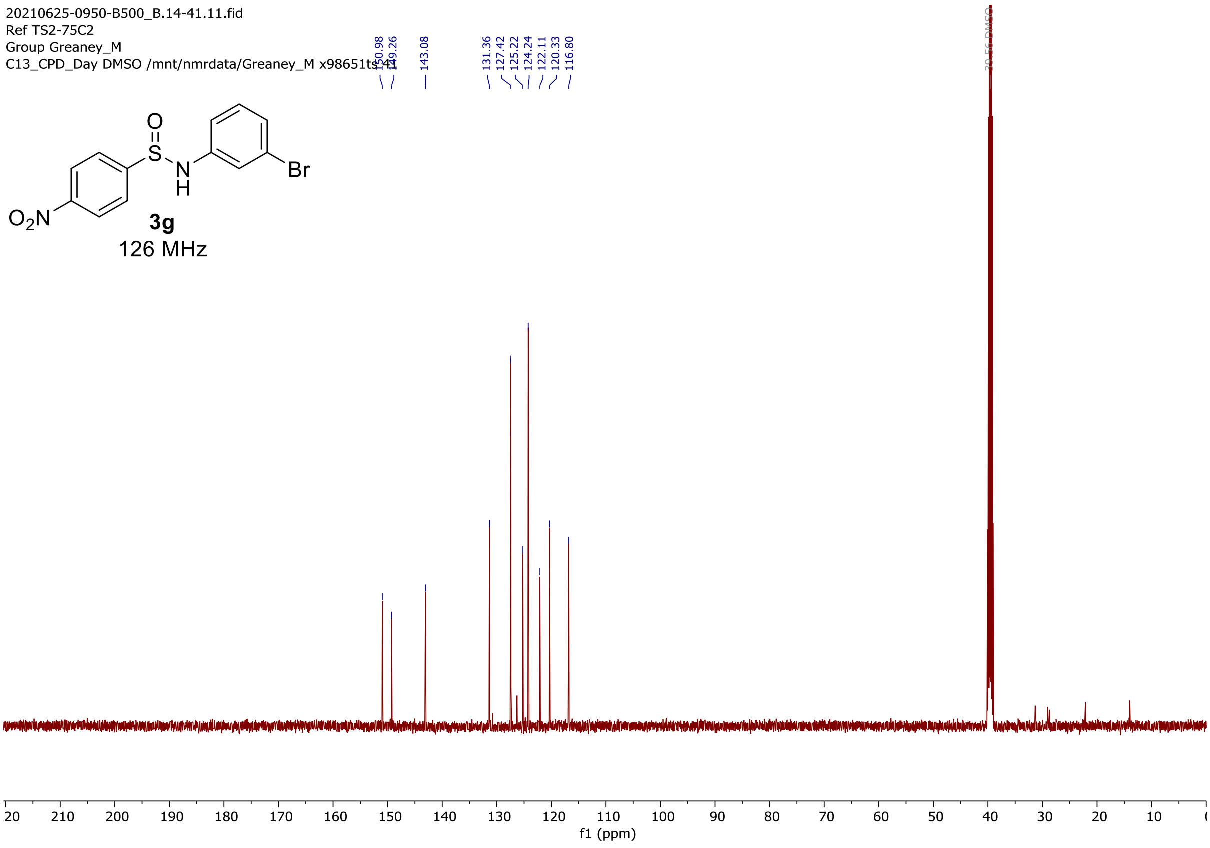
20210625-0950-B500_B.14-41.11.fid
Ref TS2-75C2
Group Greaney_M
C13_CPD_Day DMSO /mnt/nmrdata/Greaney_M x98651ts 41
150.98
149.26
143.08
131.36
127.42
125.22
124.24
122.11
120.33
116.80
O
S
N
H
Br
O_2N
3g
126 MHz
f1 (ppm)

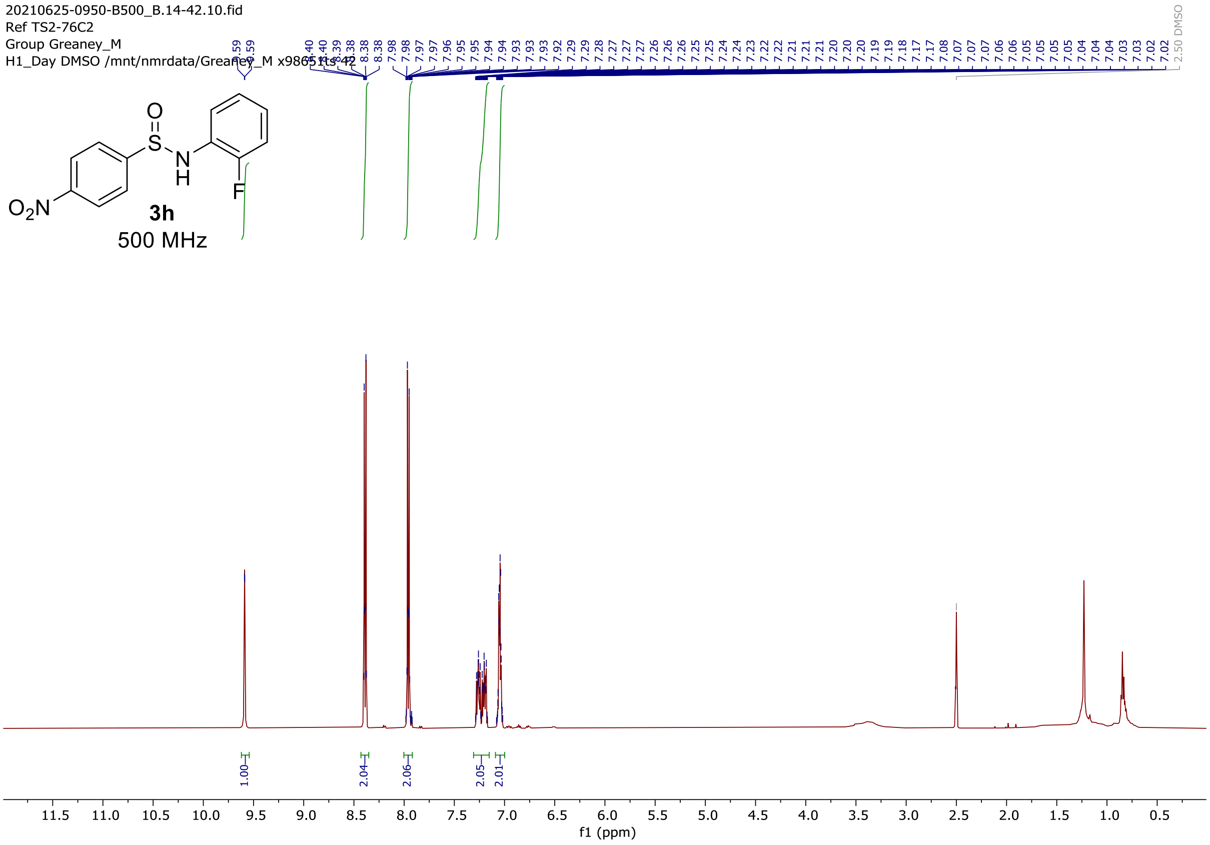
20210625-0950-B500_B.14-42.10.fid
Ref TS2-76C2
Group Greaney_M
H1_Day DMSO /mnt/nmrdata/Greaney_M x98651ts 42
3h
500 MHz
f1 (ppm)

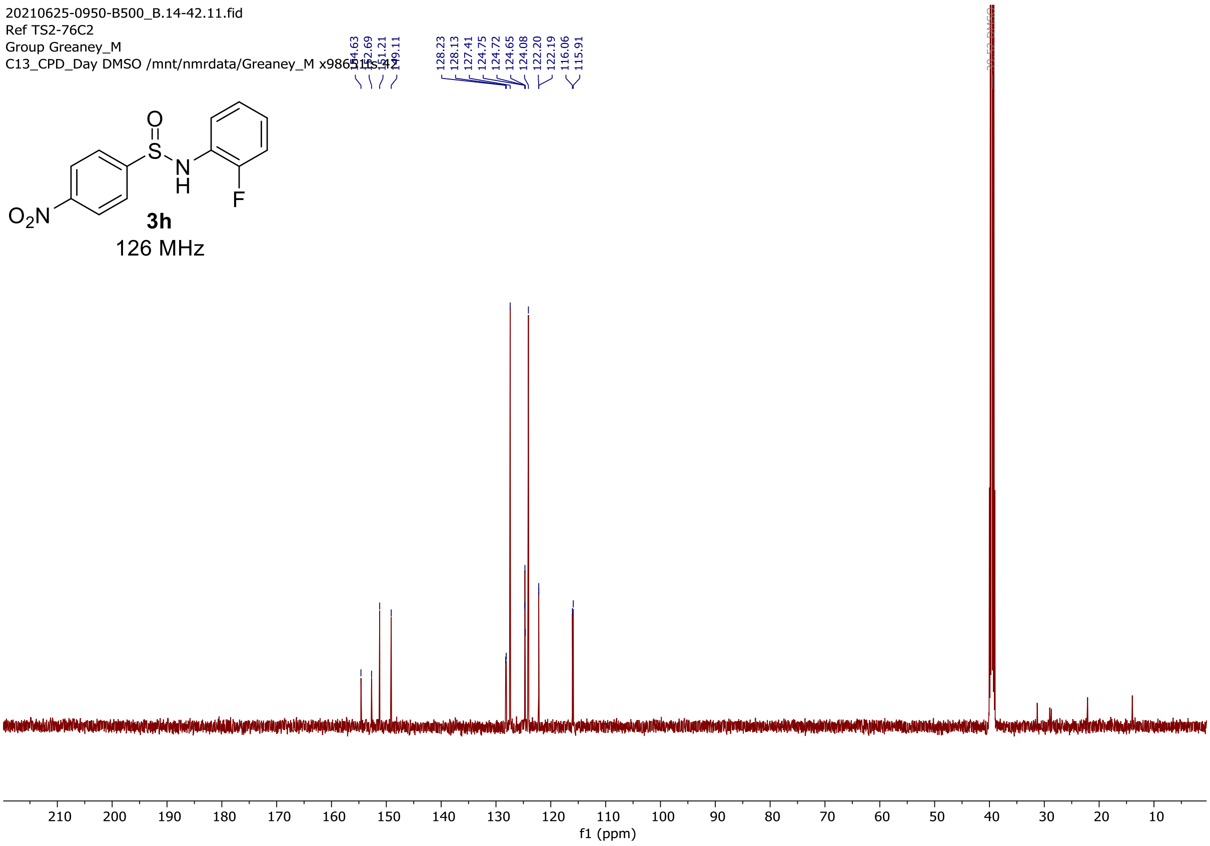
20210625-0950-B500_B.14-42.11.fid
Ref TS2-76C2
Group Greaney_M
C13_CPD_Day DMSO /mnt/nmrdata/Greaney_M x98651ts 42
3h
126 MHz
f1 (ppm)

20210625-0950-B500_B.14-42.12.fid
Ref TS2-76C2
Group Greaney_M
F19_NoCPD_Day DMSO /mnt/nmrdata/Greaney_M x98651ts 42

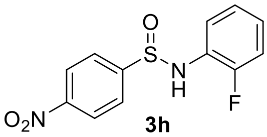

3h
376 MHz

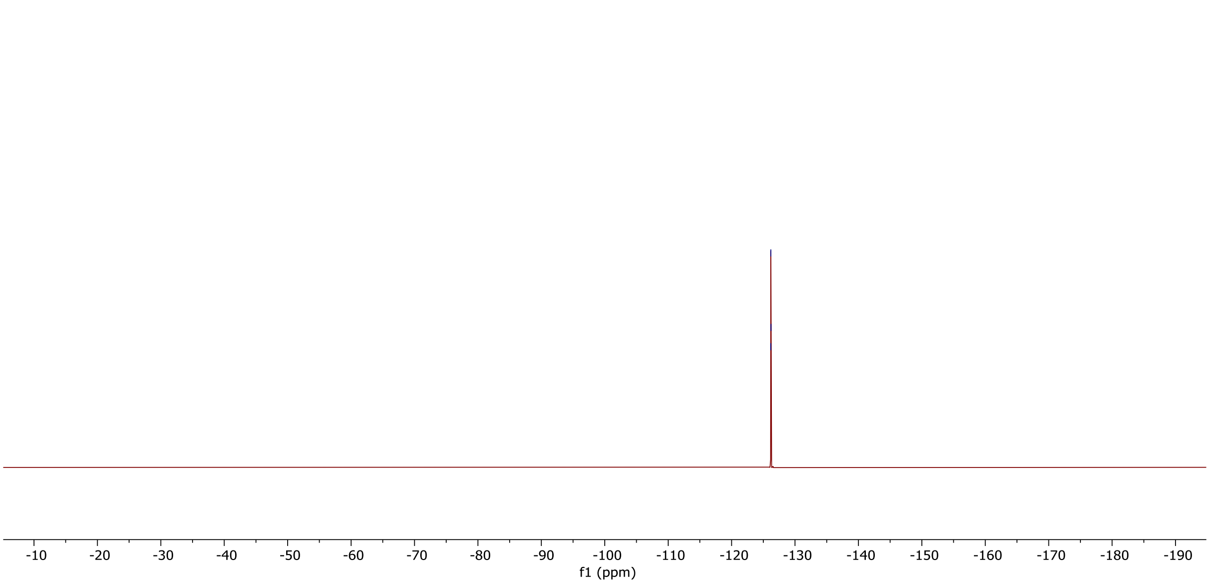

20210510-1614-B400_B.11-42.10.fid
Ref TS2-51C
Group Greaney_M
H1_Night Acetone /mnt/nmrdata/Greaney_M x98651ts 42

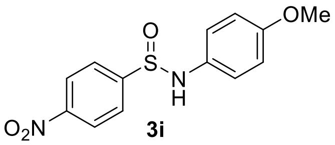

3i
400 MHz

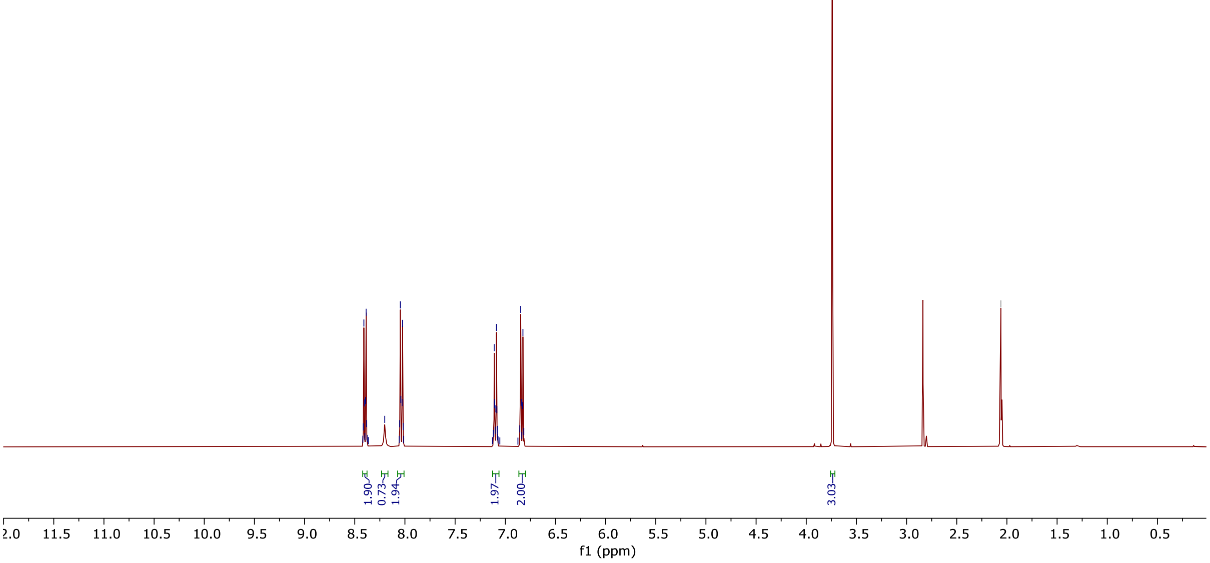

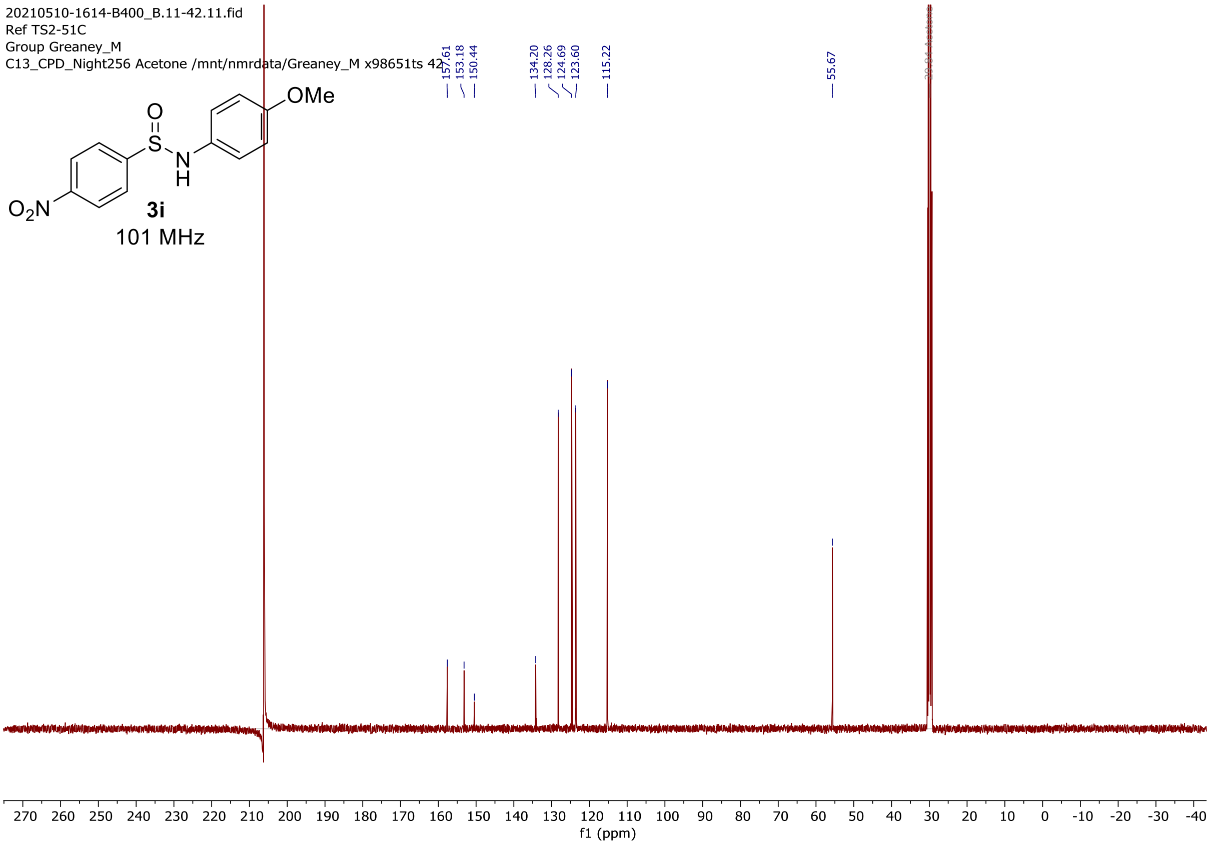

20210510-1614-B400_B.11-42.11.fid
Ref TS2-51C
Group Greaney_M
C13_CPD_Night256 Acetone /mnt/nmrdata/Greaney_M x98651ts 42
157.61
153.18
150.44
134.20
128.26
124.69
123.60
115.22
55.67
OMe
O
S
N
H
O_2N
3i
101 MHz
f1 (ppm)

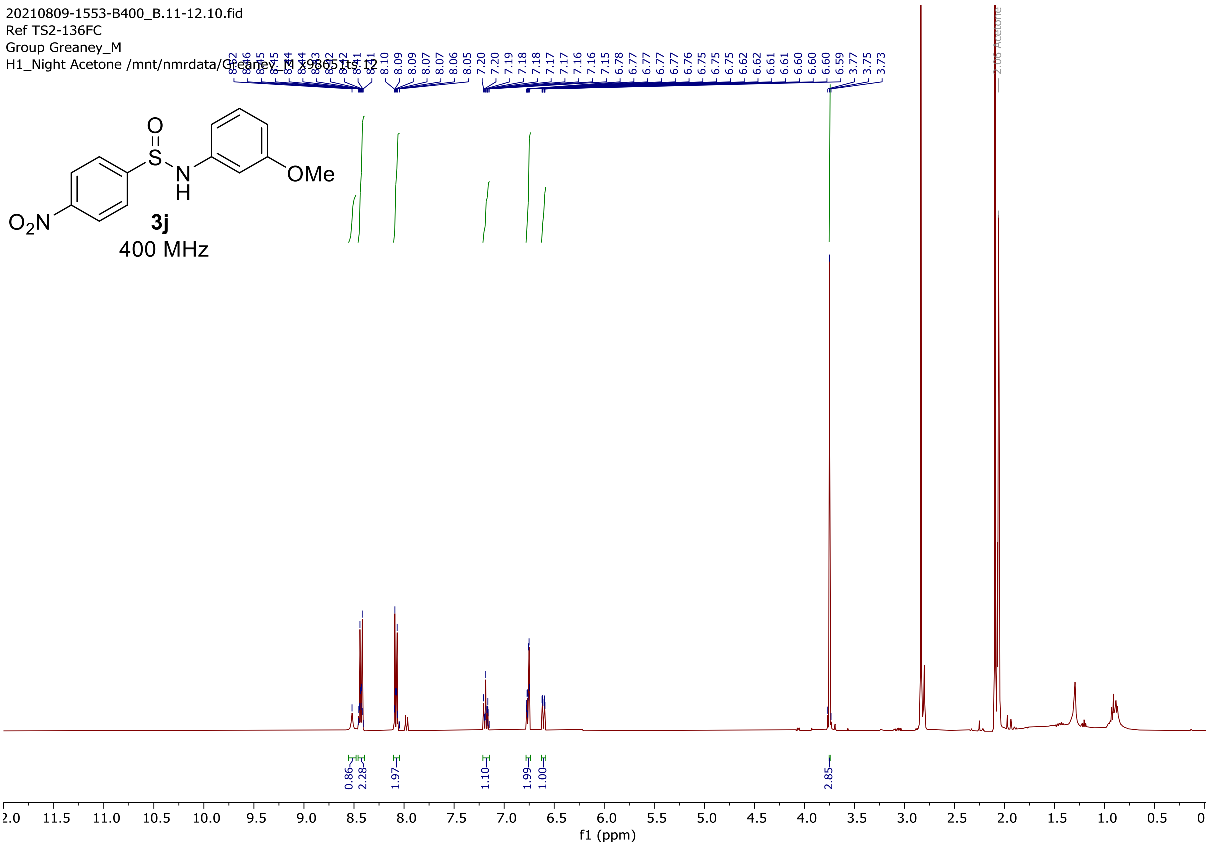

20210809-1553-B400_B.11-12.10.fid
Ref TS2-136FC
Group Greaney_M
H1_Night Acetone /mnt/nmrdata/Greaney_M x98651ts 12
O
S
N
H
OMe
O_2N
3j
400 MHz
0.86
2.28
1.97
1.10
1.99
1.00
2.85
f1 (ppm)

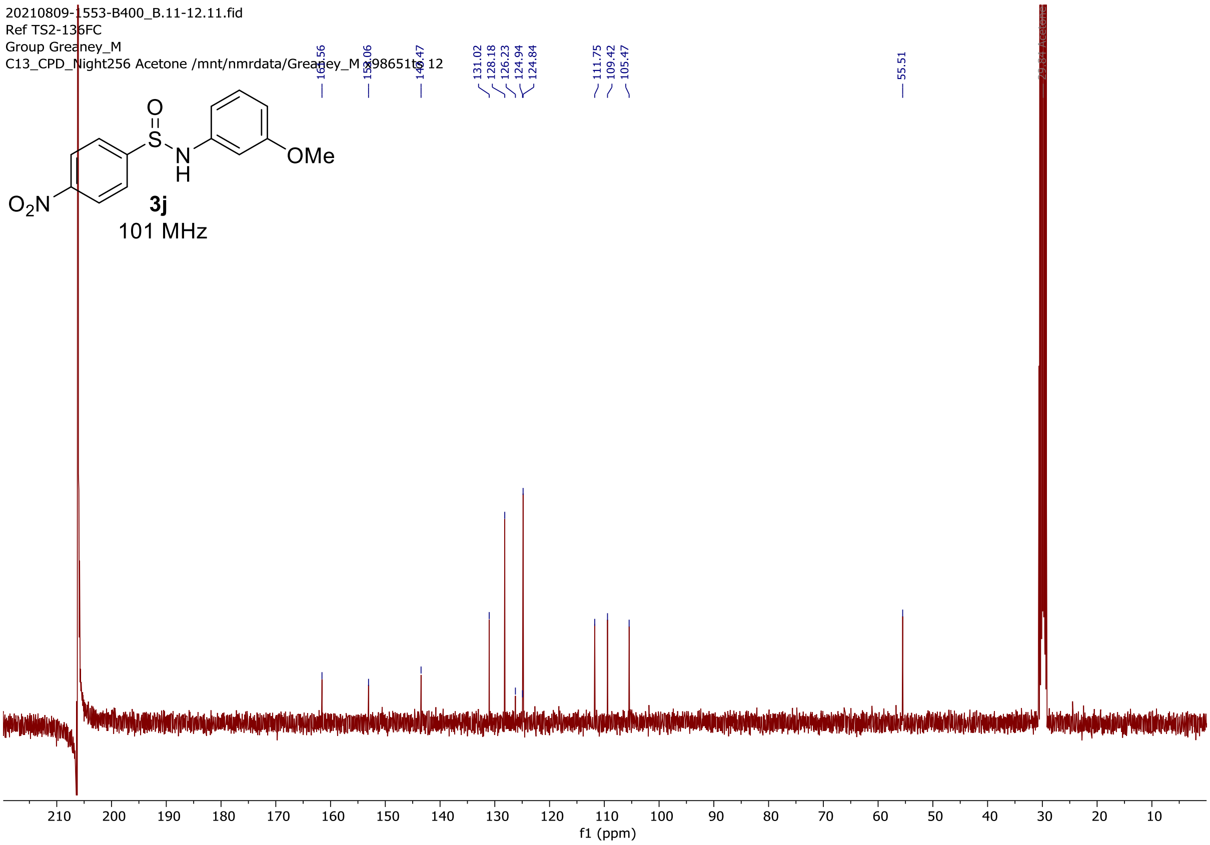
20210809-1553-B400_B.11-12.11.fid
Ref TS2-136FC
Group Greaney_M
C13_CPD_Night256 Acetone /mnt/nmrdata/Greaney_M x98651ts 12
131.02
128.18
126.23
124.94
124.84
111.75
109.42
105.47
55.51
O
S
N
H
OMe
O_2N
3j
101 MHz
f1 (ppm)

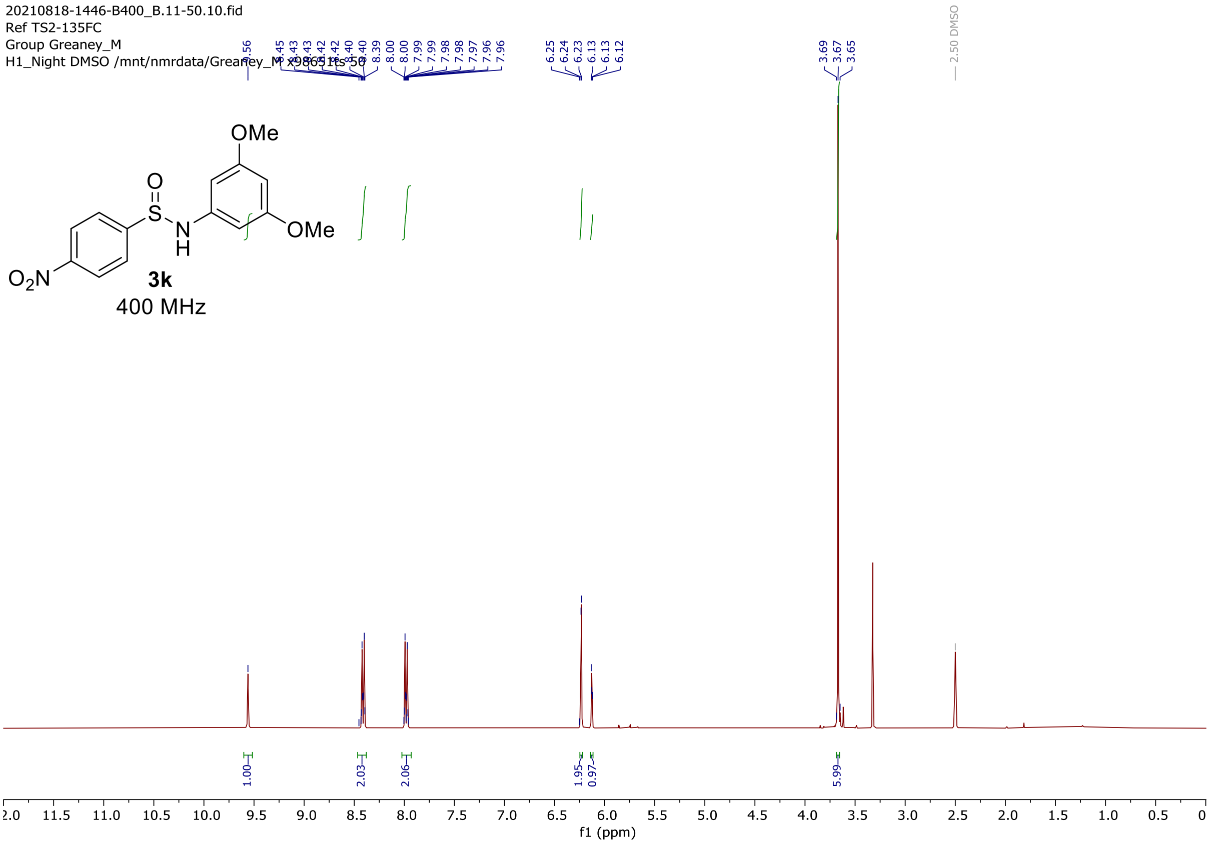
20210818-1446-B400_B.11-50.10.fid
Ref TS2-135FC
Group Greaney_M
H1_Night DMSO /mnt/nmrdata/Greaney_M x98651ts 50
9.56
8.45
8.43
8.43
8.42
8.40
8.40
8.39
8.00
7.99
7.99
7.98
7.98
7.97
7.96
7.96
6.25
6.24
6.23
6.13
6.13
6.12
3.69
3.67
3.65
2.50 DMSO
OMe
O
S
N
H
OMe
O_2N
3k
400 MHz
1.00
2.03
2.06
1.95
0.97
5.99
f1 (ppm)

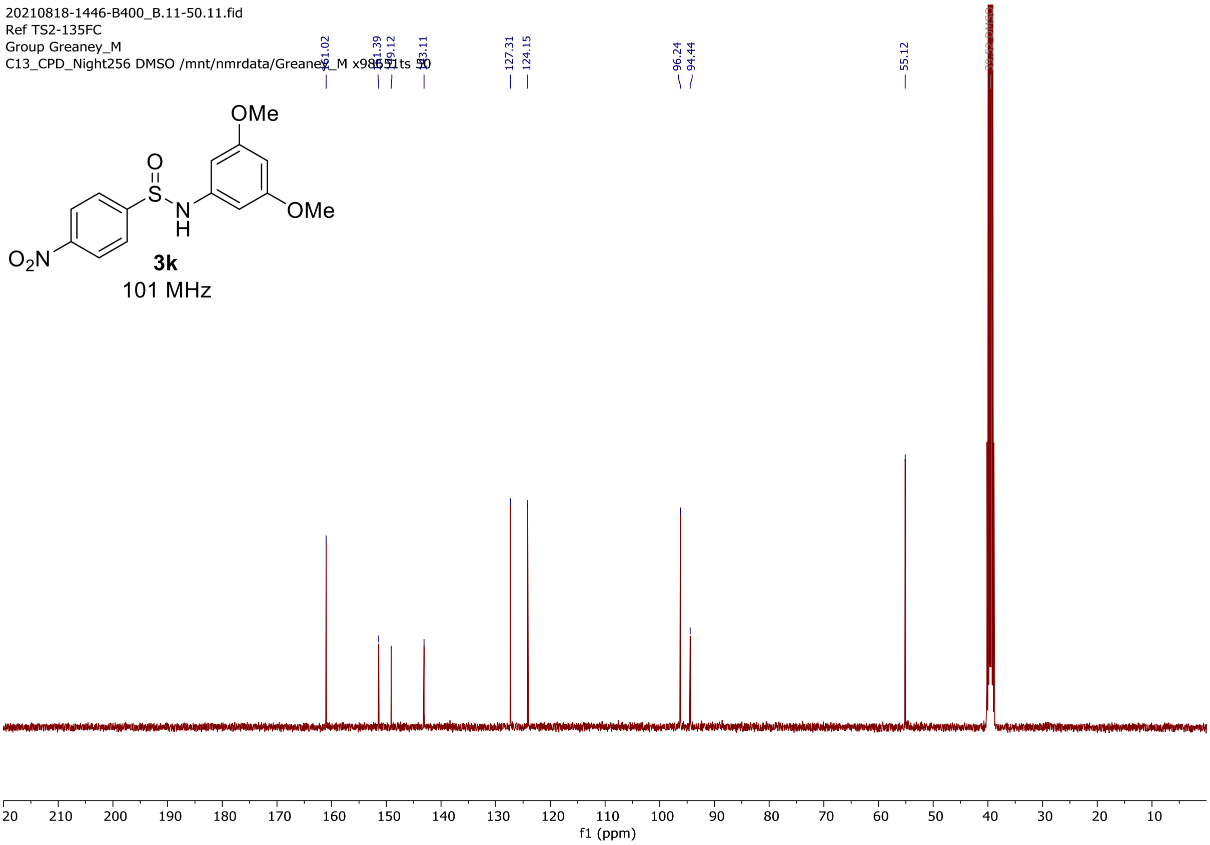
20210818-1446-B400_B.11-50.11.fid
Ref TS2-135FC
Group Greaney_M
C13_CPD_Night256 DMSO /mnt/nmrdata/Greaney_M x98651ts 50
3k
101 MHz
f1 (ppm)

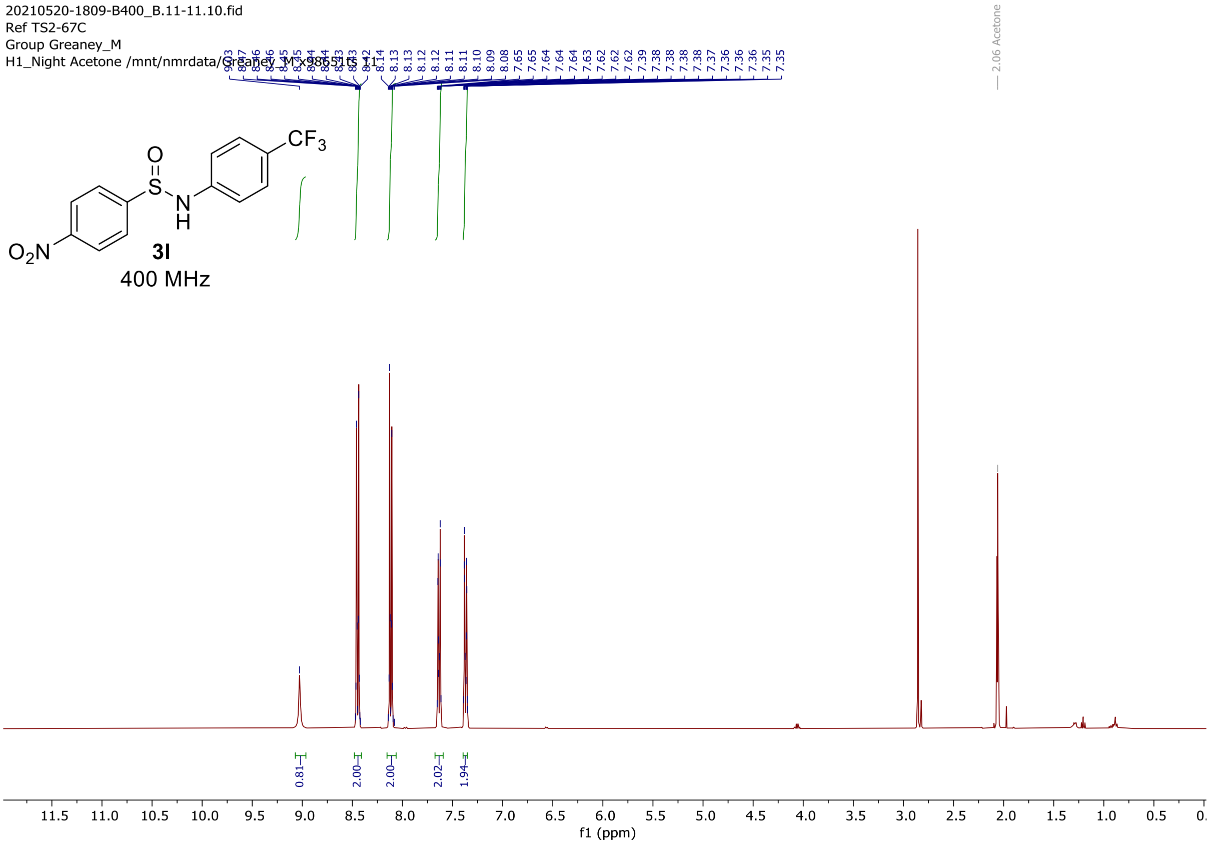
20210520-1809-B400_B.11-11.10.fid
Ref TS2-67C
Group Greaney_M
H1_Night Acetone /mnt/nmrdata/Greaney_M x98651ts 11
2.06 Acetone
3l
400 MHz
f1 (ppm)

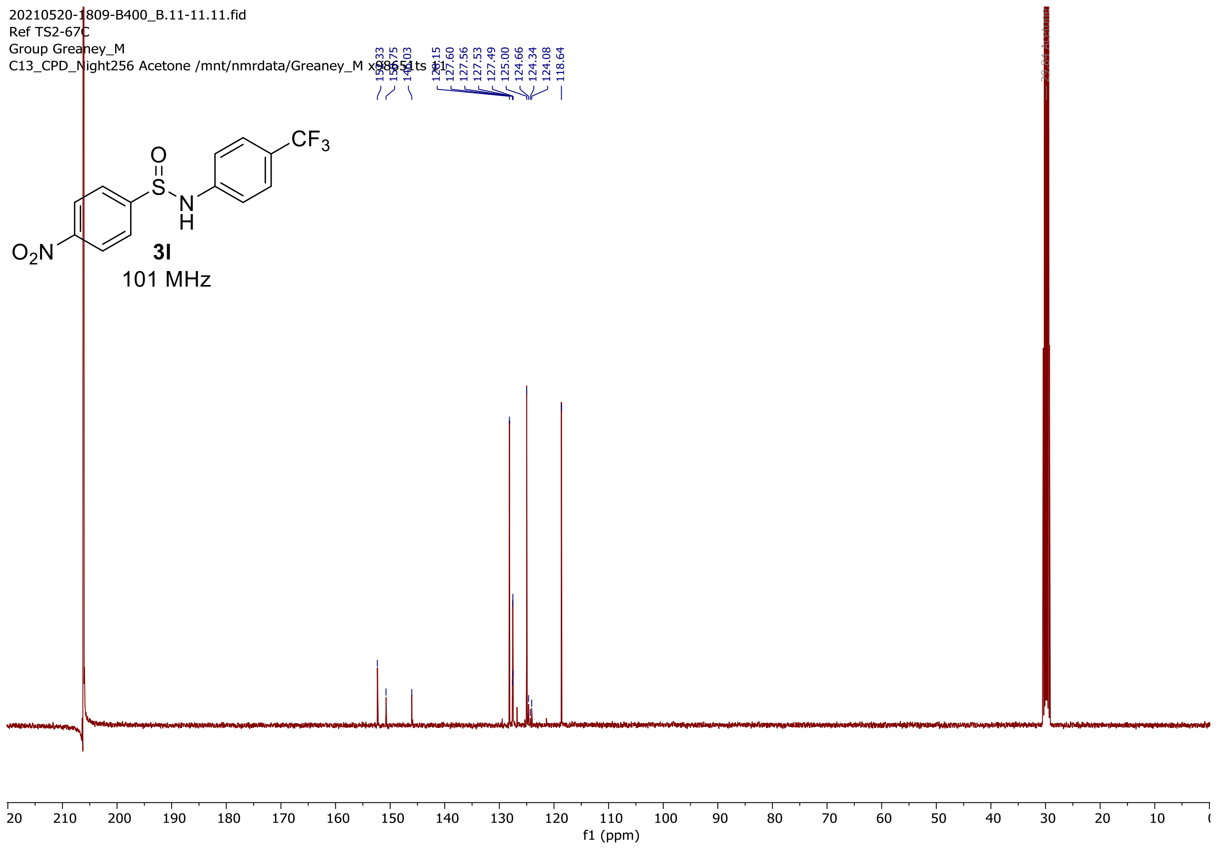

20210520-1809-B400_B.11-11.11.fid
Ref TS2-67C
Group Greaney_M
C13_CPD_Night256 Acetone /mnt/nmrdata/Greaney_M x98651ts 11
127.60
127.56
127.53
127.49
125.00
124.66
124.34
124.08
118.64
CF3
O
S
N
H
O2N
3l
101 MHz
220 210 200 190 180 170 160 150 140 130 120 110 100 90 80 70 60 50 40 30 20 10 0
f1 (ppm)

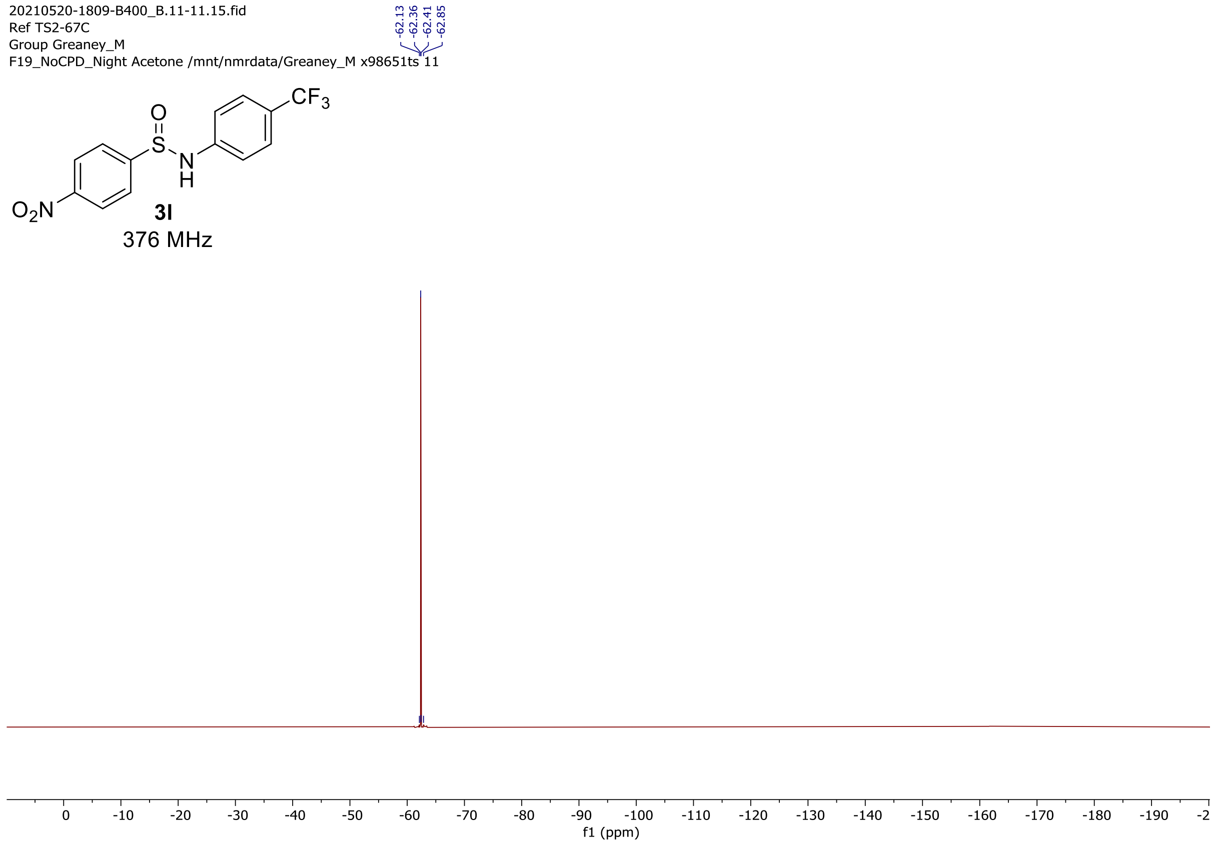

20210520-1809-B400_B.11-11.15.fid
Ref TS2-67C
Group Greaney_M
F19_NoCPD_Night Acetone /mnt/nmrdata/Greaney_M x98651ts 11
-62.13
-62.36
-62.41
-62.85
CF3
O
S
N
H
O2N
3l
376 MHz
0 -10 -20 -30 -40 -50 -60 -70 -80 -90 -100 -110 -120 -130 -140 -150 -160 -170 -180 -190 -200
f1 (ppm)

20210625-1422-B500_B.14-6.10.fid
Ref TS2-86C2
Group Greaney_M
H1_Day DMSO /mnt/nmrdata/Greaney_M x98651ts 6

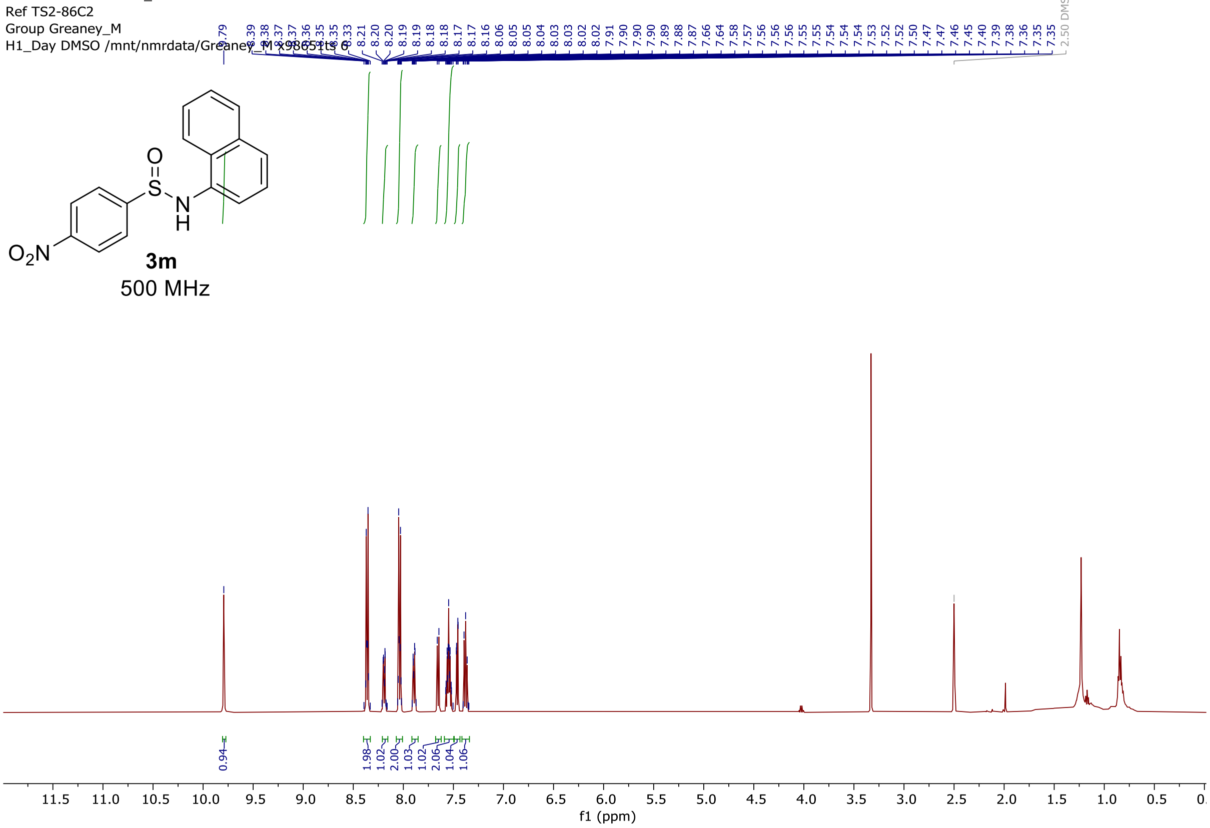

20210625-1422-B500_B.14-6.11.fid
Ref TS2-86C2
Group Greaney_M
C13_CPD_Day DMSO /mnt/nmrdata/Greaney_M x98651ts 6

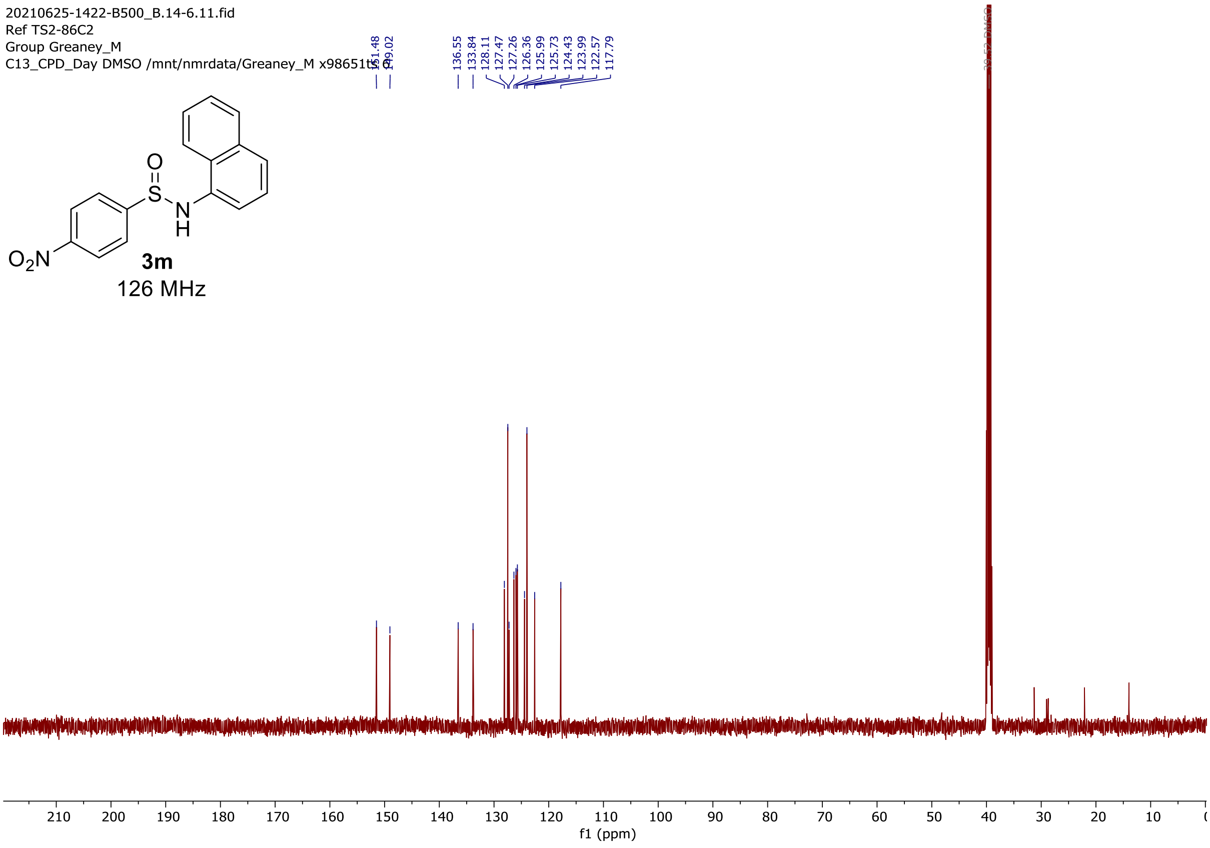

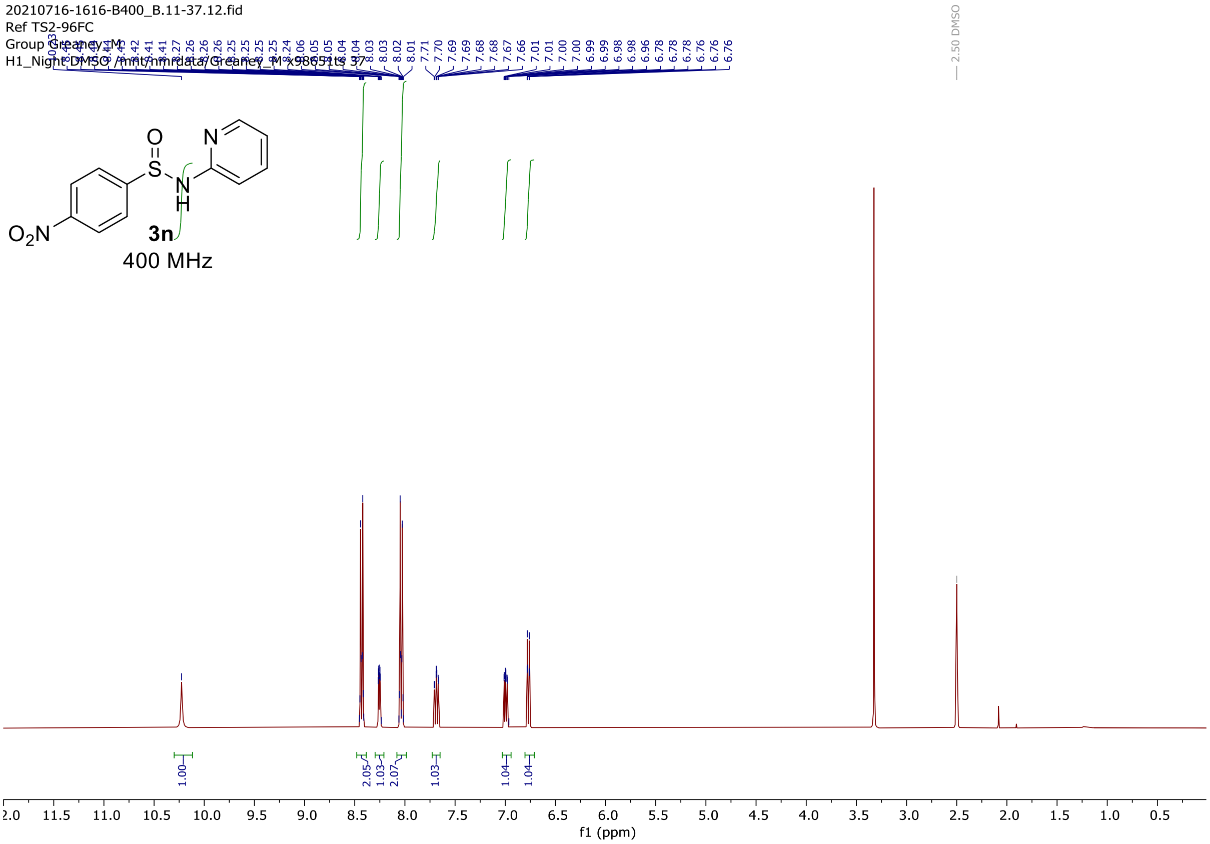
20210716-1616-B400_B.11-37.12.fid
Ref TS2-96FC
Group Greaney_M
H1_Night DMSO /mnt/nmrdata/Greaney_M x98651ts 37
8.27 8.26 8.26 8.25 8.25 8.25 8.24 8.06 8.05 8.05 8.04 8.04 8.03 8.03 8.02 8.01 7.71 7.70 7.69 7.69 7.68 7.67 7.66 7.01 7.01 7.00 6.99 6.99 6.98 6.98 6.96 6.78 6.78 6.76 6.76 6.76
2.50 DMSO
O
N
S
N
H
O2N
3n
400 MHz
1.00
2.05
1.03
2.07
1.03
1.04
1.04
f1 (ppm)

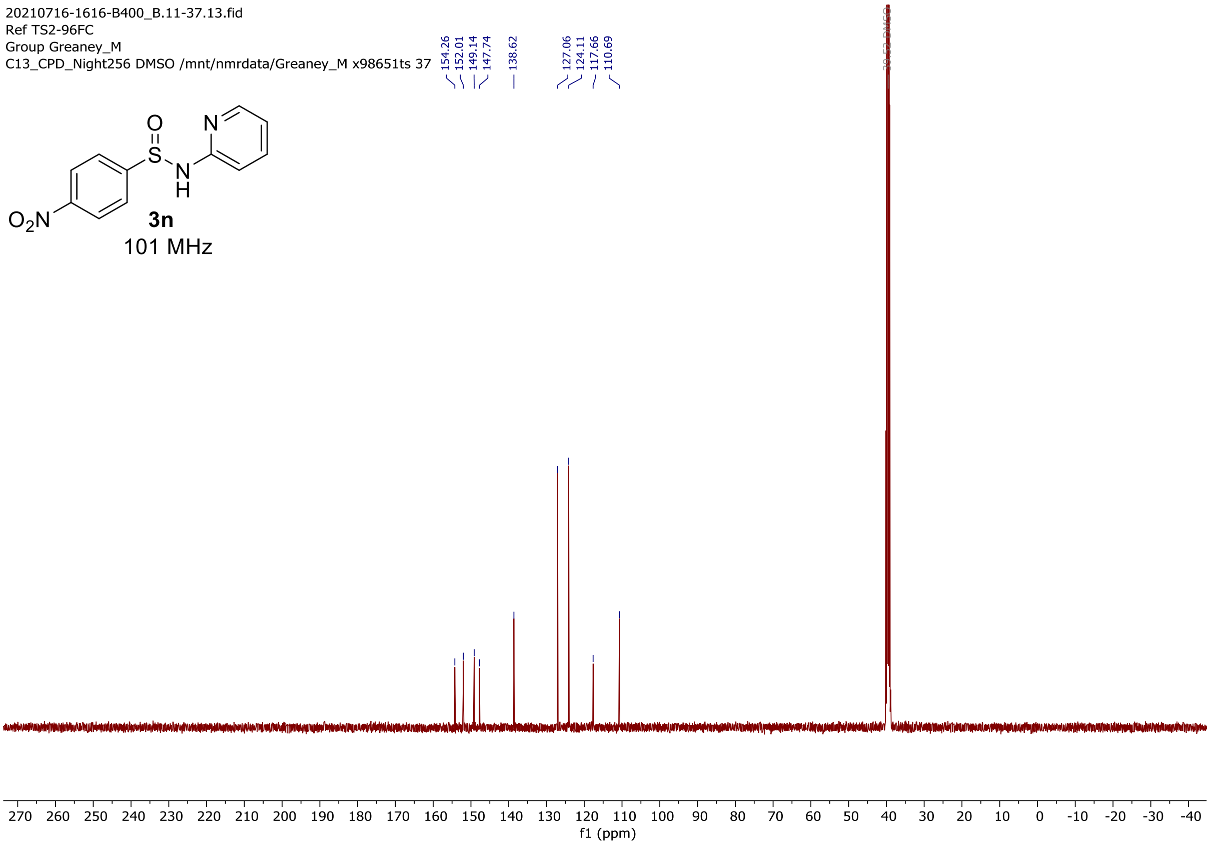
20210716-1616-B400_B.11-37.13.fid
Ref TS2-96FC
Group Greaney_M
C13_CPD_Night256 DMSO /mnt/nmrdata/Greaney_M x98651ts 37
154.26
152.01
149.14
147.74
138.62
127.06
124.11
117.66
110.69
O
N
S
N
H
O2N
3n
101 MHz
f1 (ppm)

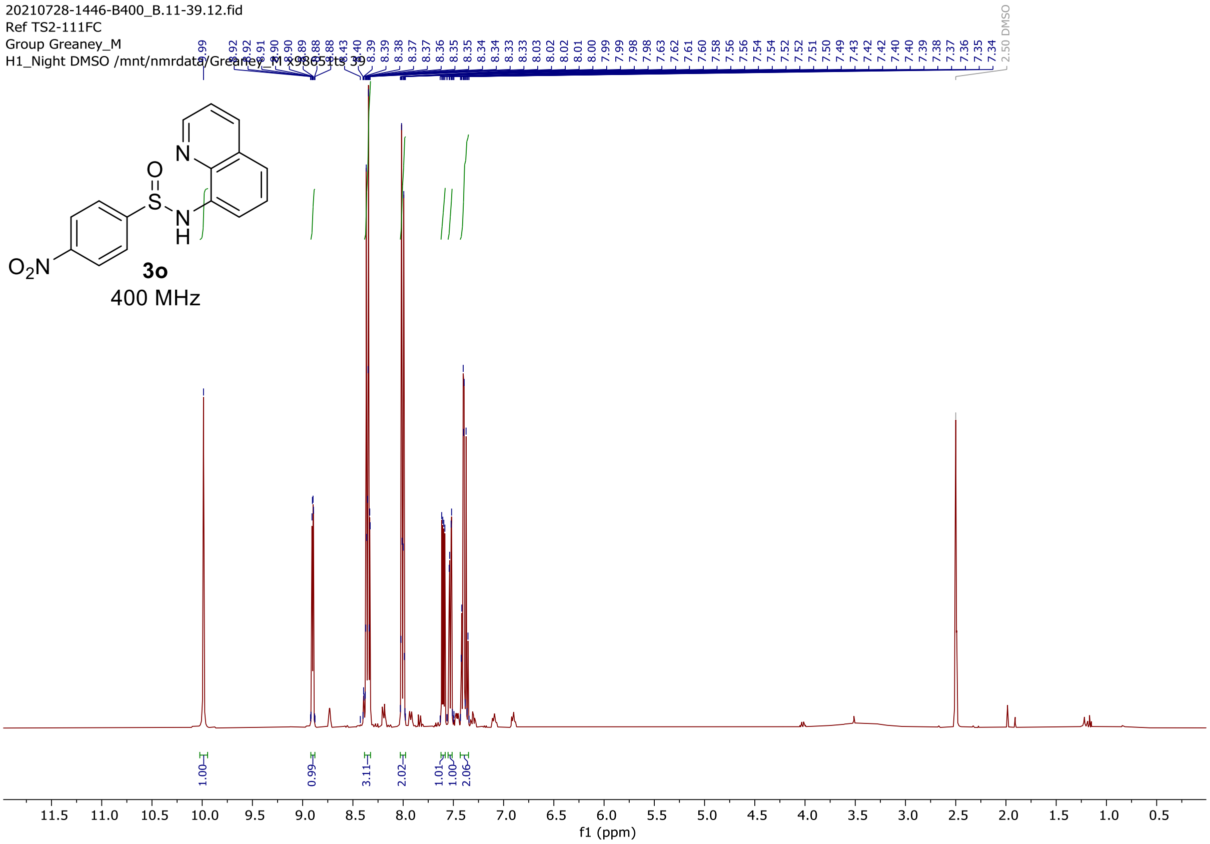
20210728-1446-B400_B.11-39.12.fid
Ref TS2-111FC
Group Greaney_M
H1_Night DMSO /mnt/nmrdata/Greaney_M [illegible] 39
9.99 8.92 8.92 8.91 8.90 8.90 8.89 8.88 8.88 8.43 8.40 8.39 8.39 8.38 8.37 8.37 8.36 8.35 8.35 8.34 8.33 8.33 8.03 8.02 8.02 8.01 8.00 7.99 7.99 7.98 7.98 7.63 7.62 7.61 7.60 7.58 7.56 7.56 7.54 7.54 7.52 7.52 7.51 7.50 7.49 7.43 7.42 7.42 7.40 7.40 7.39 7.38 7.37 7.36 7.35 7.34
2.50 DMSO
N
O
S
N
H
O_2N
3o
400 MHz
1.00 0.99 3.11 2.02 1.01 1.00 2.06
11.5 11.0 10.5 10.0 9.5 9.0 8.5 8.0 7.5 7.0 6.5 6.0 5.5 5.0 4.5 4.0 3.5 3.0 2.5 2.0 1.5 1.0 0.5
f1 (ppm)

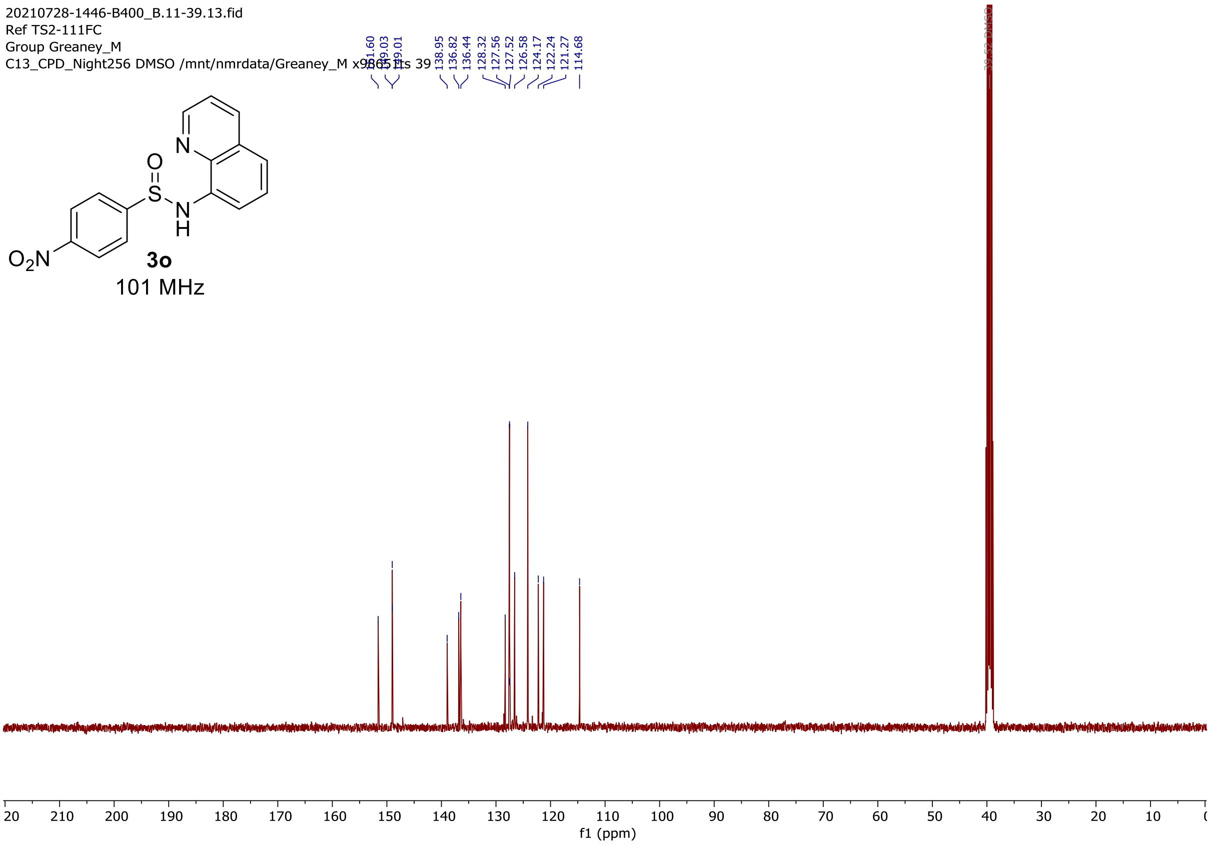
20210728-1446-B400_B.11-39.13.fid
Ref TS2-111FC
Group Greaney_M
C13_CPD_Night256 DMSO /mnt/nmrdata/Greaney_M [illegible] 39
151.60 149.03 149.01 138.95 136.82 136.44 128.32 127.56 127.52 126.58 124.17 122.24 121.27 114.68
N
O
S
N
H
O_2N
3o
101 MHz
[illegible] 210 200 190 180 170 160 150 140 130 120 110 100 90 80 70 60 50 40 30 20 10 [illegible]
f1 (ppm)

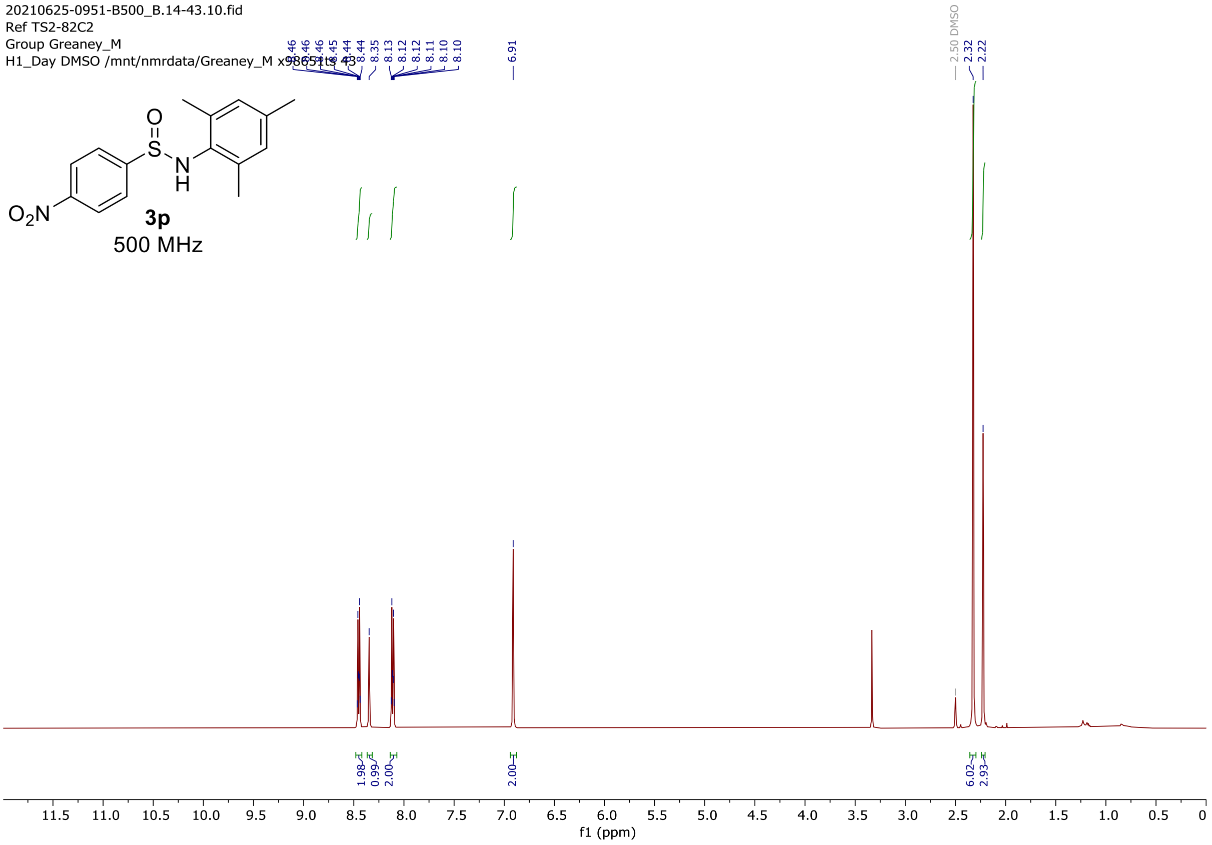
20210625-0951-B500_B.14-43.10.fid
Ref TS2-82C2
Group Greaney_M
H1_Day DMSO /mnt/nmrdata/Greaney_M x98651ts 43
3p
500 MHz
8.46
8.46
8.46
8.45
8.44
8.44
8.35
8.13
8.12
8.12
8.11
8.10
8.10
6.91
2.50 DMSO
2.32
2.22
1.98
0.99
2.00
2.00
6.02
2.93
f1 (ppm)

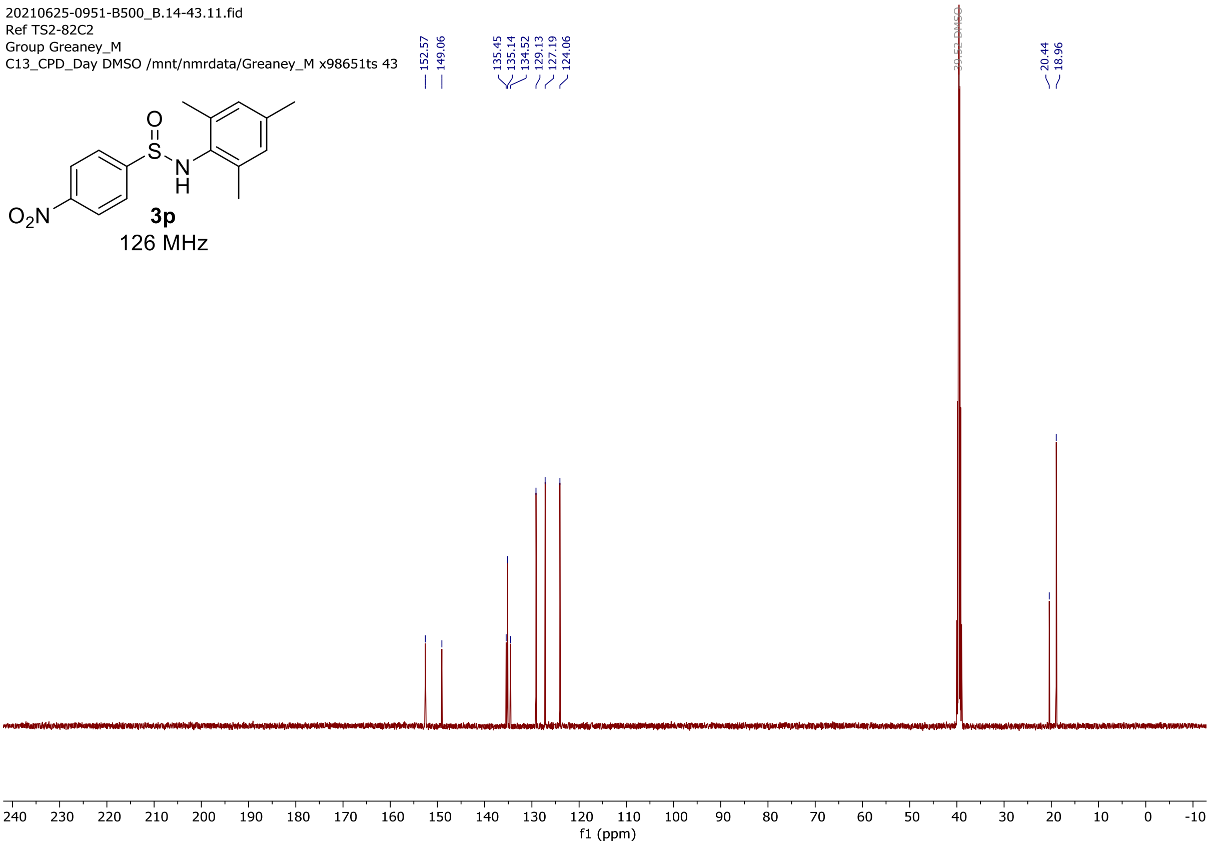
20210625-0951-B500_B.14-43.11.fid
Ref TS2-82C2
Group Greaney_M
C13_CPD_Day DMSO /mnt/nmrdata/Greaney_M x98651ts 43
3p
126 MHz
152.57
149.06
135.45
135.14
134.52
129.13
127.19
124.06
20.44
18.96
f1 (ppm)

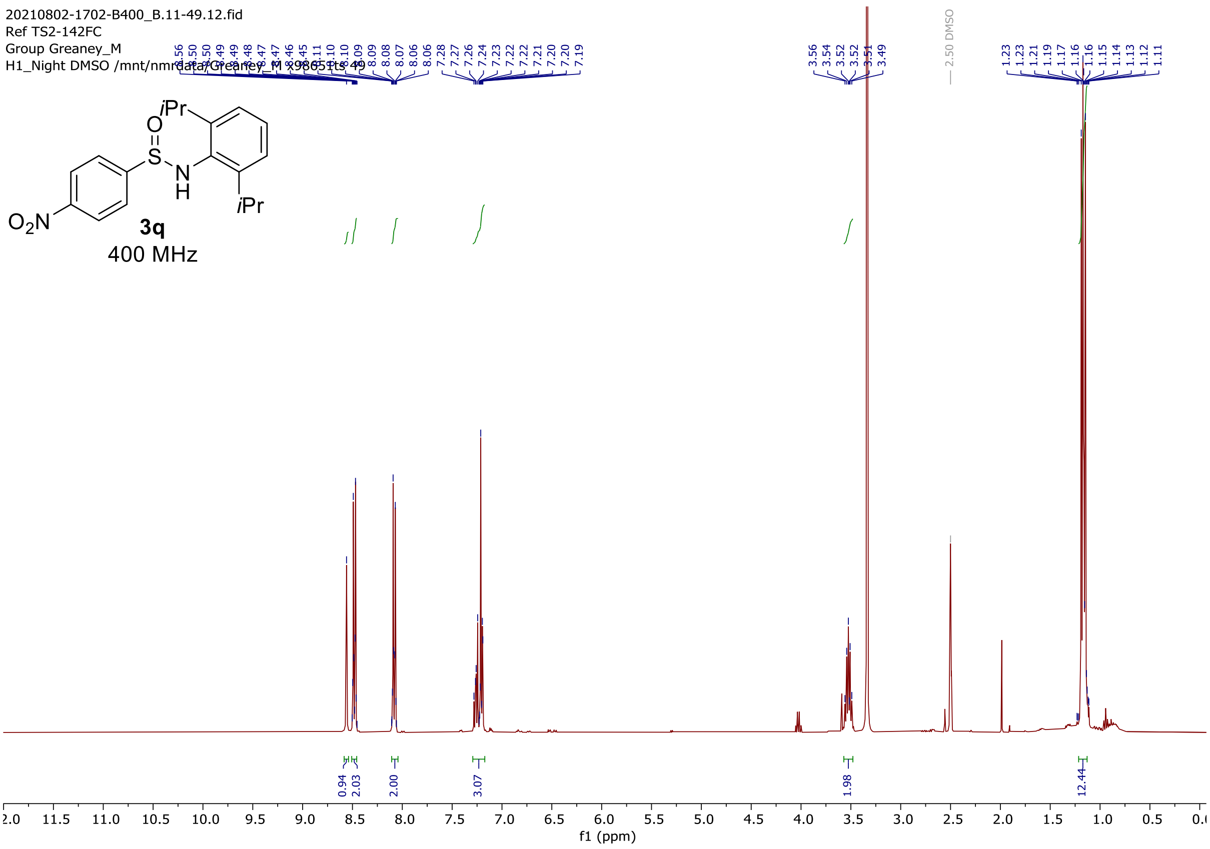

20210802-1702-B400_B.11-49.12.fid
Ref TS2-142FC
Group Greaney_M
2.50 DMSO
3.56
3.54
3.52
3.52
3.51
3.49
1.23
1.23
1.21
1.19
1.17
1.16
1.16
1.15
1.14
1.13
1.12
1.11
*i*Pr
O
S
N
H
*i*Pr
O_2N
3q
400 MHz
0.94
2.03
2.00
3.07
1.98
12.44
f1 (ppm)

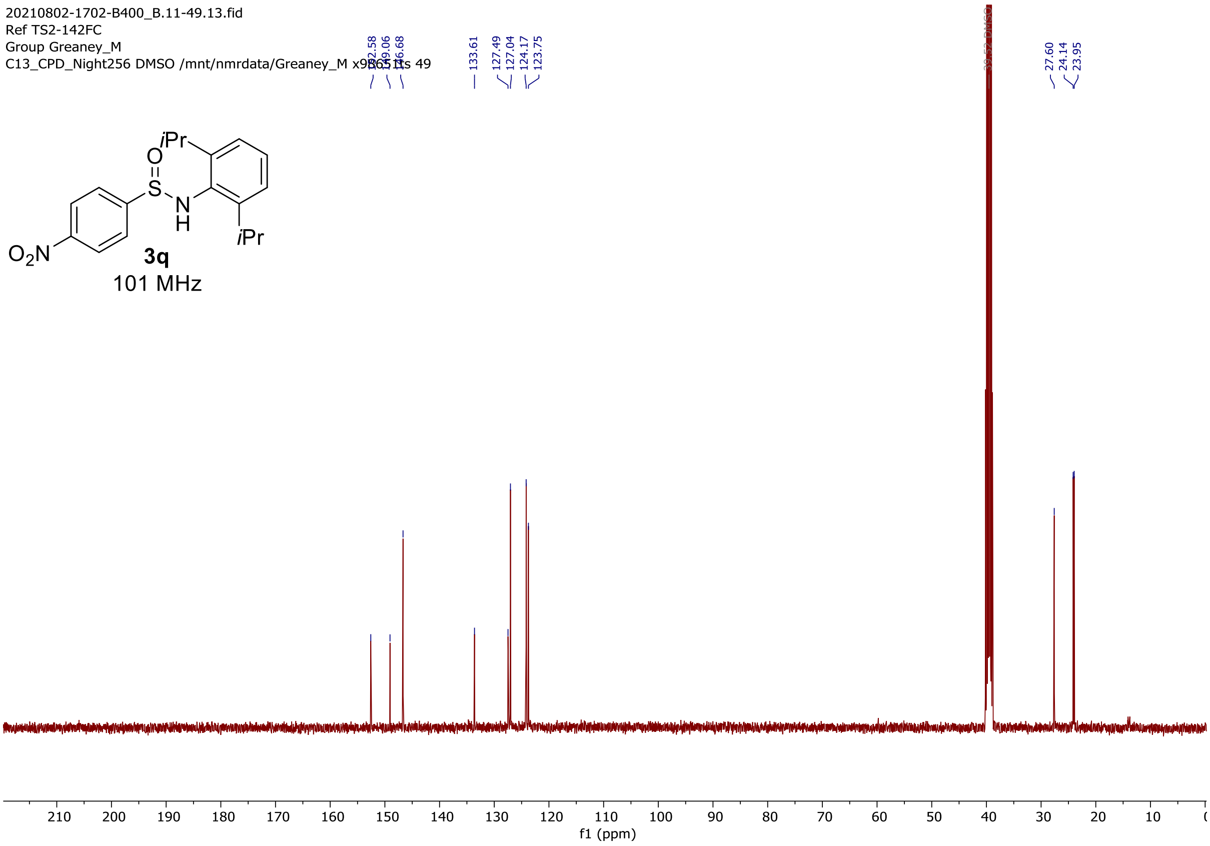

20210802-1702-B400_B.11-49.13.fid
Ref TS2-142FC
Group Greaney_M
133.61
127.49
127.04
124.17
123.75
27.60
24.14
23.95
*i*Pr
O
S
N
H
*i*Pr
O_2N
3q
101 MHz
f1 (ppm)

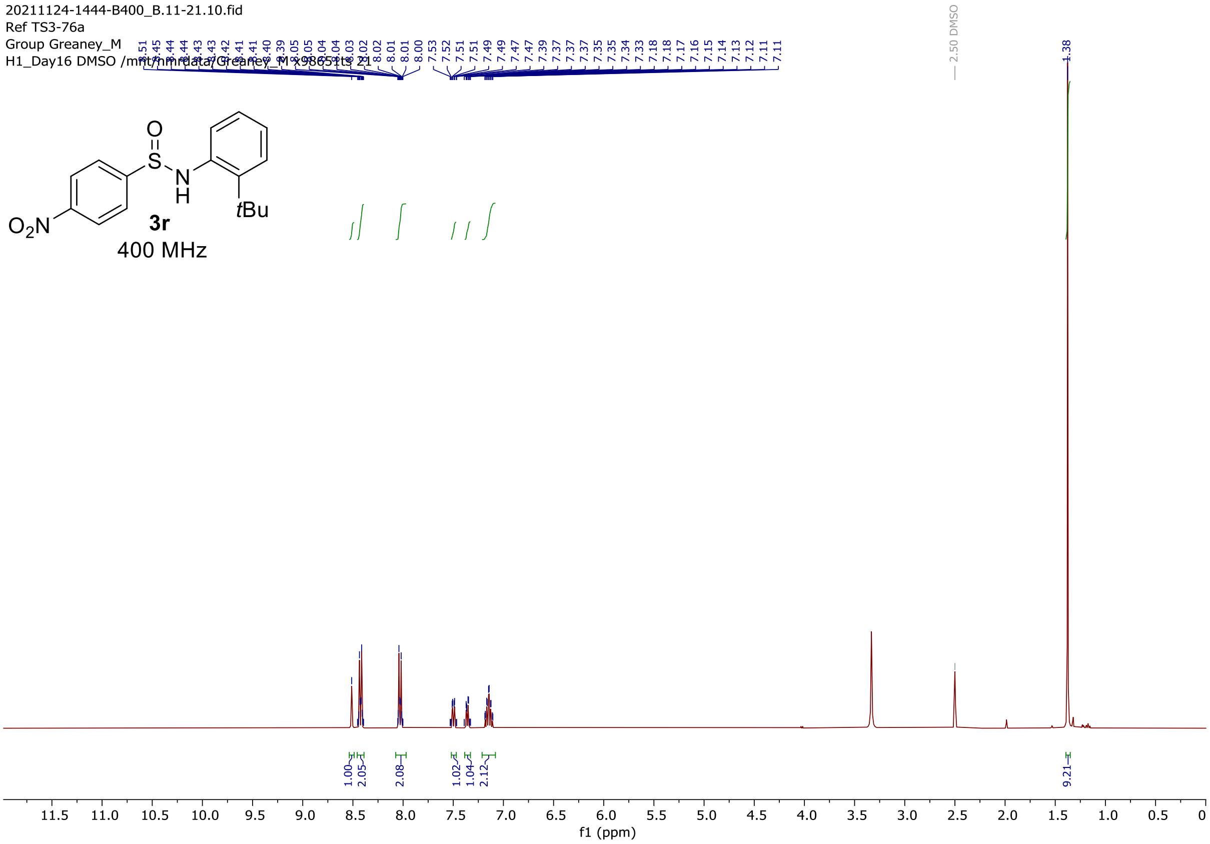
20211124-1444-B400_B.11-21.10.fid
Ref TS3-76a
Group Greaney_M
H1_Day16 DMSO /mnt/nmrdata/Greaney_M x98651ts 21
3r
400 MHz
tBu
2.50 DMSO
1.38
f1 (ppm)

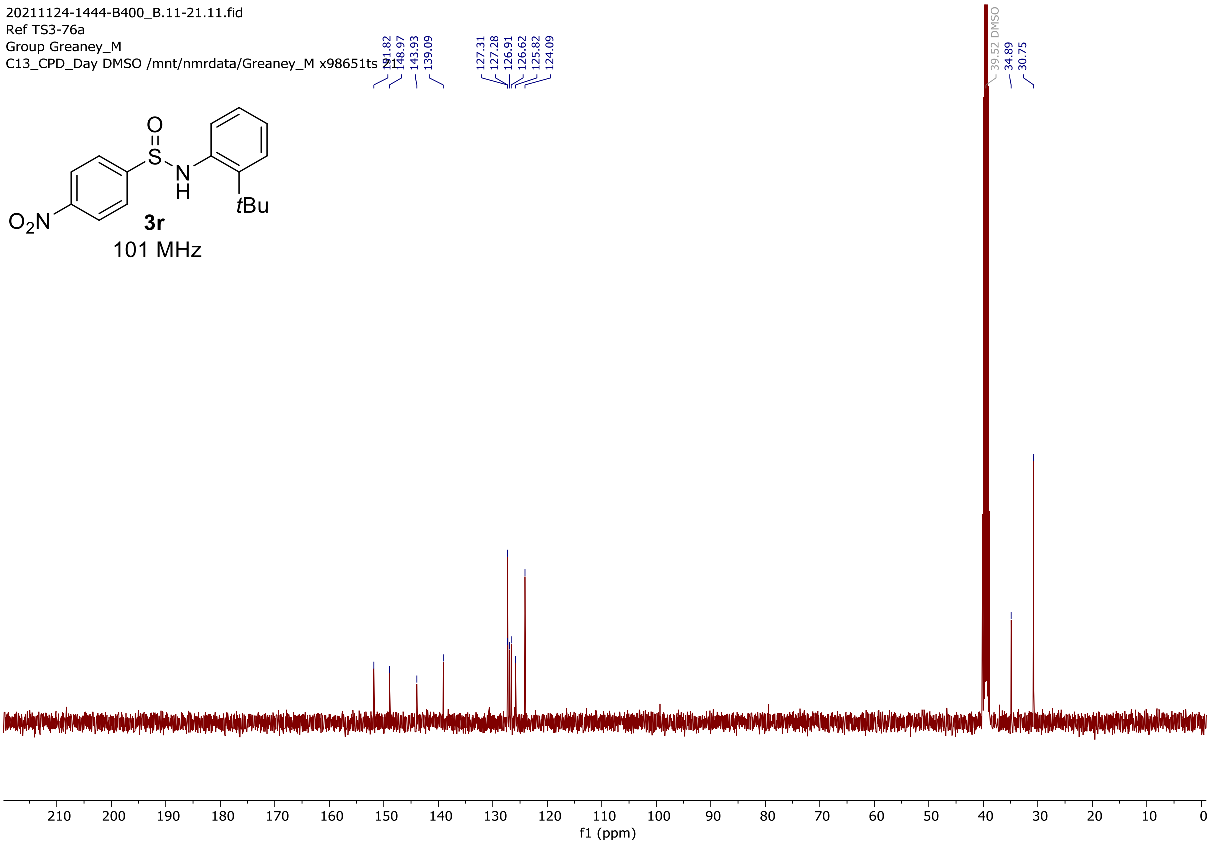
20211124-1444-B400_B.11-21.11.fid
Ref TS3-76a
Group Greaney_M
C13_CPD_Day DMSO /mnt/nmrdata/Greaney_M x98651ts 21
3r
101 MHz
tBu
39.52 DMSO
34.89
30.75
f1 (ppm)

20210906-1036-B500_B.14-24.10.fid
Ref TS2-155FC
Group Greaney_M
H1_Night DMSO /mnt/nmrdata/Greaney_M x98651ts 24

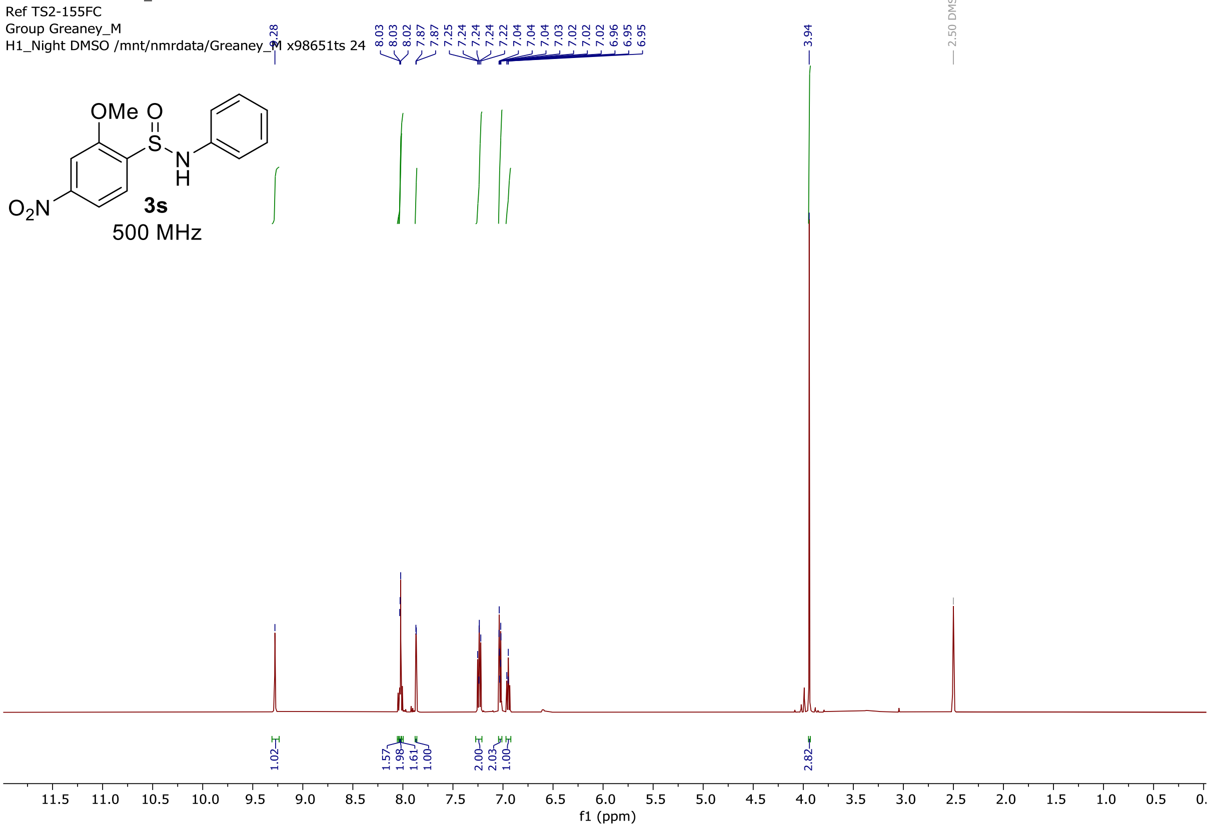

20210906-1036-B500_B.14-24.11.fid
Ref TS2-155FC
Group Greaney_M
C13_CPD_Night256 DMSO /mnt/nmrdata/Greaney_M x98651ts 24

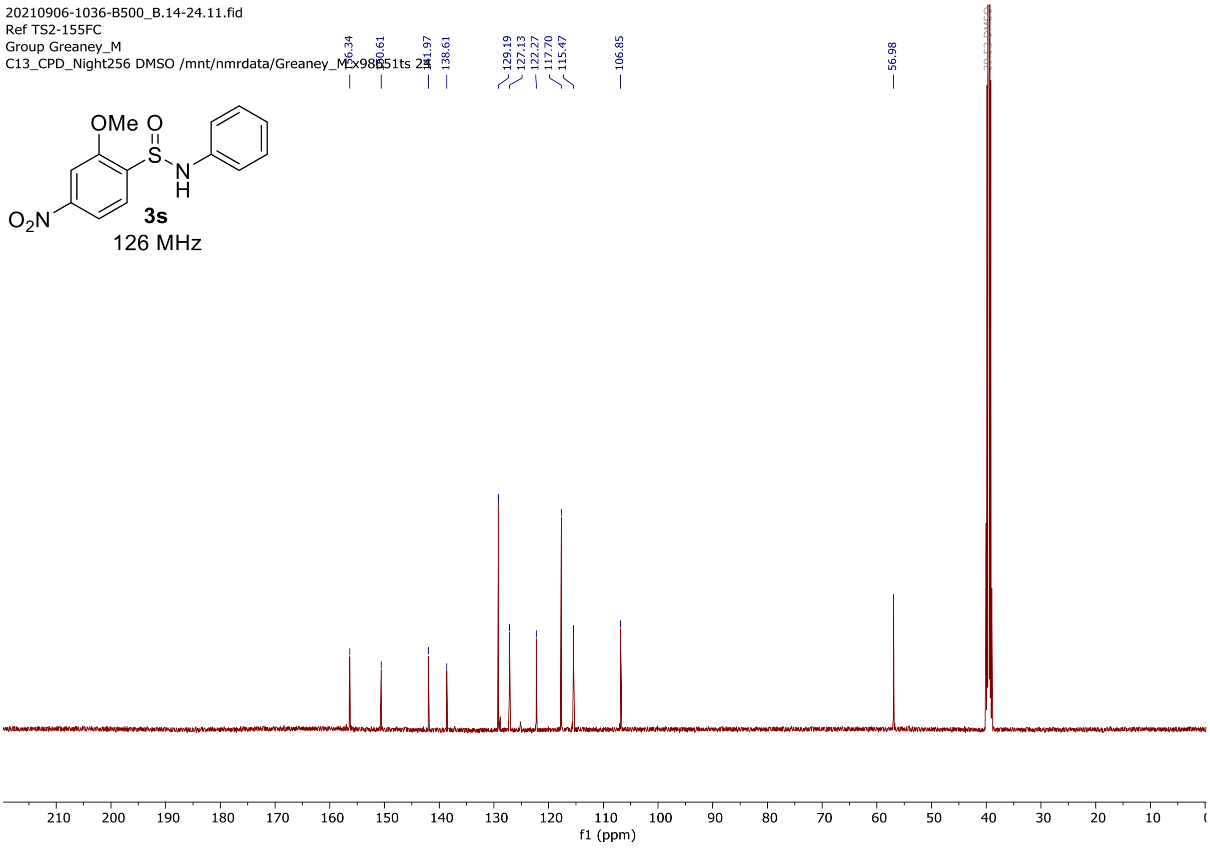

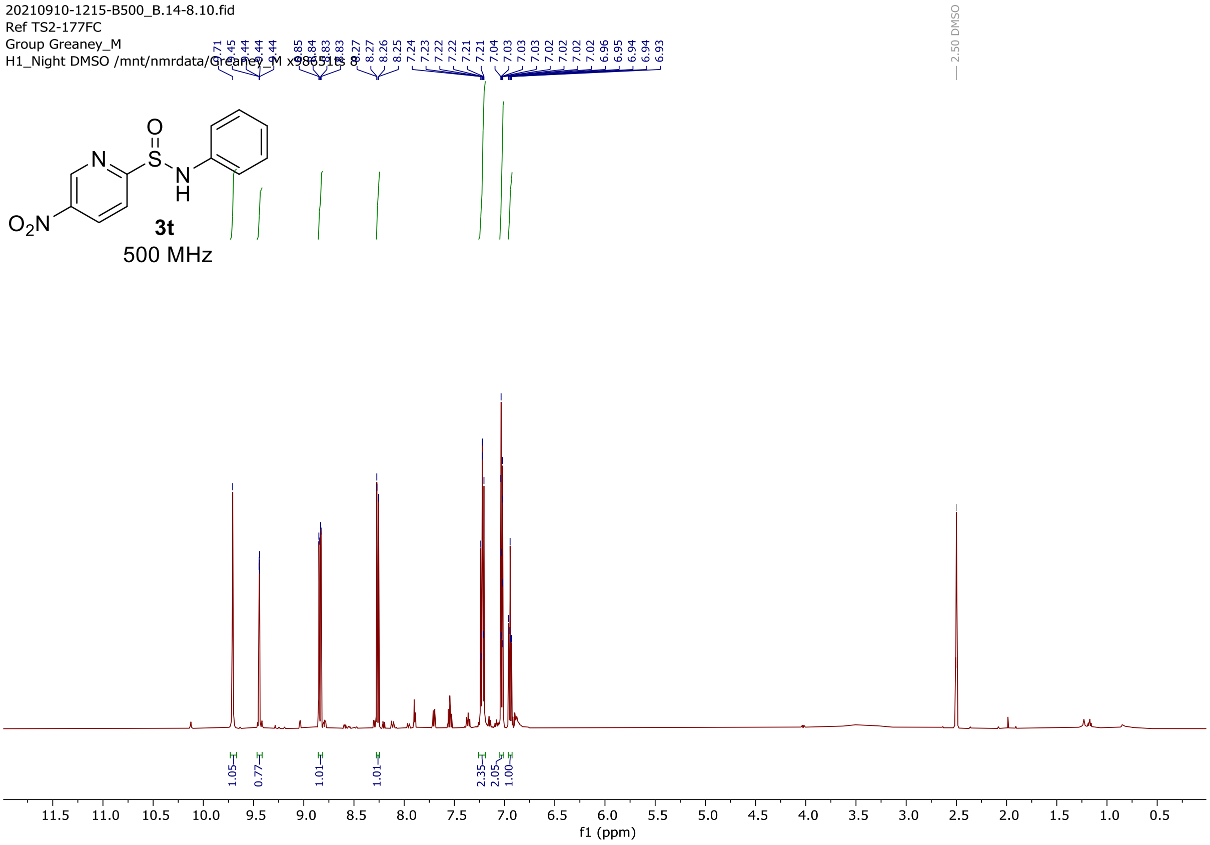
20210910-1215-B500_B.14-8.10.fid
Ref TS2-177FC
Group Greaney_M
H1_Night DMSO /mnt/nmrdata/Greaney_M x98651ts 8
3t
500 MHz
2.50 DMSO
f1 (ppm)

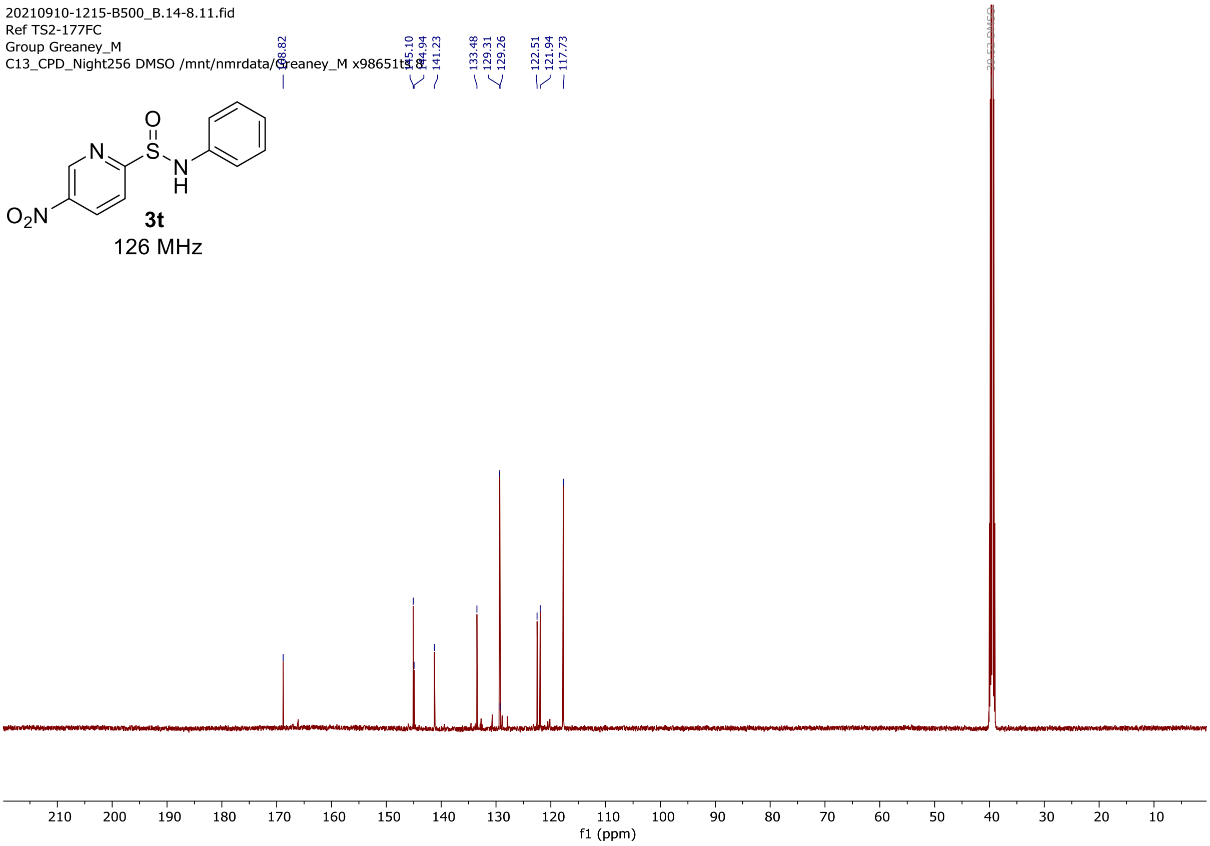
20210910-1215-B500_B.14-8.11.fid
Ref TS2-177FC
Group Greaney_M
C13_CPD_Night256 DMSO /mnt/nmrdata/Greaney_M x98651ts 9
3t
126 MHz
168.82
145.10
144.94
141.23
133.48
129.31
129.26
122.51
121.94
117.73
f1 (ppm)

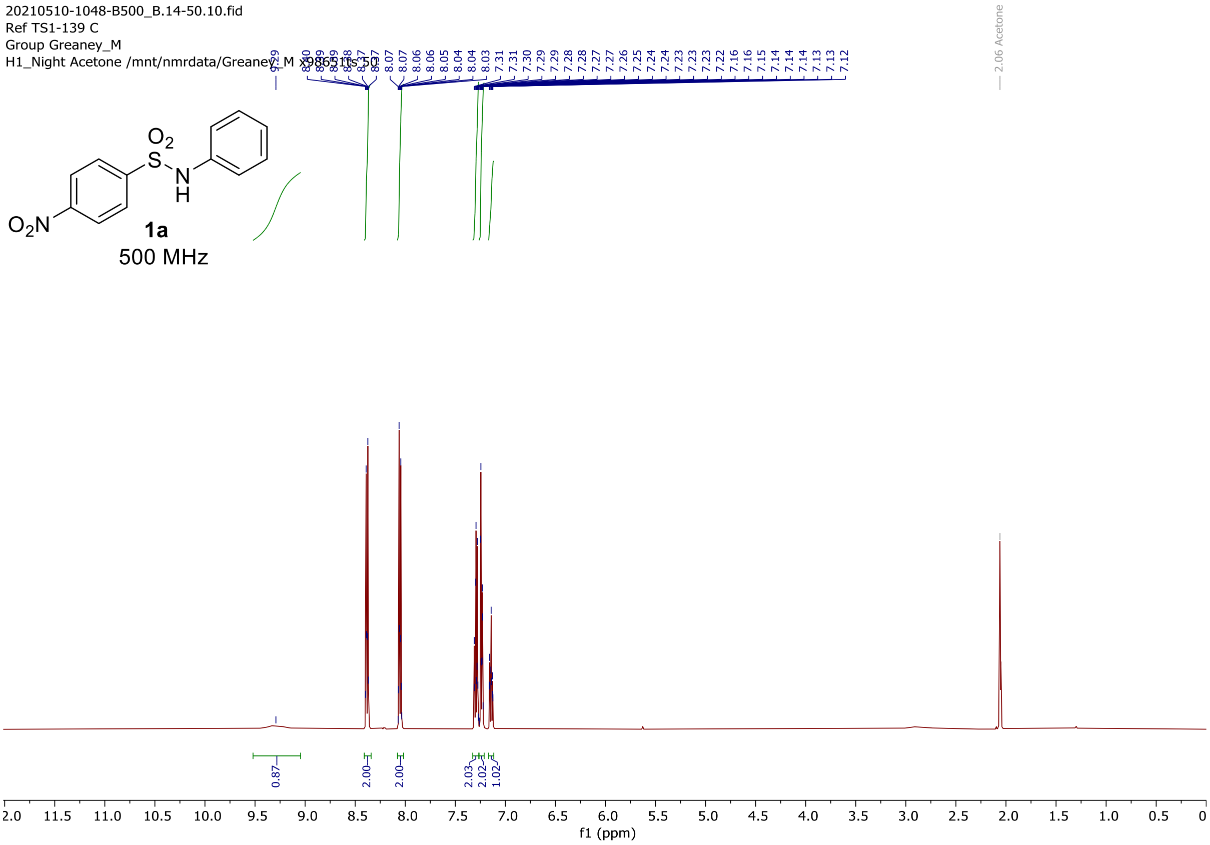
20210510-1048-B500_B.14-50.10.fid
Ref TS1-139 C
Group Greaney_M
2.06 Acetone
1a
500 MHz
f1 (ppm)

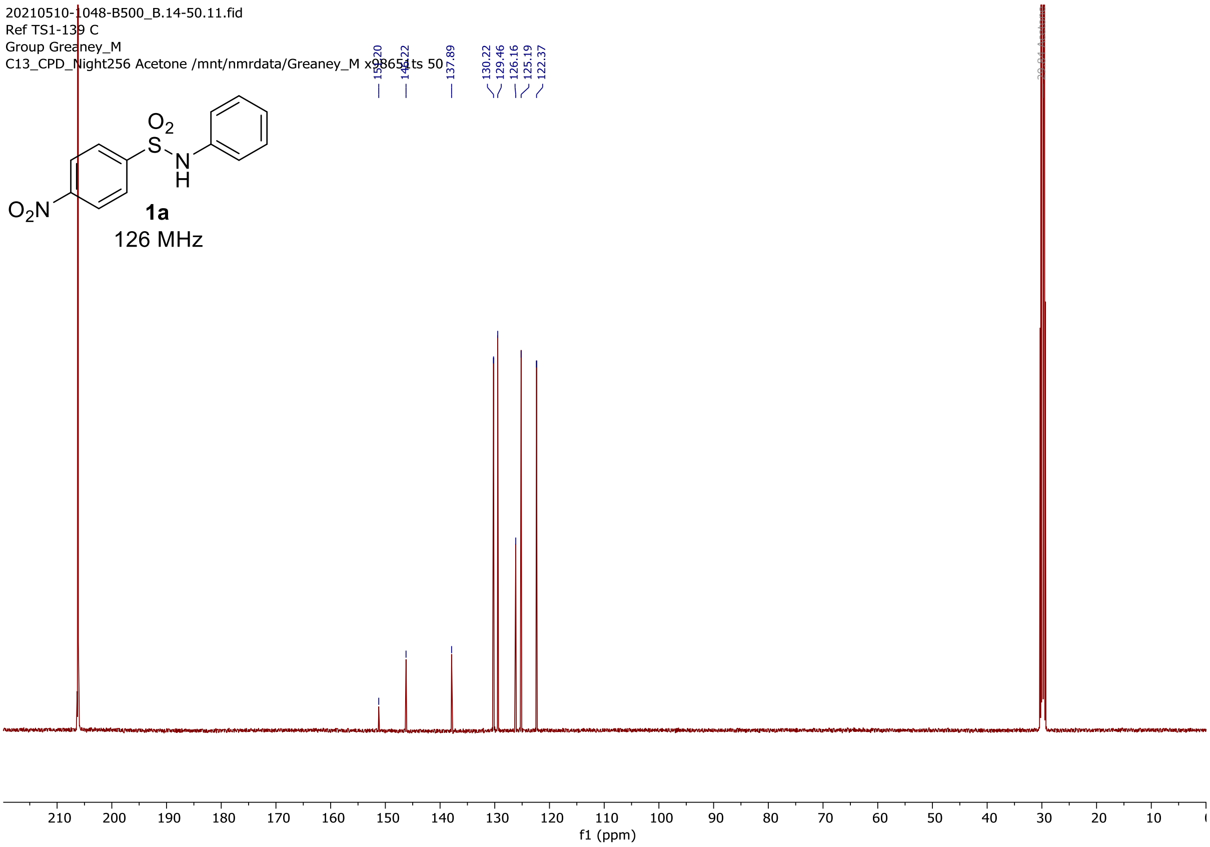
20210510-1048-B500_B.14-50.11.fid
Ref TS1-139 C
Group Greaney_M
137.89
130.22
129.46
126.16
125.19
122.37
1a
126 MHz
f1 (ppm)

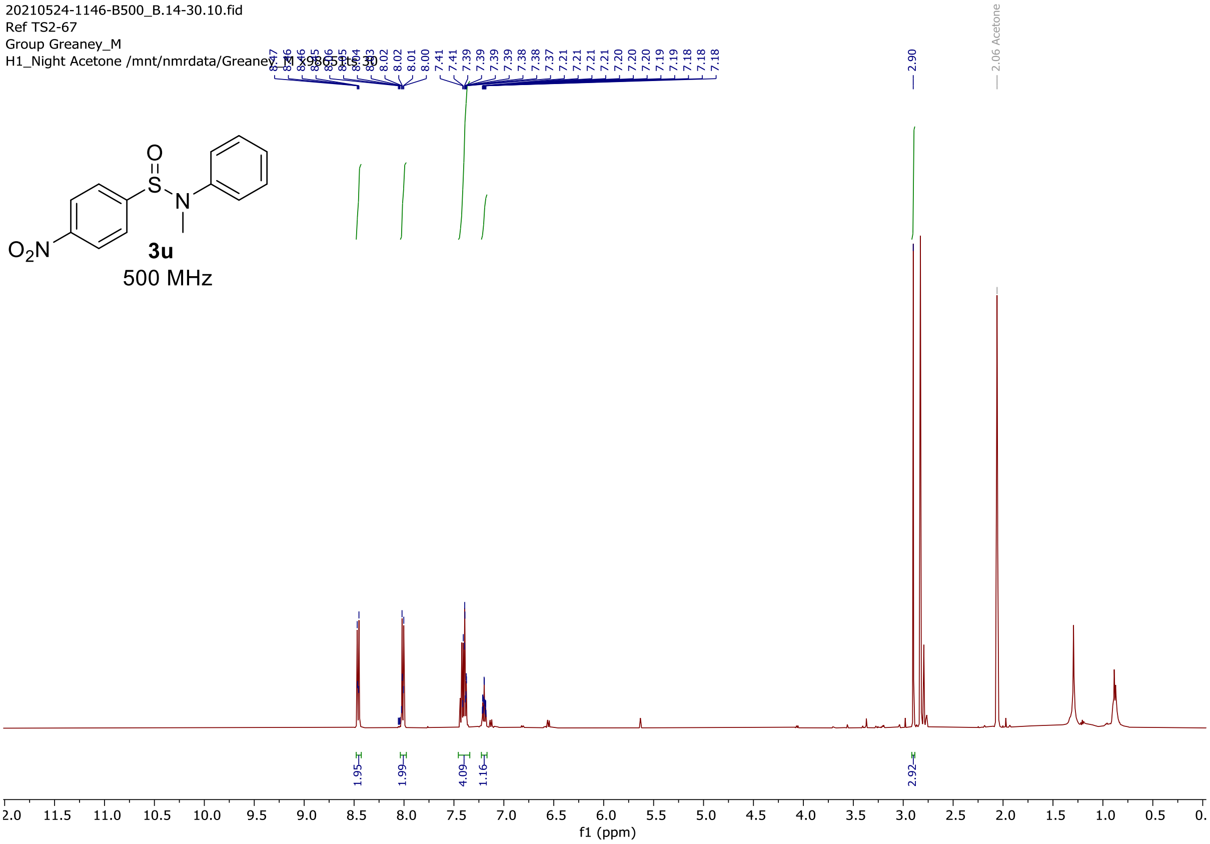
20210524-1146-B500_B.14-30.10.fid
Ref TS2-67
Group Greaney_M
H1_Night Acetone /mnt/nmrdata/Greaney_M x98651ts 30
2.90
2.06 Acetone
O
S
N
O_2N
3u
500 MHz
1.95
1.99
4.09
1.16
2.92
f1 (ppm)

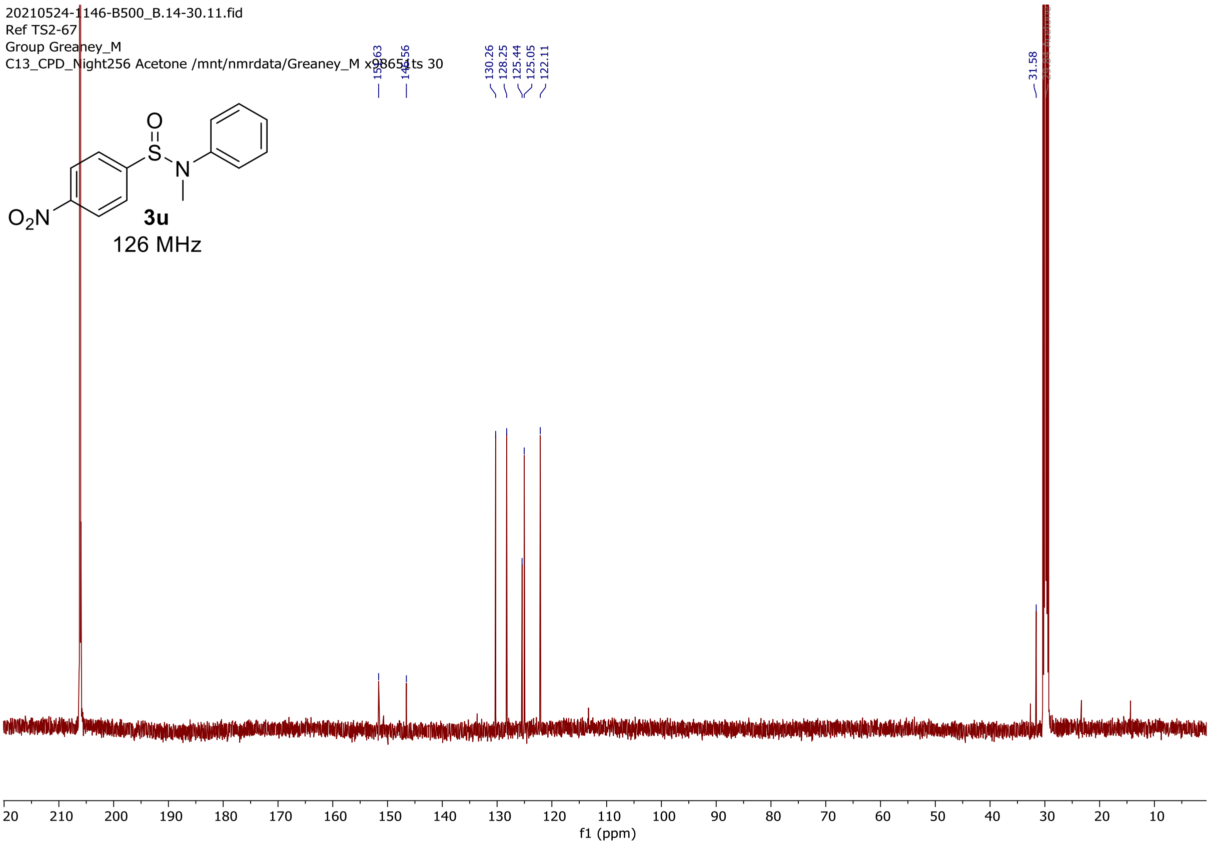
20210524-1146-B500_B.14-30.11.fid
Ref TS2-67
Group Greaney_M
C13_CPD_Night256 Acetone /mnt/nmrdata/Greaney_M x98651ts 30
151.63
146.56
130.26
128.25
125.44
125.05
122.11
31.58
O
S
N
O_2N
3u
126 MHz
f1 (ppm)

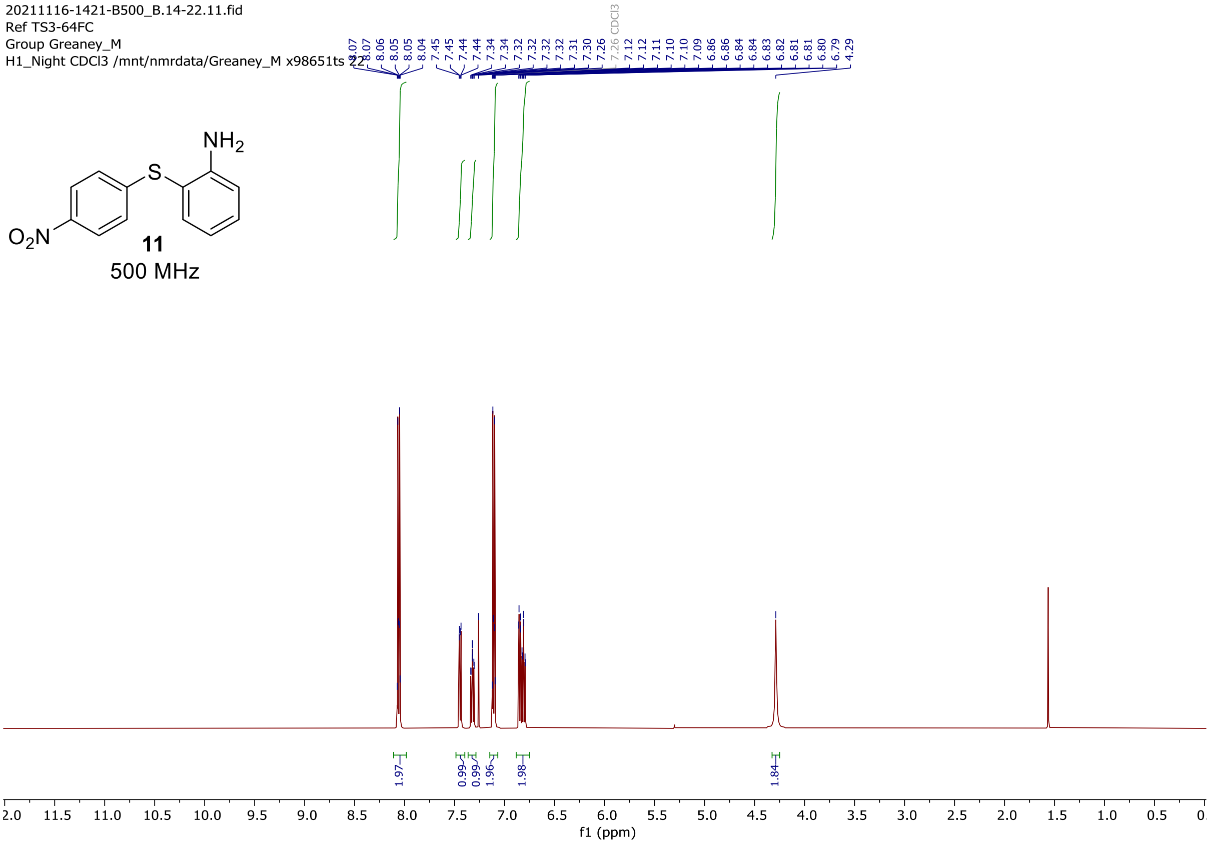
20211116-1421-B500_B.14-22.11.fid
Ref TS3-64FC
Group Greaney_M
H1_Night CDCl3 /mnt/nmrdata/Greaney_M x98651ts 22
8.07
8.07
8.06
8.05
8.05
8.04
7.45
7.45
7.44
7.44
7.34
7.34
7.32
7.32
7.32
7.32
7.31
7.30
7.26
7.26 CDCl3
7.12
7.12
7.11
7.10
7.09
6.86
6.86
6.84
6.84
6.83
6.82
6.81
6.81
6.80
6.79
4.29
NH2
S
O2N
11
500 MHz
1.97
0.99
0.99
1.96
1.98
1.84
2.0
11.5
11.0
10.5
10.0
9.5
9.0
8.5
8.0
7.5
7.0
6.5
6.0
5.5
5.0
4.5
4.0
3.5
3.0
2.5
2.0
1.5
1.0
0.5
0
f1 (ppm)

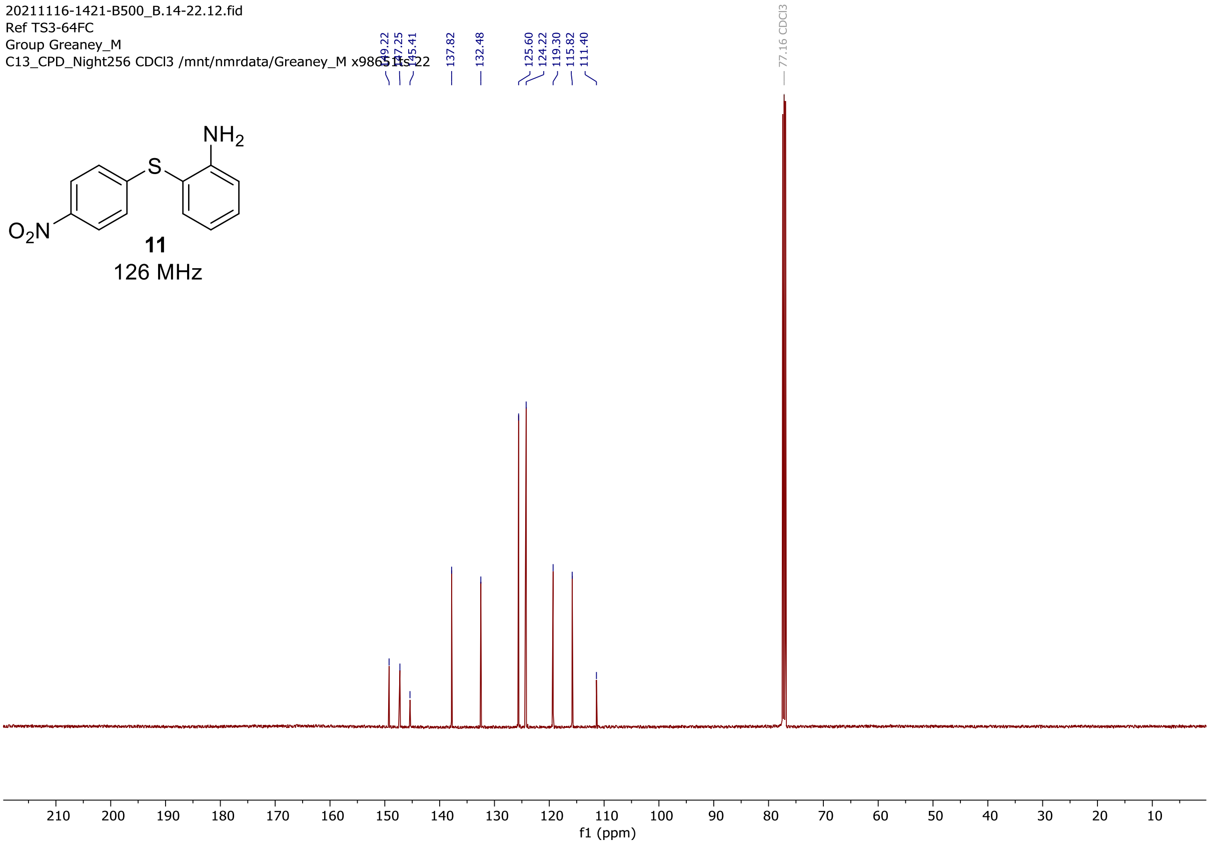
20211116-1421-B500_B.14-22.12.fid
Ref TS3-64FC
Group Greaney_M
C13_CPD_Night256 CDCl3 /mnt/nmrdata/Greaney_M x98651ts 22
149.22
147.25
145.41
137.82
132.48
125.60
124.22
119.30
115.82
111.40
77.16 CDCl3
NH2
S
O2N
11
126 MHz
210
200
190
180
170
160
150
140
130
120
110
100
90
80
70
60
50
40
30
20
10
f1 (ppm)

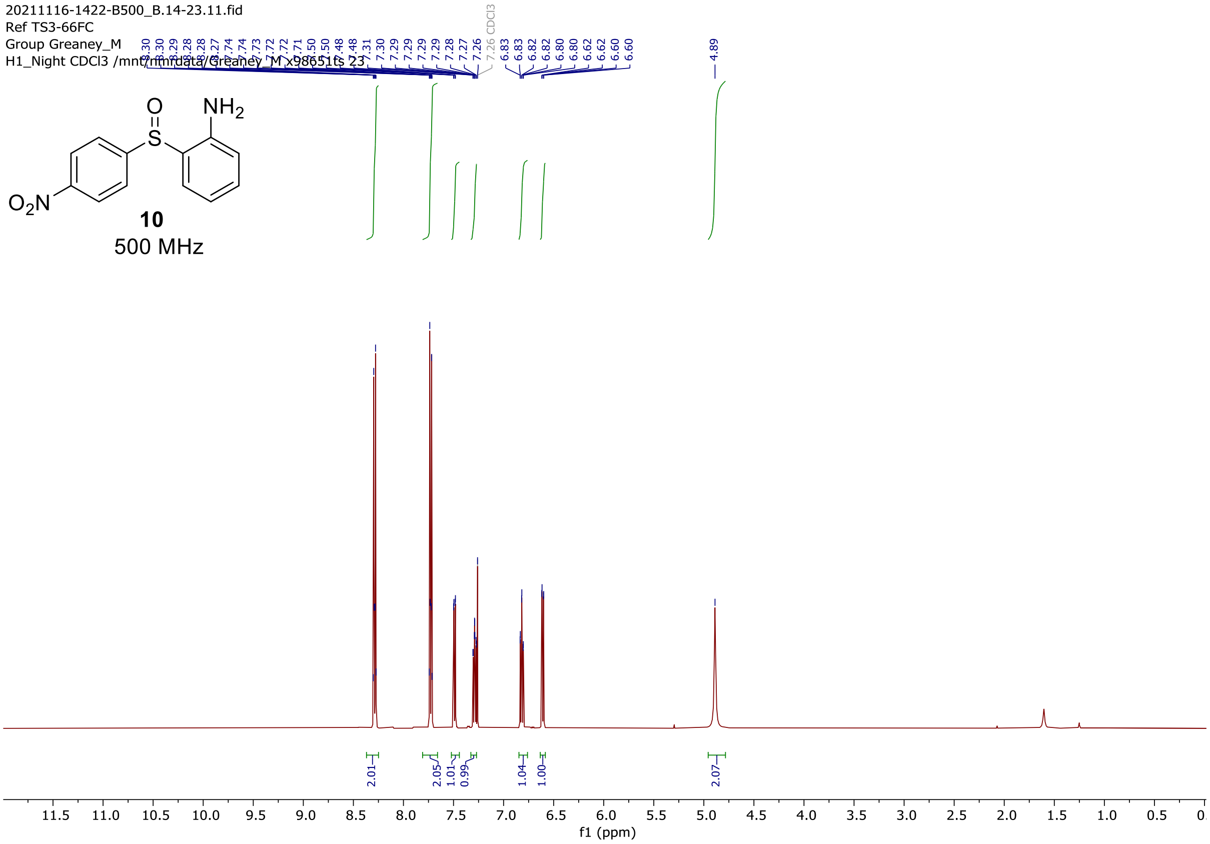
20211116-1422-B500_B.14-23.11.fid
Ref TS3-66FC
Group Greaney_M
H1_Night CDCl3 /mnt/nmrdata/Greaney_M x98651ts 23
10
500 MHz
4.89
f1 (ppm)

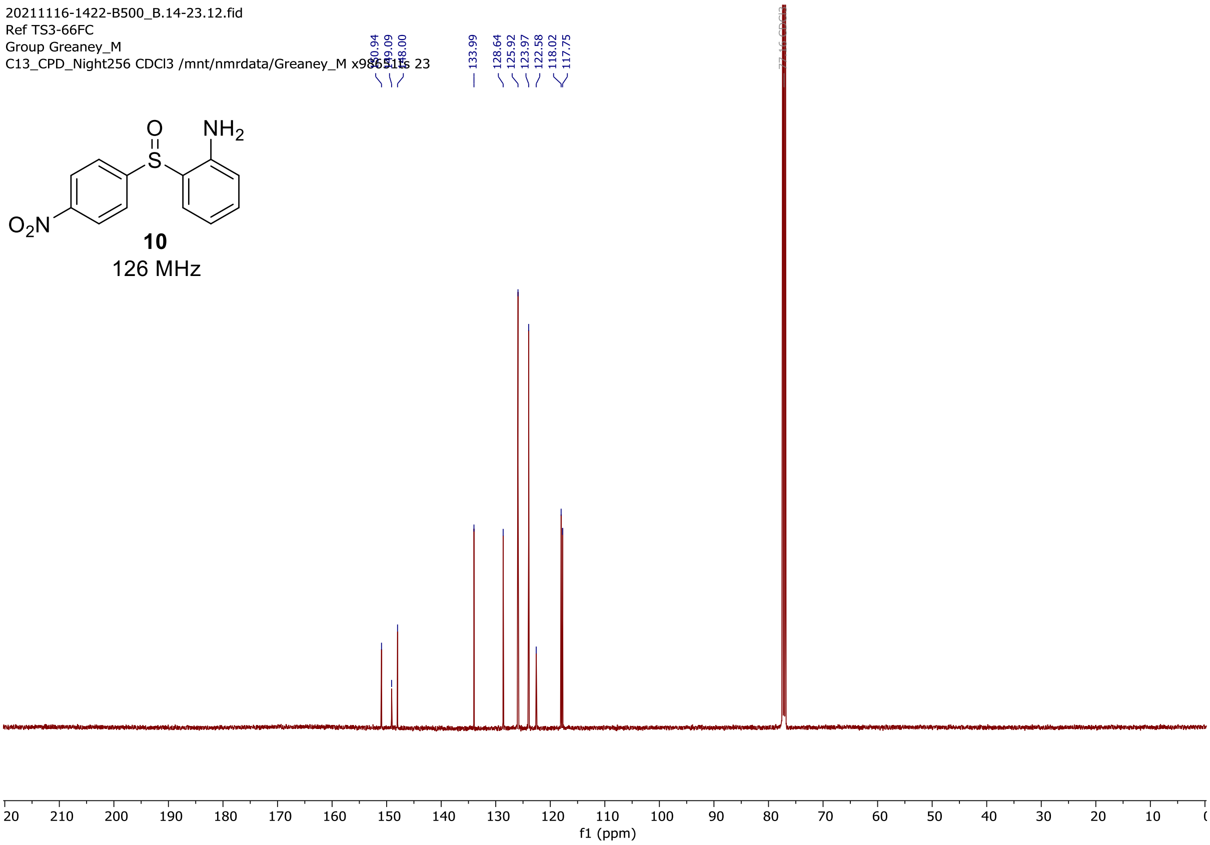
20211116-1422-B500_B.14-23.12.fid
Ref TS3-66FC
Group Greaney_M
C13_CPD_Night256 CDCl3 /mnt/nmrdata/Greaney_M x98651ts 23
10
126 MHz
133.99
128.64
125.92
123.97
122.58
118.02
117.75
f1 (ppm)

20210106-1609-B400_B.12-15.10.fid
Ref TS1-72 2D
Group Greaney_M
H1_Night CDCl3 /mnt/nmrdata/Greaney_M x98651ts 15

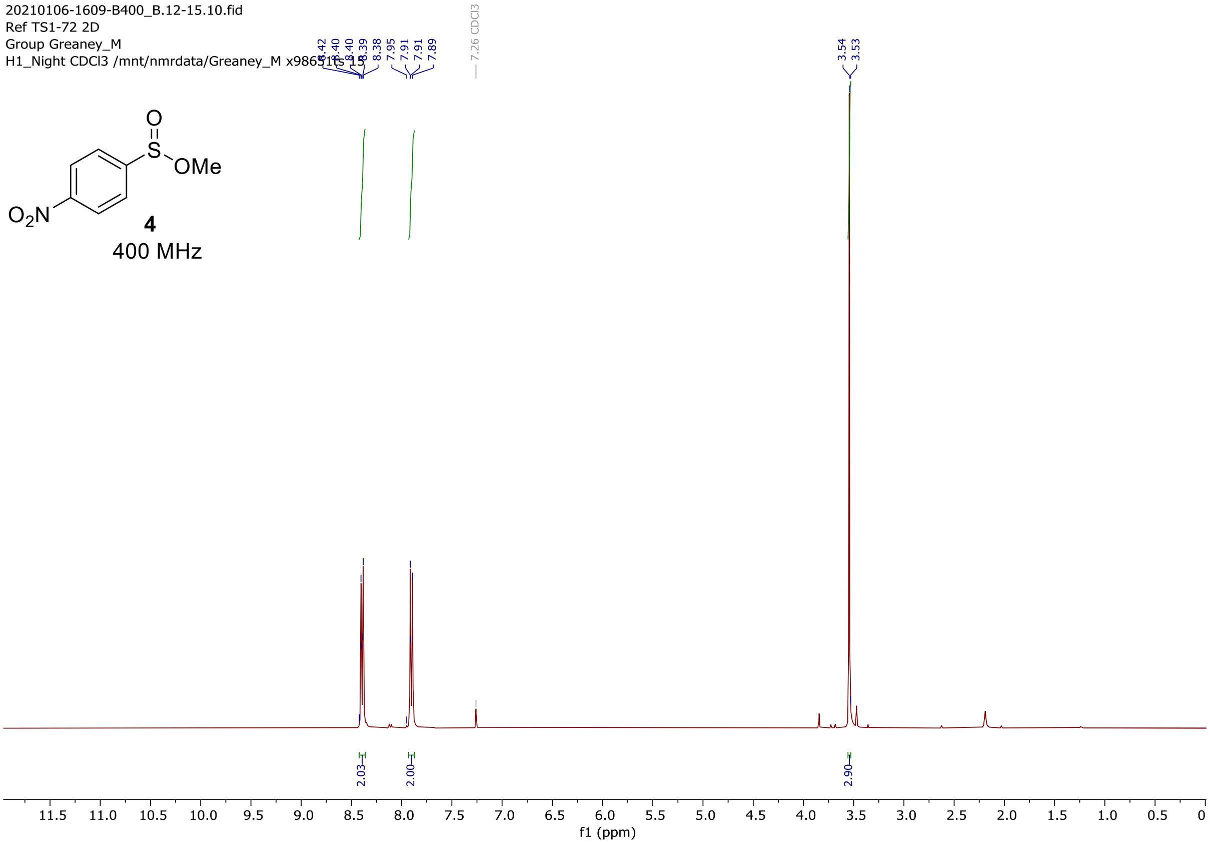

20210105-1645-B400_B.12-13.12.fid
Ref TS1-72 Carbon
Group Greaney_M
C13_CPD_Night256 CDCl3 /mnt/nmrdata/Greaney_M x98651ts 13

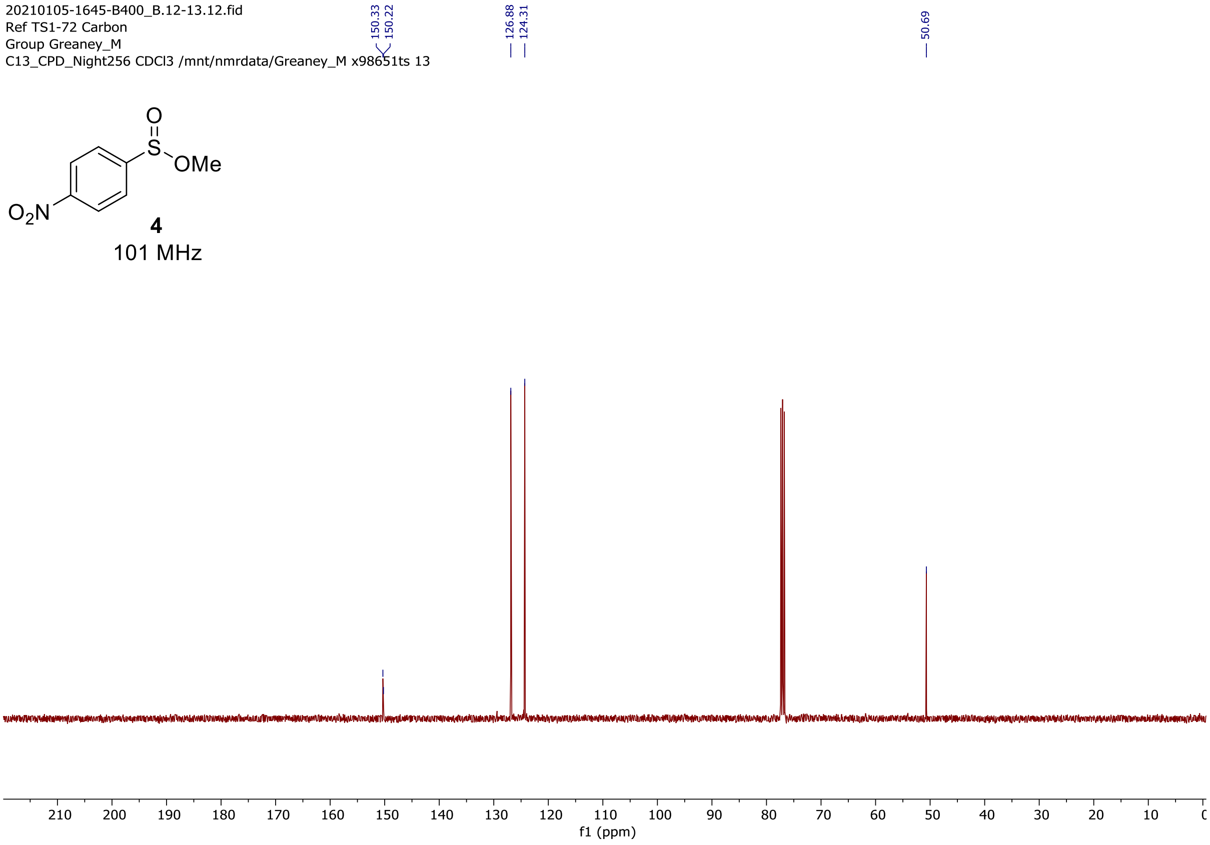

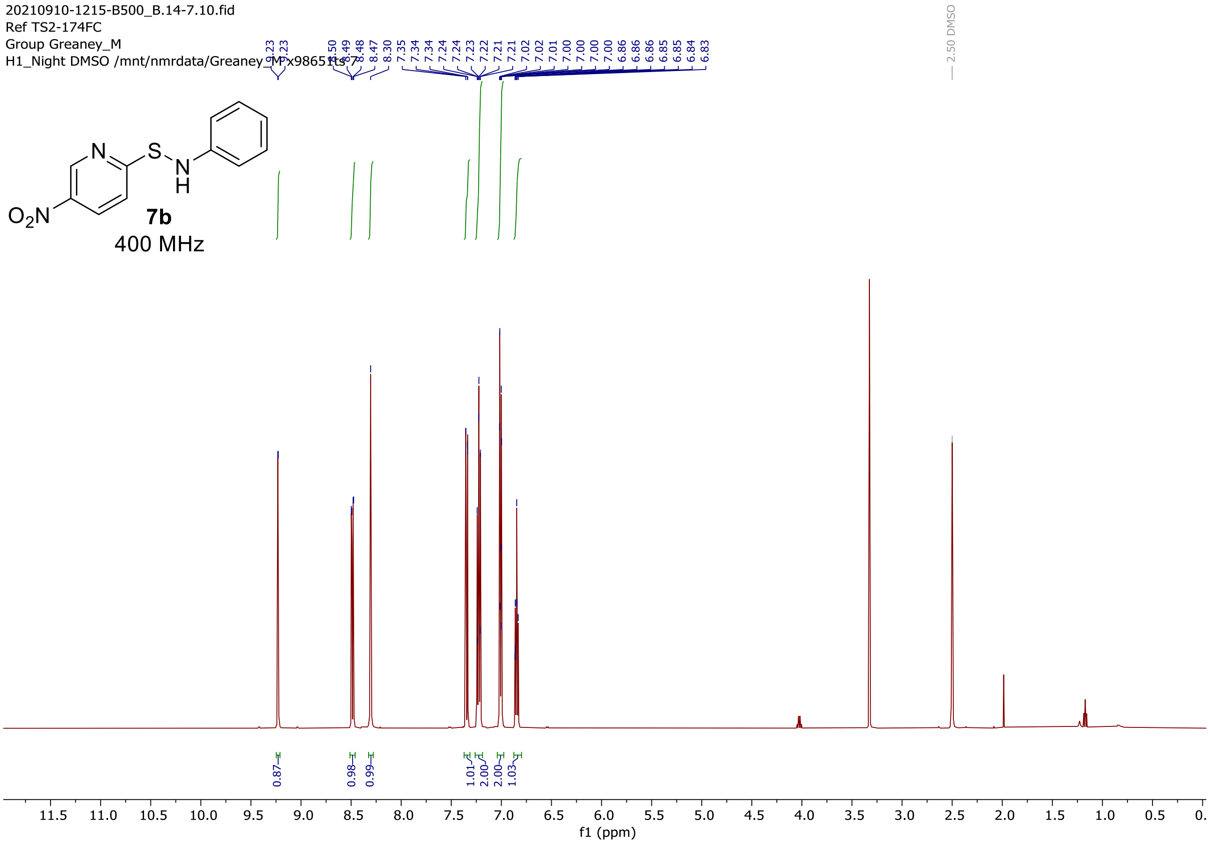
20210910-1215-B500_B.14-7.10.fid
Ref TS2-174FC
Group Greaney_M
H1_Night DMSO /mnt/nmrdata/Greaney_M x98651ts 7
9.23
9.23
8.50
8.49
8.48
8.47
8.30
7.35
7.34
7.34
7.24
7.24
7.23
7.22
7.21
7.21
7.02
7.02
7.01
7.00
7.00
7.00
6.86
6.86
6.86
6.85
6.84
6.83
2.50 DMSO
N
S
N
H
O_2N
7b
400 MHz
0.87
0.98
0.99
1.01
2.00
2.00
1.03
f1 (ppm)

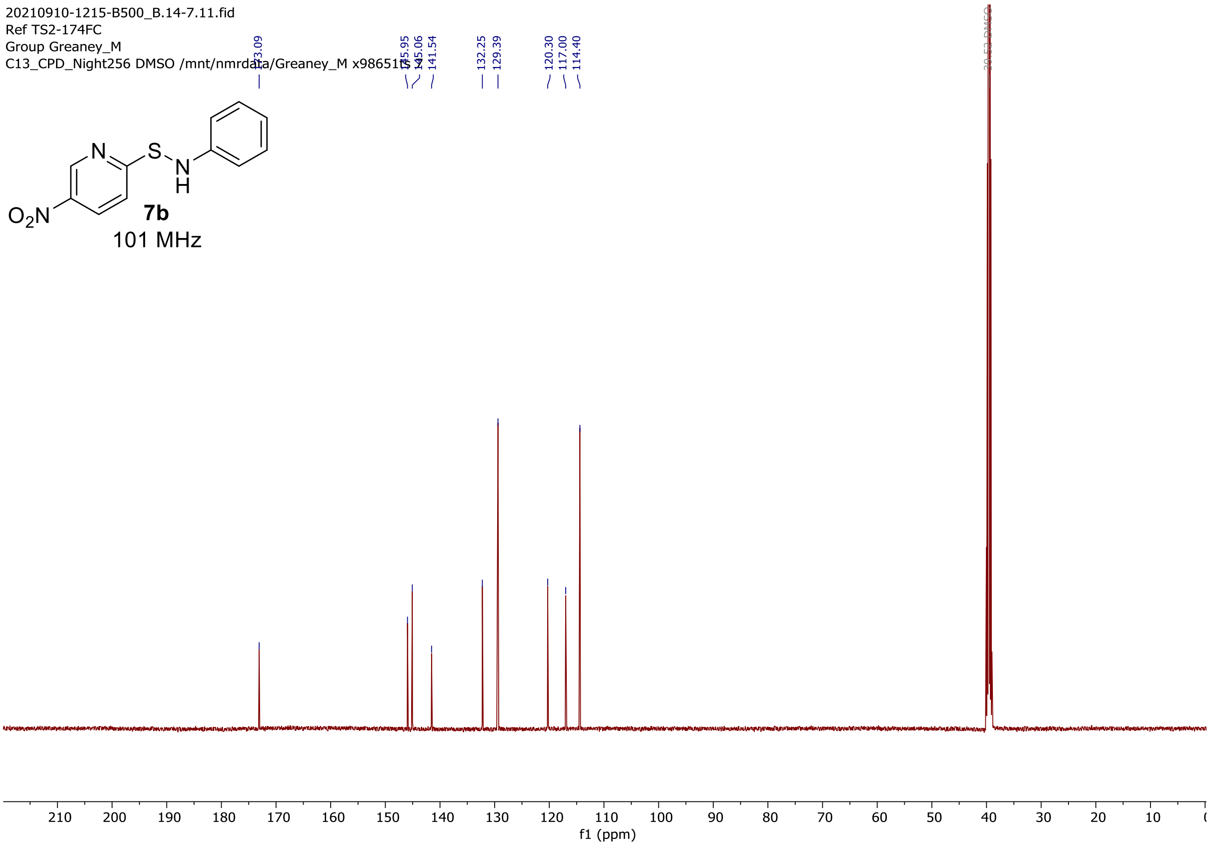
20210910-1215-B500_B.14-7.11.fid
Ref TS2-174FC
Group Greaney_M
C13_CPD_Night256 DMSO /mnt/nmrdata/Greaney_M x98651ts 8
173.09
145.95
145.06
141.54
132.25
129.39
120.30
117.00
114.40
N
S
N
H
O_2N
7b
101 MHz
f1 (ppm)

6. References

1 C. Hervieu, M. S. Kirillova, T. Suárez, M. Müller, E. Merino and C. Nevado, *Nat. Chem.*, 2021, **13**, 327–334.
2 WO2008157802 (A1), 2008.
3 X. Ding, M. Huang, Z. Yi, D. Du, X. Zhu and Y. Wan, *J. Org. Chem.*, 2017, **82**, 5416–5423.
4 M. O. Akram, A. Das, I. Chakrabarty and N. T. Patil, *Org. Lett.*, 2019, **21**, 8101–8105.
5 EP2450348 (A1), 2012.
6 Y. Fang and R. C. Larock, *Tetrahedron*, 2012, **68**, 2819–2826.
7 N. Barot, T. Shaikh and H. Kaur, *New J. Chem.*, 2017, **41**, 5347–5354.
8 J.-Q. Di, M. Zhang, Y.-X. Chen, J.-X. Wang, S.-S. Geng, J.-Q. Tang and Z.-H. Zhang, *Green Chem.*, 2021, **23**, 1041–1049.
9 M. Uchino, K. Suzuki and M. Sekiya, *Chem. Pharm. Bull. (Tokyo)*, 1979, **27**, 1199–1206.
10 M. Datta and A. J. Buglass, *Synth. Commun.*, 2012, **42**, 1760–1769.
11 M. Harmata, P. Zheng, C. Huang, M. G. Gomes, W. Ying, K.-O. Ranyanil, G. Balan and N. L. Calkins, *J. Org. Chem.*, 2007, **72**, 683–685.
12 S. W. Youn, T. Y. Ko and Y. H. Jang, *Angew. Chem. Int. Ed.*, 2017, **56**, 6636–6640.
13 Y. Shimotori, M. Hoshi, M. Murata, N. Ogawa, T. Miyakoshi and T. Kanamoto, *Heterocycl. Commun.*, 2018, **24**, 219–230.
14 P. Brownbridge and I. C. Jowett, *Synthesis*, 1988, **1988**, 252–254.
15 J. L. García Ruano, J. Alemán, C. Fajardo and A. Parra, *Org. Lett.*, 2005, **7**, 5493–5496.
16 F. A. Davis, A. J. Friedman, E. W. Kluger, E. B. Skibo, E. R. Fretz, A. P. Milicia, W. C. LeMasters, M. D. Bentley, J. A. Lacadie and I. B. Douglass, *J. Org. Chem.*, 1977, **42**, 967–972.
